# The prebiotic inulin affects virulence factor expression in *Candida albicans*

**DOI:** 10.1128/mbio.03851-25

**Published:** 2026-05-14

**Authors:** Emer Hickey, Arnab Pradhan, Qinxi Ma, Ian Leaves, Annie Philip-Brookes, Seána Duggan, Jamie A. Harvey, Ivy M. Dambuza, Paulina Cherek, Raif Yuecel, Christophe d'Enfert, Neil A. R. Gow, Gordon D. Brown, Alistair J. P. Brown

**Affiliations:** 1 MRC Centre for Medical Mycology, University of Exeter 3286, Exeter, United Kingdom; 2 Bioimaging Centre, University of Exeter 3286, Exeter, United Kingdom; 3 Exeter Centre for Cytomics, University of Exeter 3286, Exeter, United Kingdom; 4 Unité Biologie et Pathogénicité Fongiques, Institut Pasteur, Université Paris Cité 555089, Paris, France; Medizinische Universitat Graz, Graz, Austria

**Keywords:** prebiotics, inulin, *Candida albicans*, virulence factors

## Abstract

Dietary supplementation with prebiotics such as inulin has been associated with a broad range of health benefits. However, the effects of inulin on the opportunistic fungal pathogen *Candida albicans,* which resides as a commensal in the gut, have not been characterized. Here, RNA sequencing revealed that inulin affects the expression of *C. albicans* genes associated with cell wall construction, adhesion, and yeast-hypha morphogenesis. Consistent with these changes in gene expression, inulin inhibited hyphal development, increased adhesion to human Caco-2 and A431 cells, decreased the thickness of the inner layer of the *C. albicans* cell wall, reduced the exposure of cell wall pathogen-associated molecular patterns [β-(1,3)-glucan and chitin], and affected antifungal drug sensitivity. These changes impacted host immune recognition and cytokine responses, ultimately attenuating the virulence of *C. albicans* in an invertebrate infection model. Therefore, dietary supplementation with inulin is likely to influence host-fungus interactions.

**IMPORTANCE:**

The benefits of prebiotic dietary supplements, such as inulin (a natural plant dietary fiber), are thought to include a healthier gut microbiome, a reduced risk of colon cancer, and lower cholesterol levels. Unsurprisingly, prebiotic usage is increasing rapidly. However, while the effects of prebiotics upon gut bacteria have been characterized, the impacts upon *Candida albicans,* an opportunistic fungal pathogen that resides in the human gut, have remained obscure. We show that inulin affects the expression of virulence-related phenotypes and antifungal drug sensitivity in *Candida*. Furthermore, we show that inulin reduces the virulence of this fungus in an invertebrate model, consistent with the idea that inulin may lower the risk of fungal infection in healthy individuals.

## INTRODUCTION

Prebiotics are defined as non-digestible dietary ingredients that positively impact the host by enhancing the growth/activity of beneficial members of the intestinal microbiota (1, 2). The growing awareness of links between the gut microbiota and human health has driven a rapid increase in prebiotic usage that is predicted to continue into the next decade (3). In particular, the market for inulin was US$1.84 billion in 2024 and is rising by 6% year on year.

Inulin is a plant storage carbohydrate found in chicory, onions, garlic, and artichokes. This dietary fiber is a polydisperse fructan comprising chains of β-(1,2)-fructose ranging from 2 to 60 units in length, some with a terminal α-(1,2)-glucose residue (4, 5). Inulin is not catabolized efficiently by mammalian enzymes but is fermented by the gastrointestinal microbiota (6) to generate short-chain fatty acids (SCFAs) and bile acids that may account for some of the positive systemic effects of dietary inulin (7–10). Setting aside the potential hazard of fructose intolerance, the beneficial effects of dietary supplementation with inulin are reported to include the promotion of a healthy gut microbiota and bowel habits; decreased gut inflammation and infection; reduced risk of colon cancer; enhanced mineral reabsorption; lowered cholesterol levels; and enhanced immune and liver function (8, 11–14).

Dietary supplementation with inulin has been shown to promote the abundance of beneficial bacterial species in the gut in human studies (10, 15–19) and *in vitro* (20). Most of these studies report increases in *Bifidobacterium* and *Lactobacilli* species that are known to promote gut health (19, 21), but increases in *Anaerostipes*, *Bilophila,* and *Faecalibacterium* have also been observed. An *ex vivo* study suggested that *Firmicutes* and *Actinobacteria* may also degrade inulin *in vivo* (22). These investigations focused on the impact of inulin upon bacterial members of the gut microbiota. The impact upon fungi is poorly understood, and yet most individuals carry the opportunistic fungal pathogen *Candida albicans* in their gut microbiota (23–26). Furthermore, the life-threatening systemic *Candida* infections that can develop in intensive care patients (24, 27) often originate from fungal cells that colonize the gut (28, 29).

Normally, *C. albicans* exists as a harmless commensal, offering benefits to the host through the induction of protective antifungal antibodies, for example (30, 31). However, changes in the local microbiota and/or the microenvironment can trigger a transition towards *C. albicans* overgrowth and pathogenicity (23, 25, 32), including in those suffering from inflammatory bowel disease and Crohn’s disease (31, 33, 34). Yeast-hypha morphogenesis, together with the concomitant production of the toxin candidalysin and hypha-specific adhesins, are central to *C. albicans* pathogenicity (35–37). However, these virulence factors also promote *C. albicans* commensalism by protecting the fungus against mucosal shedding and by enhancing competition with the local microbiota (38–40). In addition, *C. albicans* immune evasion is enhanced *in vivo* by the masking of pathogen-associated molecular patterns (PAMPs) such as β-(1,3)-glucan in response to specific signals from the host and local microbiota (41–44). Not surprisingly, the regulation of commensalism and pathogenicity is tightly interlinked (40, 45), and subtle changes in the local environment can influence the balance between these states.

Dietary changes influence gut colonization by *C. albicans* (46, 47), and inulin supplementation has been reported to reduce gut colonization in mice (48), possibly via the enhancement of *Lactobacillus* activity and SCFA production (49, 50). However, the direct effects of inulin upon *C. albicans* cells are not known. Therefore, we tested whether inulin affects *C. albicans* phenotypes that influence the balance between commensalism and pathogenicity. Significantly, we show that inulin inhibits yeast-hypha morphogenesis, cell wall architecture, and adhesion to host cells, phenotypes closely associated with *C. albicans* virulence and commensalism. We also show that inulin reduces the virulence of *C. albicans* in the *Galleria* model.

## RESULTS

### Inulin influences the expression of *C. albicans* virulence functions

**Fig 1:**
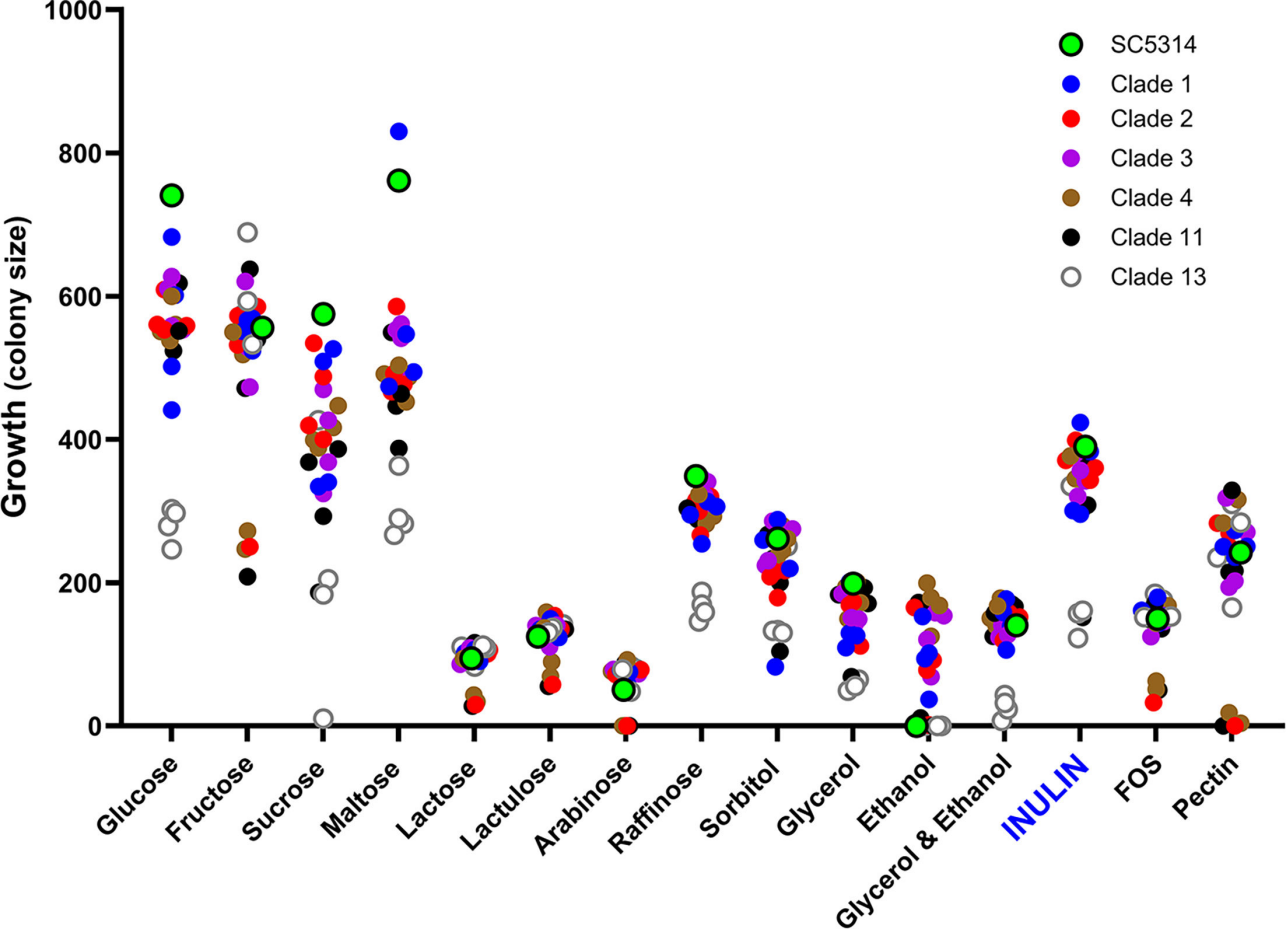
Growth of *C. albicans* clinical isolates on different carbon sources. The growth of *C. albicans* isolates on different carbohydrates as sole carbon source was assessed via spot assays on minimal agar plates. After incubation at 30°C for 72 h, the plates were imaged, and the diameters of colonies formed from the spotted suspensions were measured (pixels). Means for *n* = 6 independent replicates per isolate under each condition are presented: SC5314 (clade 1), green; other clade 1 isolates, blue; clade 2 isolates, red; clade 3 isolates, purple; clade 4 isolates, brown; clade 11 isolates, black; clade 13 isolates, white (Table S2).

Changes in carbon source affect genome-wide expression patterns in *C. albicans* (51, 52) and, consequently, virulence-related phenotypes (53). Therefore, our first objective was to explore the impact of inulin preparations on the *C. albicans* transcriptome. Before doing so, however, we first validated our test strain (the widely used isolate SC5314, clade 1) by comparing it with other epidemiologically divergent isolates in terms of their ability to grow on minimal medium containing inulin preparations as the sole carbon source. A range of additional carbon sources were tested in parallel, including glucose and fructose as controls for the fructan. As expected (54), the *Candida africana* (clade 13) strains grew less well than isolates from other clades (Fig. 1). Significantly, *C. albicans* SC5314 grew similarly to the isolates from other clades on each carbon source. Doubling times were similar for inulin and glucose, but on inulin preparations, cells reached stationary phase more quickly (Fig. S1), and therefore less growth was observed for inulin than for glucose or fructose (Fig. 1).

**Fig 2:**
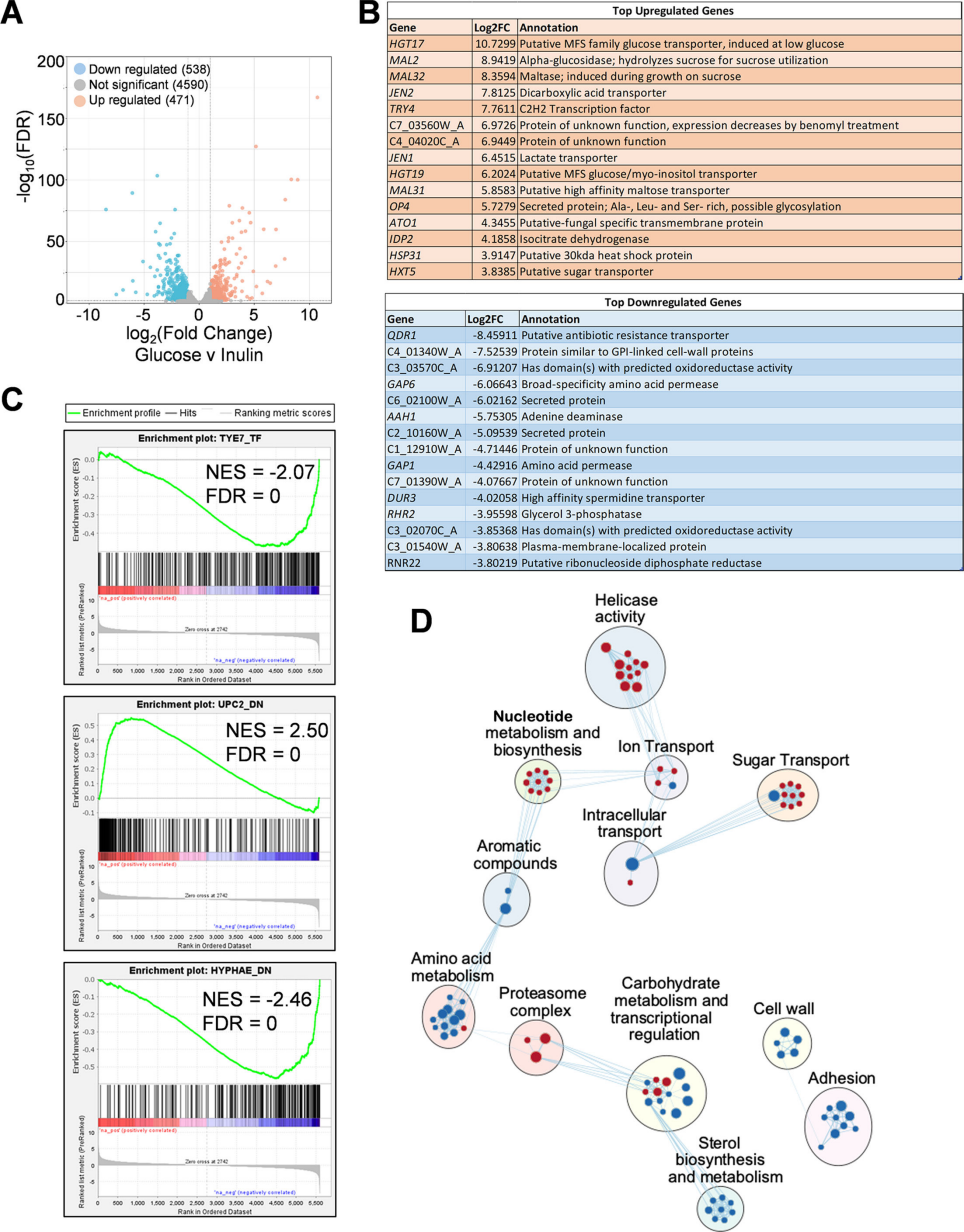
Inulin influences the expression of metabolic, cell wall, and virulence-associated genes in *C. albicans*. RNA sequencing was performed on *C. albicans* SC5314 cells grown on YNB containing inulin or glucose as the sole carbon source. DEGs were defined as those displaying significant regulation across *n* = 3 independent replicates (log_2_ fold change ≥ 1; false discovery rate [FDR] < 0.01) in response to inulin, relative to the glucose controls. (**A**) Volcano plot displaying log_2_ fold changes in response to inulin versus −log_10_ FDR, with the numbers of up- and downregulated genes in the key (top left). (**B**) The top 15 DEGs that were upregulated (pink) or downregulated (blue) in response to inulin. The complete data set is shown in Table S1. (**C**) Three of the top gene sets revealed by GSEA to be enriched among the DEGs, showing their normalized enrichment scores (NESs) and FDRs. (**D**) GSEA-generated network of functional groupings linked by DEGs that were upregulated (red) or downregulated (blue) in response to inulin.

We then explored the effects of inulin on the *C. albicans* SC5314 transcriptome. RNA sequencing was performed on triplicate cultures of cells growing at similar rates in minimal media containing inulin or glucose as the sole carbon source (Fig. S1). Transcript levels on inulin were compared to those observed on glucose (Materials and Methods; Fig. S2 and Table S1). In total, 1,009 differentially expressed genes (DEGs) were identified (log_2_ fold change ≥1 and false discovery rate <0.01): 471 *C. albicans* genes were upregulated on inulin, and 538 were downregulated (Fig. 2A).

As expected, given the change in carbon source, many of the top 15 genes that were upregulated in response to inulin are associated with carbon metabolism (Fig. 2B). The inclusion of genes involved in disaccharide (*MAL2, MAL32, MAL31*) and monosaccharide (*HGT17, HGT19, HXT5*) utilization suggested the assimilation of inulin hydrolysis products. The presence of *JEN2, JEN1,* and *IDP2* on this list was consistent with the release of glucose repression during growth on inulin. Many of the top 15 downregulated genes also encode metabolic enzymes (*AAH1*, *DUR3, GAP1, GAP6, RHR2, RNR22*) (Fig. 2B), reinforcing the view from this initial analysis that *C. albicans* retunes its metabolism in response to inulin. Glycolytic genes were downregulated in response to inulin (log_2_ fold changes: *HXK2* −1.53; *PFK1* −1.36; *PFK2* −0.61; *FBA1* −1.78; *TPI1* −1.52; *PGK1* −1.09; *GPM1* −1.68; *ENO1* −0.79; *PYK1* −2.07), and gluconeogenic genes were upregulated (*FBP1* +1.42; *PCK1* +1.46) (Table S1). Notably, our data suggest that *C. albicans* is capable of assimilating some of the mixed fructans in inulin preparations (Fig. 1; Table S1) that are likely to become available in the gut through the conversion of inulin into short-chain fructans by the local microbiota (22).

Deeper analysis of the RNA sequencing data set using Gene Set Enrichment Analysis (GSEA) (55, 56) confirmed the statistically significant enrichment of metabolic functions among inulin-regulated genes. In particular, the Tye7 and Upc2 regulons were significantly enriched among these DEGs (Fig. 2C). The bHLH transcription factor Tye7 is essential for the full induction of glycolytic genes in *C. albicans* (57): this regulon was enriched among downregulated genes (normalized enrichment score [NES] −2.07: Fig. 2C), suggesting decreased glycolytic gene expression during growth on inulin. The Zn_2_Cys_6_ transcription factor Upc2 regulates the expression of ergosterol biosynthesis and drug transporter genes (58), and genes in this Upc2 regulon were upregulated in response to inulin (NES 2.50: Fig. 2C). The deletion of *UPC2* compromises hyphal development, but the influence of Upc2 upon morphogenesis appears to be indirect. Upc2 promotes ergosterol biosynthesis, which is required for membrane and cell wall functionality and polarized growth (59–61). The influence of Upc2 upon cell wall functionality was reflected in the appearance of cell wall and adhesion genes among the GSEA network of metabolism-rich functions that were regulated by inulin (Fig. 2D). Our analyses also indicated that hypha-related genes were enriched among those repressed by inulin (NES −2.46: Fig. 2C), suggesting that inulin might inhibit yeast-hypha morphogenesis. Therefore, inulin influences the expression of key functions involved in the balance between *C. albicans* commensalism and pathogenicity.

### Inulin inhibits yeast-hypha morphogenesis

**Fig 3:**
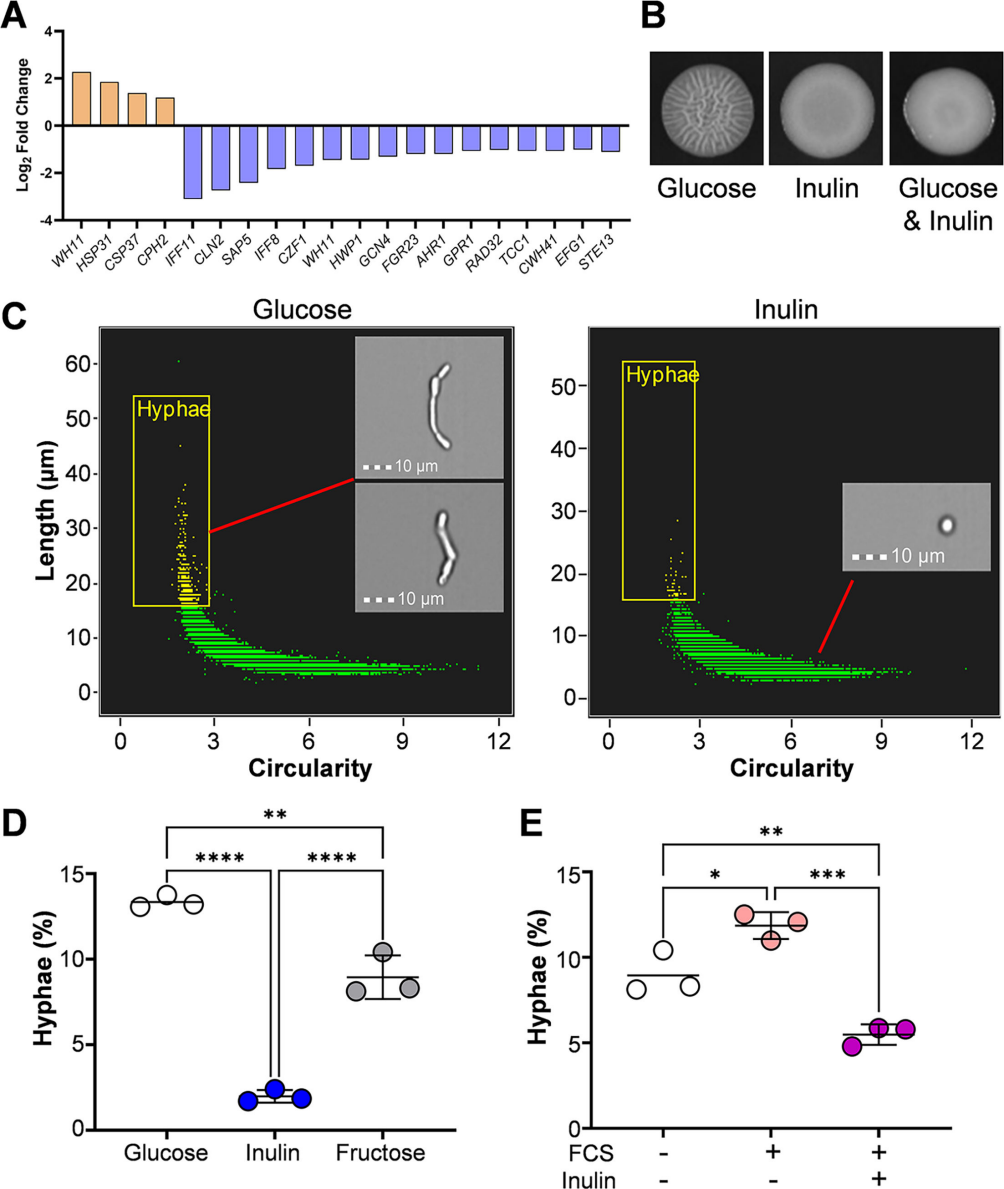
Inulin inhibits yeast-hypha morphogenesis. (**A**) DEGs associated with yeast-hypha development that were regulated in response to inulin: upregulated, pink; downregulated, blue. (**B**) Inulin inhibits the formation of wrinkly *C. albicans* SC5314 colonies when grown at 37°C on YNB plates containing either glucose, inulin, or both. (**C**) Hyphal development was quantified by imaging flow cytometry, filamentous hyphae being distinguished from ovoid yeast cells based on their length and circularity. Yellow boxes show the gating of hyphal cells, and the images show examples of gated hyphal and yeast cells. (**D**) Quantification of cells by imaging flow cytometry revealed the percentage of hyphae formed during growth at 37°C in liquid YNB containing glucose (white symbols), inulin (blue), or fructose (gray). (**E**) Percentage of hyphae formed during growth at 37°C in glucose YNB with or without fetal calf serum (FCS) and inulin: no FCS or inulin, white symbols; FCS but no inulin, pink; FCS plus inulin, purple. Means and standard deviations from *n* = 3 independent replicates are presented in panels **D** and **E**. These data were analyzed using ANOVA with Tukey’s multiple comparison test: *P* ≤ 0.05, *; *P* ≤ 0.01, **; *P* ≤ 0.001, ***; *P* ≤ 0.0001, ****.

We tested whether the downregulation of hypha-related genes by inulin (Fig. 3A) was reflected in phenotypic changes in yeast-hypha morphogenesis. First, we examined the morphology of *C. albicans* SC5314 colonies grown at 37°C on minimal media containing glucose and/or inulin as the sole carbon source. Glucose-grown colonies displayed a wrinkly morphology suggestive of hypha formation, whereas inulin-grown colonies were smooth (Fig. 3B). Interestingly, inulin blocked the formation of wrinkly colonies even in the presence of glucose (Fig. 3B).

We then quantified morphogenesis in liquid minimal media by imaging flow cytometry, gating ovoid cells versus filamentous cells (Fig. 3C). The generation of filamentous cells was significantly reduced when *C. albicans* SC5314 cells were grown at 37°C in inulin, rather than glucose or fructose (Fig. 3D). Furthermore, inulin inhibited germ tube formation in the presence of an inducer of hyphal development, fetal calf serum (FCS) (Fig. 3E). We conclude that inulin inhibits yeast-hypha morphogenesis.

### Inulin affects adhesion

**Fig 4:**
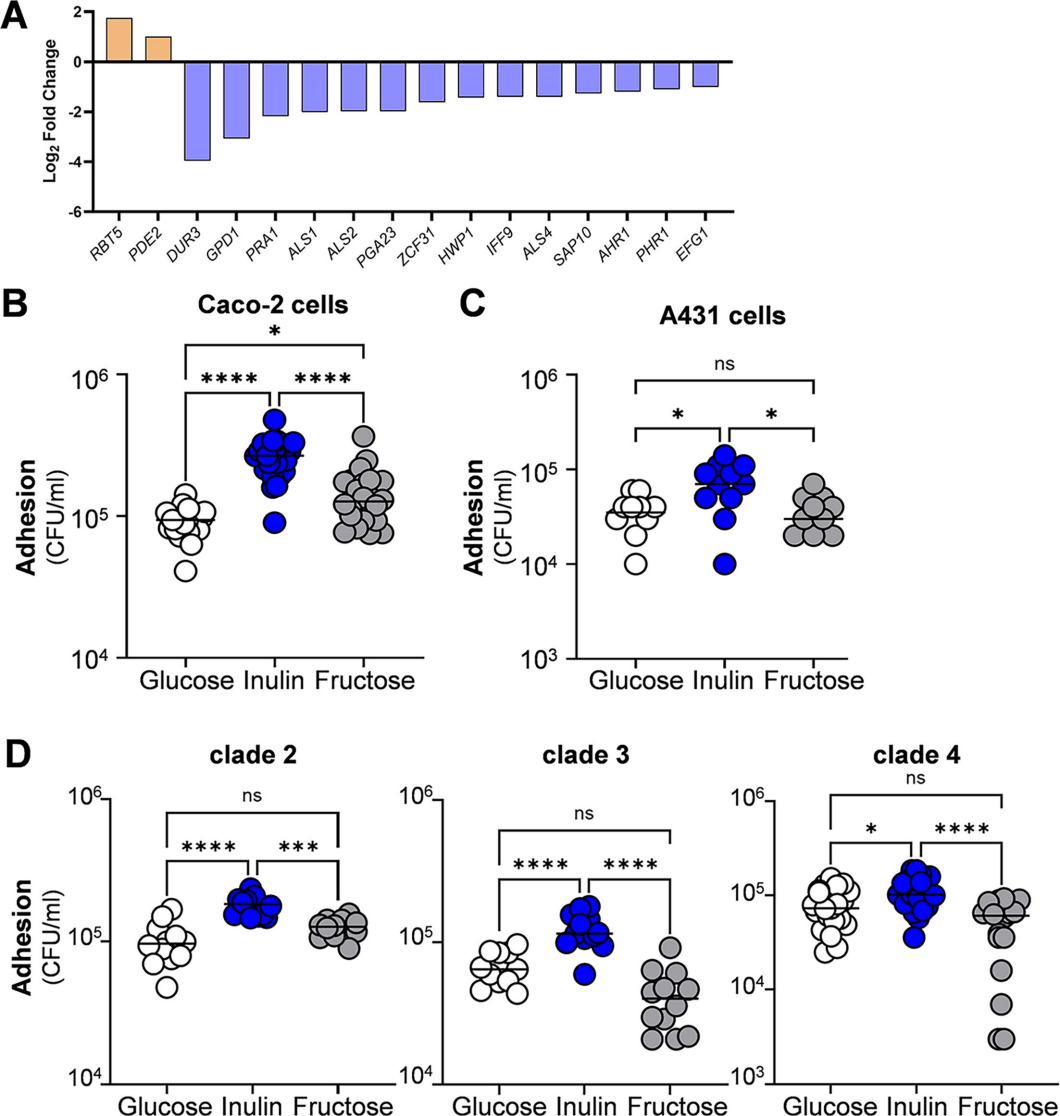
Inulin enhances adhesion. (**A**) Transcript profiling revealed inulin-regulated DEGs associated with adhesion: upregulated, pink; downregulated, blue. (**B**) *C. albicans* SC5314 cells (clade 1) were grown in YNB containing glucose (white symbols), inulin (blue), or fructose (gray), co-incubated with colonic Caco-2 cells for 1 h, and adherent cells quantified (CFUs: *n* = 24 independent replicates). (**C**) The adhesion of *C. albicans* SC5314 cells grown on glucose, inulin, or fructose to vulval A431 cells was quantified after 1 h of co-incubation (CFUs: *n* = 10 independent replicates). (**D**) *C. albicans* isolates from other epidemiological clades were grown on glucose, inulin, or fructose, and their adhesion to Caco-2 cells quantified (CFUs: *n* = 12 independent replicates): clade 2, IHEM16614; clade 3, J990102; clade 4, AM2005/0377 (Table S2). These data were analyzed using ANOVA with Tukey’s multiple comparison tests: not significant, ns; *P* ≤ 0.05, *; *P* ≤ 0.01, **; *P* ≤ 0.001, ***; *P* ≤ 0.0001, ****.

The transcript profiling revealed that inulin influences the expression of *C. albicans* genes encoding adhesion-related functions (Fig. 4A). Therefore, we tested whether inulin affects the adhesion of *C. albicans* SC5314 cells to two human cell lines: Caco-2 cells, derived from a colon carcinoma; and A431 cells, derived from an epidermal carcinoma of the vulva. Pre-growth on inulin enhanced fungal adhesion to both Caco-2 and A431 cells compared to the glucose- and fructose-grown controls (Fig. 4B and C). Focusing on the colon cell line, we confirmed that the inulin-induced increase in adhesion to Caco-2 cells was observed for *C. albicans* isolates from other major epidemiological clades (Fig. 4D).

### Inulin affects cell wall architecture and PAMP exposure

**Fig 5:**
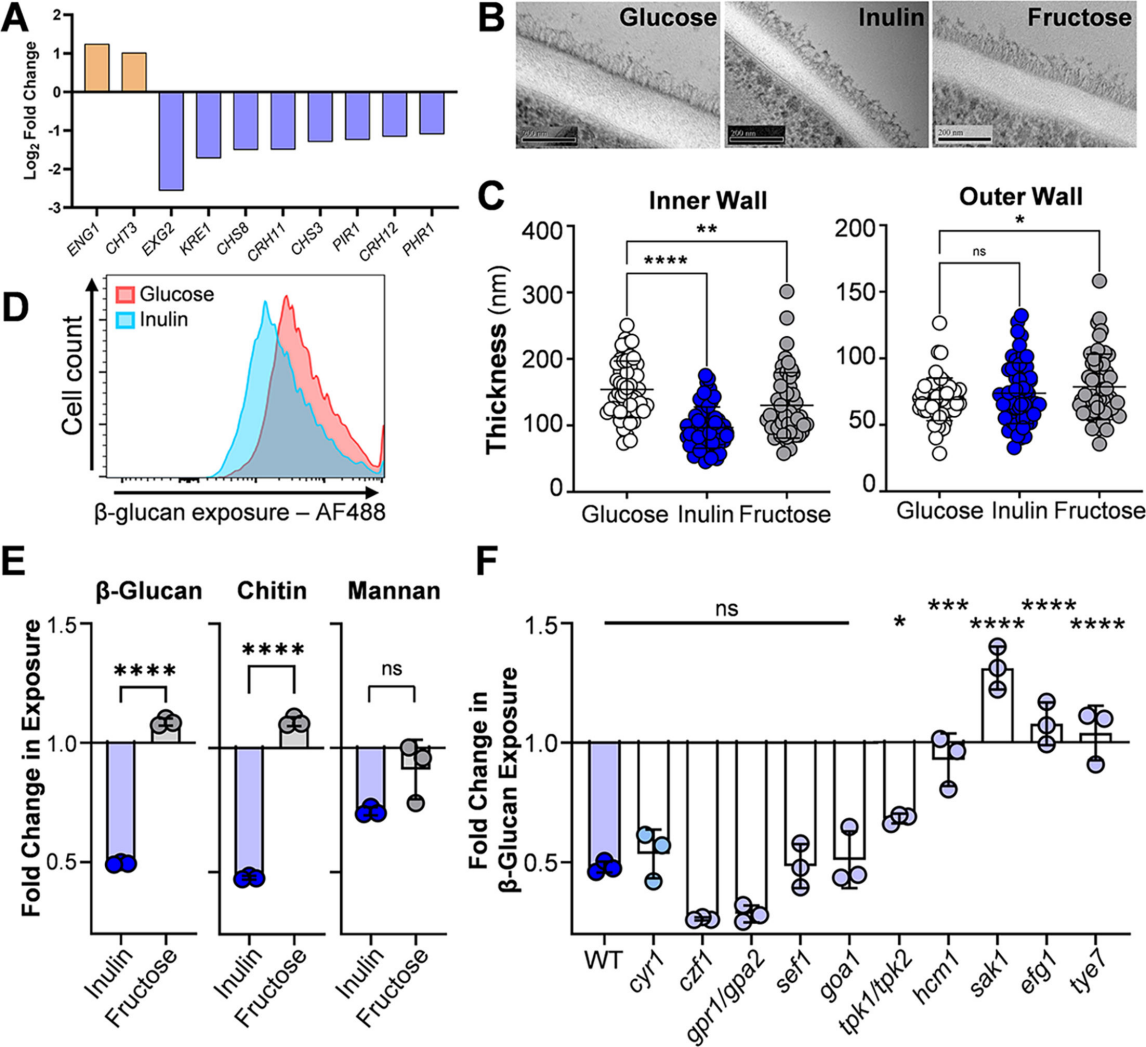
Inulin affects cell wall architecture and PAMP exposure. (**A**) Transcript profiling revealed inulin-regulated DEGs associated with cell wall construction: upregulated, pink; downregulated, blue. (**B**) TEM of cell walls of *C. albicans* SC5314 cells grown on glucose, inulin, or fructose: scale bars, 200 nm. (**C**) The thicknesses of the inner and outer cell walls were measured from TEM images using ImageJ: glucose-grown cells (white symbols); inulin (blue); fructose (gray). Means and standard deviations from *n* = 24 cells are shown. These data were analyzed using the Kruskal-Wallis test with Dunn’s multiple comparison test: not significant, ns; *P* ≤ 0.05, *; *P* ≤ 0.01, **; *P* ≤ 0.001, ***; *P* ≤ 0.0001, ****. (**D**) Cell surface exposure of β-(1,3)-glucan, chitin, and mannan on *C. albicans* SC5314 cells was quantified by flow cytometry after staining with Fc-Dectin-1, wheat germ agglutinin, or concanavalin A, respectively. The representative example of these flow cytometric analyses is for β-(1,3)-glucan exposure on cells grown on inulin (cyan) or glucose (pink) and stained with Fc-Dectin-1. (**E**) Median fluorescence indices (MFIs) were determined for each condition, and fold changes in β-(1,3)-glucan, chitin, and mannan exposure were calculated relative to the glucose control: inulin, blue (MFI_Inu_/MFI_Glu_); fructose, gray (MFI_Fru_/MFI_Glu_). Means and standard deviations from *n* = 3 independent replicates are shown. Data were analyzed using ANOVA with Tukey’s multiple comparison test: not significant, ns; *P* ≤ 0.0001, ****. (**F**) The abilities of *C. albicans* mutants to mask β-(1,3)-glucan in response to inulin were assayed by flow cytometry: WT, SC5314, dark blue circles and blue box; mutants, pale blue circles and white boxes. *C. albicans* mutants with defects in PKA signaling (*gpr1/gpa2, cyr1, tpk1/tpk2*), morphogenetic signaling (*efg1, czf1*), hypoxia-induced β-(1,3)-glucan masking (*goa1*), iron limitation-induced β-(1,3)-glucan masking (*sef1*), and metabolic regulation (*tye7, sak1, hcm1*) were examined (Table S2). Mean fold changes in β-(1,3)-glucan exposure (MFI_Inu_/MFI_Glu_) are presented. Data were analyzed using ANOVA with Tukey’s multiple comparison test: not significant, ns; *P* ≤ 0.05, *; *P* ≤ 0.01, **; *P* ≤ 0.001, ***; *P* ≤ 0.0001, ****.

Inulin also affected the expression of *C. albicans* genes encoding enzymes involved in cell wall construction (Fig. 5A). Therefore, taken together with the above effects upon morphogenesis and adhesion, we reasoned that inulin might influence cell wall architecture. We tested this by examining inulin-, glucose-, and fructose-grown *C. albicans* SC5314 cells by transmission electron microscopy (TEM) and quantifying the thicknesses of the inner and outer layers of their cell walls. The inner layer, which is composed largely of β-glucans and chitin (62), was significantly thinner in inulin-grown cells compared to the glucose- and fructose-grown controls (Fig. 5B and C). This was consistent with the reduced expression in inulin-grown cells of genes involved in chitin synthesis (*CHS3, CHS8*) and β-(1,6)-glucan synthesis (*KRE1*) and in the crosslinking, modification, and hydrolysis of cell wall polymers (*CHT3, CRH11, CRH12, ENG1, EXG1, PHR1*) (Fig. 5A). Meanwhile, inulin-grown cells displayed no significant difference in the length of their mannan fibrils in the outer cell wall layer, while those on fructose-grown cells were both longer and denser than in the glucose-grown controls (Fig. 5B and C).

Given these changes in cell wall architecture (Fig. 5B and C) and the altered expression of cell wall crosslinking functions (Fig. 5A), we tested whether growth on inulin affects the exposure of key cell wall PAMPs. Cells growing exponentially on glucose, fructose, or inulin were stained with Fc-Dectin-1, wheat germ agglutinin, or concanavalin A to reveal exposed β-(1,3)-glucan, chitin, or mannan, respectively. Median fluorescence indices (MFIs) were determined for each condition by flow cytometry (Fig. 5D), and fold changes in PAMP exposure were measured relative to the glucose-grown control. Unlike the fructose-grown controls, cells grown on inulin exposed less β-(1,3)-glucan and chitin than glucose-grown cells (Fig. 5E). This observation was consistent with the reduced thickness of the inner cell wall (Fig. 5C). We conclude that inulin-induced changes in cell wall architecture affect β-(1,3)-glucan and chitin exposure.

Lactate exposure decreases the exposure of β-(1,3)-glucan at the *C. albicans* cell surface, and this is dependent upon protein kinase A (PKA) signaling (41, 43, 63). However, when we tested whether the PKA pathway is required for inulin-induced β-(1,3)-glucan masking, we found that this phenotype was only partially blocked by inactivating Gpr1/Gpa2 (G-protein coupled receptor), Cyr1 (adenylyl cyclase), or Tpk1/Tpk2 (protein kinase A catalytic subunits) (Fig. 5F). Similarly, inulin-induced β-(1,3)-glucan masking was not dependent upon Sef1 or Goa1 (Fig. 5F), which are required for iron limitation- and hypoxia-induced β-(1,3)-glucan masking, respectively (43, 64). Instead, genes involved in metabolism, morphogenesis, and stress resistance were required for inulin-induced masking: *TYE7*, inducer of glycolytic gene expression (57); *EFG1*, regulator of yeast-hypha morphogenesis and metabolism (65); *HCM1*, forkhead transcription factor presumed to regulate respiration and assimilation of alternative energy sources (66); and *SAK1*, protein kinase acting upstream of Snf1 required for growth on alternative carbon sources (67). β-(1,3)-Glucan masking is influenced by growth in *C. albicans* (63), but growth rates on inulin and glucose were similar (Fig. S1). Therefore, the changes in β-(1,3)-glucan and chitin exposure may be mediated by the effects of the alternative carbon source on cell wall remodeling enzymes and architecture (53, 68).

### Inulin affects immune responses to *C. albicans*

**Fig 6:**
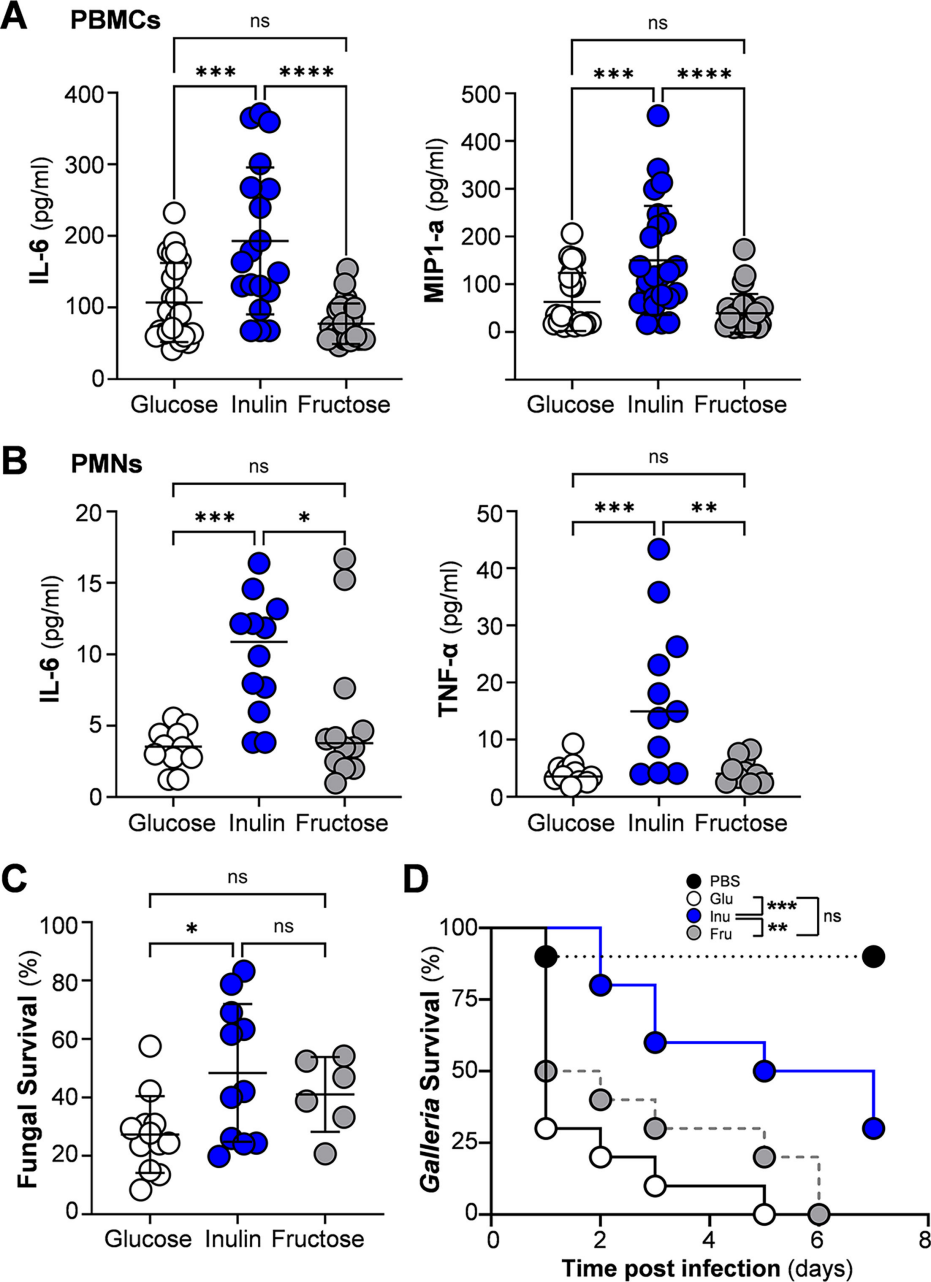
Impact of inulin upon immune responses and virulence. (**A**) Responses of human PBMCs against *C. albicans*. SC5314 cells were grown on glucose (white symbols), inulin (blue), or fructose (gray), fixed, co-incubated with PBMCs from *n* = 4 healthy volunteers (ratio of 5:1, yeast:PBMCs), and IL-6 and MIP1-α levels assayed after 12 h (*n* = 6 replicates per volunteer). (**B**) Responses of human PMNs. *C. albicans* SC5314 cells grown on glucose, inulin, or fructose were fixed and co-incubated with PMNs from *n* = 4 healthy volunteers (ratio of 5:1, yeast:PMNs). IL-6 and TNF-α levels were assayed after 12 h (*n* = 3 replicates per volunteer). Means and standard deviations are presented, and the data were analyzed using the Kruskal-Wallis test with Dunn’s multiple comparison tests: not significant, ns; *P* ≤ 0.05, *; *P* ≤ 0.01, **; *P* ≤ 0.001, ***; *P* ≤ 0.0001, ****. (**C**) Inulin increases the resistance of *C. albicans* to neutrophil killing. SC5314 cells grown on glucose, inulin, or fructose were incubated with human PMNs at a ratio of 10:1 (yeast:PMNs): *n* = 4 replicates from *n* = 3 volunteers. After 2 h, the PMNs were lysed, the viable *C. albicans* cells quantified (CFU/mL), and the percentage survival calculated relative to the corresponding control (*C. albicans* cells incubated without PMNs). Medians and standard deviations from *n* = 12 replicates are shown. These data were analyzed using ANOVA with Tukey’s multiple comparison tests: not significant, ns; *P* ≤ 0.05, *. (**D**) Inulin attenuates virulence in the *Galleria* model. *G. mellonella* larvae were infected with *C. albicans* SC5314 cells grown in glucose, inulin, or fructose (1 × 10^7^ fungal cells/mL), or with sterile PBS as a control (*n* = 10 per group). The larvae were incubated at 37°C and survival monitored for 7 days. Percentage survival is plotted, and the log-rank (Mantel-Cox) test was applied to assess statistical significance between the conditions: not significant, ns; *P* ≤ 0.01, **; *P* ≤ 0.001, ***.

The cell wall PAMPs β-(1,3)-glucan and chitin influence innate immune responses against *C. albicans* (43, 69–71). Inulin reduces the exposure of both PAMPs (Fig. 5E). However, β-(1,3)-glucan is proinflammatory (72, 73), and β-(1,3)-glucan masking in response to lactate, hypoxia, and iron limitation leads to attenuated cytokine responses against *C. albicans* (41–43, 64). In contrast, chitin can be anti-inflammatory, dampening innate immune responses (71). Therefore, we tested the effect of growing *C. albicans* on inulin on cytokine responses against the fungus. Human peripheral blood mononuclear cells (PBMCs) were prepared from the blood of four healthy volunteers and challenged with fixed *C. albicans* SC5314 cells that had been grown on glucose, inulin, or fructose. The inulin-grown cells induced higher levels of the proinflammatory cytokine interleukin-6 (IL-6), and the chemokine macrophage inflammatory protein-1 alpha (MIP1-α) compared to the glucose and fructose controls (Fig. 6A).

An analogous experiment was then performed with polymorphonuclear neutrophils (PMNs) to examine their responses to inulin-grown *C. albicans* SC5314 cells. PMNs from the four healthy volunteers produced significantly higher levels of IL-6 and tumor necrosis factor-alpha (TNF-α) when exposed to the inulin-grown fungal cells compared to their glucose and fructose controls (Fig. 6B). There was considerable variability in the responses of PBMCs and PMNs to inulin-grown cells compared to the controls (Fig. 6A and B). The basis for this was not clear. Nevertheless, both PBMCs and PMNs displayed enhanced secretion of proinflammatory cytokines/chemokines in response to the inulin-grown *C. albicans* cells (Fig. 6A and B) that no doubt reflects the balance of pro- and anti-inflammatory changes in cell wall architecture that occur in response to inulin (Fig. 5E).

The impact of inulin on the ability of *C. albicans* to survive neutrophil killing was then analyzed. PMNs from three healthy volunteers were incubated with *C. albicans* SC5314 cells for 2 h, and surviving fungal cells enumerated. Inulin-grown *C. albicans* cells displayed a slight increase in their resistance to neutrophil killing (Fig. 6C). This increase was statistically significant when compared to the glucose control, but not the fructose control. Therefore, despite enhancing inflammatory responses, inulin appears to enhance the capacity of *C. albicans* cells to endure attack by human neutrophils.

### Inulin affects virulence

The influence of inulin upon *C. albicans* virulence was tested in an invertebrate model of systemic candidiasis that is reported to reflect virulence in mammals with reasonable accuracy (74). The survival of *Galleria mellonella* larvae was monitored following infection with *C. albicans* SC5314 cells pre-grown on inulin, glucose, or fructose (Fig. 6D). In four independent experiments, the inulin-grown cells were less virulent than the glucose and fructose controls, and these differences were statistically significant. This is consistent with previous studies showing that the initial adaptive state of *C. albicans* cells can influence the outcome of virulence assays (53, 75, 76).

### Inulin influences antifungal drug resistance

**Fig 7:**
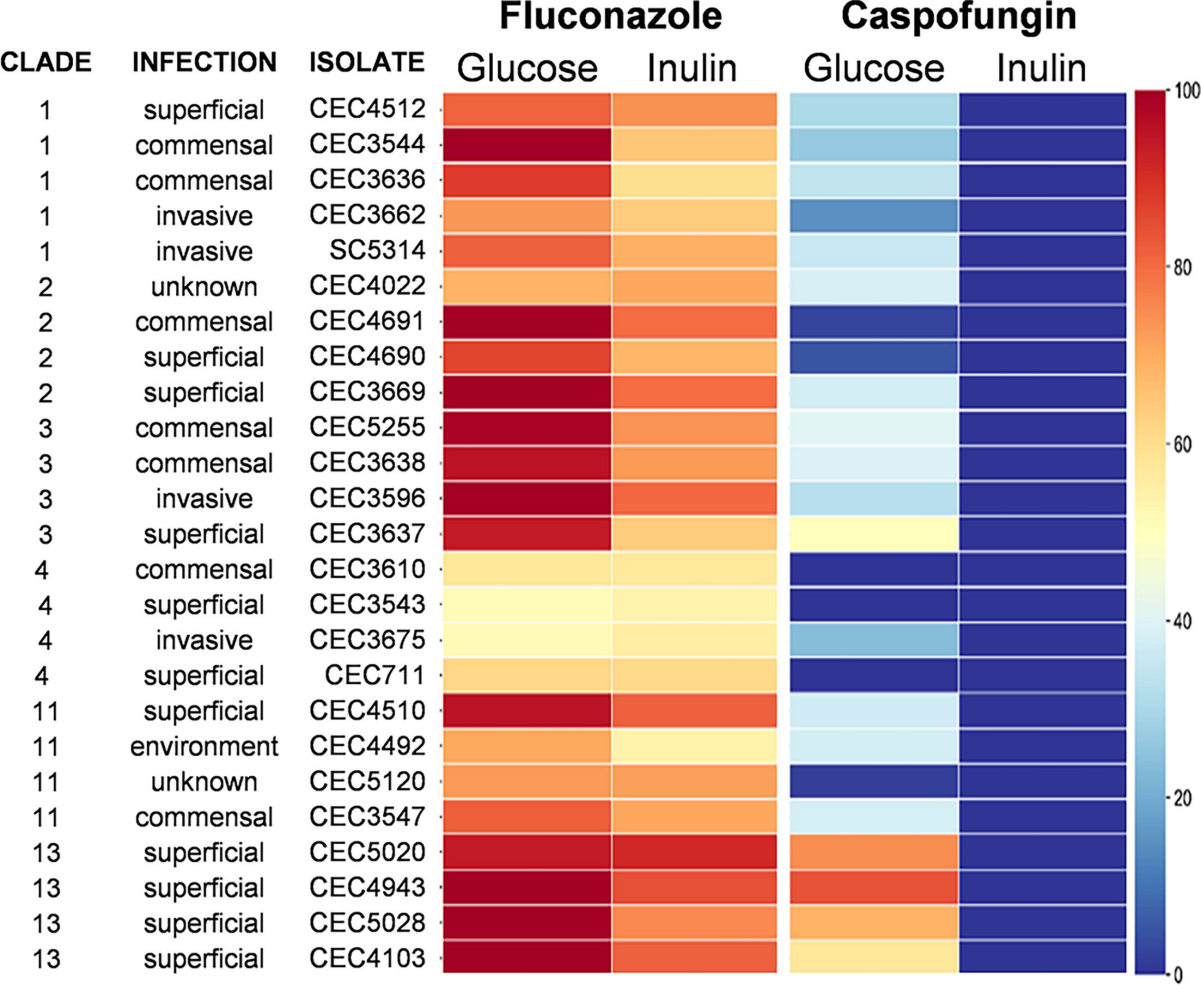
Inulin affects sensitivity to antifungal drugs. *C. albicans* isolates were replica plated onto glucose or inulin plates containing drug (0 or 0.65 μg/mL caspofungin and 0 or 0.32 μg/mL fluconazole) and incubated at 30°C for 48 h. Spots formed by each cell suspension were then imaged, and growth estimated by measuring the diameter of these spots using a Phenobooth (in pixels). For each isolate, growth in the presence of drug was divided by the growth (diameter) observed for the drug-free control, these values were expressed as a percentage, and the means for *n* = 6 independent replicate cultures were plotted in heat maps. The percentage scale is shown to the right of these heat maps: dark blue (zero growth relative to the drug-free control) up to dark red (full growth relative to the drug-free control). The clade and source (infection type) for each isolate are shown to the left (Table S2).

Inulin affected the expression of genes involved in ergosterol biosynthesis (Fig. 2) and cell wall construction (Fig. 5), the targets of azoles and echinocandins, respectively (77), and changes in carbon source are known to influence the resistance of *C. albicans* to antifungal drugs (53). Therefore, we tested whether inulin affects the sensitivity of the fungus to fluconazole or caspofungin, examining a range of clinical isolates from different infection types and divergent epidemiological clades. Rather than using standard CLSI or EUCAST assay conditions (where the fungal cells are grown on glucose), we examined the effects of inulin on drug sensitivity under conditions where either inulin or glucose was the sole carbon source. Therefore, the isolates were incubated on plates containing either inulin or glucose, plus or minus drug, and their growth quantified relative to the corresponding drug-free control. We used drug concentrations that inhibited *C. albicans* SC5314 growth by about 50% in our robotic plate assay: 0.32 μg fluconazole/mL and 0.65 μg caspofungin/mL. As expected given their known phenotypic heterogeneity (25, 78, 79), these *C. albicans* clinical isolates displayed variability with respect to their sensitivities to both fluconazole and caspofungin (Fig. 7). Nevertheless, irrespective of the type of infection or the epidemiological clade, the sensitivity of most isolates to both drugs was elevated in response to inulin.

## DISCUSSION

Mounting evidence suggests that fungi occupying intestinal niches respond to the host’s diet (80). Western diets, often low in fiber and high in sugars, salt, and saturated fats (81), are associated with reduced microbial diversity and higher levels of *C. albicans* colonization in the gut compared to non-Western societies (82). This is a concern as life-threatening systemic candidiasis infections often arise from the gut (28). Dietary supplementation with prebiotics such as inulin has been shown to promote the growth of beneficial gut bacteria in healthy individuals (10, 15–19), and might also be expected to reduce *C. albicans* colonization.

Our data indicate that inulin affects *C. albicans* phenotypes central to the balance between commensalism and virulence. Genome-wide transcript profiling suggested changes in *C. albicans* carbon metabolism that included assimilation of disaccharides and monosaccharides, which would be generated from this mixed fructan by the gut microbiota. The RNA sequencing also revealed inulin-responsive genes involved in ergosterol metabolism, cell wall construction, adhesion, and yeast-hypha morphogenesis. Corresponding phenotypic changes were observed, including changes in fungal sensitivity to fluconazole and caspofungin across a wide range of clinical isolates. Significantly, inulin reduced but did not block hyphal development in *C. albicans* SC5314. While isolates vary with regard to the strength of their morphogenetic responses (25, 39, 83), we would expect inulin to attenuate hypha formation in most isolates. This would be consistent with the promotion of *C. albicans* commensalism *in vivo* as modest levels of hyphal development and the hypha-specific toxin, candidalysin, are required for fungal retention in the oral mucosa and gut (38–40). Inulin also induced changes in cell wall architecture that were reflected in increased adhesion to human colon cells and decreased exposure of major cell wall PAMPs: β-(1,3)-glucan, which is pro-inflammatory, chitin, which is anti-inflammatory. Other cell wall components that are recognized by pattern recognition receptors (69, 70, 84) may also have been affected. For example, the mannan fibrils on inulin-grown cells were less well organized than those on glucose-grown cells (Fig. 5B). Overall, these inulin-induced changes enhanced the proinflammatory responses of human PBMCs and PMNs against the fungus. Nevertheless, human neutrophils were less effective at killing inulin-grown *C. albicans* cells, potentially because inulin enhances resistance to certain stresses.

The inactivation of *TYE7, HCM1,* or *SAK1* compromised inulin-induced β-glucan masking (Fig. 5F). As mentioned, *C. albicans* Tye7 and Sak1 are reported to regulate metabolism (57, 67) and, by inference, Hmc1 is thought to regulate genes associated with respiration and the utilization of alternative carbon sources (66) rather than morphogenesis (85). As far as we are aware, there is no evidence indicating that Tye7, Hmc1, or Sak1 are regulated directly by PKA, or vice versa, which suggests that they may lie on parallel signaling pathways to PKA in *C. albicans*. This appears to be the case for the transcription factors Sfl1 and Sfl2, for example (86). Therefore, it is not inconceivable that the inulin-related phenotypes we observed may have been manifested indirectly via metabolic adaptation, which then led to changes in the cell wall and hyphal development, thereby influencing adhesion and innate immune responses.

Hypha-related phenotypes, adhesion, PAMP exposure, and innate immune responses influence the balance between virulence and commensalism (25, 35, 39–41, 87, 88), and therefore we reasoned that inulin might influence this balance. Indeed, growth on inulin reduced the virulence of *C. albicans* SC5314 cells in *Galleria* larvae. *C. albicans* isolates display variability in their virulence (25, 39, 40), and it remains to be tested whether inulin reduces the virulence of most isolates under similar experimental conditions. In the future, it would also be valuable to examine the effects of dietary supplementation with inulin upon gut colonization. Ideally, these experiments should be performed using clinical isolates which, unlike *C. albicans* SC5314, can colonize the murine gut without prior antibiotic treatment to compromise the local microbiota (89).

It should be borne in mind that clinical studies of prebiotic supplementation have generally been performed on healthy individuals (10, 15–19), and yet life-threatening systemic candidiasis infections generally occur in those with compromised immune systems (27). Furthermore, phenotypic responses to prebiotics are influenced by host genetics (90), the shape of the gut microbiota, which varies significantly between individuals (91), and the dose of the prebiotic. Also, while recent clinical studies predominantly focus on the impact of prebiotics on bacterial members within the gut, our data show that a commonly used prebiotic influences virulence attributes in a major fungal pathogen. This suggests potential risks for prebiotic usage in patients with compromised immunity or dysbiotic guts who may be at risk of fungal disease. Prebiotics have been proposed as a possible therapeutic approach for patients with inflammatory bowel disease (IBD) (92). Fungi are known to shape the gut microbiota (93) and can exacerbate inflammation in the gut (34, 94). Furthermore, *C. albicans* is thought to enhance inflammation in IBD patients via the hypha-specific toxin, candidalysin (34, 92, 95). Inulin inhibits hypha formation (Fig. 3) and hence would be expected to reduce candidalysin production (35). However, while dietary supplementation with the prebiotic inulin enhances immunological conditioning in healthy individuals (14), it is difficult to predict whether this would offer therapeutic value to IBD patients given the complexity of gut microenvironments, human population heterogeneity, and the range of inulin-related phenotypes we observed *in vitro*. Further studies would be required to assess this, possibly including clinical trials in IBD patients.

## MATERIALS AND METHODS

### Strains and growth conditions

*C. albicans* strains used in this study are listed in Table S2. *C. albicans* cells were grown in YNB (0.67% yeast nitrogen base without amino acids) containing 2% of the stated carbon source at 30 or 37°C and 200 rpm. Growth was monitored (OD_600_), and cells were harvested in exponential phase for analysis.

To compare the growth of clinical isolates, *C. albicans* strains were grown in glucose-YNB, harvested in exponential phase, washed thrice, resuspended in sterile water (OD_600_ = 0.8), and cell suspensions seeded into 96-well plates. Using a Singer Rotor+ robot, these *C. albicans* cell suspensions were replica plated onto YNB agar plates containing different carbon sources, and the plates incubated at 30°C for 72 h. Plates were imaged and the diameters of spotted suspensions measured using a Phenobooth (*n* = 6 independent replicates, each from a different culture).

Stock solutions of 10% chicory inulin (Sigma-Aldrich) were prepared fresh for each experiment. Inulin was dissolved in Milli-Q water for 20 min at 50°C, autoclaved at 121°C for 15 min, and stored at room temperature for up to 2 days.

### RNA sequencing

Exponentially growing *C. albicans* SC5314 cells were harvested after 3 h of growth in fresh inulin-YNB or glucose-YNB (Fig. S1). RNA was isolated from *n* = 3 independent replicate cultures using the Ribopure RNA Purification Kit, Yeast (Invitrogen), according to the manufacturer’s instructions, shearing cells for 10 min with zirconia beads in an MP FastPrep 24 bead beater. RNA samples were treated with DNase I, transferred to fresh tubes, and stored at −80°C. RNA yields were measured using Qubit RNA BR Assay Kits (Invitrogen), and RNA integrity was assessed using a TapeStation (Agilent), RIN scores of >8 deemed acceptable for sequencing.

RNA sequencing was performed on an Illumina NovaSeq 6000 by the Sequencing Facility at the University of Exeter, generating >1.4 × 10^7^ reads per sample (*n* = 3 replicates per condition), and the data analyzed on the BMK Cloud Bioinformatics Analysis Platform. Sequences were aligned to the *C. albicans* SC5314 Assembly 22 (candidagenome.org) using HISAT2 and StringTie (96). The data set was evaluated via randomness inspection, fragment length distribution and saturation inspection, and normalized expression levels expressed in FPKM (fragments per kilobase per million mapped reads). DESeq2 was used to define genes that were differentially expressed in response to inulin, relative to glucose controls (log_2_ fold change ≥ 1; FDR < 0.01). GSEA (55, 56) was used to generate enrichment plots and NESs for gene sets that were significantly enriched among the DEGs. GSEA outputs were then used to generate a network of functional groupings linked by inulin-responsive DEGs in Cytoscape. The RNA sequencing data set is presented in Table S1 and available at https://www.ebi.ac.uk/biostudies/ArrayExpress/studies/E-MTAB-15594?query=E-MTAB-15594 (accession number: E-MTAB-15594).

### Cell wall PAMPs

The exposure of cell wall PAMPs was quantified by flow cytometry using published procedures (41, 64, 79). Briefly, *C. albicans* cells were grown at 37°C in glucose-, inulin-, or fructose-containing YNB overnight and then regrown in fresh media for 3 h (*n* = 3 independent replicates per condition). These exponentially growing cells were harvested, fixed with 50 mM thimerosal (Sigma-Aldrich), and washed. Cells were stained with Fc-Dectin-1 to quantify β-(1,3)-glucan exposure, or with concanavalin A or wheat germ agglutinin to examine mannan and chitin exposure, respectively. The fluorescence of 10,000 cells was acquired using an Attune NxT flow cytometer, and MFIs were determined using FlowJo software v.10.8.1. Fold changes in exposure were calculated relative to the glucose control (e.g., MFI_Inu_/MFI_Glu_).

### Yeast-hypha morphogenesis

Yeast-hypha morphogenesis was examined indirectly by analyzing the morphology of *C. albicans* SC5314 colonies after 72 h growth at 37°C on YNB plates containing glucose, inulin, or both.

Hyphal development was quantified directly by imaging flow cytometry. *C. albicans* SC5314 cells were grown for 1.5 h at 37°C either in YNB containing glucose, inulin, or fructose, or in YNB containing these carbon sources with or without 3% (vol/vol) FCS (*n* = 3 independent replicates per condition). Cells were then fixed with thimerosal, stained with 5 μg/mL Calcofluor White for 5 min in the dark, washed thrice with PBS, and resuspended in PBS containing 2 mM ethylenediaminetetraacetic acid. Cell populations were analyzed (>10,000 events) using an Amnis ImageStream MKII Imaging Flow Cytometer (Luminex). Yeast cells and germ tubes (developing hyphae) were gated using IDEAs v.6.3 software (Fig. S3), and the proportion of germ tubes versus yeast cells quantified (97).

### Adhesion

Human A431 and Caco-2 cell lines were cultured in RPMI plus 10% FCS and DMEM plus 10% FCS, respectively, at 37°C under 5% CO_2_. Confluent monolayers in 24-well plates were incubated with 10^5^ *C. albicans* cells for 1 h in fresh culture medium containing 10% FBS at 37°C under 5% CO_2_. The medium was then aspirated from wells, the wells rinsed thrice with PBS to remove non-adhering *C. albicans* cells and, after vortexing, adherent fungal cells quantified (CFUs) by plating on YPD agar (*n* > 10 independent replicates).

### Transmission electron microscopy

Exponential cells grown in YNB supplemented with glucose, inulin, or fructose were harvested using polycarbonate filters, promptly transferred to aluminum planchettes (0.1 mm depth), and subjected to high-pressure freezing using a HPM Live µ, CryoCapCell according to published methods (98). Samples were transferred under liquid nitrogen into cryotubes containing freeze substitution medium (1% osmium tetroxide and 0.5% glutaraldehyde in 100% acetone) pre-cooled in liquid nitrogen. Samples were transferred onto a rotary shaker, incubated for 3 h under dry ice, and then removed from dry ice and brought slowly up to room temperature, with shaking, over 1 h. Samples were then washed thrice in 100% acetone for 5 min and embedded in Epon resin at 60°C for 24 h. Ultrathin sections (60 nm) were prepared using an ultramicrotome (Leica EM UC7), collected on pioloform-coated 100-mesh copper grids, and post-stained with lead citrate. Samples were imaged using a JEOL 1400 JEM transmission electron microscope with a digital camera (ES1000W, Gatan, Ametek, Abingdon, UK). Sections of the *C. albicans* cell wall were selected for imaging at random at a magnification of 100,000 times. At least 30 cells were imaged per condition, and the thicknesses of the inner and outer layers of the cell wall determined (30 measurements per cell) using the line tool in ImageJ.

### Neutrophil killing

PMNs were prepared from healthy volunteers (*n* = 3) as described (99). Exponential *C. albicans* SC5314 cells were harvested after 3 h of growth in YNB containing glucose, inulin, or fructose. The fungal cells and PMNs were incubated together (10:1, yeast:PMNs) for 2 h, the PMNs lysed, and viable *C. albicans* cells quantified (CFU/mL) by plating on YPD agar (*n* = 4 per volunteer). Percentage survival was calculated relative to the control (*C. albicans* incubated without PMNs).

### Cytokine responses

*C. albicans* cells were grown to exponential phase in glucose-, inulin-, or fructose-containing YNB, harvested, fixed with 50 mM thimerosal, and washed, as described above for the analysis of cell wall PAMPs. Meanwhile, human PBMCs and PMNs were prepared from the blood of healthy volunteers as described previously (41, 99). Duplicate samples of PBMCs or PMNs from *n* = 4 individuals were mixed with *C. albicans* cells at a ratio of 5:1 (yeast:PBMCs), co-incubated for 12 h, and then cytokine levels assayed: IL-6 and MIP1-α for PBMCs; and TNF-α for PMNs. Cytokine production was assayed using ProcartaPlex multiplex immune assay kits (Invitrogen), and the data were analyzed using Luminex xMap technology.

### Fungal virulence

Virulence was first evaluated using the *G. mellonella* invertebrate model of systemic candidiasis (74). Exponential *C. albicans* cells were harvested from glucose-, inulin-, or fructose-YNB cultures, washed, and resuspended at 10^5^ cells/mL in PBS. *G. mellonella* larvae (UK Waxworms; Sheffield, UK) were injected via their the last proleg with 10 μL of fungal suspension (*n* = 10 larvae per condition). The larvae were incubated at 37°C and monitored daily for survival.

### Statistical analyses

Statistical analyses were performed in GraphPad Prism 9. Gaussian distributions were assessed to determine if the data were parametric or non-parametric. The Shapiro-Wilks test was used when the sample size was <50, and the Kolmogorov-Smirnov test was used for larger sample sizes. Parametric data consisting of two test groups were analyzed using the unpaired or paired *t*-test. One-way or two-way ANOVA tests were applied for parametric data with more than two test groups. TEM data were analyzed using the Kruskal-Wallis test with Dunn’s multiple comparison tests. The Mann-Whitney *U*-test was used to test the statistical difference between two sets of data with a non-parametric distribution. The following *P*-values were considered: *P < 0.05; **P < 0.01; ***P < 0.001; ****P < 0.0001.

## Data Availability

Data supporting the observations in this study are available within the article and its supplemental files. The RNA sequencing data are presented in Table S1 and are available at https://www.ebi.ac.uk/biostudies/studies/ (accession number: E-MTAB-15594).

## SUPPLEMENTAL MATERIAL

Figure S1 mbio.03851-25-s0001.pdf Growth on inulin.

Figure S2 mbio.03851-25-s0002.pdf RNA sequencing.

Figure S3 mbio.03851-25-s0003.pdf Gating strategy.

Table S1 mbio.03851-25-s0004.pdf Transcript profiling data set.

Table S2 mbio.03851-25-s0005.pdf *Candida* strains.

ASM does not own the copyrights to Supplemental Material that may be linked to, or accessed through, an article. The authors have granted ASM a non-exclusive, world-wide license to publish the Supplemental Material files. Please contact the corresponding author directly for reuse.
