# Supplementary figures and images for: The prebiotic inulin affects virulence factor expression in Candida albicans
Source: mBio. 2026 May 14;17(6):e03851-25. doi: 10.1128/mbio.03851-25 (PMC13251390; doi:10.1128/mbio.03851-25)

# Supplementary Figure 1

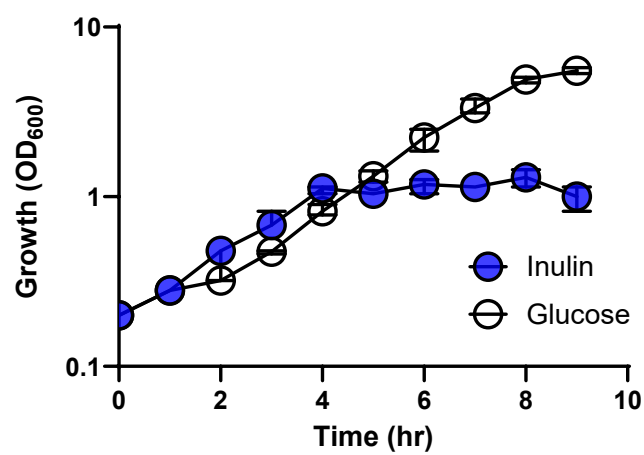

Figure S1. Growth of *C. albicans* SC5314 on YNB containing inulin or glucose.

Supplement: Figure S1 — Growth on inulin. [file mbio.03851-25-s0001.pdf]
