# Supplementary material for: The prebiotic inulin affects virulence factor expression in Candida albicans
Source: mBio. 2026 May 14;17(6):e03851-25. doi: 10.1128/mbio.03851-25 (PMC13251390; doi:10.1128/mbio.03851-25)
Supplement: Figure S2 — RNA sequencing. [file mbio.03851-25-s0002.pdf]

Supplementary Figure 2

A

Samples	Clean reads	Clean bases	GC Content	%≥Q30
G1	16,458,040	4,937,412,000	40.18%	93.93%
G3	26,573,958	7,972,187,400	40.87%	93.33%
G4	16,192,857	4,857,857,100	40.19%	93.77%
i1	14,119,636	4,235,890,800	40.13%	93.53%
i2	18,511,650	5,553,495,000	40.28%	92.13%
i3	15,549,336	4,664,800,800	40.66%	94.04%

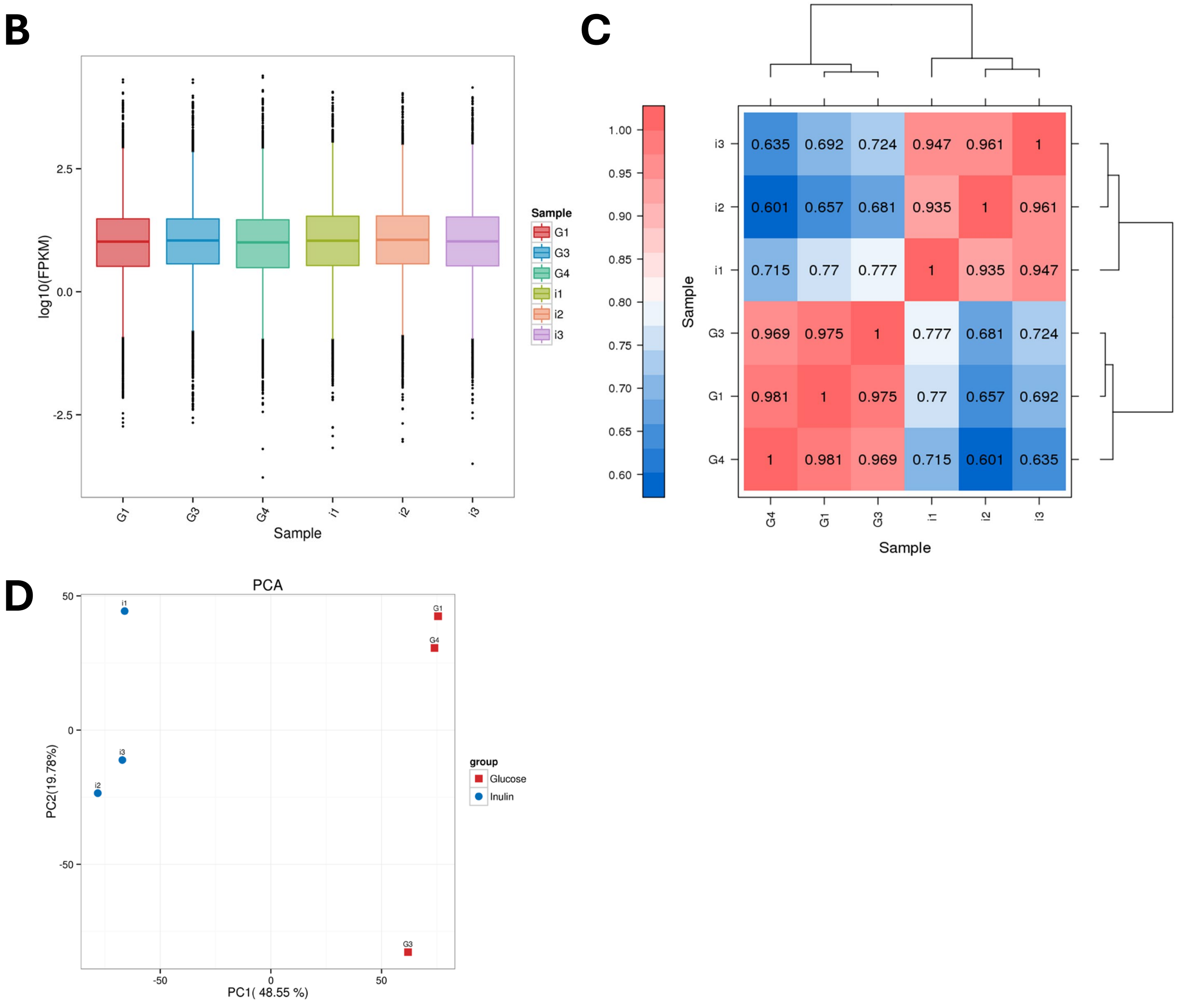

Figure S2. RNA sequencing.

(A) Over 14 million clean sequencing reads were generated for each RNA sample: G, glucose; I, inulin. Over 43 Gb of clean data were obtained, and over 92 % of bases in each sample had a Q-score of ≥30. **(B)** Fragments per kilobase of transcript per million mapped fragments (FPKM) for each replicate sample. Box plots display the range of gene expression observed within and between the independent replicates. **(C)** Correlation heatmap between the independent replicates. **(D)** Principal components analysis showing the relatedness between the independent replicates.
