# Supplementary material for: The prebiotic inulin affects virulence factor expression in Candida albicans
Source: mBio. 2026 May 14;17(6):e03851-25. doi: 10.1128/mbio.03851-25 (PMC13251390; doi:10.1128/mbio.03851-25)
Supplement: Figure S3 — Gating strategy. [file mbio.03851-25-s0003.pdf]

Supplementary Figure 3

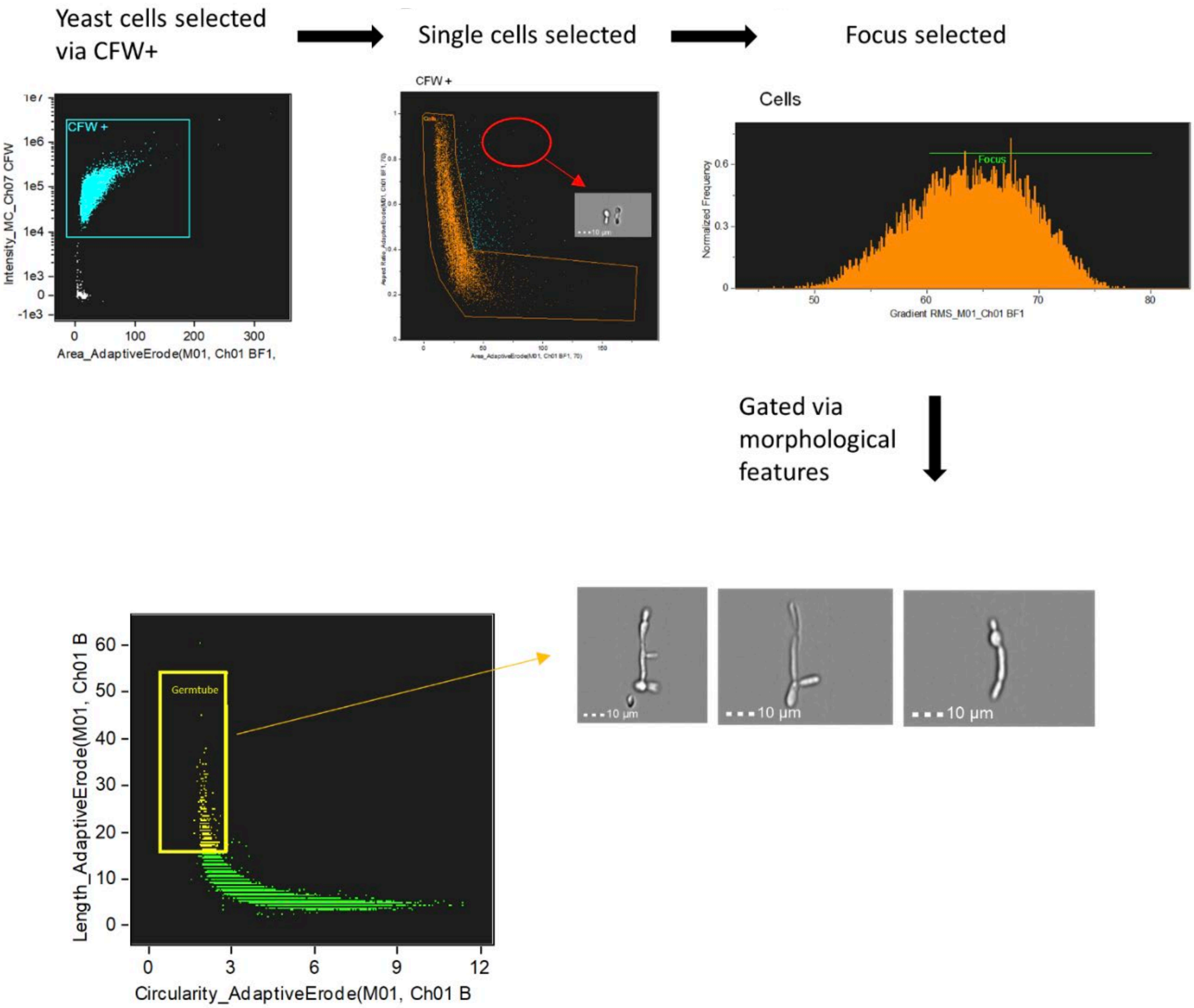

Figure S3. Imaging flow cytometry gating strategy used to determine the percentage germ tube formation in a population of cells.

Fungal cells were gated via the identification of calcofluor white positive (CFW+) cells. Single cells were selected and then focused cells were selected via the creation of a focus gate using the gradient RMS feature. Cells forming germ tubes were gated by plotting length versus circularity. Analyses were performed using IDEAs.
