# Supplementary material for: The prebiotic inulin affects virulence factor expression in Candida albicans
Source: mBio. 2026 May 14;17(6):e03851-25. doi: 10.1128/mbio.03851-25 (PMC13251390; doi:10.1128/mbio.03851-25)
Supplement: Table S1 — Transcript profiling data set. [file mbio.03851-25-s0004.pdf]

Table S1. Transcript profiling dataset

	FKPM (Fragments per Kilobase Per Million mapped reads)							
	Glucose			Inulin				
GENE #ID	Rep 1	Rep 2	Rep 3	Rep 1	Rep 2	Rep 3	FDR	log2FC
C4_01070W_A	0.08	0.02	0.17	183.95	183.51	194.35	1.18E-167	10.73
CR_10790W_A	0.51	1.62	1.10	519.97	667.97	552.18	7.78E-101	8.94
C5_04940W_A	0.43	0.35	0.36	160.71	75.92	174.54	4.14E-101	8.36
C4_04030W_A	0.13	0.38	0.50	91.57	72.33	87.27	1.11E-84	7.81
C3_05050W_A	0.05	0.07	0.11	21.33	16.91	21.61	1.36E-36	7.76
C7_03560W_A	0.37	1.08	0.39	68.51	85.47	100.20	1.59E-60	6.97
C4_04020C_A	0.00	0.19	0.55	36.17	35.49	29.65	7.99E-31	6.94
C3_06580W_A	0.05	0.17	0.00	7.11	3.49	10.26	5.68E-17	6.45
C3_00220W_A	1.12	0.22	1.08	70.93	17.26	106.84	2.79E-18	6.20
C5_04930C_A	1.62	1.59	1.66	141.22	57.68	108.60	1.14E-60	5.86
C1_13080W_A	0.00	0.20	0.00	4.73	3.76	2.76	1.15E-08	5.73
C6_02890C_A	0.08	0.81	1.08	30.46	20.48	29.63	3.25E-14	5.19
C1_02110C_A	8.44	5.28	7.32	238.83	268.71	300.57	8.19E-128	5.15
C1_00190C_A	2.37	0.63	0.85	46.22	33.36	48.85	1.60E-26	4.92
CR_03580C_A	0.16	2.43	4.98	78.44	81.48	72.22	6.57E-05	4.79
C1_11320C_A	91.30	67.43	128.81	2630.90	2537.26	2759.70	4.75E-66	4.67
C4_03840C_A	0.00	0.15	0.00	0.82	1.78	1.17	0.000250292	4.60
C1_06230C_A	4.61	2.68	2.70	78.14	81.25	93.55	4.59E-74	4.55
C3_00920W_A	0.23	0.03	0.00	3.07	1.31	2.52	6.92E-06	4.35
C3_04000C_A	1.79	1.29	0.93	24.33	26.49	29.57	3.70E-59	4.20
CR_02360W_A	7.90	6.69	13.97	199.58	183.43	178.84	6.04E-38	4.19
C1_08330C_A	48.41	59.96	65.81	1028.21	873.13	962.45	6.49E-78	3.92
C1_02070W_A	0.26	0.16	0.32	4.20	3.71	4.83	1.06E-18	3.91
C3_00230C_A	1.83	0.75	1.13	7.81	31.78	20.78	2.50E-10	3.88
CR_03450W_A	6.81	3.79	5.07	81.27	83.26	76.59	2.80E-58	3.84
C2_02550C_A	0.68	1.01	0.52	11.81	9.37	13.39	3.06E-33	3.84
C7_01510W_A	2.46	2.84	0.01	26.79	25.30	26.89	0.015466265	3.78
CR_05170C_A	13.52	13.51	17.49	235.01	227.74	160.18	5.26E-45	3.70
C2_05990C_A	1.87	1.47	1.96	25.51	22.68	24.33	1.09E-67	3.65
C4_05700W_A	0.81	0.47	0.40	7.35	6.07	8.70	2.29E-29	3.58
C1_10550C_A	2.49	2.16	4.56	36.59	30.56	39.35	7.08E-24	3.41
C1_01740W_A	0.51	0.43	0.43	7.14	3.99	4.68	2.42E-22	3.40
C5_02600W_A	1.84	1.48	1.77	23.48	15.94	18.43	1.43E-40	3.39
C4_05870C_A	0.37	0.07	0.21	1.38	4.07	1.66	5.88E-06	3.29
C1_10290W_A	1.92	2.01	0.71	21.54	11.25	16.21	6.10E-13	3.28
CR_06650C_A	1.51	0.94	0.99	7.37	10.53	18.06	4.58E-10	3.23
C2_05950C_A	2.76	4.87	5.75	48.99	40.39	43.81	1.02E-20	3.19
C3_01930W_A	0.63	1.47	1.29	12.82	5.66	11.91	3.30E-10	3.03
C1_02130C_A	8.48	10.84	8.98	87.89	77.76	84.08	1.96E-53	3.02
CR_03500W_A	29.73	20.14	23.85	209.91	207.14	222.77	6.42E-66	3.01
CR_00330C_A	2.12	2.85	6.95	20.73	36.16	44.32	2.80E-08	2.96
CR_09740W_A	0.39	0.23	0.20	3.53	1.91	1.47	3.36E-07	2.91
CR_08210C_A	57.95	16.10	49.31	251.34	277.82	459.90	2.92E-09	2.90
C4_02640C_A	0.29	0.00	0.05	1.34	0.81	0.74	0.011301955	2.89
C1_06860W_A	0.13	0.15	0.12	1.10	0.91	1.46	4.63E-07	2.87
C5_01360W_A	1.08	0.42	0.78	5.84	5.05	7.17	7.20E-15	2.85
C4_02230C_A	0.47	1.15	0.26	4.87	4.54	5.28	9.27E-07	2.81
C1_08370W_A	81.16	47.73	62.33	439.91	482.30	445.19	6.02E-34	2.74
C2_02970C_A	221.82	182.18	224.15	1461.75	1528.80	1514.09	9.79E-80	2.73
C7_02010C_A	0.22	0.64	0.34	3.08	2.76	2.64	2.95E-08	2.69
CR_07170W_A	11.03	13.00	11.46	84.70	79.35	83.44	1.08E-53	2.69
C1_02180W_A	5.45	5.03	4.61	30.59	34.85	36.63	7.46E-51	2.64
C2_07340W_A	0.16	0.98	0.24	4.31	2.43	2.87	0.000541114	2.64
C1_01620C_A	1.08	1.06	1.34	8.44	7.06	8.09	3.91E-21	2.64
C1_02150W_A	13.98	15.75	10.73	92.99	84.82	85.68	2.45E-33	2.59
C1_06810W_A	24.48	21.42	31.12	114.66	167.33	208.43	4.16E-18	2.55
C1_04500W_A	31.49	60.65	37.89	263.24	250.63	314.44	5.09E-15	2.54

C6_01450C_A	5.62	5.22	2.51	23.68	26.74	31.95	1.24E-12	2.51
C2_09880C_A	1.15	0.80	0.82	5.94	5.57	5.75	4.29E-18	2.50
C2_05840W_A	0.51	0.54	0.45	3.25	2.44	3.65	2.78E-11	2.50
C5_02080C_A	41.16	233.28	169.40	485.92	1479.87	764.94	0.000416502	2.49
C3_07800C_A	2.85	2.09	2.48	18.26	14.33	12.68	2.37E-16	2.48
C4_05230C_A	0.33	0.09	0.03	0.64	1.02	1.03	0.00466765	2.45
C3_01320C_A	1.85	1.81	1.37	7.99	8.69	12.95	9.99E-17	2.43
CR_04820W_A	16.35	14.11	14.22	79.49	77.33	103.50	6.54E-32	2.43
C2_05160C_A	0.82	0.88	1.41	6.31	6.13	5.30	1.74E-13	2.40
C2_06070W_A	5.04	4.43	2.90	20.78	24.45	25.14	5.06E-20	2.40
C4_06210C_A	2.54	2.42	1.76	18.21	8.88	11.16	8.04E-11	2.40
C1_06350W_A	2.56	0.37	3.40	12.79	14.70	7.93	0.000985995	2.39
C7_02240W_A	5.55	6.24	5.88	22.11	45.75	31.15	7.97E-12	2.38
C6_01500C_A	2.28	1.18	2.90	16.51	3.97	15.24	7.43E-05	2.37
C1_05160C_A	117.80	118.43	87.95	570.25	581.42	620.13	1.11E-36	2.34
C3_04910C_A	144.47	214.38	135.93	923.11	912.43	880.34	5.85E-19	2.34
C3_04900W_A	0.15	0.39	0.28	1.34	1.20	2.01	5.91E-06	2.33
C1_04460C_A	11.92	13.71	14.41	74.09	65.68	76.36	3.94E-35	2.32
C2_06680W_A	5.34	10.77	15.24	63.16	49.57	57.17	9.45E-08	2.31
C2_07900W_A	52.80	57.82	59.77	305.68	307.21	285.61	1.36E-44	2.29
C1_07490C_A	1.05	1.06	0.69	4.65	5.66	4.55	1.74E-14	2.28
C6_02840C_A	2.72	2.07	2.43	12.91	11.85	13.20	1.12E-32	2.28
C2_05180W_A	162.25	95.33	145.79	904.67	586.31	604.50	5.10E-14	2.27
C5_04690C_A	0.47	3.65	1.42	8.08	12.48	9.39	0.005138727	2.26
C1_05060W_A	1.14	0.37	0.99	4.59	3.05	5.28	9.21E-06	2.24
C1_01160C_A	1.90	1.86	1.43	12.59	6.32	7.76	2.35E-08	2.23
C1_10970W_A	1.75	1.06	0.87	7.01	5.91	5.61	2.38E-11	2.22
C2_05750W_A	1.06	1.18	0.76	5.37	4.58	5.25	3.26E-13	2.21
C1_04040C_A	0.43	1.08	0.34	2.66	2.82	3.90	4.86E-05	2.20
CR_04110W_A	0.75	1.13	0.98	4.71	5.06	4.40	1.41E-12	2.18
CR_00540C_A	39.91	30.18	36.90	51.22	218.69	243.49	0.00013541	2.15
C4_06490C_A	0.22	0.19	0.18	0.44	1.39	1.12	0.001623245	2.14
CR_02920C_A	25.90	45.91	63.64	124.59	243.27	285.87	5.85E-06	2.14
C6_01490C_A	9.11	5.91	10.91	60.06	11.06	53.23	0.000922556	2.14
CR_04560C_A	3.36	3.32	3.51	15.46	17.50	14.92	2.23E-23	2.11
C4_05130C_A	2.95	2.57	2.66	13.15	11.02	14.30	6.73E-27	2.11
CR_07250C_A	4.11	2.22	3.04	11.12	15.52	17.00	4.70E-12	2.11
C5_04030W_A	1.00	0.70	0.49	2.40	3.26	4.35	5.57E-06	2.05
CR_10550W_A	6.14	3.61	3.82	19.50	20.73	19.80	1.05E-15	2.04
C4_05010W_A	0.40	0.54	1.20	3.73	2.78	2.99	0.000143097	2.03
C6_00150W_A	312.86	289.48	265.58	1197.06	1318.98	1306.62	2.68E-50	2.03
CR_00680W_A	0.51	1.63	1.36	0.95	7.38	7.48	0.020522349	2.03
C6_00930C_A	29.39	2.85	31.16	33.75	88.87	154.08	0.023454696	2.02
C1_05500W_A	0.33	0.08	0.43	0.73	1.34	1.71	0.003377878	2.02
C3_07640C_A	18.41	20.91	16.23	95.25	43.33	106.30	9.15E-08	2.01
C2_09380W_A	2.64	3.36	1.73	11.58	11.24	11.16	1.20E-09	2.01
CR_09920W_A	6.00	4.66	5.76	23.03	22.69	25.75	2.16E-32	2.01
C1_08290C_A	0.63	0.60	0.62	2.95	1.99	3.11	3.45E-11	2.00
C4_04720W_A	67.50	56.73	35.18	205.19	285.33	191.15	1.38E-09	2.00
C6_03200W_A	21.53	11.99	18.76	44.03	66.55	114.63	1.78E-06	1.99
C3_06030W_A	1.22	1.56	1.92	11.69	4.13	4.44	0.000202499	1.99
C1_01330C_A	19.82	16.65	16.64	77.28	75.04	74.92	1.09E-35	1.98
C2_08580W_A	0.48	0.19	0.15	1.28	1.49	0.95	0.002512063	1.98
CR_04440C_A	13.31	13.50	18.42	54.01	77.55	61.43	5.65E-12	1.98
C1_01240W_A	7.79	5.64	16.54	39.18	40.92	48.21	9.45E-06	1.98
C3_06060W_A	1.95	2.07	2.04	10.65	7.33	7.88	1.85E-13	1.97
C1_12850W_A	550.78	329.20	680.38	1808.23	2060.99	2744.27	2.67E-09	1.97
C4_06620C_A	0.96	0.66	1.18	3.46	3.83	4.91	0.000142727	1.97
CR_05940W_A	0.03	0.64	0.72	1.91	1.70	2.56	0.046082455	1.96
C3_01130C_A	7.91	5.53	6.04	22.11	26.80	32.43	1.98E-17	1.95
C1_13670W_A	12.92	8.37	10.85	42.71	44.06	46.36	7.46E-23	1.94
C1_12600C_A	0.98	0.17	0.62	1.76	2.43	3.19	0.003308128	1.94
C5_00590W_A	197.20	208.26	264.46	901.65	852.32	1034.56	3.91E-21	1.94

C3_04940W_A	6.48	7.83	8.53	32.24	28.42	34.27	9.25E-19	1.94
CR_03260W_A	21.37	20.29	21.91	87.23	77.25	99.32	7.57E-34	1.94
C2_07960C_A	1.79	1.35	2.97	8.65	8.57	8.13	1.07E-07	1.94
C5_01060C_A	0.83	0.20	0.75	2.57	3.10	1.62	0.004258742	1.93
C1_13820C_A	0.66	0.41	0.27	1.87	1.62	2.00	0.000203078	1.91
C2_03080W_A	55.42	46.52	56.95	215.45	215.98	206.53	1.91E-35	1.90
C3_04930C_A	4.31	3.48	3.53	14.27	14.79	16.37	9.26E-30	1.89
CR_09800C_A	0.44	0.86	2.10	5.33	2.62	5.87	0.005203897	1.88
C7_00530C_A	2.29	2.35	1.92	8.61	7.36	10.60	8.00E-09	1.88
C4_02620C_A	27.97	26.92	24.64	117.64	96.86	102.19	1.77E-31	1.88
C5_04440C_A	5.68	16.19	12.54	51.75	39.12	48.30	3.01E-05	1.88
C1_00560W_A	0.47	0.26	3.09	3.94	5.83	5.49	0.050510925	1.88
CR_03250C_A	78.32	70.41	59.08	260.38	291.23	270.94	3.63E-27	1.88
C1_12570C_A	9.29	4.39	5.16	9.69	30.70	33.54	0.00065681	1.87
C6_01170W_A	0.66	0.75	0.14	3.32	0.51	2.62	0.050590065	1.87
C7_00870W_A	0.25	0.19	0.30	0.77	1.38	0.85	0.000862337	1.86
CR_07500W_A	1.55	0.74	1.00	3.45	5.28	4.39	4.88E-05	1.86
C5_02110W_A	302.18	70.97	87.51	656.37	282.80	835.41	0.004546322	1.86
C4_02830C_A	0.44	1.09	0.83	0.95	4.56	3.79	0.009668531	1.85
CR_03940W_A	1.81	1.49	1.53	5.83	5.26	7.82	2.65E-10	1.85
C2_02820C_A	4.45	2.89	4.42	15.17	16.25	14.08	8.87E-13	1.84
C2_06600W_A	12.98	5.81	8.03	30.38	33.72	38.95	1.06E-08	1.84
C5_04650C_A	0.64	1.11	0.35	4.04	1.84	2.42	0.002222342	1.84
CR_00300W_A	1.85	0.99	1.37	1.88	1.24	13.45	0.061256894	1.83
C3_00810C_A	9.45	8.28	13.99	40.15	14.64	67.76	0.00125426	1.82
C3_06380W_A	3.22	2.84	3.15	8.31	12.53	14.45	8.20E-09	1.82
C6_03670C_A	12.73	8.35	14.35	34.27	42.74	57.88	8.05E-08	1.81
C1_02530C_A	14.57	12.15	6.20	49.71	29.42	45.01	4.26E-06	1.81
C2_08480W_A	1.22	0.73	0.25	2.11	2.71	3.56	0.004230501	1.81
C3_01560W_A	0.89	0.67	1.70	4.49	3.89	3.98	2.55E-05	1.80
C2_05830C_A	0.90	0.71	1.16	3.10	3.53	3.84	2.57E-08	1.80
C2_07890W_A	8.72	7.90	7.72	22.93	29.09	39.80	4.80E-12	1.80
C1_01220C_A	35.83	19.08	41.03	107.62	128.55	121.01	5.39E-08	1.79
C4_02740W_A	16.88	8.70	9.27	42.75	41.57	44.37	1.58E-09	1.78
CR_03370C_A	2.21	1.42	2.78	7.09	7.59	9.28	2.49E-07	1.78
CR_09730C_A	1.17	0.97	0.53	3.59	2.97	3.52	6.47E-06	1.78
C1_09510W_A	2.70	1.65	1.75	9.06	6.32	7.25	1.09E-08	1.78
C1_03320C_A	5.83	4.68	6.68	23.46	19.23	21.01	7.86E-16	1.78
CR_05730C_A	20.36	14.57	18.28	64.03	65.76	66.75	4.49E-25	1.78
CR_05840W_A	8.92	5.79	2.10	14.71	24.44	22.40	0.001416557	1.77
C6_03370W_A	120.32	82.67	70.54	317.28	332.68	347.74	6.42E-14	1.76
C7_04180W_A	5.69	3.36	4.18	13.97	15.27	19.20	1.21E-11	1.76
CR_07140C_A	10.31	9.35	8.31	30.62	34.27	37.30	1.09E-20	1.76
CR_03360W_A	5.13	2.80	3.17	14.88	12.74	13.08	1.15E-08	1.75
C4_00130W_A	49.58	48.32	43.56	133.64	260.95	116.61	6.20E-06	1.75
C4_00830W_A	13.46	15.88	8.46	60.27	39.50	37.84	1.22E-06	1.75
C7_03910W_A	0.36	1.11	0.35	1.70	1.02	4.09	0.037332566	1.75
C6_03640W_A	1.38	1.07	1.17	4.48	3.76	5.01	5.35E-11	1.75
C3_03650W_A	3.21	2.60	2.32	10.96	8.41	10.08	3.06E-13	1.74
CR_03550W_A	2.12	1.87	2.44	7.11	8.12	8.00	2.13E-13	1.74
C2_02060C_A	1.55	1.51	2.03	7.84	3.11	7.55	0.000118403	1.74
CR_09140C_A	6.52	6.99	7.17	20.94	26.38	26.60	1.25E-18	1.72
C6_00810C_A	1.89	1.27	0.95	5.36	5.13	4.13	4.36E-06	1.71
C4_01500W_A	1.34	0.44	0.41	2.41	2.62	2.78	0.003420323	1.70
C7_02200W_A	8.98	8.37	8.19	19.09	34.12	36.92	2.96E-08	1.70
CR_10690W_A	0.61	0.56	0.95	3.56	1.77	2.22	0.002749843	1.70
C6_03850C_A	2.54	3.27	4.18	14.83	9.05	11.27	1.40E-06	1.70
C1_11200W_A	29.43	23.56	18.80	77.86	84.23	87.86	5.90E-16	1.69
CR_10780C_A	13.16	36.43	20.70	75.51	97.82	75.20	0.000200434	1.69
C4_01000C_A	3.16	3.43	1.76	8.26	9.98	10.94	3.44E-06	1.68
C7_01070C_A	20.51	13.77	12.40	50.37	56.35	53.87	1.18E-12	1.68
CR_07950W_A	4.95	5.48	6.07	23.10	15.36	18.37	1.06E-09	1.67
C6_02360W_A	0.93	0.87	0.55	2.22	3.30	2.91	0.001600269	1.67

C5_03250W_A	0.69	0.28	0.21	1.66	1.25	1.24	0.00505122	1.67
C6_04160C_A	1.13	0.76	0.55	2.80	2.54	3.10	1.54E-06	1.66
CR_05630W_A	1.95	3.64	3.08	9.91	10.59	9.21	1.84E-06	1.66
C1_08350C_A	2425.79	1654.08	2243.68	6053.94	7365.65	7958.50	1.58E-14	1.65
C4_00080C_A	10.33	12.30	9.67	40.59	34.81	34.35	1.17E-14	1.65
C5_02890W_A	46.19	53.63	52.73	180.10	164.22	172.62	1.33E-20	1.64
CR_08820C_A	1044.97	512.12	983.07	1530.77	3952.78	2999.64	0.000331632	1.64
C1_10950C_A	0.95	1.08	0.46	2.98	2.34	3.10	0.000177447	1.64
C2_01120W_A	102.02	130.70	114.75	397.97	383.27	383.64	5.21E-17	1.63
C1_07380C_A	2.38	2.58	5.71	10.48	14.30	10.92	0.000261032	1.63
C7_03280C_A	15.09	12.19	14.31	51.36	35.76	51.80	9.23E-14	1.63
C1_10740C_A	25.91	15.26	17.86	53.75	67.96	73.75	6.75E-10	1.62
C1_11420W_A	1.47	0.78	1.56	4.18	4.29	4.31	1.07E-05	1.62
C5_02550C_A	0.36	0.53	0.55	2.00	1.21	1.60	0.000120715	1.62
CR_07960C_A	4.42	3.45	3.89	12.03	13.33	13.42	2.42E-22	1.61
C7_00290C_A	0.15	0.48	0.60	0.45	1.60	2.18	0.068363534	1.61
C2_02390W_A	48.10	43.09	41.37	133.19	153.03	148.84	7.33E-26	1.60
C3_00880W_A	26.08	24.87	37.90	67.50	91.14	135.49	3.66E-06	1.60
C4_01170C_A	0.47	0.72	0.81	2.74	2.18	1.70	0.000141098	1.60
C4_06580W_A	34.63	27.71	34.20	117.84	81.16	117.68	1.33E-12	1.60
C1_09370W_A	10.89	7.73	12.35	38.45	32.02	30.60	4.30E-10	1.60
CR_06640C_A	8.03	8.62	12.96	36.43	22.35	38.83	3.14E-06	1.60
C2_10100W_A	0.37	0.38	0.10	0.97	0.74	1.11	0.011272946	1.60
C1_03230C_A	4.17	3.25	3.66	14.08	10.71	11.38	7.48E-13	1.59
C1_06800W_A	0.66	0.61	0.51	1.04	2.35	2.40	0.003345276	1.59
C2_10740C_A	2.63	2.74	2.89	8.54	9.42	8.88	8.26E-18	1.59
C2_09500W_A	1.81	1.44	1.99	6.34	5.37	5.36	3.28E-09	1.58
C1_12680W_A	2.01	3.18	3.24	11.53	7.45	8.48	9.72E-06	1.58
C3_02820C_A	4.25	3.99	4.53	9.72	15.90	15.73	1.45E-07	1.58
C6_03770C_A	15.23	18.33	16.47	74.84	40.18	47.01	1.30E-06	1.58
CR_01410C_A	5.61	1.99	2.59	8.74	4.96	19.14	0.013758897	1.58
C7_01760C_A	49.82	41.32	38.31	132.87	145.26	137.64	3.52E-21	1.57
C5_04840C_A	0.81	0.87	0.94	2.82	2.43	3.18	0.000215113	1.57
C1_01150C_A	2.40	3.74	3.00	13.81	7.70	7.93	4.81E-05	1.57
C5_03330C_A	7.30	4.25	5.90	17.36	19.78	18.26	1.38E-10	1.56
C1_10310W_A	3.30	2.06	2.23	7.41	8.19	8.71	2.53E-08	1.56
C1_06760C_A	1.46	1.62	0.67	4.83	3.31	3.86	0.000483786	1.55
CR_07750C_A	3.48	4.24	3.43	13.65	9.99	11.69	3.50E-10	1.55
C2_08690C_A	2.59	0.40	0.32	2.26	2.99	5.01	0.094532091	1.54
C6_03810W_A	9.24	4.98	4.75	18.45	17.51	23.58	2.48E-06	1.54
C6_02730W_A	76.39	35.81	77.18	208.68	208.52	173.15	1.07E-05	1.54
CR_08280W_A	2.17	2.64	2.40	7.10	4.40	11.41	0.000463284	1.54
CR_02890C_A	1.00	0.44	0.60	1.90	2.63	1.86	0.000816741	1.53
C1_12880C_A	7.06	7.69	9.32	27.24	21.88	26.34	7.42E-12	1.53
CR_10650W_A	2.94	3.00	2.33	8.73	9.10	8.03	4.77E-10	1.53
CR_06820W_A	12.57	15.56	17.23	45.52	48.23	48.10	4.65E-11	1.53
C1_10480W_A	10.66	10.34	10.05	41.76	25.48	29.50	5.84E-09	1.53
C4_02540W_A	2.33	2.75	2.26	8.71	8.02	6.15	9.17E-09	1.53
C3_06450W_A	4.00	5.50	6.96	18.01	13.65	20.05	4.38E-06	1.53
C7_01030C_A	0.87	0.82	1.09	3.18	2.50	3.01	2.44E-09	1.53
C2_00410C_A	1.06	0.77	0.74	3.88	1.07	3.08	0.004854357	1.52
CR_08880C_A	7.44	9.64	9.36	20.22	31.93	30.28	3.09E-06	1.52
CR_10240W_A	2.00	1.58	1.46	5.67	4.86	5.14	1.96E-10	1.52
C1_06190C_A	0.00	0.00	2.52	6.32	1.30	0.24	0.704004026	1.51
C5_01600C_A	0.69	0.04	0.22	0.65	1.13	1.11	0.147786546	1.51
C3_03490W_A	40.46	42.97	26.75	140.58	70.30	129.26	3.20E-05	1.51
C4_03940C_A	24.51	21.27	24.39	70.85	72.28	72.12	4.86E-31	1.51
C2_07640W_A	25.92	22.09	21.96	61.70	74.65	78.03	4.98E-19	1.50
C4_01450W_A	2.33	3.91	2.76	13.12	7.07	7.51	0.000281761	1.50
CR_05660W_A	3.04	2.37	1.65	7.51	6.76	7.27	1.21E-07	1.50
C6_03880W_A	7.50	5.54	6.52	16.95	20.05	22.69	5.04E-11	1.50
C1_14230C_A	2.90	2.55	2.47	8.72	7.48	7.93	1.37E-17	1.49
C3_04070C_A	16.82	14.60	16.92	48.23	48.07	50.50	9.13E-31	1.49

C6_01890C_A	1.70	1.07	1.79	5.25	4.47	4.11	2.47E-06	1.49
C1_13170C_A	3.29	4.46	3.50	12.05	11.21	10.95	1.50E-10	1.49
CR_06120W_A	3.92	2.03	2.98	7.94	8.14	11.02	1.16E-05	1.49
CR_00320C_A	23.12	32.81	60.09	118.30	66.81	170.77	0.004172417	1.48
C2_00030W_A	0.73	1.72	2.18	5.34	2.36	6.54	0.017912169	1.48
C2_06990W_A	0.85	0.74	0.72	2.31	2.79	1.88	7.21E-05	1.48
C1_07980C_A	19.41	19.76	14.21	56.40	44.78	59.88	2.85E-10	1.48
C4_03340C_A	0.68	0.07	0.19	0.89	0.71	1.32	0.121289964	1.47
C6_02250W_A	0.64	0.14	0.20	0.77	1.11	1.16	0.063846321	1.47
C3_05160C_A	1.76	1.23	0.94	4.44	3.86	3.42	6.40E-06	1.47
C2_03350W_A	0.21	0.08	0.37	0.75	0.77	0.46	0.068602444	1.47
CR_07420W_A	67.62	54.80	59.97	169.44	192.74	180.92	2.08E-22	1.47
C3_04300C_A	5.67	5.02	5.08	16.88	15.14	15.02	3.08E-22	1.47
C4_03160C_A	7.79	4.98	1.69	16.40	11.98	14.45	0.010028403	1.46
C5_00070W_A	27.33	23.08	11.51	61.40	44.41	78.90	0.00029132	1.46
CR_00200W_A	128.79	136.85	207.84	141.31	594.48	686.19	0.017435252	1.46
C7_00160C_A	23.67	0.22	15.76	34.55	41.50	38.48	0.406542659	1.45
CR_02350C_A	9.13	7.43	8.75	20.12	28.43	25.99	3.53E-11	1.45
C1_07220W_A	8.52	7.87	7.44	15.15	28.06	27.07	3.52E-06	1.45
C2_06690C_A	9.83	14.39	9.24	28.06	29.57	41.89	6.81E-06	1.45
C7_03540C_A	0.73	0.23	0.43	0.63	1.80	1.63	0.032944188	1.44
C2_02510W_A	4.70	5.31	13.66	18.57	23.85	27.65	0.00547152	1.44
CR_01580C_A	6.08	4.41	4.58	13.35	16.32	14.37	4.68E-11	1.44
C3_02020W_A	4.75	3.39	3.64	12.24	10.41	11.80	6.94E-13	1.44
C1_05360C_A	2.85	3.11	3.30	7.92	9.73	9.45	9.05E-11	1.44
C1_05930C_A	1.97	1.44	1.56	4.92	4.78	4.87	7.41E-13	1.44
CR_06930W_A	7.23	3.28	2.00	9.50	8.75	18.25	0.013833729	1.43
C1_07550W_A	11.56	6.37	5.10	18.71	24.27	23.43	9.69E-05	1.43
CR_08250C_A	71.61	111.05	94.41	249.87	218.99	346.39	2.15E-06	1.43
CR_03120W_A	12.24	16.79	17.63	46.57	38.15	51.72	8.73E-08	1.43
C1_06630W_A	2.99	2.73	1.50	7.11	5.43	8.48	6.37E-05	1.42
C5_00670C_A	12.17	7.87	8.50	25.32	26.87	30.31	1.11E-10	1.42
C5_03700C_A	23.07	8.61	18.01	67.28	29.61	45.27	0.003397243	1.42
C7_02030W_A	113.39	214.04	152.60	511.28	435.93	450.76	1.33E-05	1.42
C2_04570W_A	2.70	1.28	1.12	5.33	5.10	4.21	0.001418626	1.42
C3_07830W_A	66.78	48.75	68.60	200.33	169.79	158.76	2.34E-10	1.42
C3_01520C_A	4.61	3.14	2.52	10.26	8.86	10.55	2.45E-06	1.42
C1_12310C_A	0.84	0.19	0.04	0.00	2.05	1.09	0.45741864	1.41
C5_01900C_A	7.70	5.67	7.99	19.45	23.38	18.85	8.37E-05	1.40
C5_02070C_A	12.00	13.87	12.05	41.35	32.68	34.52	4.86E-11	1.40
C2_10610W_A	19.12	18.06	18.75	52.13	52.78	54.76	1.96E-29	1.40
C6_02150C_A	0.91	0.53	0.31	1.44	1.28	2.34	0.01781091	1.39
C3_07980C_A	4.55	2.75	3.01	10.68	10.56	8.10	0.000266155	1.39
C1_06090C_A	4.99	3.89	4.17	11.67	11.77	13.56	3.72E-16	1.39
CR_00150C_A	21.62	22.51	4.92	51.90	28.34	59.14	0.029290372	1.39
C4_06430C_A	1.20	0.99	1.08	1.98	2.27	5.17	0.005556012	1.39
CR_01470W_A	89.58	91.22	69.68	257.83	220.00	227.67	1.99E-12	1.38
C7_03020C_A	30.78	33.23	15.80	70.25	63.73	91.54	0.000121385	1.38
C4_01930C_A	6.81	2.60	3.16	11.37	8.66	15.19	0.00386339	1.38
CR_10410C_A	6.72	5.14	8.21	17.77	16.34	22.31	6.55E-07	1.38
C1_07150W_A	6.35	3.66	5.68	21.43	13.24	9.42	0.008117966	1.38
C2_01760C_A	3.75	2.50	3.40	7.18	8.44	11.43	3.66E-05	1.37
C1_00990C_A	3.04	3.51	3.61	10.60	9.11	8.72	4.76E-10	1.37
C1_03980W_A	2.21	1.58	1.36	5.94	3.80	4.84	0.00174574	1.37
C1_07400C_A	5.04	3.57	5.06	15.48	14.30	8.08	0.0002536	1.37
C1_05340C_A	6.15	6.93	5.23	16.29	17.82	17.04	1.96E-11	1.37
C3_02720W_A	1.86	1.53	1.68	4.97	4.49	4.70	3.66E-08	1.37
C1_09490C_A	18.45	10.80	16.26	54.61	27.92	43.89	9.80E-05	1.37
C4_06390W_A	4.92	7.65	7.13	17.80	16.52	20.96	4.74E-06	1.37
C6_02970C_A	2.75	2.82	2.76	6.42	7.13	9.71	1.35E-07	1.36
CR_01780W_A	1.51	2.12	1.50	4.84	5.25	4.24	0.000127813	1.36
C2_06000W_A	0.92	1.49	1.93	4.41	3.29	4.31	0.001267323	1.35
C1_05050C_A	2.17	2.22	2.12	6.29	5.92	5.68	1.16E-11	1.35

CR_02030C_A	2.37	2.12	1.76	5.42	5.25	6.50	5.00E-08	1.34
C2_07730W_A	10.75	8.98	9.54	34.43	23.01	22.53	2.98E-07	1.34
C2_04150C_A	72.12	33.67	60.84	108.94	142.84	202.58	0.000504511	1.34
C1_07950C_A	0.67	1.03	0.33	1.46	1.26	2.90	0.060800905	1.34
C6_02780C_A	23.70	20.12	19.93	55.14	60.80	57.67	3.26E-19	1.34
CR_02800C_A	1.57	3.61	3.67	9.82	6.56	7.96	0.003290231	1.33
C2_08700C_A	49.70	20.16	21.50	60.38	76.69	109.18	0.00308026	1.33
C5_04740C_A	5.59	3.99	4.42	14.62	11.19	12.26	1.35E-08	1.33
C1_03770W_A	7.43	3.66	4.07	13.86	12.79	14.19	4.34E-05	1.33
C2_04010C_A	165.90	197.14	148.41	396.65	477.11	519.00	1.68E-09	1.33
CR_08500W_A	6.13	4.72	4.76	14.75	12.77	14.84	3.08E-11	1.33
C2_06630C_A	6.75	22.66	10.63	27.98	40.07	42.24	0.023342327	1.33
C1_04360C_A	213.71	189.93	346.44	678.96	299.00	1080.48	0.012688424	1.32
C3_04630W_A	4.89	3.79	4.17	8.51	17.58	8.33	0.002149226	1.32
CR_09590W_A	8.32	5.38	6.19	15.40	16.87	21.46	5.68E-07	1.32
C4_02790C_A	0.76	1.06	1.05	3.42	1.77	2.64	0.002342287	1.32
C1_10520W_A	0.67	0.18	0.62	1.82	0.64	1.52	0.073807877	1.32
C6_04180W_A	3.02	1.47	1.70	4.81	4.81	6.92	0.00026878	1.31
C2_01780W_A	5.03	9.19	9.81	21.07	22.27	21.72	0.000160314	1.31
C2_01200C_A	35.17	41.00	37.77	91.83	95.69	119.02	1.06E-11	1.31
C1_04610W_A	314.37	285.64	302.57	790.69	813.61	805.24	6.25E-30	1.31
C1_13880C_A	8.71	9.84	6.21	22.85	21.52	21.74	1.42E-07	1.30
CR_01990C_A	1.56	1.18	1.24	2.87	2.92	4.87	0.000100861	1.30
C5_04660C_A	4.42	9.29	8.94	26.57	16.03	18.15	0.003850536	1.30
C2_05770W_A	13.78	14.21	12.33	34.23	35.46	37.28	2.05E-16	1.29
CR_10540C_A	1.58	1.50	2.93	5.08	5.62	5.27	0.000709927	1.29
C3_01460C_A	11.16	11.65	14.42	29.70	31.71	37.27	2.96E-09	1.29
CR_01980C_A	14.06	16.58	20.14	54.99	37.76	41.83	6.63E-06	1.29
C2_07920W_A	3.05	4.35	5.77	9.63	12.17	13.12	0.000381465	1.29
C4_01300W_A	62.11	34.64	33.21	92.28	111.97	135.29	5.72E-05	1.28
C3_06370C_A	11.64	5.72	7.18	20.91	20.63	22.68	3.10E-05	1.28
C2_06360C_A	0.28	0.60	0.58	1.37	1.03	1.57	0.045999487	1.28
C6_04530C_A	5.12	4.78	4.80	12.88	13.02	12.80	1.10E-18	1.28
C3_06520C_A	2.78	3.21	2.84	6.26	11.59	5.22	0.005161545	1.28
CR_10140W_A	18.01	19.00	25.34	76.16	41.18	46.25	0.00025302	1.28
C3_07300W_A	2.69	1.68	1.80	6.26	4.02	5.92	0.000256643	1.27
C1_09780C_A	8.84	11.33	7.75	25.69	22.89	24.64	9.45E-08	1.27
C2_05510C_A	2.81	2.20	2.38	6.79	6.55	5.96	1.11E-07	1.27
C3_06760W_A	0.81	0.71	0.81	2.38	1.50	2.25	0.007806262	1.27
C2_03390C_A	22.97	26.65	28.43	95.55	62.53	44.69	0.000557821	1.27
C4_02500C_A	1.18	0.84	0.82	3.02	1.97	2.45	2.55E-05	1.27
C1_02170C_A	2.35	2.96	1.56	4.52	6.73	6.69	0.001589431	1.26
C2_03130W_A	6.75	9.81	9.58	24.34	22.96	20.67	1.45E-05	1.26
C2_02170W_A	4.00	2.53	1.92	6.95	6.01	8.98	0.000620536	1.26
C2_02710C_A	10.77	3.68	7.41	29.68	8.58	17.93	0.041802038	1.26
C2_00150W_A	9.94	7.86	10.16	22.08	23.44	26.92	7.20E-11	1.26
C4_02000C_A	27.81	10.81	31.06	69.61	53.63	55.70	0.004211337	1.26
C5_01950C_A	10.96	6.88	9.53	28.32	19.58	22.71	3.40E-06	1.26
CR_05340C_A	231.63	256.78	157.57	844.66	540.57	274.60	0.010831017	1.26
C1_04390C_A	6.15	4.96	3.91	14.12	11.24	13.48	1.88E-07	1.26
C7_00070C_A	73.60	0.36	59.15	109.30	110.32	116.98	0.527654695	1.26
C1_13350W_A	45.39	32.72	32.55	126.76	81.80	74.79	4.33E-05	1.26
C5_02000C_A	45.12	48.72	61.70	129.02	111.60	162.95	4.82E-07	1.25
C5_04990W_A	1.02	1.42	1.32	2.53	4.03	3.19	0.016636455	1.25
CR_08860W_A	26.88	26.32	27.75	71.01	68.84	68.31	9.51E-20	1.25
CR_03680C_A	1.90	1.45	1.59	5.71	3.29	3.67	0.000102723	1.25
C1_03680W_A	17.29	14.48	11.93	35.72	40.25	35.64	1.47E-09	1.25
C2_04120C_A	3.70	1.88	2.72	5.30	9.82	5.88	0.003930827	1.24
C1_01890C_A	36.11	40.22	32.85	93.12	89.17	97.18	1.62E-13	1.24
C2_01060C_A	0.74	1.78	0.97	2.95	3.80	2.23	0.069587804	1.24
C1_10010C_A	16.71	12.28	12.76	33.58	38.29	33.75	1.14E-10	1.24
C5_03710C_A	0.84	1.01	1.92	4.85	1.26	3.62	0.094187006	1.23
C1_00410C_A	5.01	6.71	7.83	17.28	15.83	16.86	5.75E-06	1.23

C5_01460W_A	1.60	1.67	1.54	4.33	4.30	3.59	3.27E-08	1.23
C6_00290W_A	48.97	29.57	34.44	95.38	94.74	94.09	6.87E-08	1.23
C2_01660C_A	7.50	3.78	4.80	9.00	15.36	16.29	0.003245171	1.23
C2_03520C_A	92.98	93.05	79.26	224.03	239.37	207.48	2.00E-12	1.23
C1_11350C_A	18.72	13.00	16.44	44.74	36.99	39.82	1.58E-09	1.23
C3_04560W_A	4.38	2.52	4.62	10.76	11.72	6.39	0.002153058	1.23
CR_03650W_A	2.32	0.73	1.63	4.45	3.32	3.95	0.009061373	1.23
C1_12590W_A	34.24	22.03	35.48	77.74	76.99	76.66	1.05E-06	1.23
C5_05340W_A	24.06	25.69	22.13	55.14	52.31	74.46	1.07E-08	1.22
C1_07420C_A	2.32	2.57	2.05	5.52	5.33	6.58	1.16E-06	1.22
C5_05120W_A	9.60	16.83	20.85	40.19	38.38	40.95	0.001055598	1.21
C5_04470C_A	68.47	111.20	104.17	219.45	237.49	258.97	2.72E-05	1.21
CR_09580C_A	13.63	11.02	18.83	27.50	48.68	32.03	0.001138609	1.21
C2_04550C_A	25.58	34.11	52.13	92.23	90.29	98.60	0.00043683	1.21
C5_03630C_A	1.84	3.13	2.19	2.26	9.98	5.59	0.093588314	1.20
CR_00290W_A	22.16	20.39	24.91	55.67	54.95	57.40	2.79E-13	1.20
C3_04230W_A	2.31	1.43	1.03	3.37	3.88	4.60	0.001477453	1.20
C4_01290W_A	12.89	9.08	7.50	14.64	19.87	38.99	0.007207357	1.20
C5_00790C_A	23.20	41.98	22.47	86.88	44.72	89.18	0.006032421	1.20
C3_00930W_A	20.01	17.01	21.95	59.76	42.25	43.88	1.72E-06	1.20
CR_08160W_A	33.07	29.63	24.71	77.31	38.08	102.05	0.003038505	1.19
CR_01950W_A	11.46	10.19	11.98	29.31	26.85	26.87	1.94E-11	1.19
C2_10170C_A	34.63	33.60	32.38	82.86	76.86	88.25	1.64E-18	1.19
CR_01090W_A	9.60	8.28	2.82	8.01	19.13	23.77	0.058780485	1.19
C3_07070C_A	37.84	31.93	35.18	81.55	84.71	91.68	7.74E-19	1.19
C6_00280W_A	53.94	32.22	47.02	106.34	109.57	109.65	3.57E-07	1.19
C1_01140C_A	36.89	49.27	48.91	130.97	96.40	106.13	4.42E-06	1.18
C5_02050W_A	4.32	2.72	4.39	9.35	9.36	9.24	1.84E-06	1.18
CR_08490W_A	3.31	3.27	3.79	9.89	8.17	7.35	7.71E-07	1.18
C1_08850C_A	2.58	3.11	3.04	7.05	7.22	7.23	6.80E-08	1.18
CR_08670C_A	5.29	2.22	7.95	16.60	20.03	0.48	0.343715925	1.18
C3_00330W_A	2.88	3.75	3.34	8.15	6.15	10.28	8.59E-05	1.18
C2_02430W_A	2.53	0.93	1.61	5.35	3.14	3.77	0.011664145	1.17
C1_06660W_A	35.45	32.36	34.01	89.94	67.80	90.39	9.07E-11	1.17
C7_02090C_A	6.73	6.63	3.47	15.96	13.16	11.74	0.001797303	1.17
CR_06840W_A	2.23	2.17	2.18	5.62	4.68	5.71	1.97E-08	1.17
C2_05250C_A	65.30	19.27	39.16	51.88	99.03	146.23	0.043939442	1.17
C2_09660W_A	1.47	1.75	2.61	4.60	4.43	5.23	0.00512195	1.17
C3_06720W_A	7.33	5.97	7.01	17.19	15.18	16.80	3.56E-13	1.16
CR_04570C_A	26.64	29.99	34.30	79.56	68.50	72.52	2.14E-08	1.16
CR_02420W_A	7.61	5.35	7.20	15.74	13.10	20.02	2.49E-05	1.16
CR_06320C_A	4.76	3.49	3.75	11.12	10.86	6.77	0.00048284	1.16
C1_09070W_A	5.01	2.68	2.02	6.53	7.06	9.73	0.004454365	1.16
C2_07120W_A	20.97	37.05	44.37	64.06	96.88	88.27	0.003834116	1.16
C7_03180C_A	213.01	200.57	234.85	481.58	569.10	511.51	3.66E-11	1.16
C3_01960C_A	16.85	19.32	22.81	55.65	35.89	51.49	2.60E-05	1.16
C1_07360W_A	5.58	4.87	4.68	12.55	13.67	10.07	2.67E-07	1.16
C1_13630W_A	18.77	15.72	15.90	36.03	39.96	45.33	2.51E-12	1.16
C6_02290C_A	0.48	0.26	0.33	1.00	0.83	0.79	0.011791334	1.16
C3_01120W_A	3.55	1.94	1.48	3.46	7.21	6.02	0.025538481	1.16
C1_01490W_A	167.49	168.20	203.42	261.51	543.91	494.35	0.001142821	1.16
CR_09100C_A	10.73	11.80	10.34	21.16	23.48	34.71	2.31E-05	1.15
C7_00370W_A	1.38	0.35	1.74	2.92	2.40	3.04	0.080666617	1.15
C1_05910W_A	18.35	19.27	16.93	47.59	39.32	43.96	3.84E-11	1.15
C2_06860W_A	1.62	1.07	1.27	3.60	2.84	3.00	5.02E-06	1.14
C2_03280W_A	70.29	55.24	52.24	119.80	100.27	205.37	0.000540739	1.14
C2_05230C_A	2.09	0.25	1.85	3.87	2.31	3.80	0.179023741	1.14
C2_05500W_A	168.54	159.63	170.52	392.16	400.49	391.61	4.13E-19	1.14
C1_10360C_A	11.33	16.05	11.90	36.25	24.35	33.11	9.50E-05	1.13
C4_02760C_A	2.51	2.70	3.94	6.55	7.42	7.78	0.000445498	1.13
C2_09320C_A	9.86	5.43	7.96	20.12	14.19	20.52	0.000195124	1.13
C4_01890C_A	20.94	19.56	14.78	41.83	31.11	58.03	0.000422933	1.12
C4_02020W_A	11.88	8.73	20.56	38.26	27.51	31.08	0.005655623	1.12

CR_07350W_A	4.68	5.32	6.20	13.95	12.48	11.68	2.02E-06	1.12
CR_09510C_A	10.60	6.96	9.05	14.95	26.53	20.75	0.000899353	1.12
C6_00250W_A	26.48	27.30	25.63	57.82	63.34	65.40	2.90E-14	1.12
C1_04930C_A	1.31	0.90	1.52	4.13	2.78	1.88	0.050730859	1.12
C4_01900C_A	328.42	292.51	285.85	615.25	710.12	801.57	6.03E-12	1.12
C5_04570C_A	7.00	8.36	7.84	19.69	18.44	16.26	5.16E-07	1.12
C3_06290W_A	12.73	9.37	11.18	17.91	29.94	29.77	0.000147347	1.11
CR_06950C_A	22.17	15.66	18.81	30.52	20.13	83.20	0.071807825	1.11
C2_00070C_A	3.92	7.69	15.39	27.01	8.86	28.25	0.119158949	1.11
C7_01780W_A	42.49	25.06	25.78	71.46	70.67	73.33	1.53E-05	1.11
C1_06180W_A	76.17	56.79	63.85	146.18	170.96	138.36	3.94E-08	1.11
C6_00130C_A	59.42	57.74	52.05	127.20	131.57	135.05	6.78E-17	1.11
C3_00680C_A	1.36	1.11	0.94	2.56	2.84	2.69	0.036493624	1.11
C1_00900W_A	3.48	3.07	4.75	11.29	6.25	8.79	0.005565561	1.10
C2_03110W_A	0.67	0.52	0.24	1.25	1.23	0.88	0.117632078	1.10
C1_02750C_A	18.44	18.56	17.28	43.74	38.61	43.88	1.17E-14	1.10
C7_01200C_A	4.66	3.65	3.05	9.56	7.80	8.95	4.77E-06	1.10
C1_13320C_A	17.45	26.93	33.28	102.24	44.42	33.75	0.050546886	1.10
C2_05340C_A	2.62	2.86	3.02	7.44	5.15	7.15	0.000234223	1.10
CR_02550C_A	2.36	3.50	2.63	5.49	6.70	7.37	0.001375975	1.09
C7_02220C_A	11.20	6.76	7.55	16.79	18.20	23.36	9.93E-05	1.08
C2_00600C_A	21.33	24.74	19.44	56.44	44.47	49.63	1.01E-06	1.08
CR_08890C_A	52.42	51.78	41.93	60.56	130.62	144.33	0.006176227	1.08
C1_07600W_A	7.11	8.17	8.01	9.16	20.57	24.00	0.018729442	1.08
C5_05500C_A	227.30	165.86	175.55	430.34	490.83	369.69	1.74E-06	1.08
C2_06670C_A	1.60	0.91	1.46	3.06	3.68	2.31	0.005487865	1.08
C1_02850W_A	6.84	4.97	5.73	14.05	11.88	14.12	3.62E-08	1.08
C4_04290W_A	7.03	7.73	4.60	16.44	16.71	10.89	0.001988155	1.08
C3_03170W_A	70.89	37.57	65.68	132.38	131.32	131.06	0.000195029	1.08
CR_03710C_A	20.81	21.61	16.79	41.59	53.99	39.06	3.07E-05	1.08
C4_01770W_A	9.97	11.28	12.35	28.19	23.55	25.09	1.59E-06	1.08
C2_09010W_A	41.38	31.71	30.64	70.81	83.18	80.89	1.34E-08	1.07
C2_03790C_A	118.70	153.55	138.19	292.43	297.01	348.35	4.65E-07	1.07
C5_02280C_A	1.49	0.69	0.72	2.07	2.31	2.19	0.021623101	1.07
C2_07450C_A	3.31	3.55	3.61	7.30	7.37	9.14	8.04E-06	1.07
C5_00780C_A	0.00	8.08	1.81	12.10	0.18	11.20	0.750597087	1.07
C1_10910C_A	8.86	7.87	7.56	16.69	17.58	20.86	1.88E-09	1.07
C1_09910C_A	2.73	2.67	3.38	6.91	6.92	6.15	0.000236407	1.07
C4_00490W_A	6.38	2.83	3.23	7.45	9.53	11.10	0.008117966	1.07
CR_02510W_A	32.81	26.56	23.48	63.13	59.15	65.10	1.13E-09	1.07
CR_00230W_A	5.98	6.48	6.33	14.08	14.09	14.45	2.44E-12	1.07
C3_06900W_A	11.33	11.89	10.84	24.17	27.56	25.43	4.50E-08	1.07
C5_05230C_A	110.04	92.77	174.50	378.71	242.46	231.38	0.005619972	1.07
C3_00270C_A	11.57	12.99	14.88	27.28	23.09	39.55	0.000260606	1.06
C7_00880C_A	14.73	8.93	9.73	21.42	26.78	26.63	4.86E-05	1.06
C2_04660C_A	19.37	10.12	13.45	29.83	30.77	35.63	0.000159436	1.06
C2_03710W_A	0.58	0.44	0.14	0.58	0.85	1.18	0.137972779	1.06
C1_04110W_A	3.12	2.59	2.08	5.11	6.47	6.02	0.000621513	1.06
CR_07270C_A	1.20	0.53	0.26	1.04	1.47	1.97	0.141610722	1.06
C1_02500W_A	1.43	0.34	0.56	0.54	1.98	2.60	0.300473237	1.06
C4_01840C_A	53.05	74.49	79.05	206.99	117.96	141.43	0.002399299	1.05
CR_02630C_A	0.40	0.39	0.80	1.01	0.90	1.72	0.144333011	1.05
C2_00570W_A	23.97	17.62	17.59	41.67	45.73	44.35	2.80E-08	1.05
C2_04270W_A	3.84	2.24	1.95	4.13	5.73	8.03	0.019749469	1.05
C7_01500W_A	2.64	1.62	0.00	2.48	3.09	3.82	0.484607171	1.05
C6_04310W_A	13.85	10.42	10.20	18.52	28.65	29.81	0.000405145	1.05
C6_01720C_A	62.45	71.64	61.31	117.18	152.66	168.33	4.42E-06	1.05
C2_02120W_A	2.30	2.68	2.46	3.43	5.24	8.12	0.017898142	1.05
C1_07080W_A	23.93	25.53	20.99	39.91	57.90	59.71	6.03E-05	1.05
C6_01360W_A	21.32	6.80	6.81	11.03	33.60	32.36	0.145526441	1.04
C1_02700C_A	25.89	16.10	15.12	25.06	44.80	57.53	0.022394535	1.04
C1_05490C_A	34.40	41.10	8.69	89.48	43.70	54.42	0.119545414	1.04
C2_03490C_A	12.71	10.72	10.66	27.98	24.24	23.67	1.47E-08	1.04

C1_03740W_A	4.73	2.96	2.86	10.63	5.93	6.80	0.005082193	1.04
C5_05390C_A	191.90	145.12	149.41	206.44	548.18	317.18	0.016681896	1.04
C6_00590W_A	8.17	11.05	9.08	21.35	19.83	22.00	6.63E-06	1.04
C2_09980W_A	142.23	115.28	112.91	255.24	293.20	271.00	4.65E-10	1.04
C4_03120C_A	8.07	3.78	9.73	15.39	12.36	20.11	0.016743138	1.04
C5_00260W_A	23.47	16.61	28.16	50.04	47.92	53.23	6.11E-05	1.04
C1_10880W_A	3.04	1.90	3.60	6.88	7.01	4.94	0.007019508	1.04
C4_02850W_A	3.37	2.22	2.94	5.42	7.38	6.02	0.000191771	1.03
C3_00630W_A	4.33	4.33	3.92	7.20	9.76	10.92	1.41E-05	1.03
C7_01910C_A	9.73	6.01	7.60	19.95	14.69	16.69	0.000222716	1.03
C1_01110C_A	7.93	6.84	11.93	20.35	20.11	18.29	0.000797122	1.03
CR_09900C_A	1.07	0.41	0.63	2.27	1.94	0.37	0.240081379	1.03
CR_08870W_A	32.78	28.38	35.08	72.44	73.87	65.11	2.24E-08	1.03
C4_03720C_A	15.56	20.57	22.78	62.90	19.92	48.22	0.042265874	1.03
C6_00420W_A	13.76	9.93	14.41	22.57	27.89	33.30	0.000856763	1.03
C3_03830W_A	2.33	9.16	12.58	13.21	24.16	16.22	0.154389308	1.03
C1_00040W_A	80.49	58.58	58.38	124.45	152.24	155.28	1.13E-06	1.02
C1_02210W_A	21.20	23.89	16.47	54.38	42.71	38.02	0.000254302	1.02
C1_07170C_A	5.70	3.97	5.78	16.78	6.94	10.19	0.0175863	1.02
C7_00080C_A	27.44	0.15	19.68	26.85	40.32	33.85	0.613711527	1.02
C3_03110W_A	2.48	1.70	0.65	2.55	4.44	3.53	0.093425714	1.02
CR_10110W_A	10.74	20.33	20.42	38.61	38.52	36.68	0.00475222	1.02
CR_09600C_A	53.43	38.30	36.22	81.15	79.73	119.19	0.000164575	1.02
C1_05460W_A	9.79	12.00	8.51	22.35	21.26	22.88	3.80E-06	1.02
C1_13870W_A	9.99	8.55	10.37	23.52	19.24	20.36	1.43E-07	1.02
C6_03400C_A	61.05	42.30	45.49	77.46	104.67	143.07	0.001084228	1.02
C1_01850C_A	17.79	17.13	20.17	39.75	37.46	43.31	6.35E-09	1.02
C2_00010W_A	10.04	25.09	34.30	43.61	54.81	55.18	0.060779469	1.02
C2_07660W_A	3.98	3.40	3.82	6.42	11.61	6.26	0.005030971	1.01
C1_06970C_A	24.58	23.45	24.45	54.17	61.24	42.28	2.00E-05	1.01
C4_02600C_A	3.23	3.35	1.95	7.07	5.44	6.12	0.002042091	1.01
C2_03760C_A	38.50	39.00	50.08	114.94	81.82	81.24	0.000172118	1.01
C1_02840W_A	7.04	7.12	10.00	15.32	16.73	20.67	0.000188667	1.01
C3_07310C_A	152.70	105.29	98.66	251.00	224.05	297.15	1.82E-05	1.01
C1_04100C_A	212.02	180.85	302.34	624.91	420.71	465.57	0.001512589	1.01
C1_04480C_A	21.98	21.07	18.45	43.05	44.17	46.33	1.76E-12	1.01
C4_00270W_A	592.52	578.07	616.68	1314.26	1321.09	1244.26	7.34E-13	1.01
C4_07250C_A	74.42	50.62	63.21	108.33	157.28	140.87	7.72E-05	1.01
C4_05240C_A	27.81	7.00	3.26	24.38	26.97	29.00	0.209318362	1.01
C2_02440W_A	5.83	1.30	4.28	10.02	6.62	7.76	0.089914398	1.01
C5_04090C_A	50.70	44.07	38.58	73.34	86.55	130.07	0.000678499	1.01
C2_06710W_A	1.87	2.34	2.11	3.61	4.23	5.95	0.004366919	1.00
C1_11670W_A	81.36	83.82	152.13	232.06	248.59	208.53	0.00480484	1.00
C4_06610C_A	60.68	63.30	72.13	151.40	140.52	133.27	5.57E-08	1.00
C1_10180C_A	30.09	25.08	30.18	56.08	67.01	61.31	1.20E-08	1.00
C4_01720C_A	139.61	90.82	84.47	221.60	224.32	231.03	3.51E-05	1.00
C1_10190W_A	28.69	19.46	17.87	39.99	58.00	43.75	0.000852617	1.00
CR_05960W_A	3.79	12.98	14.32	18.70	21.63	28.05	0.119748653	1.00
C2_07740W_A	23.16	16.03	10.80	39.84	32.24	35.29	0.002430513	1.00
C3_00830C_A	7.46	5.11	5.74	13.59	9.17	16.90	0.001255467	1.00
C3_00560C_A	6.95	6.70	8.01	15.29	15.59	15.99	1.07E-07	1.00
CR_07600W_A	3.20	2.84	2.43	5.96	5.97	6.35	1.07E-07	1.00
C3_06890W_A	16.14	20.64	22.09	39.87	44.60	43.15	1.84E-05	1.00
C6_04540C_A	30.68	31.50	31.48	79.78	64.78	57.31	7.97E-06	1.00
C1_02120C_A	58.83	76.36	65.20	210.85	91.91	131.78	0.011026306	1.00
C1_10160W_A	105.16	105.42	181.53	286.23	276.73	285.78	0.001566656	1.00
CR_04830C_A	1.93	1.42	1.10	2.85	3.02	3.72	0.001609384	1.00
C7_00050C_A	23.41	2.08	25.00	34.37	36.81	36.06	0.253424736	1.00
C2_01110C_A	103.40	100.64	100.35	219.91	218.15	218.07	2.50E-16	1.00
C1_14290C_A	172.13	127.85	126.64	280.36	335.56	298.38	6.06E-07	1.00
C1_09450C_A	2.59	2.72	2.31	5.65	4.87	5.90	1.13E-07	0.99
C6_03240W_A	0.73	0.74	0.98	0.86	1.60	2.81	0.135854916	0.99
C1_00590W_A	74.38	69.37	83.75	173.86	162.04	153.17	1.79E-08	0.99

C2_08330W_A	10.37	10.12	10.39	19.11	20.60	26.79	4.05E-06	0.99
CR_05920C_A	32.50	30.61	38.34	89.13	72.31	55.98	0.000364281	0.99
C3_01170W_A	8.53	4.34	4.24	8.73	11.90	15.89	0.017672824	0.99
C5_01130W_A	9.95	7.29	9.22	17.18	18.04	21.55	7.96E-06	0.99
C3_05410W_A	9.67	9.05	8.07	18.15	18.06	21.23	1.64E-09	0.99
CR_07030C_A	2.11	1.50	1.81	5.38	4.27	1.96	0.08177934	0.99
C1_10460W_A	8.39	8.80	11.13	20.23	18.13	22.46	2.15E-05	0.99
C7_00090C_A	11.69	0.14	16.10	22.66	18.68	17.54	0.604929354	0.98
C2_10730W_A	0.50	0.51	0.97	1.71	1.47	1.12	0.110521527	0.98
C4_01710C_A	154.62	118.14	110.22	286.06	287.11	238.49	7.27E-06	0.98
C2_01020W_A	394.87	410.49	444.64	856.25	936.25	874.87	1.74E-09	0.98
C2_02310W_A	29.52	27.78	34.82	61.28	62.52	72.86	3.00E-07	0.98
C1_10030W_A	2.17	1.36	2.14	4.68	3.44	3.98	0.002502718	0.98
C1_00430W_A	24.02	20.18	19.53	44.42	46.00	44.93	6.84E-11	0.98
C2_02240C_A	20.15	12.15	18.01	28.02	39.93	38.49	0.000714926	0.98
C2_03550C_A	16.28	11.84	11.37	27.09	26.95	29.58	5.23E-07	0.98
C1_06200W_A	1.95	3.99	6.46	8.95	8.33	9.35	0.066569723	0.98
C2_04170C_A	110.57	140.92	206.83	377.61	265.82	336.58	0.005470913	0.97
C2_01070W_A	5.24	3.93	4.32	8.62	10.83	9.03	9.69E-05	0.97
C2_08260W_A	49.66	41.44	89.25	131.32	126.99	123.81	0.0109697	0.97
C4_00010W_A	84.03	63.13	67.30	124.14	214.14	109.93	0.008028358	0.97
CR_06780W_A	27.26	22.44	23.61	51.82	47.60	55.19	7.33E-11	0.96
CR_10800C_A	12.35	30.08	15.06	34.91	49.64	37.94	0.040914497	0.96
CR_04240C_A	5.81	3.21	6.40	11.61	10.04	10.63	0.003383891	0.96
C1_13410W_A	15.57	15.32	15.47	27.89	36.17	33.58	2.16E-05	0.96
C5_04120C_A	2.43	3.38	1.93	5.92	4.96	5.48	0.00750386	0.96
C1_14090W_A	36.77	42.38	54.69	105.88	82.40	93.76	0.000490099	0.96
C1_08010W_A	58.71	50.49	47.91	102.04	112.93	114.48	5.01E-10	0.96
C1_01860W_A	24.60	24.11	23.77	47.99	47.31	57.19	1.17E-09	0.96
C6_02320C_A	4.36	2.06	2.59	6.45	8.63	3.52	0.07075737	0.96
C6_03720W_A	8.02	8.47	10.08	19.74	16.56	19.48	3.57E-06	0.95
C2_02180W_A	9.10	6.20	5.45	15.13	10.27	18.06	0.004248153	0.95
C2_06510W_A	16.90	13.89	12.55	31.03	24.36	35.30	3.21E-05	0.95
C5_05430W_A	22.33	11.86	17.03	32.35	38.04	36.05	0.001381584	0.95
CR_02810W_A	41.18	52.42	52.77	127.81	82.23	97.23	0.000786394	0.95
C4_07100C_A	3.33	3.51	2.06	6.69	6.73	5.10	0.003362366	0.95
C1_06640C_A	35.13	11.76	2.62	20.49	16.88	66.03	0.391805446	0.95
C5_00900C_A	8.95	10.81	4.87	20.86	15.62	14.89	0.021340702	0.95
C3_02230C_A	1.85	1.02	0.88	2.86	2.15	2.79	0.013687392	0.95
CR_09570W_A	6.33	4.09	7.41	11.96	15.94	8.95	0.022513587	0.95
C6_02910W_A	2.50	2.00	2.39	6.04	3.80	4.58	0.008736707	0.94
CR_03640C_A	14.74	6.53	19.71	30.87	23.91	30.02	0.044823647	0.94
C6_00920W_A	49.94	43.32	45.90	87.20	109.82	90.89	1.99E-06	0.94
C2_05980C_A	2.59	3.69	3.84	7.86	6.50	6.67	0.004488142	0.94
C1_12100C_A	4.10	3.80	7.06	5.63	11.84	13.67	0.059004275	0.94
C3_05030W_A	24.09	43.86	28.32	66.84	59.30	75.38	0.005973099	0.94
CR_05120W_A	10.15	7.91	6.97	20.67	15.59	15.31	0.000252095	0.94
CR_10860C_A	64.96	56.53	59.50	124.26	128.73	120.76	1.87E-11	0.94
C3_06260C_A	1.73	1.96	1.78	4.48	3.49	3.39	0.000906927	0.94
CR_09500C_A	11.49	5.87	10.88	10.23	28.19	19.43	0.073601349	0.94
CR_09620C_A	8.64	4.75	6.93	14.81	11.50	15.54	0.004027771	0.93
CR_07920W_A	11.66	11.66	11.13	23.28	22.99	24.92	4.13E-11	0.93
C3_06490W_A	74.26	76.07	83.78	167.60	153.39	162.50	3.34E-09	0.93
C4_00630C_A	14.69	12.67	12.50	24.51	28.19	29.37	1.11E-06	0.93
C4_03430W_A	19.11	23.43	15.15	45.81	35.69	37.76	0.000916165	0.93
C2_09310C_A	7.79	9.32	10.52	18.47	18.07	20.39	0.000396031	0.93
C6_01040C_A	0.48	0.63	0.45	0.87	1.11	1.27	0.024123511	0.93
C1_12140W_A	13.39	24.45	21.62	37.82	40.00	45.74	0.007942462	0.93
CR_03570C_A	2.24	3.34	3.54	6.64	5.09	7.08	0.011881913	0.92
CR_02910W_A	8.88	10.30	13.98	20.24	25.60	22.34	0.001616952	0.92
C3_04920C_A	58.93	57.13	54.86	109.53	110.81	130.40	2.77E-10	0.92
C3_07460W_A	13.49	12.88	13.32	27.62	25.61	28.06	4.23E-12	0.92
C4_04420W_A	23.17	18.86	17.00	44.19	43.33	32.58	0.000220562	0.92

C2_04280W_A	26.08	23.56	28.84	55.18	52.01	53.41	1.31E-08	0.92
C5_02380W_A	36.68	52.52	56.31	122.36	80.25	97.26	0.003757697	0.92
CR_03100W_A	1.46	1.45	1.39	3.19	2.59	2.98	4.01E-05	0.92
C1_09690W_A	105.78	159.43	122.04	264.68	242.48	290.03	0.000457061	0.92
C3_04030C_A	2.81	2.39	2.19	3.20	5.12	6.83	0.013366708	0.92
C1_05180C_A	7.88	5.28	4.17	7.89	11.30	16.09	0.075564885	0.91
CR_09690C_A	12.36	16.31	15.53	32.32	26.95	31.22	9.04E-05	0.91
C2_09020W_A	8.20	10.92	9.67	23.36	18.22	17.21	0.000758168	0.91
CR_08540C_A	13.75	12.15	12.19	26.55	25.10	25.85	9.13E-12	0.91
C4_06930C_A	16.38	18.20	17.40	33.54	33.58	38.80	8.35E-07	0.91
C2_09520C_A	20.03	22.04	24.16	47.17	46.33	41.14	8.36E-06	0.91
C7_00570W_A	5.32	3.38	2.20	6.59	7.47	7.90	0.01741198	0.91
CR_06490C_A	23.54	19.62	17.89	39.08	38.07	46.66	4.65E-07	0.91
C7_03400C_A	1.98	2.79	1.40	4.33	3.98	4.20	0.011976976	0.90
C3_02610C_A	24.25	43.78	38.91	72.37	68.95	77.14	0.00629129	0.90
C2_05130W_A	2.78	11.62	10.64	5.85	40.30	4.31	0.430608308	0.90
C7_01520W_A	2.73	3.20	2.50	5.76	4.49	6.83	0.001628615	0.90
C1_12610W_A	7.44	8.94	7.76	15.34	16.05	17.29	9.58E-05	0.90
C6_02060W_A	43.45	30.50	29.63	69.12	69.50	68.68	1.33E-05	0.90
C2_02980C_A	38.04	36.98	34.64	70.96	74.27	75.46	8.49E-12	0.90
C1_07520C_A	8.10	5.43	6.67	10.33	16.20	13.85	0.001896201	0.90
C1_12250C_A	16.11	18.11	21.73	35.86	42.12	34.59	0.000269855	0.90
C5_04100W_A	16.13	13.75	13.80	29.27	27.24	31.21	2.08E-09	0.89
CR_00020W_A	92.38	74.09	67.07	147.61	175.95	142.97	3.78E-05	0.89
C4_01910W_A	6.11	5.78	5.31	13.22	10.17	11.21	3.19E-05	0.89
C1_07160C_A	844.13	504.90	1131.14	2923.74	1357.10	641.49	0.188341199	0.89
C5_00930C_A	252.64	319.89	173.09	530.10	529.85	435.40	0.006063579	0.89
C4_03140C_A	4.15	2.43	1.78	5.29	6.08	5.23	0.041819742	0.89
C5_01490C_A	10.89	8.32	9.12	20.59	16.92	19.19	2.27E-06	0.89
C1_09020W_A	13.89	9.44	10.77	22.11	21.87	24.21	4.31E-05	0.89
C1_05370C_A	6.81	6.25	6.74	12.62	13.11	13.93	4.61E-09	0.89
C6_00400C_A	5.62	5.44	5.36	10.74	10.62	11.56	4.65E-07	0.89
C3_01000W_A	1.50	1.22	0.58	2.48	1.82	2.32	0.04923674	0.89
C4_05150W_A	29.90	30.56	35.01	63.61	70.18	57.10	1.92E-05	0.89
C4_05270C_A	4.05	3.33	3.32	8.18	5.35	8.00	0.007984362	0.89
CR_04850C_A	1.74	1.10	0.80	1.39	2.24	3.67	0.175189153	0.89
C3_00240C_A	119.10	71.89	88.15	162.67	152.98	240.33	0.002521874	0.89
C6_04170C_A	3.64	2.49	1.98	4.81	4.95	6.36	0.00506671	0.89
C2_04370W_A	2.57	1.28	0.97	2.61	0.51	6.63	0.397706655	0.88
C2_05490W_A	7.43	4.82	4.88	10.49	12.63	11.15	0.006765837	0.88
C1_08670W_A	3.20	2.77	3.55	7.17	5.35	6.48	0.000253919	0.88
C7_01490W_A	8.51	8.07	0.00	9.43	11.50	11.84	0.72861557	0.88
C3_04370C_A	1.04	1.41	1.16	2.20	2.60	2.38	0.002312575	0.88
C1_08070W_A	133.01	105.62	115.84	271.03	257.09	171.59	0.001536472	0.88
C3_02700W_A	0.52	0.24	0.17	0.42	0.70	0.76	0.171348573	0.88
C3_00290W_A	163.80	141.00	140.82	239.45	232.77	417.87	0.003230196	0.88
C5_03790W_A	4.10	2.25	2.42	5.39	6.84	5.26	0.037971418	0.88
C1_11610C_A	7.48	6.65	8.96	16.01	16.98	12.73	0.001008614	0.88
C1_10330C_A	3.00	1.97	2.21	6.06	2.64	5.55	0.058126241	0.88
C5_02990W_A	0.70	2.70	1.36	2.83	2.31	4.40	0.264142768	0.88
C1_03310W_A	48.00	39.52	40.03	75.61	86.02	90.83	6.11E-08	0.88
C6_02900C_A	1.40	1.08	1.04	1.57	2.18	3.25	0.054115788	0.88
CR_09660W_A	1.77	1.45	1.13	3.00	2.33	3.37	0.02002207	0.87
C1_12890W_A	2.76	2.41	2.60	4.60	5.41	5.35	0.000325381	0.87
C2_05090W_A	2.04	2.00	2.25	4.77	3.60	4.09	0.000427027	0.87
CR_08710W_A	68.26	34.00	37.29	56.31	118.79	97.60	0.048541993	0.87
C2_09510C_A	6.91	5.22	5.05	10.98	10.88	12.11	1.87E-05	0.87
C2_08120W_A	65.99	64.80	68.65	145.07	117.26	132.52	1.38E-07	0.87
CR_02980C_A	1.62	1.23	2.11	3.09	3.70	3.01	0.019990239	0.87
C4_07080C_A	4.57	3.35	2.93	7.98	6.08	7.32	0.000812931	0.87
C5_02120C_A	10.34	5.03	5.71	13.31	12.62	15.35	0.010645201	0.87
C6_03020W_A	5.41	4.17	3.64	11.38	8.24	6.36	0.012871048	0.87
C1_05440C_A	14.62	28.06	22.60	49.03	38.11	42.95	0.015798051	0.87

C6_03840C_A	2.16	1.99	2.09	4.56	4.84	2.84	0.007723471	0.87
C7_03700C_A	0.56	0.22	0.29	0.72	1.14	0.25	0.370954913	0.87
C3_07550C_A	2.00	2.56	1.58	6.27	3.13	2.73	0.091470833	0.87
C2_05820W_A	5.85	5.24	6.05	11.48	10.85	11.35	1.85E-06	0.86
C3_00370C_A	7.78	7.45	7.12	13.52	14.73	15.76	3.75E-05	0.86
C2_09680W_A	32.14	37.16	37.07	41.37	85.71	82.86	0.024292545	0.86
C6_01050W_A	6.80	6.42	7.24	13.68	9.65	17.09	0.002810546	0.86
CR_04840C_A	13.03	9.89	9.06	23.89	17.93	20.81	0.000262014	0.86
C1_01130W_A	7.89	8.03	9.19	17.92	15.43	15.90	1.04E-05	0.86
C2_01450C_A	0.66	0.74	0.75	1.98	1.18	1.17	0.176461828	0.86
CR_07120C_A	16.66	10.52	13.12	24.37	26.60	27.62	0.000195124	0.86
C4_05660C_A	3.13	3.93	5.03	9.27	7.19	7.24	0.007902326	0.86
C6_03830W_A	16.40	15.48	15.23	33.64	37.40	20.53	0.009471609	0.86
C7_01840W_A	5.47	3.14	2.78	8.12	7.01	6.94	0.009827455	0.86
C1_06360W_A	2.75	0.94	4.85	6.60	5.75	4.23	0.216085808	0.86
CR_07130C_A	0.62	1.40	0.95	2.06	1.14	2.71	0.165863085	0.86
C2_04200W_A	3.12	2.99	2.57	6.03	5.77	5.15	8.22E-05	0.86
C4_05440C_A	18.57	17.40	17.77	36.27	29.56	39.31	1.90E-06	0.85
C2_09070C_A	5.13	3.44	3.53	5.81	8.09	9.66	0.00639249	0.85
C4_02750W_A	1.14	1.23	1.48	1.82	2.61	3.17	0.150662234	0.85
C4_02460W_A	3.84	1.28	0.35	1.73	5.54	3.18	0.431519151	0.85
C7_00390W_A	100.06	34.86	74.25	131.83	135.29	134.72	0.045066252	0.85
C1_02200C_A	10.64	15.13	9.64	27.21	19.63	22.44	0.008655159	0.85
C1_13200C_A	1.30	0.64	0.55	1.82	1.88	1.13	0.166730708	0.85
CR_02610C_A	28.62	14.89	12.08	23.65	39.20	43.97	0.06345532	0.84
C1_01630W_A	31.23	46.40	42.96	88.86	67.34	79.43	0.003349886	0.84
C1_02270C_A	6.86	4.58	5.93	13.69	11.17	8.63	0.011715406	0.84
C1_10260C_A	2.60	1.97	2.19	5.01	4.73	3.35	0.03322613	0.84
C1_08740C_A	70.23	75.01	72.14	148.07	130.40	142.83	3.06E-08	0.84
C2_03780C_A	14.74	11.27	12.48	23.15	24.56	26.57	4.56E-07	0.84
C2_06290C_A	193.95	200.62	239.22	442.69	383.73	400.36	2.11E-05	0.84
C5_00270W_A	453.54	147.55	708.60	809.95	898.61	804.39	0.169052967	0.84
C1_13470W_A	1.48	3.73	4.62	6.45	5.51	7.28	0.123846086	0.84
CR_10700W_A	9.92	12.20	15.47	27.58	22.67	22.56	0.005121505	0.84
C7_00920C_A	5.60	3.39	4.06	6.31	6.85	12.10	0.040379473	0.84
C4_02150C_A	1.35	1.06	1.05	1.98	2.39	2.32	0.001157925	0.83
C3_04590W_A	68.35	63.33	67.41	101.24	187.69	91.82	0.031828551	0.83
C1_05740C_A	0.48	0.50	0.44	0.68	0.69	1.31	0.204358117	0.83
C4_03790W_A	17.92	13.26	13.03	24.80	27.15	32.85	0.00019041	0.83
C1_11310C_A	37.05	27.13	27.66	57.60	58.58	59.13	4.30E-06	0.83
C1_11680C_A	7.88	6.77	15.70	18.18	16.65	23.77	0.105427249	0.83
C5_04720C_A	33.25	18.49	21.16	48.76	42.94	47.01	0.003502799	0.83
C1_09880C_A	2.38	5.09	4.47	5.57	10.53	6.81	0.155512249	0.83
C1_07830C_A	7.91	7.04	5.73	10.96	13.79	14.89	0.001981743	0.82
C1_08100W_A	18.71	21.91	18.71	48.54	45.37	18.88	0.082612467	0.82
C6_04580W_A	27.93	25.83	24.48	41.83	54.33	53.33	2.13E-05	0.82
CR_01900C_A	20.80	61.37	26.58	65.74	74.55	70.63	0.120663622	0.82
C4_03030C_A	1.01	1.06	0.91	2.01	1.80	1.93	0.002961942	0.82
C1_06540C_A	2.95	2.53	2.34	4.03	5.35	5.59	0.005871826	0.82
C1_12580W_A	15.19	10.58	13.68	21.33	25.02	28.84	0.000397402	0.82
C5_00490C_A	5.81	4.45	4.35	7.44	10.37	9.99	0.000833478	0.82
C1_07870C_A	140.26	105.32	118.42	227.36	219.11	246.12	1.21E-07	0.82
C1_13660W_A	2.87	3.14	3.66	5.94	6.42	6.13	0.000381238	0.82
C7_00400W_A	69.78	0.06	80.39	57.20	120.61	103.81	0.749864047	0.82
C1_04370C_A	13.54	11.48	11.63	27.62	20.54	21.73	0.000646425	0.82
C3_07540C_A	6.69	6.86	5.66	18.24	8.13	10.32	0.05898052	0.82
C5_04960W_A	7.64	3.72	4.14	6.99	11.98	10.24	0.049331184	0.82
C7_01810W_A	76.65	47.52	50.42	108.33	112.76	108.34	0.00074785	0.82
C2_00430C_A	14.77	18.16	19.83	40.93	24.84	35.16	0.009417951	0.82
CR_03970C_A	8.64	4.67	4.59	7.71	11.91	14.34	0.068552758	0.81
C1_13190W_A	46.65	38.41	36.45	82.57	73.69	74.10	7.21E-06	0.81
C2_05350C_A	3.17	3.21	3.06	4.67	6.30	7.01	0.009661295	0.81
C1_09060C_A	8.87	3.38	2.98	6.98	9.47	12.16	0.165568295	0.81

C2_07220W_A	25.58	25.70	25.76	47.07	50.82	48.22	3.67E-07	0.81
C7_03190C_A	1.84	2.01	2.07	2.21	4.37	4.69	0.165568295	0.81
C2_10700C_A	29.24	21.11	24.45	42.66	46.53	52.30	3.33E-05	0.81
C4_01410W_A	11.54	9.06	9.72	16.60	18.88	21.90	3.82E-05	0.81
C5_05490C_A	51.98	31.19	36.15	66.66	84.08	73.10	0.002421281	0.81
C3_07790W_A	27.96	20.44	24.15	40.01	56.07	40.29	0.002471537	0.81
CR_02500W_A	2.09	2.11	1.73	4.90	3.30	3.07	0.024329407	0.81
CR_00800C_A	0.47	3.27	4.16	5.68	4.42	5.15	0.349979015	0.81
C3_06200C_A	1.69	4.31	2.74	6.14	4.09	6.53	0.142564803	0.81
CR_01170W_A	7.89	3.26	6.72	10.40	10.95	12.14	0.035955902	0.81
C6_02230W_A	2.64	1.53	0.08	2.39	2.54	2.99	0.482249229	0.81
C5_02040W_A	15.40	5.42	15.67	20.73	22.60	25.08	0.09291277	0.81
CR_10750C_A	15.86	8.30	9.97	22.31	24.46	17.24	0.034462056	0.80
C7_00150W_A	105.21	0.83	119.50	126.15	147.76	144.71	0.693522786	0.80
C4_02710C_A	17.49	28.80	21.53	28.40	54.51	45.69	0.05154728	0.80
C5_04280C_A	14.89	21.74	16.41	36.68	33.93	29.65	0.004441751	0.80
CR_03380W_A	1.08	1.08	1.18	2.25	2.21	1.88	0.039856947	0.80
C1_01520C_A	116.94	55.52	87.99	165.43	165.64	153.21	0.013324182	0.80
C6_01590W_A	20.48	16.75	18.72	33.88	33.62	37.56	5.96E-08	0.80
CR_04730W_A	45.10	63.41	53.20	104.12	103.99	96.51	0.001797303	0.80
C7_01900W_A	54.80	36.48	40.40	85.43	80.74	79.45	0.000153367	0.80
C1_09100W_A	2.73	1.87	1.82	4.14	3.68	4.32	0.028162297	0.80
C6_00980C_A	18.59	12.31	16.12	25.62	27.72	34.60	0.001183534	0.79
C1_01080W_A	6.83	3.11	3.48	8.17	8.30	8.48	0.041350188	0.79
CR_02070C_A	748.23	679.03	834.84	1361.00	1437.73	1435.34	9.68E-07	0.79
C6_01290C_A	8.30	6.48	6.57	11.55	11.96	16.50	0.000837515	0.79
C1_07990C_A	10.07	11.01	10.48	16.75	20.85	21.60	0.000201122	0.79
C1_04200C_A	19.35	14.80	13.62	22.91	30.20	36.17	0.004727188	0.79
CR_01710W_A	0.63	0.97	0.69	1.21	1.35	1.70	0.163806447	0.79
C5_00860W_A	7.44	9.85	14.15	18.33	18.17	22.58	0.037620302	0.79
C5_02020C_A	23.26	28.46	28.19	45.73	50.67	53.58	0.000196992	0.79
C3_06910C_A	13.30	9.51	9.33	13.72	23.38	22.75	0.015164008	0.79
C2_00610C_A	84.75	138.44	165.57	343.50	174.53	214.43	0.085506102	0.79
C3_01550C_A	57.10	72.67	95.76	135.54	142.55	144.95	0.006840665	0.79
C1_05660C_A	5.80	5.75	6.19	10.47	10.90	11.73	3.32E-07	0.79
CR_01740W_A	68.48	65.83	70.59	131.40	126.75	123.44	2.27E-07	0.79
C5_05360C_A	17.35	18.18	13.33	25.38	27.69	38.35	0.003230196	0.79
C1_00450C_A	33.28	48.68	53.52	109.99	66.36	77.73	0.033854497	0.79
C7_04360C_A	27.01	110.42	71.64	93.06	113.06	192.52	0.236958813	0.78
CR_00600C_A	12.33	5.24	6.09	18.05	12.18	13.16	0.070903371	0.78
C1_00340W_A	22.80	24.88	22.18	43.37	36.24	50.73	0.000287262	0.78
C7_03850W_A	0.24	0.55	0.23	0.59	0.67	0.68	0.237229727	0.78
C1_07190W_A	19.43	14.20	14.40	26.73	31.68	30.33	0.000132164	0.78
C2_01700C_A	7.69	4.64	4.90	10.89	9.08	11.79	0.005540966	0.78
C4_02330C_A	28.33	19.14	17.06	30.86	38.26	50.14	0.018786971	0.78
C1_01390C_A	7.59	7.19	5.95	11.54	14.32	12.40	0.000580945	0.77
C1_04180W_A	626.47	643.75	733.11	1198.36	1251.98	1249.95	3.83E-06	0.77
CR_07020W_A	30.52	39.36	35.77	67.51	57.40	70.99	0.000880522	0.77
C6_02520W_A	33.58	31.59	16.20	43.10	47.42	59.51	0.040671893	0.77
C1_00370W_A	6.63	8.52	6.82	6.90	14.83	18.82	0.156213322	0.77
C3_07290W_A	6.86	5.75	5.52	11.35	9.38	12.60	0.000204376	0.77
C1_09170W_A	7.60	7.43	6.03	12.30	14.05	12.32	0.0002536	0.77
CR_10420W_A	4.29	4.22	7.62	9.69	9.17	10.90	0.035033028	0.77
C4_04970C_A	45.10	57.16	69.38	112.33	94.45	110.15	0.00675488	0.77
C7_02930C_A	10.71	2.58	12.70	25.79	2.62	19.27	0.460906154	0.77
C3_02260C_A	37.17	14.81	29.62	40.60	59.22	48.14	0.070617485	0.76
C1_08110W_A	6.07	6.95	5.71	14.16	11.78	8.39	0.016207825	0.76
C5_00010W_A	32.04	43.98	27.88	76.14	58.74	56.30	0.017435644	0.76
C2_06620W_A	1.46	0.92	0.53	0.96	2.11	2.24	0.260741139	0.76
C6_04570W_A	53.60	50.87	41.93	73.45	88.43	106.69	0.000781206	0.76
C5_00640C_A	7.30	4.47	4.78	11.07	6.41	12.82	0.053135528	0.76
C3_07320W_A	14.51	12.59	10.49	21.28	21.85	25.63	9.88E-05	0.76
C2_08920W_A	14.05	5.37	8.08	24.39	7.44	18.00	0.216605232	0.76

C1_01710W_A	24.59	11.76	11.52	31.49	27.76	27.11	0.048180467	0.76
C3_06250W_A	42.29	34.31	39.99	80.03	62.17	70.43	9.96E-05	0.76
C2_02540W_A	0.78	0.43	0.35	0.61	1.01	1.22	0.141779608	0.76
CR_05080W_A	21.79	15.20	16.82	26.91	31.14	40.02	0.002752088	0.76
C1_01260C_A	9.29	6.04	8.59	13.43	11.95	18.40	0.024715837	0.76
C3_00210C_A	3.75	2.14	7.95	10.40	1.80	13.32	0.442653457	0.76
C7_02170C_A	38.06	25.62	50.32	31.74	66.14	111.47	0.187393408	0.76
C2_01730W_A	1.41	0.66	0.62	1.28	0.86	2.93	0.367821358	0.76
C3_04080W_A	196.50	177.58	171.95	325.21	310.37	360.51	1.30E-08	0.76
CR_01420W_A	16.41	11.18	11.83	23.56	26.24	21.46	0.001797303	0.75
C5_04750C_A	14.32	10.18	13.49	25.98	22.43	20.48	0.001487782	0.75
C1_08230C_A	18.09	16.99	19.34	32.43	32.08	34.62	2.40E-07	0.75
C1_03610C_A	25.56	1.82	26.47	29.97	32.73	34.05	0.441766185	0.75
CR_02250C_A	4.43	3.65	4.35	7.06	7.34	8.23	7.34E-05	0.75
CR_03310C_A	39.25	31.50	35.44	64.54	59.21	69.27	5.41E-07	0.75
C7_03230C_A	9.38	3.81	3.42	5.67	10.94	13.41	0.257678769	0.75
C2_05220C_A	4.84	2.84	3.03	6.09	6.38	6.90	0.007897254	0.75
C2_03090C_A	29.74	31.33	34.47	62.03	55.67	56.18	7.89E-05	0.75
CR_04170W_A	14.10	9.59	11.66	21.05	20.89	22.06	0.000167622	0.75
C1_08870C_A	3.60	3.21	3.08	6.28	5.13	6.61	0.000968393	0.75
C2_08300C_A	102.70	59.22	63.13	120.77	127.92	157.42	0.010226177	0.75
C1_04330W_A	33.11	27.50	33.96	59.51	54.34	57.53	4.42E-06	0.75
CR_08440W_A	1.70	1.42	1.44	2.55	2.86	2.87	0.005160557	0.75
C3_01200W_A	0.61	0.11	0.33	0.55	0.55	0.78	0.305311686	0.74
CR_07810W_A	0.58	1.11	0.69	1.33	0.46	2.59	0.409152976	0.74
C2_08490W_A	10.45	6.75	6.67	12.35	13.08	17.63	0.010313642	0.74
C2_05520W_A	8.34	10.97	7.79	19.08	16.53	13.45	0.022662328	0.74
CR_08920W_A	11.42	13.01	14.41	15.13	26.90	28.53	0.037620302	0.74
C2_10090C_A	0.21	0.13	0.18	0.24	0.37	0.32	0.24442765	0.74
C7_02950C_A	4.03	0.96	4.87	8.44	3.57	5.68	0.330852127	0.74
C4_06730C_A	47.36	46.01	43.34	80.60	78.82	87.74	1.35E-08	0.74
C5_01590W_A	2.79	1.92	1.31	2.58	4.60	3.63	0.116819474	0.74
C6_03180C_A	32.69	39.53	27.62	41.29	73.13	66.27	0.030715067	0.74
C3_06300W_A	7.20	6.41	6.13	12.78	11.33	11.48	5.50E-06	0.74
C4_04730W_A	10.78	7.51	4.21	11.61	18.11	10.31	0.130152019	0.74
C4_05900C_A	21.62	34.66	23.42	40.79	68.73	34.15	0.100427025	0.74
C4_06250W_A	22.22	15.03	13.24	27.00	32.63	30.73	0.006196151	0.74
C4_00420C_A	33.15	38.25	34.18	47.28	75.31	68.07	0.007942462	0.74
C7_03980W_A	23.50	35.70	22.98	35.06	44.84	69.92	0.074035197	0.74
C2_04850C_A	88.38	94.40	87.86	149.78	160.01	178.79	3.83E-06	0.74
CR_09150W_A	6.22	4.92	5.05	7.95	9.89	11.41	0.013501064	0.74
C3_07970C_A	95.57	88.67	85.76	153.01	167.87	164.67	1.98E-07	0.74
C2_00240C_A	29.35	24.12	23.80	45.76	50.02	42.75	0.000110582	0.74
C6_03790C_A	0.58	1.00	0.09	0.66	1.10	1.24	0.489638746	0.74
C1_12350W_A	2.78	1.57	2.09	3.43	3.13	5.01	0.036760337	0.74
CR_05030W_A	16.36	12.59	12.59	25.08	23.14	26.36	1.89E-05	0.74
C6_01560W_A	5.07	4.02	3.20	6.99	7.61	7.55	0.00938509	0.73
C5_02910C_A	3.49	2.38	2.57	4.17	5.50	5.47	0.008367961	0.73
C3_07810C_A	101.09	100.41	98.44	222.56	162.72	152.04	0.002749843	0.73
C1_07810C_A	3.49	0.54	2.52	2.71	5.03	3.80	0.348012544	0.73
C7_02310C_A	0.63	0.58	0.38	0.94	0.92	1.02	0.18844536	0.73
C1_00350C_A	19.58	33.33	16.43	39.81	34.88	51.06	0.073807877	0.73
C4_03780C_A	1.52	0.86	1.16	2.15	1.82	2.39	0.069248449	0.73
C1_07270W_A	9.49	5.22	14.69	21.48	15.85	15.13	0.130815895	0.73
CR_02620C_A	50.21	25.43	29.60	21.44	75.81	90.06	0.266163298	0.73
CR_04530W_A	30.04	36.43	44.55	74.35	62.81	62.13	0.007258167	0.73
C3_05430W_A	0.39	2.04	2.09	0.45	0.13	7.95	0.749220077	0.73
C2_00360C_A	43.19	49.34	44.48	98.53	89.41	56.44	0.026068992	0.73
CR_05180C_A	60.25	39.98	70.86	118.91	98.24	87.20	0.023809062	0.73
C2_06740W_A	6.63	1.58	2.75	5.71	4.76	8.85	0.258631422	0.73
C3_01150C_A	2.00	0.79	1.25	1.14	2.76	3.27	0.307342701	0.72
C4_06410W_A	1.91	0.79	0.95	1.08	2.09	3.33	0.259842545	0.72
C4_03890W_A	10.08	7.84	5.30	9.30	13.59	18.49	0.076534796	0.72

C3_07780C_A	35.85	27.97	28.63	50.91	61.60	51.48	0.000332573	0.72
C7_02550C_A	13.17	12.39	10.84	20.70	23.12	21.00	0.000529504	0.72
C5_02190C_A	168.58	138.36	163.56	252.67	292.25	292.31	1.98E-05	0.72
C1_04260W_A	1493.14	1482.26	1792.16	2577.60	2992.78	2929.51	0.000175634	0.72
C5_02610C_A	4.48	2.91	2.71	7.53	4.53	5.84	0.038669893	0.72
C3_02540C_A	10.62	10.22	14.37	20.20	19.72	22.83	0.004041245	0.72
C1_01840C_A	84.61	86.78	111.16	164.80	159.28	180.17	0.000759992	0.72
CR_04600W_A	10.06	7.08	6.91	15.23	13.79	13.57	0.002302974	0.72
C4_04700W_A	104.74	71.26	53.08	118.34	125.44	160.45	0.027708804	0.71
C6_03460W_A	14.55	13.40	12.22	24.75	24.23	22.10	2.34E-05	0.71
C1_02060W_A	35.36	38.28	37.94	65.73	63.44	68.72	0.000131817	0.71
C2_04180C_A	7.52	5.29	7.20	10.70	11.72	13.01	0.003607134	0.71
C1_06250W_A	1.23	1.50	1.11	2.10	1.69	3.07	0.072043717	0.71
C1_03660W_A	12.98	14.55	16.32	26.89	22.85	27.74	0.011355703	0.71
C2_06160W_A	20.73	6.55	9.46	19.16	19.96	25.22	0.191939474	0.71
CR_07660C_A	12.15	12.50	12.97	19.67	20.27	26.87	0.001584462	0.71
C3_06100C_A	18.76	13.17	17.53	24.51	30.07	32.74	0.002328262	0.71
C1_12830C_A	4.12	2.41	2.13	3.87	4.47	6.91	0.083086568	0.71
C5_03000C_A	2.13	1.75	1.91	3.52	3.05	3.70	0.000566353	0.71
C2_07350W_A	4.44	3.88	1.85	9.60	4.04	4.22	0.233835972	0.71
C3_05520C_A	9.35	10.05	9.90	15.66	18.70	17.43	0.000183465	0.71
C3_02130W_A	0.86	0.63	1.17	1.70	1.13	1.86	0.119065915	0.71
C6_03290W_A	47.90	32.53	30.58	59.64	71.86	62.91	0.005570907	0.71
C1_00400W_A	31.62	33.25	34.73	61.92	57.49	56.43	3.75E-05	0.71
C6_02980C_A	21.12	18.04	17.07	35.68	23.22	40.52	0.012773064	0.71
C3_07170C_A	13.98	12.42	11.67	21.42	20.63	25.14	2.13E-05	0.71
C5_03350W_A	43.89	45.92	59.68	108.59	76.63	78.66	0.014609743	0.71
CR_01750C_A	81.40	72.39	93.87	154.45	138.37	143.57	0.000225473	0.71
C4_07120C_A	13.24	13.71	11.14	19.55	23.99	23.62	0.000635835	0.71
C7_03210W_A	3.17	2.12	1.10	3.60	3.19	4.46	0.174242733	0.70
C3_06050C_A	4.84	4.37	6.13	14.70	6.79	5.51	0.202215925	0.70
C6_02530C_A	5.29	3.13	0.83	2.78	5.70	7.80	0.438872568	0.70
C7_00060C_A	39.22	0.79	45.59	51.24	47.78	49.20	0.698649739	0.70
CR_10230W_A	51.37	42.07	41.99	75.42	80.91	81.19	3.50E-06	0.70
C5_00920W_A	3.16	3.38	1.16	6.52	4.29	2.79	0.340541074	0.70
C2_01340W_A	10.13	11.57	13.73	23.00	17.93	21.57	0.005268941	0.70
C3_04740C_A	3.53	2.54	4.47	6.12	5.66	6.71	0.025014392	0.70
C2_01620W_A	307.20	297.71	319.31	495.35	545.08	581.08	2.12E-06	0.70
C4_02690W_A	74.76	37.08	32.65	84.75	98.45	65.57	0.106264788	0.70
C1_01380C_A	2.04	2.03	1.25	3.22	3.54	2.60	0.071216848	0.70
C5_02300C_A	25.15	26.75	21.44	40.87	43.48	44.24	0.00013438	0.70
C2_04590C_A	317.97	173.34	281.18	301.06	416.55	633.77	0.089232601	0.70
C1_13610C_A	19.28	14.49	13.45	11.03	34.15	37.27	0.209940311	0.69
C6_02220W_A	26.27	35.42	0.88	37.41	32.65	39.52	0.682728271	0.69
CR_08550W_A	50.34	61.98	67.56	110.90	100.22	104.57	0.00241548	0.69
C2_00510W_A	7.73	7.55	7.34	7.44	15.90	16.29	0.097599852	0.69
C2_08650W_A	35.54	29.37	37.69	49.12	62.50	67.63	0.003282837	0.69
CR_10660W_A	24.73	27.32	23.49	40.97	45.42	45.66	0.000158551	0.69
C2_02210C_A	5.56	3.17	2.50	6.87	4.60	8.08	0.131291839	0.69
C4_03850W_A	8.26	5.43	5.83	8.80	9.05	16.22	0.064424922	0.69
C7_03930C_A	15.34	24.87	15.62	20.80	24.89	53.43	0.186272012	0.69
C2_01500W_A	5.35	7.46	6.90	9.82	11.64	12.83	0.046064314	0.69
C4_06240W_A	7.02	5.06	4.97	7.07	9.40	13.30	0.052591813	0.69
C1_03790C_A	12.14	4.23	12.06	20.19	15.97	12.77	0.19616751	0.69
C3_01820W_A	7.84	6.96	6.42	9.17	16.44	11.09	0.040648524	0.69
C7_00560C_A	1.64	2.58	0.26	0.86	3.26	3.73	0.556641862	0.69
C4_02860W_A	110.67	109.93	124.46	203.50	206.61	189.69	8.07E-05	0.69
C2_05170W_A	40.15	40.19	37.00	86.51	50.24	67.44	0.017321474	0.69
C1_06930W_A	51.00	36.78	33.86	70.08	73.75	66.02	0.002446525	0.69
C2_05690C_A	134.25	141.95	140.90	251.08	221.88	253.24	9.54E-06	0.68
C1_04060W_A	3.84	1.97	2.36	4.76	3.74	5.65	0.073846936	0.68
C4_04260C_A	13.57	11.19	15.26	21.21	23.90	24.31	0.000943078	0.68
C1_08020W_A	10.04	7.71	6.99	15.44	13.58	13.80	0.002059904	0.68

CR_09520C_A	2.24	1.39	3.25	4.91	3.55	3.46	0.129429596	0.68
C1_01270W_A	20.86	18.25	19.23	38.63	26.38	36.28	0.002688098	0.68
C6_00820W_A	116.45	144.75	149.23	252.14	233.30	228.01	0.002151534	0.68
C1_14240W_A	26.04	25.07	24.71	52.19	41.43	37.44	0.002728776	0.68
C1_00660C_A	31.93	33.15	27.53	51.28	54.47	54.63	9.98E-05	0.68
C4_05690W_A	38.83	57.96	51.60	91.52	62.04	106.12	0.045303882	0.68
C2_00170C_A	9.49	8.93	11.36	18.49	18.35	14.56	0.008412494	0.68
C3_02940C_A	140.56	125.89	139.58	211.39	277.10	210.88	0.002370265	0.68
C4_02120W_A	13.32	14.19	14.83	23.48	22.42	27.45	0.000552971	0.68
C3_07690C_A	19.82	24.51	21.23	41.16	36.23	36.22	0.002707776	0.68
C4_02780W_A	15.46	16.87	19.69	30.98	31.94	26.96	0.003762876	0.68
C4_02490W_A	5.67	3.45	2.70	6.41	7.17	6.65	0.059826691	0.68
C2_04710C_A	5.16	4.62	6.80	12.67	8.76	7.20	0.08821001	0.68
C2_00400C_A	30.67	37.25	49.10	114.19	32.39	56.54	0.254294691	0.68
C6_02340W_A	89.45	65.02	74.38	175.73	148.13	66.15	0.154494362	0.68
C5_00740W_A	1.35	1.53	1.74	2.82	2.83	2.32	0.200554027	0.68
C3_05360C_A	23.62	18.08	16.49	32.48	30.69	36.92	0.000834164	0.67
C1_03480C_A	16.62	13.04	11.41	23.95	22.78	23.84	0.000857018	0.67
C5_01510W_A	26.58	29.48	33.99	59.28	48.69	47.39	0.009949936	0.67
C7_02040C_A	246.01	253.02	196.37	453.48	457.32	280.57	0.038924169	0.67
C2_09970C_A	16.22	25.98	24.55	33.98	38.64	43.35	0.036225048	0.67
C2_05530C_A	49.30	39.53	39.40	77.28	73.72	69.07	0.000122723	0.67
C3_01390C_A	8.91	5.28	3.76	6.34	10.72	13.66	0.170951764	0.67
CR_06540W_A	2.64	2.21	1.17	1.90	3.65	4.84	0.229651539	0.67
C2_05740W_A	8.24	6.95	7.39	11.62	12.93	14.28	0.000301877	0.67
C2_00590W_A	4.71	4.57	4.96	8.25	7.86	8.41	0.00044536	0.67
C3_00670C_A	18.83	16.80	18.57	34.77	29.79	28.35	0.000332369	0.67
C6_04250W_A	271.99	315.45	383.85	646.24	494.41	532.83	0.012476895	0.67
C4_00510C_A	4.50	3.58	4.33	6.85	5.29	9.32	0.071702613	0.67
C5_00510W_A	0.59	0.55	0.78	1.13	1.10	1.07	0.16348496	0.67
C3_06010W_A	92.42	99.23	118.88	274.35	126.19	132.33	0.129420626	0.67
C1_13480W_A	126.28	271.36	256.27	369.01	357.96	408.75	0.107501817	0.67
C3_00850C_A	112.71	99.10	101.57	164.08	168.16	204.91	1.61E-05	0.66
C7_02650W_A	0.85	0.28	0.75	0.89	1.07	1.28	0.326601272	0.66
C2_00250W_A	3655.78	2995.60	3235.81	5111.19	6573.24	5161.03	0.001684043	0.66
C1_09040C_A	0.61	0.23	0.31	0.48	0.58	0.92	0.38605055	0.66
C4_06190C_A	5.91	3.45	3.48	7.90	6.45	7.48	0.03024628	0.66
C3_07250W_A	20.90	19.40	17.35	36.83	30.89	30.83	0.000563394	0.66
C1_02320C_A	10.47	9.22	15.77	21.67	18.06	21.11	0.036430935	0.66
CR_10770W_A	16.96	14.57	10.66	26.36	27.30	18.01	0.048567238	0.66
C4_00050W_A	51.42	44.73	41.93	80.52	78.25	76.97	2.07E-05	0.66
C4_06790W_A	4.84	5.36	6.21	9.33	9.58	9.21	0.012736647	0.66
C7_01300C_A	1.75	1.27	1.54	1.49	2.18	4.19	0.2979841	0.66
C1_11100W_A	6.74	7.06	6.48	9.74	9.77	15.37	0.04923674	0.66
CR_01700C_A	3.99	3.22	3.25	7.01	5.01	5.88	0.006655492	0.66
C4_00330C_A	206.98	174.33	180.38	295.95	342.09	319.06	4.46E-05	0.66
C7_01210C_A	5.87	8.34	7.14	12.94	11.13	12.56	0.023473539	0.66
C1_03210C_A	60.09	59.04	62.31	99.50	99.95	110.51	1.56E-06	0.66
C7_03240W_A	45.58	42.02	42.65	73.35	72.32	76.47	1.14E-08	0.66
C2_05080C_A	2.44	1.83	1.97	4.39	3.40	2.89	0.089482269	0.66
C3_03090W_A	2.65	1.53	2.11	4.06	3.13	3.56	0.049245688	0.66
C2_00880W_A	5.25	5.93	5.58	9.15	8.17	11.37	0.01092946	0.66
CR_09470W_A	2.14	1.61	0.93	2.11	2.77	3.06	0.122754663	0.66
C2_01010W_A	96.31	140.45	159.52	251.13	209.04	218.31	0.036992157	0.65
C1_09760C_A	16.26	20.79	22.88	46.61	22.28	33.67	0.103612883	0.65
C6_00270W_A	11.92	10.68	20.93	28.46	22.26	23.48	0.103612883	0.65
C6_00950C_A	3.46	3.82	2.62	5.08	6.40	5.36	0.024804055	0.65
CR_05210W_A	4.58	2.37	5.01	7.40	5.50	7.41	0.117589712	0.65
C6_02450W_A	9.31	10.49	13.59	18.66	16.26	22.14	0.027572786	0.65
C4_06650W_A	23.45	19.39	17.55	37.53	29.05	35.86	0.001710625	0.65
CR_04790W_A	15.67	9.94	12.99	22.44	19.00	24.00	0.016796275	0.65
CR_04380C_A	21.64	22.50	22.57	38.94	37.46	36.90	2.03E-05	0.65
CR_08110W_A	2.63	2.11	2.82	3.24	5.12	4.37	0.067221261	0.65

C5_04490C_A	6.66	16.42	15.69	22.79	20.77	23.00	0.182084599	0.65
C4_02670W_A	5.70	2.82	3.49	6.11	6.67	7.47	0.072575663	0.65
C3_04430W_A	231.54	215.33	236.52	385.46	383.24	388.64	5.01E-07	0.65
C5_00800C_A	57.12	81.82	78.88	117.36	129.82	123.55	0.01500929	0.65
C5_01770C_A	173.62	172.95	166.94	280.33	284.70	304.62	2.53E-07	0.65
CR_02870W_A	10.98	8.43	8.62	14.13	15.63	17.58	0.000913388	0.65
CR_05740C_A	20.14	18.85	22.22	37.57	30.50	35.53	0.00083335	0.65
C4_01780C_A	7.97	3.51	4.50	7.79	8.56	10.45	0.125602808	0.65
CR_10850C_A	555.12	416.60	521.57	744.91	923.92	841.42	0.001601468	0.64
C3_06560W_A	44.75	63.60	49.79	103.28	74.74	90.64	0.023978444	0.64
C5_04610W_A	4.52	7.92	4.01	8.46	9.39	10.10	0.117692457	0.64
C4_01160W_A	3.44	1.29	5.71	6.87	4.03	6.63	0.432814008	0.64
C6_01740C_A	51.66	38.32	38.78	66.73	78.21	70.82	0.001574315	0.64
C6_02500C_A	19.37	19.33	23.08	37.64	30.93	35.72	0.001841751	0.64
C1_00880W_A	5.24	4.28	5.31	7.63	8.60	8.69	0.002480842	0.64
CR_08630W_A	5.15	5.45	9.43	5.67	14.15	14.10	0.248917841	0.64
C1_07000W_A	2.46	1.60	1.50	3.01	4.12	2.15	0.155374742	0.64
C3_06570C_A	28.28	45.73	42.54	73.76	40.55	84.54	0.138627696	0.64
C2_07800W_A	3.05	1.08	0.31	1.49	2.40	3.51	0.586482949	0.64
C7_02920W_A	2.12	0.87	2.83	5.16	1.29	3.39	0.42198408	0.64
C4_00090W_A	40.92	42.21	47.23	78.51	76.22	64.31	0.002702916	0.64
C3_02350W_A	2.98	1.15	1.40	2.13	3.25	3.86	0.245029172	0.64
CR_04860C_A	3.51	1.24	0.79	2.93	2.95	3.34	0.366716045	0.64
C6_00310W_A	32.04	21.74	19.30	38.91	41.42	41.68	0.012669123	0.64
C1_00820W_A	0.11	0.85	0.64	0.29	1.02	1.43	0.612444807	0.64
C2_05850C_A	3.05	2.10	2.36	3.68	3.99	4.92	0.019157997	0.64
CR_04910W_A	0.95	0.65	0.98	1.14	1.78	1.39	0.224035682	0.64
C1_00200C_A	4.25	4.48	4.65	6.83	9.21	6.49	0.12271199	0.64
C1_05390C_A	8.44	8.02	10.40	14.63	15.19	15.31	0.002670846	0.64
CR_07380C_A	12.34	13.45	14.31	27.35	21.02	18.89	0.023108761	0.63
CR_07840C_A	18.89	6.51	10.19	11.34	16.95	30.95	0.314357956	0.63
C1_13640W_A	18.52	15.26	13.79	26.45	26.82	26.22	0.000561937	0.63
CR_03530W_A	91.21	96.13	91.18	165.33	146.81	154.90	4.24E-05	0.63
C3_01310W_A	0.50	0.30	0.18	0.67	0.42	0.55	0.345587752	0.63
C4_01960C_A	21.18	19.27	17.90	43.25	25.65	28.67	0.039920247	0.63
C4_00100C_A	38.11	40.23	31.56	70.36	68.81	44.05	0.049121106	0.63
C2_08780W_A	68.99	72.02	67.15	113.05	110.74	125.31	1.50E-05	0.63
C4_07030W_A	10.32	11.94	16.08	24.25	18.81	21.33	0.047220681	0.63
C5_03920C_A	5.68	3.68	2.73	4.71	7.02	8.48	0.197197638	0.63
CR_04300W_A	17.84	14.73	17.23	27.61	27.39	28.23	2.39E-05	0.63
C1_11890W_A	7.23	4.92	4.82	8.99	9.16	10.19	0.019921153	0.63
CR_09780C_A	0.57	0.42	0.08	0.89	0.48	0.41	0.532688536	0.63
C5_00760W_A	10.09	9.00	8.89	15.00	15.15	16.63	3.89E-05	0.63
C3_04020C_A	4.23	3.25	2.61	4.67	6.24	5.91	0.033290132	0.63
CR_08600C_A	29.08	51.12	60.21	47.33	92.83	97.39	0.208174029	0.63
C5_05110C_A	5.90	6.28	7.48	9.52	10.60	12.87	0.010583928	0.63
CR_09460C_A	38.31	36.98	24.87	46.00	49.35	72.60	0.063047831	0.63
C4_03800C_A	3.57	2.53	2.21	3.17	5.11	5.59	0.097531197	0.63
C4_01700C_A	683.40	893.76	1077.86	1443.35	1358.45	1667.45	0.027228403	0.63
CR_06080W_A	120.46	106.67	106.04	171.98	182.43	201.93	5.31E-06	0.63
C6_04330W_A	0.24	0.37	0.30	0.56	0.28	0.71	0.382740329	0.63
C2_02090W_A	4.75	4.67	7.43	7.78	9.72	10.68	0.07557439	0.63
C7_00330C_A	1.61	1.68	1.52	2.91	2.25	2.91	0.071192646	0.63
C7_03990C_A	10.76	19.04	11.63	17.60	22.14	30.12	0.140025158	0.63
C2_08840W_A	3.82	2.48	3.79	1.79	8.52	6.62	0.443535214	0.63
C3_05650W_A	26.09	74.73	79.76	115.79	91.01	98.83	0.259277398	0.63
C2_00740C_A	28.43	33.12	32.43	55.69	41.52	60.40	0.009981511	0.63
C2_01840C_A	7.00	6.12	5.04	9.90	10.98	9.29	0.008851555	0.62
C1_07970C_A	12.47	11.87	8.30	17.17	16.05	21.22	0.016817746	0.62
C6_03220W_A	1.62	1.16	1.18	1.14	2.47	3.03	0.293405896	0.62
C1_05640C_A	2.66	3.45	3.29	6.20	3.92	5.58	0.082612467	0.62
C3_02810C_A	5.03	4.81	6.40	8.84	8.82	9.35	0.006911216	0.62
C2_03460C_A	28.17	24.74	27.05	43.40	44.74	44.61	1.23E-05	0.62

C1_10670C_A	0.93	0.66	0.94	2.01	0.89	1.30	0.18261215	0.62
C3_01450C_A	1.80	0.43	0.75	1.04	1.90	1.95	0.389671193	0.62
C2_07470W_A	10.77	10.48	9.35	15.00	17.63	18.19	0.000515592	0.62
C2_04740C_A	2.30	1.01	0.72	2.42	2.10	2.16	0.352904908	0.62
C1_01870C_A	36.22	36.10	58.45	66.59	77.35	73.59	0.051332171	0.62
C2_08290C_A	880.65	613.03	771.80	1232.54	1111.25	1405.60	0.002404192	0.62
C2_03210W_A	4.71	3.79	5.10	6.17	4.17	12.21	0.357434906	0.62
C2_04870C_A	23.57	14.43	13.32	27.36	29.28	27.63	0.034348335	0.62
C6_01460C_A	7.27	11.05	7.53	15.84	5.98	21.76	0.30841	0.62
C1_09630W_A	192.62	221.68	234.41	387.26	347.09	342.36	0.002608156	0.62
CR_03150W_A	15.78	6.40	8.96	14.61	16.08	20.34	0.140025158	0.62
C7_03010W_A	13.57	11.61	6.58	17.89	15.77	18.76	0.080501782	0.62
C1_01830C_A	161.02	181.13	189.63	287.55	286.69	307.73	0.000594935	0.61
C4_03270W_A	11.57	9.90	11.71	27.15	12.93	14.66	0.144500804	0.61
C6_02790C_A	14.53	18.77	17.93	24.81	33.10	26.88	0.025050708	0.61
C1_00140W_A	41.35	29.88	27.98	49.48	55.76	57.84	0.005422601	0.61
C4_05750C_A	8.32	8.26	0.42	11.58	7.44	9.03	0.586407641	0.61
CR_01310W_A	68.23	99.29	118.48	188.87	141.72	144.53	0.082153152	0.61
C2_02450C_A	2.09	0.76	1.33	2.44	2.35	2.02	0.200056739	0.61
C2_07100W_A	32.16	36.06	40.12	51.06	66.34	61.66	0.010249133	0.61
CR_01600C_A	0.19	0.30	0.15	0.40	0.41	0.23	0.411894076	0.61
C5_03080C_A	2.47	3.07	2.53	4.20	4.79	4.33	0.025014392	0.61
C5_00210C_A	27.44	32.55	39.25	52.56	55.45	56.16	0.013255916	0.61
C1_07590C_A	5.20	2.99	3.07	5.65	5.87	6.94	0.053984943	0.61
C3_04860W_A	78.81	37.60	40.78	66.77	106.14	82.06	0.146285864	0.61
C2_10350C_A	49.16	52.91	60.81	85.39	88.02	95.38	0.001540159	0.61
C6_03820C_A	22.37	16.23	18.62	26.24	33.53	33.94	0.008833996	0.61
C2_07630C_A	70.24	129.61	99.78	184.91	148.18	164.20	0.093882464	0.61
C3_02240C_A	23.61	14.53	16.63	29.86	31.82	27.66	0.021133975	0.61
C1_11380W_A	5.93	3.99	6.33	8.80	9.83	7.99	0.067067722	0.60
C1_10090C_A	49.32	41.83	36.17	65.18	69.26	74.10	0.000946529	0.60
C2_04640C_A	2.06	1.25	1.41	2.01	2.60	3.13	0.08794357	0.60
C2_09370C_A	2.40	1.77	1.81	3.00	2.90	3.92	0.038061007	0.60
CR_09710W_A	6.73	5.78	6.64	12.34	8.21	10.93	0.025643694	0.60
CR_10740W_A	19.53	21.31	17.23	33.38	30.69	31.16	0.003704078	0.60
CR_08100C_A	5.88	4.29	5.71	5.67	12.29	7.87	0.169200175	0.60
C3_03180C_A	66.92	53.32	60.91	95.90	97.94	102.07	5.13E-05	0.60
CR_02720C_A	8.82	5.82	7.57	9.06	11.60	15.66	0.084087813	0.60
C3_07030C_A	3.55	3.90	2.96	5.85	4.85	6.40	0.01834705	0.60
C1_00360W_A	5.02	9.42	5.10	9.29	9.43	13.58	0.178929734	0.60
CR_01390W_A	65.46	52.01	64.76	91.01	90.22	116.69	0.003306929	0.60
C7_04310C_A	52.86	29.31	30.79	54.07	67.58	61.04	0.064272208	0.60
C3_07090W_A	194.76	277.51	187.71	323.86	365.47	395.35	0.036225048	0.60
C7_02100W_A	64.49	58.61	1.89	76.88	77.66	46.59	0.731634503	0.59
C3_00200C_A	97.36	53.03	94.65	173.17	29.12	199.65	0.462254833	0.59
C4_03130W_A	16.91	16.25	12.99	24.46	22.93	27.96	0.0031318	0.59
CR_03160W_A	2.68	0.98	3.98	3.19	3.56	5.69	0.408994479	0.59
C3_02530W_A	17.58	9.77	12.25	11.69	18.30	34.52	0.327136612	0.59
C5_03980W_A	6.88	5.58	5.39	8.18	11.04	9.76	0.013431194	0.59
C2_01710W_A	9.52	9.61	11.29	22.33	8.78	18.69	0.238991131	0.59
C1_04730C_A	2.84	11.63	15.76	21.65	8.17	20.58	0.483887559	0.59
C2_08130W_A	20.89	23.86	24.04	32.63	35.13	44.83	0.011620745	0.59
C3_03730C_A	52.50	43.99	47.16	72.01	87.77	73.19	0.002554864	0.59
C5_00060C_A	10.31	8.46	4.43	7.65	16.27	13.65	0.256395375	0.59
C6_04610C_A	5.75	4.33	3.98	6.82	7.57	8.48	0.01898692	0.59
C4_02800W_A	1.96	1.83	1.73	2.63	2.78	3.60	0.063835893	0.59
C5_05350W_A	11.41	20.62	15.31	22.72	22.31	32.97	0.12699066	0.59
C3_06950W_A	1.40	0.86	1.15	1.42	2.22	1.87	0.12282342	0.59
C7_00470C_A	3.15	0.00	2.93	2.17	3.94	3.59	0.820144207	0.59
C2_08200W_A	3.57	3.35	2.21	3.14	5.30	6.42	0.221359633	0.59
CR_09200C_A	4.75	5.71	0.66	4.15	6.98	6.97	0.522500465	0.59
C4_06720W_A	26.58	19.16	25.81	44.08	37.26	34.34	0.018543025	0.59
C1_08380W_A	2587.16	2288.83	2373.64	3551.67	3832.85	4386.51	6.35E-05	0.59

CR_09770C_A	3.60	1.70	0.40	3.67	2.48	2.94	0.513175328	0.59
CR_02140W_A	69.19	65.54	68.82	120.85	107.31	101.85	0.000724574	0.59
CR_07650W_A	51.76	48.16	51.80	78.05	81.83	86.26	1.74E-05	0.59
C2_10280C_A	53.66	54.02	55.41	94.49	83.14	86.89	0.00014018	0.58
C4_01130C_A	7.19	4.87	4.50	6.83	9.73	10.25	0.111910776	0.58
C2_08640C_A	23.12	28.88	26.78	40.84	45.23	41.69	0.01221646	0.58
C6_00860W_A	2.95	3.67	4.66	6.30	5.58	6.38	0.119320905	0.58
C3_07080W_A	12.05	17.25	10.14	20.19	21.42	22.56	0.071989199	0.58
CR_06960W_A	3.14	0.98	1.21	1.46	2.62	4.49	0.448488558	0.58
CR_02090C_A	80.90	61.16	60.84	104.11	106.21	116.54	0.001101152	0.58
CR_01120C_A	42.84	27.92	17.84	19.61	62.22	60.24	0.365865577	0.58
C1_03560C_A	75.20	35.40	82.45	109.25	101.66	98.66	0.151865616	0.58
C1_04950C_A	7.74	5.01	4.17	7.84	9.11	10.22	0.072332682	0.58
C4_00670W_A	6.78	6.40	5.69	9.74	10.11	10.59	0.000914609	0.58
CR_07040W_A	7.39	4.48	7.14	9.48	10.75	10.33	0.051630862	0.58
CR_02530W_A	6.20	6.72	7.97	10.59	11.28	11.90	0.012281061	0.58
C2_00970C_A	34.24	28.38	29.69	53.95	47.55	47.07	0.000529504	0.58
C1_06740C_A	7.91	3.03	4.79	8.54	8.97	7.73	0.230844452	0.58
CR_09650W_A	34.65	33.59	30.54	59.48	50.99	48.67	0.002193161	0.58
C3_04440C_A	0.30	0.37	0.37	0.60	0.51	0.56	0.348525217	0.58
C4_05210W_A	5.16	3.63	3.69	6.92	7.25	5.89	0.084427402	0.58
C3_02030W_A	4.15	8.26	7.20	11.13	8.89	11.93	0.181707397	0.58
C4_02440C_A	47.74	47.54	40.56	68.01	70.33	80.57	0.000740646	0.57
C6_04350C_A	30.69	23.66	16.30	43.02	34.60	35.41	0.073846936	0.57
C1_04860W_A	11.13	11.43	11.05	19.15	15.92	19.12	0.000960789	0.57
C1_05380C_A	1.93	2.15	1.88	3.08	3.19	3.32	0.004468973	0.57
C5_05170W_A	10.88	8.36	9.43	16.85	15.97	13.07	0.014225949	0.57
C3_01420C_A	21.02	17.26	14.72	24.06	22.03	39.27	0.114407554	0.57
C1_00060W_A	238.96	171.12	190.13	268.29	333.46	359.54	0.009870124	0.57
C1_03140W_A	26.33	19.94	22.15	31.26	37.77	40.83	0.01189812	0.57
C1_10140C_A	10.29	8.27	6.77	14.14	11.01	15.53	0.033744469	0.57
C6_02120W_A	21.72	23.06	22.42	33.96	29.91	44.63	0.011807071	0.57
C1_00540C_A	0.28	2.79	1.70	2.20	2.50	3.17	0.559724186	0.57
CR_04710W_A	9.55	5.84	3.65	6.69	11.91	11.84	0.321304109	0.57
C4_04510W_A	7.34	5.85	5.56	9.66	9.36	11.04	0.002278254	0.57
C1_11220C_A	23.21	19.21	19.85	37.56	29.51	32.67	0.003183772	0.57
C3_07280C_A	52.89	51.27	43.96	69.67	79.23	88.75	0.002866972	0.57
C2_00470W_A	3.13	4.09	4.31	7.56	3.58	7.51	0.190732774	0.57
CR_01190C_A	5.90	1.65	2.25	3.07	5.87	6.61	0.50035146	0.57
C2_05760C_A	133.69	123.85	144.47	214.48	204.31	223.85	0.000285852	0.56
C2_01480W_A	57.91	52.19	54.02	87.66	84.58	89.74	8.68E-07	0.56
C5_00250C_A	7.24	5.08	6.84	10.20	9.73	10.62	0.040233866	0.56
CR_02680W_A	1.42	0.80	1.04	1.62	1.52	2.03	0.139748485	0.56
C3_03600C_A	3.01	1.14	4.09	5.35	2.98	4.84	0.418341416	0.56
C5_03820C_A	13.28	10.41	10.35	18.41	17.28	18.49	0.001301671	0.56
C1_10790W_A	151.74	193.19	177.88	289.94	268.55	279.00	0.008162311	0.56
CR_05460W_A	21.41	22.39	14.40	19.26	26.23	47.87	0.243362554	0.56
C5_03360W_A	25.62	21.61	31.31	42.45	40.65	41.79	0.022229783	0.56
C2_10260C_A	19.51	19.70	19.03	27.07	30.26	35.46	0.003140838	0.56
C4_06590W_A	2.47	1.83	1.15	2.67	2.24	3.84	0.300782336	0.56
C2_10290W_A	183.49	184.32	187.11	283.39	309.27	289.03	0.000256225	0.56
CR_01660C_A	29.32	26.79	26.16	44.59	40.90	45.21	0.000170576	0.56
C2_10650W_A	22.81	23.98	20.36	28.34	37.69	40.82	0.065951906	0.56
C1_09650W_A	58.09	53.57	54.36	88.46	81.80	93.42	1.19E-05	0.55
C6_02200C_A	13.78	13.60	11.66	15.95	15.29	31.08	0.192024916	0.55
C2_07000W_A	16.63	15.52	17.05	27.81	22.43	27.88	0.006101773	0.55
C5_00340W_A	29.54	13.49	19.74	37.60	29.61	31.33	0.138823792	0.55
C2_06490W_A	17.27	23.11	26.53	40.65	27.85	38.25	0.094330334	0.55
C5_00480C_A	22.40	20.00	22.53	35.46	34.28	33.01	0.00106284	0.55
C4_06200W_A	2.55	10.44	7.96	18.44	9.12	6.21	0.556641862	0.55
CR_02930W_A	10.05	7.44	7.80	13.78	11.49	14.67	0.006970037	0.55
C1_07540C_A	62.37	45.52	50.54	72.30	91.47	85.66	0.009694197	0.55
C1_13580W_A	9.29	6.81	7.63	11.05	13.66	12.68	0.012688955	0.55

C1_10530W_A	19.30	4.55	26.86	41.05	7.99	31.03	0.574500283	0.55
C2_03770C_A	13.63	14.71	13.25	24.50	19.58	21.74	0.005604199	0.55
C3_01300C_A	0.59	0.56	1.09	1.53	0.74	1.27	0.469883906	0.55
C1_04160C_A	1.54	0.74	0.75	0.71	1.95	2.10	0.493283063	0.55
C5_03130W_A	5.10	3.50	6.64	9.31	6.88	7.84	0.155071301	0.55
C1_03640C_A	11.63	0.00	7.87	8.49	11.10	10.39	0.839384464	0.55
C4_03350C_A	10.39	10.91	10.83	16.92	16.17	17.63	0.001710625	0.55
C4_01490W_A	4.36	2.90	1.73	5.04	3.81	5.24	0.225619225	0.55
C1_11440C_A	1.38	1.01	1.19	1.51	2.26	1.84	0.096098634	0.55
CR_10160W_A	43.71	41.88	50.98	86.84	70.27	57.91	0.049121106	0.55
C1_13270W_A	19.18	8.96	11.36	15.24	22.89	23.60	0.19616751	0.55
C1_01300W_A	32.31	28.53	27.76	40.87	39.11	60.10	0.032081675	0.54
C6_02180W_A	19.53	14.27	17.68	24.28	25.60	31.08	0.021133975	0.54
C3_00280C_A	10.24	6.82	7.17	8.59	10.04	19.68	0.220889662	0.54
C3_03680W_A	15.28	16.83	14.32	23.32	23.24	26.80	0.003404625	0.54
C4_05610C_A	64.33	46.13	37.93	97.48	54.86	80.46	0.128395943	0.54
C2_06250C_A	3.40	3.52	3.50	6.22	6.07	4.11	0.090465811	0.54
CR_07620W_A	45.85	56.44	62.90	98.04	78.96	84.10	0.037049092	0.54
C3_03910W_A	756.00	929.34	1026.91	1418.09	1536.67	1325.31	0.030262074	0.54
C5_00680W_A	4.57	2.78	2.28	6.15	3.58	5.41	0.231276264	0.54
C7_01410C_A	13.28	9.82	10.27	22.50	2.43	28.11	0.572518646	0.54
C4_06600W_A	8.18	6.99	7.93	12.95	11.11	12.24	0.000876925	0.54
C6_03300C_A	11.14	9.32	10.32	14.31	18.03	15.89	0.008433485	0.54
CR_01350C_A	50.12	34.36	39.14	83.52	46.28	63.95	0.105931893	0.54
C1_13060C_A	7.36	8.52	8.45	14.60	12.41	11.29	0.029388937	0.54
C2_02380W_A	57.25	57.23	57.45	89.70	89.07	91.29	4.89E-05	0.54
CR_06710C_A	18.21	13.69	16.35	23.96	24.44	27.18	0.003275514	0.54
CR_07670W_A	2.25	2.84	1.85	3.23	3.04	4.63	0.222464328	0.54
C3_07110W_A	2.06	1.72	1.19	2.87	2.24	2.67	0.102033301	0.54
CR_04080C_A	22.28	26.58	25.20	36.83	38.52	41.12	0.006171999	0.54
CR_08720W_A	1.81	0.37	1.62	1.58	1.78	2.56	0.485138775	0.54
C2_05930W_A	174.95	178.91	194.37	276.16	276.96	306.85	0.001286653	0.54
C1_14440C_A	83.42	62.11	158.96	194.14	229.61	47.37	0.50811641	0.53
C2_04470W_A	1.71	2.81	4.01	4.45	2.70	6.36	0.40072849	0.53
C4_05220C_A	1.69	1.58	0.82	1.68	2.40	2.31	0.247778987	0.53
C5_05310W_A	106.90	47.56	127.01	143.78	155.58	137.40	0.245076807	0.53
C2_01220W_A	8.69	6.79	6.32	10.17	11.91	11.91	0.013243787	0.53
C7_03250C_A	126.85	147.02	144.84	260.86	188.60	207.09	0.029627	0.53
C6_02850W_A	6.73	5.12	6.22	8.25	7.73	12.39	0.094187006	0.53
CR_03210C_A	19.34	16.32	14.41	24.25	25.62	28.38	0.011722541	0.53
C4_05550C_A	22.73	23.71	24.18	38.75	32.72	38.97	0.00364683	0.53
C2_00950C_A	8.50	10.62	10.53	14.10	16.96	15.29	0.044762414	0.53
C2_10810W_A	16.58	0.65	16.54	15.93	15.23	20.80	0.65483529	0.53
C1_12670C_A	0.64	0.54	0.32	0.92	0.53	0.93	0.343810032	0.53
C3_01910C_A	130.48	64.71	96.56	133.53	158.04	160.06	0.11954924	0.53
C3_03890W_A	17.45	15.64	16.05	30.42	25.79	20.13	0.043939442	0.53
C3_06500W_A	52.47	49.75	45.80	93.78	68.39	68.19	0.026741894	0.53
C2_07670C_A	16.56	10.32	10.83	13.22	27.26	17.65	0.225147477	0.53
C2_07540W_A	49.39	37.99	53.17	71.18	70.13	77.50	0.016973662	0.53
C5_03020W_A	2.38	0.39	1.34	1.96	2.13	2.19	0.482827304	0.53
C7_00120W_A	76.68	0.36	84.46	84.68	80.78	81.87	0.814462924	0.53
C7_04190C_A	4.97	3.12	3.43	1.91	8.38	7.51	0.460091464	0.53
C4_03460C_A	5.69	4.29	5.26	9.06	7.71	6.91	0.041937023	0.53
C3_01230C_A	1.90	1.61	1.47	2.02	2.27	3.47	0.308357204	0.53
C4_06870W_A	118.53	119.48	116.21	183.32	188.78	179.44	0.000274424	0.53
C4_00150C_A	108.41	75.57	87.39	141.87	149.03	128.83	0.014049205	0.53
C1_03410W_A	2.38	1.32	2.01	3.46	1.67	3.81	0.367734576	0.53
C3_01700W_A	7.31	6.06	4.62	7.91	8.41	11.70	0.089815052	0.53
C1_01010W_A	101.35	89.86	108.73	147.77	166.75	151.29	0.004591365	0.53
C4_06800W_A	40.75	22.41	28.88	47.76	46.42	47.83	0.064191249	0.53
C6_03890C_A	14.17	13.87	14.02	22.48	19.29	23.75	0.003316348	0.53
C4_01820C_A	30.66	42.62	32.49	81.24	58.24	24.43	0.37311625	0.52
C4_03740W_A	4.89	6.37	5.70	11.20	5.54	9.79	0.220808341	0.52

C3_06940W_A	21.22	20.05	23.65	17.01	34.79	49.76	0.281865627	0.52
C1_12760W_A	1.29	0.35	0.83	1.16	1.54	1.16	0.491753936	0.52
C2_04140W_A	86.05	66.00	66.18	100.81	113.34	123.72	0.005914491	0.52
C7_02050C_A	11.88	10.96	8.43	16.36	18.98	12.93	0.08764157	0.52
C3_02550C_A	1.53	1.54	1.45	1.47	2.78	2.75	0.348886157	0.52
C4_01060W_A	13.31	8.36	9.01	18.73	14.49	13.99	0.076294097	0.52
C1_08220W_A	22.59	18.82	19.33	31.47	30.14	32.42	0.000214085	0.52
CR_02150W_A	9.78	13.08	11.02	20.87	15.21	16.66	0.071690072	0.52
C2_05970C_A	1.58	1.27	1.75	2.34	2.57	2.23	0.218459827	0.52
C1_06080C_A	11.93	4.50	4.27	5.81	9.98	16.04	0.447851843	0.52
C1_14340C_A	47.74	38.38	36.65	67.40	77.59	43.39	0.134996571	0.52
C4_04910C_A	3.07	4.13	7.11	9.31	7.01	5.83	0.346992055	0.52
C4_07050W_A	13.88	16.84	15.33	18.87	24.79	27.77	0.064706092	0.52
C2_08950W_A	38.71	9.56	17.53	57.36	8.46	34.47	0.587858511	0.52
CR_05540C_A	18.21	31.09	14.73	27.16	35.25	37.42	0.234386307	0.52
C7_03960C_A	25.59	49.06	25.15	41.38	43.07	72.22	0.288155896	0.52
C2_09230C_A	78.66	69.96	69.31	119.92	114.65	101.05	0.003806655	0.52
C6_02630C_A	10.72	13.02	8.04	21.51	14.83	12.72	0.186382741	0.52
C1_09870W_A	0.15	0.46	0.94	0.96	0.92	0.55	0.61384795	0.51
CR_05480W_A	51.59	80.73	59.13	64.70	46.00	191.71	0.45741864	0.51
C2_01580W_A	5.07	4.02	5.41	6.16	7.10	9.11	0.123897931	0.51
C7_01470C_A	42.32	55.94	0.63	51.87	48.26	52.37	0.796581984	0.51
C2_05670C_A	0.92	2.11	1.54	2.57	2.08	2.39	0.399568567	0.51
C2_06880C_A	66.20	72.42	71.40	161.26	63.24	100.24	0.253114487	0.51
C4_01980C_A	160.71	84.75	198.78	283.93	186.40	210.81	0.249458324	0.51
C4_02370C_A	6005.17	4087.90	5207.68	6871.24	8967.79	7570.36	0.039245693	0.51
C7_03500W_A	4.27	4.87	4.77	3.29	9.84	8.31	0.365534884	0.51
C4_05330C_A	1.08	0.82	0.72	1.43	1.68	0.92	0.350043963	0.51
C2_10050W_A	9.37	14.41	12.86	9.95	23.69	23.23	0.307491389	0.51
C1_01440C_A	2.36	1.93	0.94	3.55	1.80	2.74	0.496903117	0.51
C2_08170W_A	0.54	0.48	0.80	0.54	1.12	1.14	0.457356894	0.51
C1_04380W_A	28.58	28.25	29.18	47.80	36.14	48.64	0.015312964	0.51
C2_03140C_A	16.37	19.11	18.16	28.22	25.58	28.95	0.00801317	0.51
CR_03900W_A	29.15	21.57	14.99	26.01	45.71	28.21	0.243238763	0.51
C4_03700W_A	6.10	21.50	11.93	29.59	14.47	17.67	0.45741864	0.51
C3_06230W_A	4.55	3.92	4.04	6.84	6.23	6.14	0.0057928	0.51
C1_04300C_A	44.46	48.83	47.20	85.45	68.22	62.13	0.037212188	0.51
C7_01460C_A	1.75	0.64	0.00	0.97	1.14	1.47	0.844125341	0.51
CR_01680C_A	19.30	14.07	10.37	16.66	25.89	24.20	0.168576573	0.51
CR_06940W_A	4.58	2.50	2.17	3.41	5.47	5.19	0.274252493	0.51
C5_05400W_A	21.55	17.11	17.12	20.40	40.41	24.13	0.203185637	0.51
C1_08580C_A	9.65	14.02	14.58	21.06	14.80	23.42	0.158362069	0.51
C1_01780C_A	8.67	8.19	9.74	14.61	12.21	14.05	0.012290002	0.51
C2_07380W_A	59.39	64.58	25.37	127.15	61.74	38.59	0.465374117	0.51
C1_07560W_A	8.04	7.01	7.00	10.62	11.57	11.68	0.013229144	0.51
C3_03010C_A	78.83	68.09	75.76	116.05	118.80	105.79	0.003525377	0.51
C4_00970C_A	2.08	0.99	1.06	2.14	1.84	2.29	0.196825941	0.51
C3_06550C_A	12.64	10.54	11.65	16.49	16.53	20.41	0.01151612	0.50
C2_01900C_A	12.79	8.64	4.64	12.19	12.63	14.87	0.281617417	0.50
C2_10080W_A	7.35	6.20	8.21	12.29	11.02	10.00	0.049449259	0.50
C2_05150W_A	10.78	13.06	14.76	18.35	23.04	17.74	0.093703209	0.50
C4_03600C_A	35.32	22.26	25.99	39.11	39.17	49.15	0.049530313	0.50
C2_01190C_A	4.09	3.95	5.98	5.69	7.96	7.80	0.186966136	0.50
CR_05550C_A	4.49	3.92	4.11	4.96	6.79	7.38	0.048694443	0.50
C5_04510W_A	1.71	4.14	3.67	5.18	5.01	4.56	0.313354192	0.50
C6_02490C_A	23.72	16.95	19.11	31.10	29.27	30.91	0.014678299	0.50
C4_02870C_A	34.34	33.90	53.34	72.40	61.18	52.30	0.148056025	0.50
C3_03590W_A	34.11	38.37	41.28	57.56	54.53	62.21	0.012782239	0.50
C1_01250W_A	17.08	11.92	26.42	36.02	19.90	28.81	0.289497307	0.50
C3_03150W_A	11.98	8.25	8.16	13.58	14.66	14.89	0.03322613	0.50
C1_12320C_A	164.25	149.82	146.10	219.90	244.09	237.83	0.001380576	0.50
C1_00020C_A	149.70	130.25	156.19	213.07	232.29	219.17	0.003865513	0.50
CR_09760W_A	11.05	5.42	1.44	9.86	8.68	8.46	0.564827412	0.50

C6_02260C_A	18.75	16.42	12.94	22.83	23.63	26.86	0.024641446	0.50
C7_04270C_A	124.19	50.67	48.27	104.54	112.07	118.32	0.300181262	0.50
C2_00420W_A	23.75	21.12	21.36	53.04	6.06	42.67	0.572780735	0.50
CR_01240C_A	12.37	14.41	9.09	18.19	18.19	18.37	0.071774306	0.50
CR_03820C_A	6.41	4.62	5.48	9.33	7.86	7.89	0.046243515	0.50
CR_06040W_A	37.61	41.77	30.04	56.79	54.99	55.14	0.027927803	0.50
C3_06770W_A	2.30	2.64	1.78	4.54	2.77	2.91	0.280055047	0.50
C1_12710C_A	14.98	10.01	12.13	15.47	18.74	22.24	0.068499438	0.50
C3_01410C_A	16.01	8.07	7.33	12.80	17.08	17.53	0.262789284	0.50
C1_03080C_A	13.05	11.28	16.19	21.44	19.77	20.51	0.059827476	0.50
C3_02490C_A	10.44	7.20	7.62	12.59	12.07	13.66	0.021143278	0.50
C1_03360W_A	16.02	11.13	11.03	20.11	14.98	22.94	0.080873401	0.49
C1_00380C_A	7.47	11.21	7.62	11.65	12.40	16.33	0.143113888	0.49
C2_07130C_A	20.19	27.54	27.35	36.34	39.11	39.40	0.059004275	0.49
C1_01000C_A	13.95	10.61	12.05	17.76	17.32	20.61	0.013064062	0.49
C2_04950C_A	172.37	160.04	218.62	312.94	254.84	271.07	0.042468717	0.49
C1_06730W_A	23.03	25.54	35.51	39.34	44.21	44.76	0.099743789	0.49
C1_10490W_A	45.84	41.79	35.86	95.53	31.13	61.30	0.337865433	0.49
CR_01430W_A	10.15	12.46	10.21	18.28	14.75	16.94	0.06882157	0.49
C3_06820C_A	58.21	49.01	41.15	74.96	77.43	72.12	0.017993977	0.49
C3_05040C_A	4.93	4.69	4.16	6.70	5.68	8.62	0.055482028	0.49
C1_11790W_A	3.72	2.71	3.49	5.72	5.12	4.28	0.186939417	0.49
C1_04430C_A	20.23	27.88	32.19	36.27	45.70	40.40	0.13728507	0.49
C4_00400W_A	35.43	27.86	28.97	31.19	60.30	47.85	0.167217054	0.49
C2_01600C_A	54.13	50.99	50.43	82.61	75.44	77.94	0.000370462	0.49
C4_05180C_A	4.72	3.92	4.50	7.74	5.96	6.22	0.028826879	0.49
C1_04680W_A	306.40	492.55	481.57	771.43	516.95	673.16	0.175189153	0.49
CR_07400C_A	363.73	379.11	364.06	547.62	559.62	573.85	0.000466966	0.49
C3_07680W_A	7.27	7.53	6.06	11.51	10.01	10.12	0.018531396	0.49
C1_01820C_A	10.88	9.95	11.04	17.17	15.70	15.44	0.00969224	0.49
C4_02310W_A	4.79	5.25	4.72	7.87	7.46	7.06	0.015219736	0.49
C2_06750C_A	3.58	2.19	1.73	4.24	3.33	3.85	0.307202961	0.49
C4_06710W_A	13.72	13.95	13.53	21.18	21.18	20.04	0.006531889	0.49
C4_01950W_A	39.73	40.70	42.78	76.46	50.36	60.23	0.062452443	0.49
C2_00830C_A	18.79	15.96	14.41	27.26	22.47	24.59	0.014930492	0.49
CR_09040W_A	0.21	8.00	2.32	2.84	5.02	8.63	0.73713515	0.49
C1_01810C_A	23.05	25.51	23.67	35.92	33.89	39.81	0.006191737	0.49
C1_02860C_A	52.14	22.78	36.01	84.18	38.05	43.69	0.370302713	0.49
C5_02220C_A	17.44	13.08	17.65	22.95	23.67	26.09	0.036803699	0.49
CR_04350C_A	14.91	25.03	16.97	24.83	29.18	32.76	0.171640938	0.48
C2_01320W_A	6.41	1.20	2.53	2.42	6.05	6.56	0.580131445	0.48
C4_00910C_A	3.15	4.50	4.85	8.28	4.76	5.93	0.249446993	0.48
C5_01690C_A	7.25	8.41	9.32	13.73	12.14	11.95	0.054709034	0.48
C5_01710C_A	0.41	0.24	0.19	0.40	0.56	0.32	0.520544538	0.48
C2_03470C_A	102.10	88.31	95.67	133.77	132.71	165.88	0.005023912	0.48
C5_04150C_A	8.16	7.86	6.10	12.25	8.71	12.44	0.1253903	0.48
C2_03250W_A	7.20	6.79	7.24	9.49	10.31	12.29	0.025414904	0.48
CR_04130C_A	13.29	30.82	29.52	35.60	36.04	40.93	0.310795908	0.48
C4_02920W_A	20.92	16.65	17.73	10.50	35.08	37.84	0.428156912	0.48
C6_02960W_A	2.68	5.10	3.89	6.00	5.03	6.75	0.267022971	0.48
CR_04950W_A	3.79	2.05	1.98	2.72	4.04	4.97	0.290230307	0.48
C6_02770W_A	4.80	2.08	1.60	4.24	3.98	4.49	0.441069585	0.48
C2_04290W_A	15.28	8.76	8.25	17.09	9.69	21.75	0.318506591	0.48
C4_02840C_A	8.16	9.01	11.19	13.80	14.14	14.78	0.086296966	0.48
C5_01280C_A	6.31	4.80	6.85	9.18	7.04	10.86	0.122166879	0.48
C4_03820C_A	2.10	0.95	1.28	1.31	2.67	2.48	0.432372938	0.48
C5_01910W_A	246.43	254.07	228.07	498.05	290.42	306.55	0.152958615	0.48
C4_06770W_A	13.40	2.67	7.12	6.71	13.64	13.88	0.526696391	0.48
C1_13840W_A	22.37	25.70	25.00	41.41	33.72	34.93	0.030100856	0.48
C3_06530W_A	4.22	2.84	3.03	3.20	6.60	5.27	0.318350998	0.48
C4_04280C_A	12.46	13.59	7.00	13.82	21.97	13.55	0.31269312	0.48
C2_02150C_A	3.93	3.43	3.09	5.40	4.83	5.47	0.025072266	0.48
CR_05140W_A	1.70	1.60	1.18	1.94	2.41	2.40	0.325993315	0.48

C1_12230W_A	32.05	26.52	28.01	31.83	61.26	35.92	0.217029437	0.47
C3_06350W_A	21.53	18.93	17.76	22.99	31.70	32.65	0.062754759	0.47
CR_00520C_A	12.43	10.26	9.01	3.50	20.66	23.50	0.562060084	0.47
C2_03300W_A	54.44	59.48	69.49	88.61	72.12	116.52	0.103612883	0.47
C2_09990C_A	0.86	0.87	1.00	1.08	1.45	1.59	0.14424368	0.47
C5_04890C_A	2.92	1.00	2.06	3.19	2.32	3.40	0.415538079	0.47
C2_08940C_A	19.34	3.01	4.75	20.92	4.44	14.32	0.656094998	0.47
C2_10480W_A	45.15	52.98	73.44	98.53	78.91	80.61	0.149848103	0.47
C5_03240W_A	54.16	44.82	18.55	55.79	59.66	59.53	0.342802296	0.47
C1_01450W_A	38.69	38.45	45.10	76.49	40.67	66.75	0.169200053	0.47
C1_03520W_A	51.25	59.48	69.91	107.93	78.06	85.41	0.099130348	0.47
C1_01610C_A	4.38	4.06	5.00	5.62	6.55	7.94	0.126873675	0.47
C1_08510W_A	34.38	29.34	29.40	43.45	48.04	47.59	0.005849578	0.47
C3_00060W_A	15.37	11.40	9.11	14.71	18.22	20.51	0.171275835	0.47
C1_01320W_A	25.91	29.50	30.55	51.75	35.55	41.43	0.081624148	0.47
C2_05940C_A	5.58	3.85	3.83	6.55	5.80	7.42	0.084721265	0.47
C4_03320W_A	12.45	11.30	15.35	25.60	15.58	17.14	0.184189367	0.47
CR_00130C_A	19.03	29.18	3.66	43.84	20.10	13.36	0.64133963	0.47
CR_05150W_A	6.62	4.55	8.38	12.90	9.08	7.23	0.390695902	0.47
C1_00440W_A	50.05	46.15	48.95	79.51	68.85	67.98	0.006415998	0.47
C1_08300W_A	24.24	27.06	23.14	37.93	35.55	37.83	0.009699769	0.47
C2_02340C_A	89.25	90.83	89.80	147.57	125.34	129.90	0.006357512	0.47
C6_00240C_A	19.47	25.95	17.88	29.65	33.35	31.73	0.101701456	0.47
C2_05380W_A	11.39	7.46	9.37	13.91	12.59	15.47	0.067405004	0.46
C4_00920C_A	3.90	2.41	3.75	4.90	4.40	5.72	0.202638543	0.46
C3_01600W_A	40.30	37.27	43.71	59.00	58.41	63.49	0.003881958	0.46
C2_00700W_A	13.25	14.18	13.82	26.16	11.79	23.92	0.25779065	0.46
C4_06170C_A	26.39	54.35	45.94	83.64	39.53	68.18	0.378643092	0.46
C1_10990C_A	21.95	22.20	19.23	28.14	33.74	32.49	0.01814641	0.46
CR_03700C_A	9.99	8.99	7.78	13.45	13.97	12.32	0.022484102	0.46
C2_01930C_A	6.12	3.49	2.63	5.08	6.44	6.61	0.322834982	0.46
C2_06130W_A	17.03	5.59	7.02	20.02	6.87	16.68	0.519595037	0.46
C4_02090C_A	9.19	9.14	7.78	10.90	12.97	14.96	0.083182555	0.46
C2_01400C_A	0.76	0.68	0.52	0.24	1.26	1.41	0.606305238	0.46
C6_02190C_A	12.42	12.67	13.86	17.96	17.40	22.69	0.036555759	0.46
C1_06390W_A	8.53	5.23	4.71	7.92	7.87	11.53	0.25634288	0.46
C5_00390C_A	208.30	110.34	118.36	166.27	218.15	259.68	0.224145474	0.46
C5_03150W_A	45.58	42.80	49.69	84.33	65.30	55.04	0.102740417	0.46
C7_00450C_A	10.43	0.00	8.42	6.07	10.51	10.83	0.869840157	0.46
C1_03500W_A	116.80	142.41	148.86	209.83	188.52	210.03	0.041063241	0.46
C1_06270W_A	1.59	2.27	1.71	2.06	1.89	4.44	0.398951921	0.46
C1_05650W_A	4.06	5.23	5.01	9.10	6.65	5.54	0.270230281	0.46
C4_00680W_A	29.90	30.84	33.03	44.51	44.96	49.61	0.006424222	0.45
C1_11850W_A	68.98	43.41	49.11	75.35	76.42	85.98	0.062452443	0.45
C7_00130W_A	25.29	0.17	24.55	21.10	27.21	24.35	0.831431894	0.45
C7_01740C_A	5.81	3.09	3.16	4.35	5.46	8.02	0.3775724	0.45
C2_01150W_A	11.87	6.32	9.85	12.84	13.05	15.40	0.166491838	0.45
C3_07770C_A	31.45	21.73	22.62	30.93	46.18	34.08	0.1540072	0.45
C2_08560W_A	2.88	1.23	1.12	2.23	2.61	2.80	0.38673396	0.45
C3_02400C_A	36.63	12.94	11.84	14.52	31.10	43.95	0.553012063	0.45
CR_01730W_A	252.89	229.91	224.76	336.50	332.89	374.89	0.000469736	0.45
CR_07290W_A	13.03	7.81	7.06	12.96	12.95	14.99	0.187823351	0.45
C1_08540C_A	75.68	62.40	55.24	86.58	93.42	104.51	0.02344904	0.45
C1_03430W_A	12.56	10.55	7.44	18.85	15.22	10.68	0.280626588	0.45
C4_06130W_A	12.42	10.56	5.67	19.17	8.94	14.00	0.373769573	0.45
C1_02730W_A	2.95	2.37	2.37	3.58	3.61	4.16	0.171516301	0.45
CR_00360C_A	27.04	32.75	30.98	18.55	57.81	58.15	0.440031274	0.45
C1_07460C_A	8.19	7.41	2.70	5.55	13.12	8.05	0.516837021	0.45
C3_03480C_A	36.03	40.81	33.50	58.09	45.97	59.04	0.046120734	0.45
C1_10240C_A	12.84	11.81	21.18	26.52	21.48	19.40	0.272916006	0.45
CR_08900C_A	22.57	18.71	21.44	21.58	36.84	33.77	0.164453471	0.45
C1_07840W_A	42.49	37.53	45.86	64.60	59.10	61.43	0.012435492	0.45
C1_01590C_A	22.67	20.64	22.53	33.53	30.87	32.40	0.001341875	0.44

C3_07880C_A	21.70	16.39	16.48	35.22	23.48	21.20	0.219031822	0.44
CR_07310W_A	1.49	2.03	1.33	2.68	1.90	2.56	0.27010437	0.44
C2_08180C_A	1.57	1.24	0.75	1.71	1.81	1.72	0.366046723	0.44
C1_09140C_A	6.58	7.34	7.65	10.75	11.50	9.47	0.092025765	0.44
C3_01590W_A	5.66	2.57	6.18	6.26	7.76	7.02	0.342828691	0.44
C4_00300C_A	14.42	12.74	14.04	18.39	21.70	20.31	0.011477911	0.44
C4_03450C_A	15.87	12.55	12.22	22.73	16.11	20.74	0.073377534	0.44
C7_04000W_A	14.47	27.60	20.73	24.47	25.21	43.99	0.346824554	0.44
CR_05450C_A	17.04	21.43	16.26	17.39	18.31	45.67	0.430991519	0.44
C3_05020W_A	3.30	4.44	3.43	5.99	3.36	7.19	0.327514517	0.44
C1_13830C_A	87.98	84.53	75.02	118.20	119.81	124.23	0.003175587	0.44
CR_05560W_A	13.37	11.44	11.43	14.13	18.76	20.14	0.072208472	0.44
C7_03630C_A	4.27	4.97	5.14	7.59	7.27	6.27	0.342862951	0.44
C1_14250C_A	20.32	16.72	14.82	23.55	25.37	26.69	0.026667556	0.44
CR_03140C_A	11.20	10.46	10.47	13.75	15.46	17.77	0.047695956	0.44
C3_02980C_A	9.83	11.51	12.37	14.78	17.99	16.46	0.118084667	0.43
CR_05690W_A	15.96	22.94	16.36	30.09	25.01	25.98	0.157941815	0.43
CR_04550W_A	96.74	133.91	136.25	179.01	195.59	162.90	0.140315988	0.43
C1_04130W_A	1.61	1.23	1.31	1.69	2.37	1.98	0.195300446	0.43
CR_10810C_A	7.30	19.20	13.73	16.64	24.70	17.96	0.438529144	0.43
C3_07900C_A	9.49	8.23	8.68	13.99	13.27	11.17	0.071174475	0.43
C1_11930W_A	13.86	10.82	13.99	17.87	19.13	19.31	0.023936348	0.43
C1_03600W_A	34.81	2.07	36.90	33.93	34.07	38.08	0.69729299	0.43
C2_06300W_A	20.55	24.30	30.13	40.45	35.50	33.56	0.16128739	0.43
C3_03280C_A	120.90	233.40	170.11	181.80	186.20	411.68	0.434818289	0.43
C6_00970C_A	40.40	37.61	25.49	32.44	68.72	48.88	0.334146862	0.43
C2_05320W_A	6.10	4.15	3.92	5.85	7.70	6.96	0.168449218	0.43
C3_02420C_A	0.43	0.09	0.18	0.22	0.53	0.27	0.686302582	0.43
CR_02200C_A	31.43	53.09	43.98	82.88	48.93	56.49	0.307491389	0.43
C6_03750C_A	78.24	66.17	59.79	90.37	108.45	96.99	0.041367502	0.43
C3_04520C_A	33.08	36.80	42.31	65.73	48.28	49.34	0.125948687	0.43
C5_02680W_A	6.56	5.25	7.14	11.15	4.37	12.19	0.436764413	0.43
C1_06050C_A	222.90	144.33	177.00	216.52	281.71	288.72	0.115801633	0.43
C4_07200C_A	25.70	18.28	17.07	25.04	34.36	28.72	0.146168206	0.43
C3_03510C_A	7.04	1.89	5.41	7.36	5.13	8.13	0.494848923	0.43
C4_00540C_A	1.00	1.23	1.76	1.79	1.73	2.28	0.307202961	0.43
C2_08630C_A	9.20	6.36	6.51	9.12	9.60	13.29	0.161505743	0.43
C3_03160C_A	10.45	10.18	14.63	16.91	17.62	16.60	0.162307611	0.43
C6_03780C_A	16.62	13.68	12.07	23.40	17.16	20.85	0.086763818	0.43
C1_00930C_A	34.92	27.93	25.96	39.74	44.47	44.18	0.02623864	0.42
C1_06060C_A	9.74	5.71	5.93	5.46	13.06	12.36	0.463689344	0.42
C3_05220W_A	32.79	37.91	23.26	42.84	50.45	42.91	0.169568972	0.42
C6_04220C_A	10.01	12.79	12.57	14.12	18.10	19.29	0.145107681	0.42
C2_04050C_A	13.86	14.22	8.93	17.40	16.29	20.01	0.149370139	0.42
C3_01610W_A	73.34	92.61	107.71	160.16	118.39	119.58	0.18477642	0.42
C1_05240C_A	125.39	122.35	112.53	161.72	178.63	181.71	0.005229232	0.42
CR_04270C_A	1.77	2.07	2.76	2.29	3.06	4.11	0.487761482	0.42
CR_00340C_A	4.66	12.77	27.23	21.13	14.46	30.44	0.647364915	0.42
C3_07270C_A	722.07	793.27	794.42	1142.02	1085.01	1124.60	0.011570245	0.42
C7_01540W_A	0.31	0.26	0.53	0.54	0.57	0.47	0.585455759	0.42
C6_04500C_A	15.32	16.20	13.61	21.87	21.38	22.06	0.01537486	0.42
C1_12690C_A	2.12	1.08	0.93	0.98	2.39	2.58	0.605826077	0.42
C4_02470C_A	45.88	46.21	35.99	69.06	55.04	61.14	0.072096054	0.42
C3_01190C_A	10.09	7.16	7.57	11.62	11.98	12.20	0.065535563	0.42
C1_03690W_A	67.06	59.91	78.39	101.77	111.10	83.17	0.115723535	0.42
C2_02600C_A	5.83	8.62	7.97	11.42	10.66	10.42	0.207130682	0.42
C2_03610W_A	0.88	2.12	1.98	3.27	1.98	2.00	0.514585466	0.42
C2_09830C_A	9.68	4.22	4.21	2.97	11.94	11.09	0.64086994	0.42
C1_01350C_A	197.63	173.26	215.24	288.64	265.22	292.48	0.014563113	0.42
CR_02760C_A	8.08	12.60	10.77	16.38	15.21	13.98	0.207365618	0.42
C1_04210C_A	30.49	37.45	37.15	58.23	44.76	49.21	0.103990068	0.42
C4_06540W_A	3.04	2.11	2.04	3.44	3.20	3.72	0.131197526	0.42
C6_02930W_A	31.99	28.14	25.12	46.44	33.68	42.85	0.066955055	0.42

C5_00400C_A	7.85	8.55	9.92	12.72	12.04	13.29	0.060516208	0.42
CR_02130W_A	148.79	201.71	180.28	249.56	250.25	269.90	0.08764157	0.42
C1_08090C_A	35.76	24.38	23.74	49.92	49.52	19.62	0.441457161	0.42
C5_05300W_A	93.93	70.21	173.03	161.10	166.24	158.87	0.374864156	0.42
C1_03100W_A	28.90	31.39	30.82	44.27	43.62	43.61	0.011556867	0.42
C1_00130C_A	19.81	21.20	21.92	27.39	29.74	33.83	0.031128232	0.42
C1_03160C_A	18.23	22.13	23.15	30.95	27.60	33.31	0.088059647	0.42
C2_10680W_A	43.71	31.62	36.04	49.52	53.12	57.26	0.033724665	0.41
C1_01070C_A	10.45	6.40	9.09	15.09	10.65	11.47	0.212590696	0.41
C3_02920W_A	136.40	150.72	148.56	193.86	240.77	192.53	0.079564938	0.41
C1_09790C_A	12.83	13.20	9.73	15.30	18.48	17.71	0.112912539	0.41
C3_01810C_A	62.57	61.16	81.48	104.99	166.32	19.65	0.648647767	0.41
C1_13990W_A	21.48	14.47	18.99	25.86	26.76	26.11	0.09235161	0.41
C5_01320W_A	73.15	71.55	70.49	101.39	109.70	97.97	0.011620745	0.41
C1_09360C_A	14.17	13.85	19.73	28.35	18.05	22.42	0.222299048	0.41
C6_02030C_A	15.58	22.40	20.93	28.54	26.36	30.24	0.151773657	0.41
C1_02650W_A	104.26	102.25	121.53	143.12	172.95	155.33	0.056490483	0.41
CR_06740W_A	1.25	1.03	1.19	1.67	1.58	1.73	0.153797469	0.41
C4_04750W_A	22.14	19.15	16.59	28.29	29.03	25.57	0.057359195	0.41
C2_02500W_A	14.60	8.47	18.17	9.96	23.09	26.16	0.481882666	0.41
CR_05330W_A	17.55	13.53	13.00	26.76	19.42	17.28	0.313622548	0.41
C6_03900W_A	14.12	17.93	18.37	26.91	22.45	23.28	0.117488521	0.41
C1_04490W_A	68.14	59.13	54.41	84.11	83.12	93.24	0.011026306	0.41
CR_06420W_A	14.67	5.15	11.01	14.65	14.27	14.81	0.401325875	0.41
C3_07760C_A	6.46	6.75	8.83	10.68	9.91	11.04	0.153699288	0.41
C2_05480C_A	3.77	6.17	6.38	9.17	8.13	6.16	0.475611035	0.41
CR_01910C_A	34.38	82.54	50.60	81.14	87.08	74.46	0.405726696	0.41
C1_08610C_A	106.56	173.23	161.77	285.58	167.95	183.32	0.33994514	0.41
C7_03220C_A	34.74	30.13	32.81	47.45	45.76	46.44	0.002401128	0.41
C1_09030C_A	68.66	53.68	72.16	103.19	79.19	95.78	0.090812943	0.41
CR_01280C_A	8.49	5.61	7.90	10.26	9.04	12.15	0.216085808	0.41
C2_05190W_A	21.17	13.61	16.75	24.84	27.08	21.23	0.16285282	0.40
C6_00570C_A	21.28	18.90	23.96	34.31	29.14	28.21	0.073856904	0.40
C1_07340W_A	24.87	23.84	20.38	43.07	27.72	27.83	0.198599365	0.40
C2_06830C_A	6.44	4.54	5.51	7.02	8.48	7.99	0.086513091	0.40
C4_03050C_A	15.71	20.13	30.34	55.88	7.57	32.19	0.648554648	0.40
C1_04540C_A	5.52	6.38	5.89	8.51	8.16	8.80	0.0474353	0.40
C5_01850C_A	9.03	8.74	7.72	11.37	12.39	12.67	0.02288895	0.40
C1_09110W_A	1.04	0.55	0.39	0.75	1.15	0.96	0.562060084	0.40
C2_08140C_A	32.35	37.89	36.81	52.53	49.01	51.72	0.039378964	0.40
C2_09240C_A	2.83	0.74	0.72	1.21	2.07	2.78	0.687009307	0.40
C1_02080W_A	1.88	1.47	1.69	2.77	1.77	2.64	0.339955658	0.40
CR_06560C_A	40.15	40.35	31.67	40.00	57.57	62.61	0.184244182	0.40
C4_00560C_A	8.51	8.37	9.96	11.37	13.08	13.86	0.085893955	0.40
C2_02110C_A	4.07	2.32	2.79	3.05	5.08	4.96	0.445361518	0.40
CR_03600C_A	33.39	31.96	34.05	48.45	40.49	53.01	0.044823647	0.40
C7_01430C_A	1.82	3.41	3.66	5.53	2.40	4.90	0.536679563	0.40
C2_05920C_A	276.66	392.70	432.98	619.49	440.80	521.37	0.243483854	0.40
C5_04070C_A	28.47	27.59	29.79	35.85	40.87	45.49	0.076756912	0.40
C3_05570W_A	4.55	4.90	4.92	8.17	6.06	6.29	0.162992965	0.40
C1_13540W_A	1.48	0.99	1.01	1.28	1.57	2.08	0.373419517	0.40
C5_03900W_A	3485.06	3739.68	3776.48	4713.75	5450.08	5534.55	0.028653422	0.40
C3_05540C_A	2.22	1.59	0.72	2.31	1.24	2.92	0.671621159	0.40
C5_04640C_A	21.09	21.33	13.49	24.03	31.69	23.54	0.24438739	0.40
C1_00110W_A	69.99	46.06	48.92	68.28	77.54	87.86	0.121051027	0.40
CR_05710C_A	5.35	4.59	5.38	8.01	5.96	7.91	0.173051766	0.40
C3_06620W_A	10.48	9.97	11.24	16.85	15.36	12.79	0.092747473	0.40
CR_03090C_A	34.93	71.99	75.46	98.10	73.23	91.20	0.406542659	0.40
CR_00110W_A	14.38	6.12	8.63	4.45	24.41	11.77	0.634777448	0.40
C7_02630W_A	8.42	6.38	6.53	9.18	9.73	11.37	0.076741264	0.40
CR_09180W_A	4.27	6.45	2.09	5.01	6.80	6.49	0.530543052	0.40
C4_02130W_A	8.04	6.21	3.94	7.55	8.24	10.06	0.342862951	0.40
C3_01630W_A	45.31	44.93	51.62	65.28	65.28	71.41	0.021138433	0.40

C5_00220W_A	26.55	21.89	27.88	35.47	35.63	37.24	0.027002154	0.40
C4_04500C_A	10.43	14.16	13.66	20.05	17.13	17.37	0.190938529	0.39
C5_02470W_A	4.23	4.62	4.60	6.27	6.30	6.57	0.031307281	0.39
C1_04830W_A	24.30	28.39	30.14	39.46	39.08	39.48	0.06282219	0.39
C5_04900C_A	9.18	8.92	7.55	11.40	11.03	14.00	0.093551418	0.39
C5_02560C_A	7.37	6.48	6.38	8.19	7.92	12.70	0.195233351	0.39
C4_01400W_A	3.72	3.10	4.26	6.41	4.68	4.60	0.389697652	0.39
C6_00370C_A	20.72	15.14	18.25	31.91	19.52	25.19	0.201119582	0.39
CR_05670C_A	518.62	1021.06	674.34	892.87	1309.47	958.96	0.382627354	0.39
C2_05650W_A	14.51	11.70	10.97	14.73	18.58	19.23	0.091985343	0.39
C1_00600W_A	5.25	5.12	5.43	7.72	7.52	7.12	0.026041745	0.39
CR_01180W_A	9.87	4.02	10.96	9.32	11.83	13.87	0.473727733	0.39
C5_02360C_A	2.50	1.42	0.72	2.75	1.58	2.21	0.642762272	0.39
C7_03390C_A	30.63	41.55	47.90	55.87	53.52	61.90	0.208895948	0.39
C3_04290C_A	18.40	14.84	14.37	22.93	21.23	23.12	0.027769697	0.39
C2_04260W_A	4.83	4.65	4.21	5.86	6.38	7.15	0.075862401	0.39
C7_02140W_A	1.83	1.45	0.92	1.65	2.42	1.92	0.494557416	0.39
CR_09050C_A	0.21	6.07	1.41	2.98	3.20	5.11	0.773663571	0.39
CR_10830C_A	8.69	15.34	13.87	15.31	18.72	19.92	0.357659439	0.39
C1_04620W_A	13.40	10.49	8.32	13.27	16.69	15.40	0.185213813	0.39
C3_07060W_A	26.12	45.95	32.92	65.67	40.68	43.29	0.380844262	0.39
C1_11660W_A	66.69	110.07	114.15	170.64	117.80	126.19	0.334586002	0.39
C1_07500C_A	16.20	7.21	9.49	14.03	16.76	15.16	0.357875076	0.39
C3_01580W_A	4.80	2.93	5.39	6.57	6.10	5.83	0.301227978	0.39
C4_00690C_A	3.31	4.61	6.46	7.82	5.80	6.88	0.377469943	0.39
C4_06350C_A	7.70	5.44	6.03	8.86	9.17	9.00	0.090904529	0.39
C1_05400C_A	6.35	5.90	4.31	6.71	7.63	9.02	0.277778836	0.39
C1_14000C_A	17.64	7.00	16.63	14.29	20.68	22.90	0.447609667	0.39
C1_06590C_A	39.67	31.02	32.50	46.30	45.62	53.62	0.030862691	0.39
C3_00750W_A	3.54	1.07	2.18	2.65	3.50	3.29	0.509901078	0.39
C1_10340W_A	2.20	4.00	3.63	6.59	3.07	4.36	0.492937274	0.39
C2_08190W_A	20.69	10.68	10.10	14.93	22.07	20.88	0.386085834	0.38
C6_03070C_A	3.11	2.56	2.17	2.55	4.60	4.12	0.541994996	0.38
C1_00310W_A	33.35	42.59	33.63	55.40	46.38	53.49	0.118920471	0.38
C4_02890C_A	110.48	200.24	252.12	298.17	267.05	235.11	0.419426844	0.38
CR_03180W_A	15.06	13.39	11.90	21.19	18.60	17.01	0.106778237	0.38
C7_00850W_A	0.99	2.47	0.91	1.80	2.18	2.27	0.553628857	0.38
C2_03290W_A	22.02	18.10	18.11	21.55	21.71	39.13	0.298055812	0.38
C4_00280W_A	17.04	21.53	21.12	30.36	26.54	27.52	0.124801118	0.38
C6_04270W_A	10.02	11.21	8.60	16.64	11.99	13.47	0.156468804	0.38
C7_02560W_A	2.32	1.37	0.78	1.52	1.89	2.84	0.528675818	0.38
C3_07020W_A	103.75	86.32	63.51	128.20	120.63	106.09	0.184170797	0.38
C3_07950C_A	606.32	470.48	488.06	803.55	710.41	678.69	0.053759799	0.38
C3_03850C_A	9.18	30.77	34.47	24.72	46.72	34.63	0.604003336	0.38
C2_06120C_A	4.18	1.06	3.03	4.59	1.59	5.36	0.663127172	0.38
C2_01410C_A	16.57	17.28	22.76	27.33	24.70	27.88	0.140017924	0.38
C3_05890W_A	3.87	6.05	2.07	5.15	5.45	6.44	0.510184124	0.38
CR_04020C_A	27.12	25.26	21.12	36.08	29.61	37.83	0.099666147	0.38
C3_05550C_A	13.31	12.58	13.52	18.57	17.99	18.87	0.042080158	0.38
C3_03250W_A	2.07	2.37	3.76	3.88	3.95	3.73	0.32945594	0.38
C6_04110W_A	23.95	27.33	21.82	31.65	34.89	36.47	0.078616665	0.38
C7_02530C_A	6.47	6.05	6.17	7.59	7.94	10.79	0.138880222	0.38
C2_08310W_A	53.49	32.88	48.35	60.95	64.48	62.85	0.166189016	0.38
CR_06530W_A	26.50	26.09	23.74	30.09	38.75	38.38	0.117561482	0.38
C4_00960W_A	5.74	3.68	3.05	6.00	5.53	5.95	0.310294557	0.38
C1_09850C_A	72.94	70.10	44.28	80.42	110.79	70.09	0.330827918	0.38
C7_01480W_A	9.20	7.40	0.06	6.46	7.55	9.16	0.858634637	0.38
C2_10270W_A	2.28	1.70	1.91	2.28	2.60	3.41	0.247778987	0.38
C2_04020C_A	37.29	39.57	28.98	48.83	50.09	49.63	0.101724844	0.38
C1_04170C_A	522.17	969.54	1132.49	1609.46	1089.36	1011.82	0.451518957	0.38
C7_03770C_A	14.07	17.51	12.19	17.29	19.61	24.74	0.241811268	0.38
C2_05370C_A	16.44	11.63	10.74	17.85	17.74	18.62	0.136554289	0.38
C1_13300C_A	30.52	26.17	27.31	37.85	37.64	42.19	0.006083169	0.38

C5_04550W_A	17.30	15.20	19.42	28.17	22.04	22.50	0.146014638	0.38
C6_04470C_A	38.66	12.07	35.59	55.57	39.78	23.51	0.580949242	0.38
C2_10570W_A	24.39	10.87	15.54	20.34	22.95	27.26	0.346115721	0.38
C1_00230C_A	17.75	13.58	14.73	21.60	19.61	23.28	0.054179513	0.37
C4_02170C_A	1.18	1.54	0.90	1.81	1.62	1.69	0.405604077	0.37
C3_00460W_A	237.89	286.90	326.29	618.74	86.70	502.00	0.660578777	0.37
C6_00170C_A	32.83	20.82	23.63	33.02	38.32	36.09	0.151180404	0.37
C5_01890W_A	19.79	15.70	13.88	22.46	21.19	25.42	0.125231414	0.37
C7_02640W_A	7.67	6.50	7.23	9.08	10.71	10.09	0.068082465	0.37
C7_00410C_A	5.39	0.00	4.14	1.94	5.78	5.33	0.904272049	0.37
CR_08990C_A	44.00	61.44	63.22	29.31	96.95	112.05	0.576454625	0.37
C1_11810W_A	8.43	6.55	6.74	9.54	10.27	10.51	0.057094242	0.37
C5_03950W_A	4.13	3.30	2.95	4.32	4.53	5.67	0.168805849	0.37
C1_04090C_A	4.27	1.48	3.30	4.69	4.44	3.38	0.51633572	0.37
C2_08110W_A	10.18	9.81	9.93	15.05	12.93	13.88	0.04651361	0.37
C1_03530W_A	12.47	7.71	8.91	12.47	13.18	14.81	0.178033229	0.37
C3_03710W_A	10.00	10.66	9.46	14.71	14.56	12.80	0.120774269	0.37
C3_01720C_A	17.62	15.73	17.85	16.68	25.52	29.47	0.242722775	0.37
C3_01210C_A	39.66	31.69	31.81	48.48	45.20	50.19	0.032661018	0.37
C2_07500C_A	24.32	15.58	22.09	22.91	31.10	32.24	0.235151505	0.37
C2_03890W_A	3.12	1.61	1.68	2.90	2.71	3.32	0.435213825	0.37
C4_00940W_A	2.10	1.18	0.63	1.83	1.81	1.85	0.59687088	0.37
CR_07520C_A	38.08	41.92	39.89	59.44	51.57	56.65	0.056911798	0.37
C4_06830C_A	24.32	27.29	28.10	40.56	35.99	34.89	0.090150367	0.37
CR_05520W_A	3.02	3.35	2.21	3.89	4.11	3.98	0.241271	0.37
C1_09710C_A	6.37	4.98	6.63	12.53	10.33	1.88	0.676864265	0.37
C2_07820C_A	8.25	6.45	5.23	10.40	8.86	8.47	0.248917841	0.37
C1_02960C_A	9.31	27.14	21.30	26.26	27.20	28.27	0.514108045	0.37
C5_00630C_A	3.95	3.77	2.75	6.63	3.39	4.56	0.399682187	0.37
C3_00760W_A	20.00	23.08	29.47	33.59	33.98	33.87	0.189683602	0.37
C7_03450C_A	15.51	6.99	10.85	15.91	18.58	11.30	0.437001985	0.37
CR_09090C_A	2.41	3.94	3.96	4.78	4.31	5.38	0.38023935	0.37
C4_03170W_A	3.83	2.36	2.10	2.97	3.79	4.81	0.438529144	0.37
C7_00440C_A	112.38	0.21	119.10	56.72	129.52	131.47	0.887181802	0.37
C1_11300C_A	17.06	13.56	13.50	18.95	21.54	20.78	0.063620537	0.37
C1_08950W_A	39.82	34.19	35.05	57.48	48.59	45.34	0.075024543	0.37
C1_10840C_A	186.43	155.74	158.80	239.53	222.84	233.66	0.01006169	0.37
CR_05860W_A	17.77	20.91	17.10	24.12	29.45	24.08	0.182854655	0.36
CR_02170W_A	101.53	104.41	107.22	160.88	127.12	148.58	0.058349481	0.36
CR_03350C_A	4.71	4.64	6.40	7.43	6.64	7.88	0.209198946	0.36
C2_03340W_A	7.20	11.50	10.35	16.58	10.62	13.54	0.351897651	0.36
C5_00370W_A	391.22	222.54	30.17	273.47	302.96	303.78	0.714220557	0.36
C1_02490C_A	4.95	1.93	2.39	4.11	4.04	4.64	0.473371842	0.36
C2_09570C_A	3.66	2.32	4.05	5.40	3.78	4.82	0.422106313	0.36
C2_05400W_A	3.36	1.12	0.91	1.97	3.07	2.33	0.645390883	0.36
C1_03830C_A	3.77	2.78	3.35	5.38	4.08	4.30	0.28959611	0.36
C1_05680C_A	146.67	164.40	200.57	249.30	225.46	237.43	0.147190577	0.36
C3_01760W_A	15.98	12.74	12.05	17.74	20.24	18.37	0.089885576	0.36
C6_00030W_A	39.64	40.55	40.16	53.60	55.78	57.55	0.033818495	0.36
C6_04450W_A	13.66	4.06	12.94	19.29	14.06	8.47	0.614849374	0.36
C7_03760W_A	11.76	13.66	8.68	11.06	16.71	19.65	0.388986348	0.36
C3_04400C_A	8.34	9.03	10.05	14.22	11.05	12.83	0.140740962	0.36
C2_04030C_A	32.00	37.49	40.63	51.88	45.13	56.05	0.168027963	0.36
C1_00330C_A	51.56	49.12	51.86	63.31	77.61	70.10	0.074099101	0.36
C1_11740W_A	2.98	3.49	6.79	6.38	7.20	4.75	0.536101126	0.36
C4_07210W_A	33.11	25.77	24.57	30.27	43.10	41.66	0.203984239	0.36
C3_00940W_A	76.40	75.08	64.86	98.71	107.69	92.27	0.075372499	0.36
C2_06310C_A	206.24	189.09	204.91	262.70	279.81	287.22	0.010130811	0.36
CR_08590W_A	2.78	6.04	5.22	3.96	8.03	7.64	0.546150785	0.36
CR_05780W_A	112.27	152.17	117.97	57.73	283.44	188.39	0.629940589	0.36
C2_06960W_A	30.34	36.42	37.75	46.84	42.34	56.10	0.162796538	0.36
C1_05010C_A	4.46	3.37	3.88	4.12	5.65	6.40	0.317692008	0.35
CR_07210W_A	36.38	38.43	61.53	76.32	57.09	55.42	0.378147239	0.35

C3_03840C_A	5.29	11.19	14.25	11.77	18.22	12.75	0.549079969	0.35
C3_02640C_A	25.61	22.82	23.04	29.35	36.14	32.94	0.062288225	0.35
C2_07310W_A	20.21	34.63	36.79	44.03	48.13	34.97	0.411894076	0.35
C4_03150W_A	3.80	3.56	3.09	4.53	4.58	5.31	0.096669507	0.35
CR_03720W_A	410.84	541.97	455.64	652.56	659.66	638.63	0.143481064	0.35
C5_02010C_A	4.81	3.91	3.28	5.07	4.84	6.65	0.236085794	0.35
C6_02050W_A	4.46	3.77	3.70	5.39	5.43	5.63	0.136434429	0.35
C4_01200C_A	1.33	0.39	0.27	0.78	0.95	0.98	0.719122397	0.35
C1_13390W_A	0.96	0.71	1.01	1.08	1.13	1.49	0.411455856	0.35
CR_00350W_A	22.81	43.59	70.13	24.87	66.88	99.83	0.663719429	0.35
C5_02750C_A	7.91	4.99	7.93	9.77	8.79	10.04	0.22887949	0.35
C3_04220C_A	4.62	5.08	4.61	5.65	6.22	7.87	0.200512178	0.35
C6_03920W_A	44.69	57.37	62.45	81.76	66.73	79.41	0.212529603	0.35
C6_00160W_A	133.69	106.51	136.97	160.34	194.32	162.76	0.13944955	0.35
C3_07100C_A	5.15	6.62	6.30	8.31	7.97	8.68	0.167430605	0.35
C1_05230W_A	3.49	2.69	3.61	3.56	3.96	5.94	0.448877392	0.35
C3_05070W_A	3.71	2.27	2.60	3.80	3.23	4.75	0.359950656	0.35
CR_05220C_A	2.85	1.54	2.61	3.71	2.66	3.21	0.406538814	0.35
C2_00790C_A	6.53	7.71	10.53	18.69	3.05	12.70	0.681862398	0.35
C4_07060W_A	10.89	10.02	10.93	14.91	14.68	14.13	0.027742135	0.35
C1_14150C_A	24.15	23.90	33.69	37.74	33.90	40.91	0.248496392	0.35
C2_08550C_A	0.67	0.21	0.10	0.49	0.64	0.19	0.766063673	0.35
CR_03850W_A	1.61	1.92	1.85	2.78	1.94	2.68	0.336039634	0.35
C1_11090C_A	9.92	11.11	10.87	15.48	15.42	12.92	0.159910056	0.35
C6_00680C_A	24.26	21.72	19.69	25.86	28.41	35.94	0.175189153	0.35
C4_06840W_A	2.45	3.63	3.57	4.64	3.57	5.11	0.441766185	0.34
C2_07760W_A	24.33	26.59	16.27	41.23	24.72	26.17	0.414279122	0.34
C2_09840W_A	7.26	4.55	2.08	4.02	9.27	5.53	0.645395263	0.34
C1_11690W_A	14.15	10.90	21.22	23.37	19.08	20.98	0.38023935	0.34
CR_04450C_A	19.09	10.70	18.29	16.38	33.26	15.33	0.531482251	0.34
C5_01430C_A	5.15	6.51	6.37	8.10	8.10	8.56	0.207721395	0.34
C1_03330C_A	12.01	7.08	7.17	16.24	7.76	11.73	0.471693832	0.34
C5_03640W_A	1.39	12.35	10.32	7.32	21.43	4.36	0.778944783	0.34
C4_00220C_A	64.16	92.38	108.44	144.79	111.15	108.78	0.36499363	0.34
C4_05340W_A	2.96	2.33	2.83	3.10	4.07	3.95	0.229007341	0.34
C3_02830W_A	4.67	3.69	4.30	5.06	6.16	6.08	0.130344184	0.34
C3_06730W_A	2.29	2.17	3.59	4.32	3.46	3.24	0.482734226	0.34
C5_04370C_A	4.89	4.24	1.95	5.26	4.31	5.55	0.550605517	0.34
C1_00950C_A	12.03	13.16	13.20	19.17	15.20	18.29	0.122045267	0.34
C7_00730W_A	62.84	60.46	71.74	90.04	74.54	102.92	0.147066609	0.34
C3_03080W_A	5.69	2.86	5.14	6.97	6.47	5.18	0.440085626	0.34
C2_10560C_A	260.87	164.42	272.28	297.42	270.04	385.26	0.301440361	0.34
CR_07050C_A	5.59	4.39	8.54	7.37	11.97	5.79	0.526464501	0.34
C5_03290C_A	6.14	5.13	3.76	5.25	6.92	8.37	0.51603725	0.34
C1_02610W_A	6.27	5.11	5.68	7.99	7.58	7.69	0.047822989	0.34
C5_03050C_A	1.75	1.57	1.30	1.79	2.10	2.36	0.503857	0.34
C1_09200W_A	10.38	8.44	7.87	11.61	11.10	13.70	0.119158949	0.34
CR_06750C_A	65.36	94.82	97.70	125.51	108.63	120.33	0.281608648	0.34
CR_07590W_A	30.22	42.15	37.68	50.25	45.88	55.00	0.234430209	0.34
C2_01720C_A	4.96	2.35	3.92	5.65	3.39	6.37	0.581482155	0.34
C4_06500W_A	13.55	11.33	11.79	14.79	18.70	16.41	0.113156384	0.34
C7_00360W_A	65.43	41.73	64.44	82.59	75.66	74.75	0.230866551	0.34
CR_09190C_A	8.40	13.51	4.08	11.38	12.98	11.29	0.590489434	0.34
C2_07190C_A	28.89	12.83	17.60	18.82	25.08	36.49	0.511916452	0.34
CR_04930W_A	20.41	12.88	15.60	23.08	20.53	22.73	0.194296943	0.34
CR_07200W_A	3.34	2.23	2.20	3.12	3.76	3.71	0.33447544	0.34
C6_04060W_A	2.26	0.92	1.34	1.32	2.50	2.27	0.569321151	0.34
C7_03370C_A	39.54	68.74	16.79	44.86	64.41	62.47	0.627729009	0.34
C4_02340W_A	79.34	81.13	78.39	79.31	100.58	147.45	0.324762636	0.33
CR_03560W_A	11.62	12.01	11.61	16.44	14.74	16.84	0.109159925	0.33
C1_12260W_A	6.66	3.32	4.18	5.65	6.28	7.21	0.458834567	0.33
C1_03150C_A	26.83	30.96	29.19	32.86	42.07	43.79	0.19612218	0.33
C4_05790W_A	2.37	3.38	2.29	5.26	2.50	3.25	0.501618968	0.33

CR_00460C_A	3.55	3.62	4.41	6.69	1.82	7.41	0.666349928	0.33
C7_00350C_A	189.88	133.85	103.45	179.49	198.46	199.33	0.292435497	0.33
C5_00030W_A	14.85	8.41	4.50	10.44	14.92	11.90	0.586378639	0.33
C3_01330W_A	3.02	2.23	2.65	2.84	3.21	4.70	0.33994514	0.33
CR_07230W_A	98.13	68.47	71.27	106.88	108.68	106.20	0.142779191	0.33
C2_01640W_A	49.20	38.39	37.14	53.97	57.72	57.22	0.078263136	0.33
C1_06550W_A	64.43	61.21	77.14	94.70	89.22	91.95	0.100204535	0.33
C1_03340C_A	16.27	18.45	12.16	22.92	17.77	23.22	0.296420683	0.33
C7_00420C_A	21.43	0.00	19.38	8.66	22.29	23.47	0.911121179	0.33
C2_07430C_A	10.69	11.87	11.08	18.06	15.46	12.17	0.296043884	0.33
CR_05700C_A	22.18	15.45	14.19	22.61	26.16	21.17	0.28342493	0.33
C2_02190C_A	7.93	3.76	3.60	7.29	5.18	8.17	0.542329006	0.33
C1_08640W_A	83.05	59.60	54.65	69.30	92.93	104.72	0.292559898	0.33
C3_00410C_A	1.97	1.36	0.93	2.07	1.94	1.77	0.619584681	0.33
CR_06090W_A	42.76	41.32	38.46	62.96	50.76	52.58	0.10973759	0.33
C3_01260C_A	16.88	12.19	12.98	16.96	21.42	18.38	0.192153541	0.33
CR_07870W_A	6.04	9.15	3.41	8.67	8.32	8.40	0.54782835	0.33
C1_09260C_A	27.12	33.13	32.11	39.13	43.46	42.99	0.158995427	0.33
C1_13310W_A	4.18	3.36	3.06	5.69	4.28	4.39	0.291293029	0.33
C2_09280C_A	14.45	13.01	14.60	20.00	19.35	17.59	0.097898046	0.33
C2_02100W_A	10.48	6.23	9.67	9.93	14.20	11.34	0.351391789	0.33
CR_07150W_A	216.95	324.66	307.84	429.18	353.64	376.31	0.304344822	0.33
CR_09120C_A	107.20	126.83	142.02	102.62	205.23	204.27	0.453752359	0.33
C5_04080C_A	24.98	28.57	26.89	38.60	34.55	35.96	0.132389542	0.33
CR_08910C_A	57.47	48.50	46.15	37.15	83.18	85.49	0.484668828	0.33
C7_01440W_A	4.37	1.35	0.00	1.86	2.59	3.08	0.858271192	0.32
C2_01990C_A	6.89	6.21	5.22	8.08	8.11	8.55	0.090400345	0.32
C1_10920W_A	8.44	7.10	7.45	10.80	10.25	9.97	0.104147477	0.32
C2_05390C_A	2.16	0.80	1.45	1.45	2.07	2.37	0.693254782	0.32
C1_11950W_A	2.28	1.38	3.98	3.68	3.34	3.30	0.604224759	0.32
C1_12160W_A	106.16	79.06	96.70	93.81	147.45	138.34	0.301386497	0.32
C2_05030C_A	28.59	34.83	39.06	49.79	45.02	44.04	0.246744321	0.32
C3_04800C_A	13.59	23.82	10.83	20.53	22.74	22.35	0.492443031	0.32
CR_01110W_A	64.05	47.69	39.59	62.63	63.85	77.30	0.243240093	0.32
C4_00020W_A	159.02	118.48	115.46	140.08	212.73	174.63	0.290451047	0.32
C2_10340W_A	10.65	7.14	9.45	10.25	11.50	14.94	0.32309142	0.32
C4_03040W_A	27.55	36.13	42.25	60.86	39.61	43.07	0.438323051	0.32
CR_09370W_A	2.39	2.79	2.92	4.37	3.11	3.49	0.302179104	0.32
C7_03140W_A	0.92	1.41	1.78	2.08	1.62	1.87	0.518122393	0.32
C2_02870W_A	23.22	26.34	26.57	34.96	29.99	37.96	0.179679116	0.32
C2_01180W_A	11.74	1.43	2.48	1.76	11.15	7.83	0.82244987	0.32
C3_03520C_A	58.77	18.59	44.47	62.82	46.80	52.47	0.567189762	0.32
C3_00960W_A	2.70	4.06	2.11	3.76	4.28	3.95	0.498967305	0.32
C1_11290W_A	6.87	9.06	8.69	11.17	10.64	11.41	0.266404804	0.32
C5_01680C_A	1.98	1.54	1.18	1.59	2.25	2.47	0.464916232	0.32
C3_04380C_A	27.89	45.29	46.47	68.72	46.57	46.79	0.471463456	0.32
CR_03290C_A	0.38	0.99	0.76	1.03	0.92	0.93	0.663719429	0.32
CR_02590C_A	2.01	1.42	0.38	0.30	1.80	3.03	0.790602447	0.32
C4_04710W_A	29.34	22.50	18.77	32.36	33.77	28.33	0.260862067	0.32
C3_04600C_A	2.55	1.94	2.09	2.12	4.15	2.61	0.665718013	0.32
C2_06870C_A	48.47	52.69	70.01	79.06	68.85	83.54	0.273378939	0.32
C3_03540W_A	11.93	8.45	6.76	12.98	11.45	11.95	0.326732087	0.32
C2_08660C_A	2.11	1.27	1.51	2.01	1.80	2.78	0.455081775	0.32
C5_05330C_A	22.69	20.40	16.15	22.94	22.41	34.51	0.329170663	0.32
C1_06030C_A	3.30	0.81	0.98	0.79	2.42	3.61	0.77294734	0.32
CR_08560C_A	127.20	111.99	127.34	161.98	168.49	161.67	0.046196201	0.32
C3_05470W_A	20.54	27.28	17.50	24.69	32.13	31.36	0.344494474	0.32
C2_04730W_A	1.97	0.55	0.95	0.57	1.51	2.53	0.749759374	0.32
C6_00100C_A	119.02	158.33	149.14	198.92	199.21	177.03	0.246108478	0.31
C5_00150C_A	28.26	47.21	25.43	69.35	4.37	64.51	0.782008861	0.31
C6_04120C_A	5.71	3.58	3.47	5.42	5.83	5.79	0.316286628	0.31
C5_04870W_A	65.83	42.30	44.13	52.91	69.80	80.94	0.33988243	0.31
C4_04250W_A	4.60	4.48	5.68	6.32	6.94	6.58	0.235967222	0.31

C6_02600W_A	32.70	23.90	26.04	37.04	36.87	36.65	0.105025736	0.31
CR_05620C_A	2.11	3.36	1.82	3.43	3.07	3.32	0.490654356	0.31
CR_07850W_A	15.28	10.59	12.09	17.36	15.70	17.75	0.162992965	0.31
C1_03280W_A	23.02	23.72	26.07	31.73	34.96	31.05	0.148583128	0.31
CR_03170W_A	107.70	42.84	83.96	79.96	118.85	112.46	0.518082342	0.31
C1_06410W_A	4.73	2.59	2.34	2.95	4.54	5.38	0.534055323	0.31
C1_01030W_A	18.01	16.43	26.23	33.21	23.95	24.15	0.413388604	0.31
C2_00380C_A	44.28	45.76	39.38	72.18	20.78	82.20	0.638054857	0.31
C7_00520W_A	27.76	14.96	18.94	28.56	25.92	27.40	0.330852127	0.31
C2_03590C_A	36.36	31.31	34.26	49.02	44.67	42.69	0.117589712	0.31
CR_07910C_A	16.98	14.99	11.44	11.07	23.92	23.01	0.510883381	0.31
C3_02120W_A	26.72	28.77	24.72	36.82	35.28	35.36	0.108653444	0.31
C1_03290W_A	13.47	11.34	11.07	14.73	17.00	16.18	0.117201698	0.31
C1_10040W_A	36.85	30.33	31.94	44.40	38.98	49.27	0.088547834	0.31
C4_04220W_A	6.59	2.03	7.12	10.18	3.83	6.95	0.709506112	0.31
C3_01140W_A	6.35	5.77	3.02	5.24	7.05	7.92	0.547593539	0.31
C1_03060C_A	8.13	6.08	6.07	10.45	7.66	9.04	0.351091905	0.31
C4_01970W_A	7.65	7.51	8.19	15.66	6.34	9.31	0.541994996	0.31
C2_06090W_A	2.75	2.32	1.27	2.70	2.11	3.67	0.543393491	0.31
C3_05190C_A	73.62	74.01	73.57	103.82	95.52	96.57	0.052140735	0.31
C1_08160W_A	19.81	18.07	16.51	27.15	22.01	23.56	0.155498023	0.31
C3_02090C_A	49.10	53.45	53.52	63.69	72.54	72.75	0.118259038	0.31
CR_07450C_A	41.07	30.77	27.81	44.02	44.99	43.48	0.187196124	0.31
C2_02410W_A	47.59	57.77	47.34	53.22	73.77	77.83	0.311649288	0.31
C3_01800C_A	11.02	8.08	12.11	12.25	24.63	4.08	0.720037371	0.31
C1_04590W_A	72.77	42.95	64.77	62.00	83.25	95.42	0.412493973	0.31
C1_02220C_A	7.32	6.92	7.02	9.45	7.70	11.30	0.269807359	0.31
C4_06030W_A	232.67	380.43	351.13	472.68	405.58	418.43	0.390111765	0.30
C5_01920C_A	14.54	14.20	12.01	17.93	18.56	17.84	0.133280977	0.30
C1_11900C_A	6.78	5.70	4.47	7.05	7.78	7.77	0.303725176	0.30
C1_09090C_A	3.60	2.67	2.65	1.77	4.44	5.68	0.615642933	0.30
C2_07930C_A	41.61	28.60	26.58	32.69	51.18	44.26	0.387142895	0.30
CR_00250W_A	616.60	583.58	605.91	776.30	814.05	817.48	0.024170404	0.30
C3_06750W_A	41.28	29.49	37.34	36.28	44.28	63.53	0.421045126	0.30
C2_00660C_A	11.38	18.81	14.46	21.92	12.70	25.62	0.538139525	0.30
C2_00870W_A	11.35	12.68	10.10	14.13	16.62	14.73	0.313042142	0.30
C1_07110W_A	18.25	16.88	14.30	31.81	13.32	20.72	0.516560518	0.30
C6_00840W_A	72.27	60.41	57.73	64.73	84.18	104.67	0.305311686	0.30
C2_01690W_A	79.00	83.70	88.23	195.92	50.29	88.56	0.655363686	0.30
C6_02330W_A	470.88	345.36	382.92	519.87	594.17	471.07	0.21621926	0.30
C4_03540C_A	10.50	10.44	12.63	13.83	13.94	17.01	0.206343466	0.30
C6_03940C_A	49.48	47.40	48.18	59.69	66.97	66.32	0.056365125	0.30
C1_00620W_A	29.91	44.10	29.63	55.02	42.46	40.97	0.414626203	0.30
C4_02530W_A	2.56	0.94	1.24	2.01	1.92	2.31	0.593662989	0.30
C3_04890W_A	5.67	9.35	6.45	8.90	11.57	8.21	0.464490645	0.30
C3_02500W_A	11.31	13.45	15.23	13.77	18.57	21.04	0.406980729	0.30
C2_03330C_A	65.79	81.39	55.90	84.87	106.40	78.76	0.362386119	0.30
C1_12510W_A	2.39	2.69	2.44	3.69	3.10	3.22	0.224900778	0.30
CR_05200C_A	6.76	2.29	3.62	5.68	5.80	5.13	0.650735222	0.30
C1_13780W_A	5.13	6.57	5.24	8.74	4.76	9.20	0.471068242	0.30
C2_08460C_A	6.42	2.49	1.05	2.13	4.85	6.14	0.771518985	0.30
C5_02260C_A	9.18	11.84	10.93	15.73	12.03	14.90	0.305311686	0.30
C4_05640C_A	11.21	15.56	12.91	17.33	16.72	18.96	0.311836946	0.30
C3_01400W_A	14.39	9.50	6.41	6.96	14.84	18.29	0.629128345	0.30
C3_03900C_A	1437.07	1108.44	1399.88	1842.75	1915.51	1452.76	0.258461191	0.30
C2_01490C_A	20.46	22.57	22.28	28.74	30.21	27.91	0.145368146	0.30
C2_04080W_A	7.40	8.13	3.06	5.45	8.74	10.59	0.627296619	0.30
C5_03110C_A	4.24	7.76	5.25	6.17	7.97	8.83	0.579231182	0.30
C4_07130W_A	12.80	7.94	9.15	10.66	14.07	14.71	0.342315025	0.30
C5_01190W_A	16.49	19.98	17.81	22.92	26.44	22.78	0.24719684	0.30
C4_06810C_A	28.08	25.12	24.35	38.55	31.23	33.02	0.132542829	0.30
C2_10530C_A	36.48	33.62	33.78	49.96	44.32	43.40	0.096521906	0.30
C3_06830C_A	3.18	2.39	2.50	3.52	3.68	3.48	0.201874054	0.30

C3_01250W_A	22.42	19.91	17.57	24.58	26.21	28.62	0.110611783	0.30
C4_02290W_A	40.63	43.15	43.90	55.96	55.61	58.05	0.084038044	0.30
CR_06810W_A	714.05	801.77	951.38	1055.75	1128.46	1097.59	0.222770765	0.30
CR_06800C_A	856.83	905.61	1129.56	1260.16	1292.08	1291.48	0.210599684	0.30
CR_00060C_A	30.19	27.90	29.70	34.62	46.12	35.29	0.243614071	0.29
C1_05210C_A	4.89	5.72	5.39	8.53	6.81	5.88	0.396657053	0.29
C3_00400C_A	12.16	10.37	11.26	13.67	14.55	16.54	0.094130543	0.29
C4_01420W_A	0.93	0.55	0.56	0.79	0.88	1.04	0.452092991	0.29
C1_02880C_A	60.54	42.84	45.51	71.63	62.12	62.35	0.21985297	0.29
CR_07490C_A	99.31	151.81	168.98	214.57	178.93	165.94	0.447328074	0.29
C1_08440C_A	5.57	5.13	7.73	9.54	6.64	8.20	0.525567482	0.29
C7_03780C_A	4.48	4.26	2.04	3.40	5.08	5.76	0.595874911	0.29
C7_00610C_A	17.28	13.78	9.46	20.78	16.57	15.94	0.429734467	0.29
CR_00420W_A	4.14	3.09	5.73	5.49	5.98	5.65	0.463216772	0.29
C4_05600W_A	9.88	9.47	5.93	16.45	5.64	11.36	0.623210616	0.29
CR_06720W_A	7.58	7.16	8.80	10.21	9.59	11.36	0.210966319	0.29
C5_00890C_A	2.07	2.45	2.73	3.49	2.60	3.50	0.432686279	0.29
C1_03650C_A	23.27	12.48	37.86	30.66	32.35	34.06	0.610598916	0.29
C1_00080C_A	97.19	53.48	62.19	71.34	99.04	108.57	0.446786585	0.29
C7_02300W_A	9.05	6.78	5.48	9.13	9.69	9.18	0.336191952	0.29
C1_10780C_A	97.29	111.80	106.81	141.64	129.36	147.23	0.150335206	0.29
C5_04110W_A	105.46	97.81	85.48	124.09	126.68	129.89	0.084184103	0.29
C3_00700W_A	1.74	1.09	0.99	1.47	1.62	1.95	0.46605464	0.29
CR_01100C_A	15.24	17.41	8.98	16.71	16.35	22.06	0.522813384	0.29
C1_13740W_A	3.45	3.66	3.92	4.33	4.92	5.33	0.258159033	0.29
C6_01300W_A	29.91	25.79	24.29	32.03	31.22	42.36	0.214467362	0.29
C4_01320C_A	2.80	2.36	1.78	2.90	3.00	3.26	0.354226684	0.29
C2_08830W_A	70.83	65.14	56.64	75.12	84.38	94.44	0.162668759	0.29
C3_04390W_A	163.47	209.33	184.92	239.78	250.10	248.30	0.226537841	0.29
C7_01640W_A	264.53	224.25	208.53	289.80	328.82	297.11	0.138126988	0.29
C1_14420W_A	47.39	17.62	112.01	101.01	106.68	22.46	0.789052416	0.29
C6_01220C_A	11.69	7.97	10.40	14.76	10.88	13.85	0.317394329	0.28
C4_05910C_A	6.36	5.19	4.43	7.10	6.76	7.14	0.227100589	0.28
C2_10550C_A	15.94	17.59	14.71	21.84	20.48	21.27	0.138570971	0.28
CR_10030W_A	32.10	19.36	18.54	36.70	26.72	27.93	0.444100132	0.28
C3_06320W_A	3.29	3.96	3.07	4.28	4.59	4.74	0.259819214	0.28
C1_09830W_A	8.38	16.01	4.36	12.80	13.17	12.13	0.667141972	0.28
C1_08180C_A	36.24	34.41	30.00	42.28	43.09	46.99	0.090465811	0.28
C4_01510W_A	55.45	67.00	52.85	91.16	72.10	67.49	0.338744878	0.28
C4_00800W_A	1.42	1.69	0.31	1.08	1.94	1.48	0.771070756	0.28
CR_07280W_A	3.32	5.43	4.67	5.09	5.80	6.80	0.517588903	0.28
CR_08640C_A	7.64	6.22	21.43	9.75	17.68	19.24	0.710977522	0.28
C2_03930C_A	10.76	6.85	7.28	10.92	9.90	11.78	0.337378192	0.28
C5_04780W_A	11.38	8.44	10.41	13.15	12.19	14.32	0.205251358	0.28
C5_05470W_A	21.30	19.74	18.56	24.85	28.06	25.26	0.133484449	0.28
C1_01880C_A	4.91	7.52	6.72	8.20	7.85	9.29	0.408792966	0.28
C2_08670C_A	4.84	2.72	4.30	2.98	5.67	6.85	0.611312076	0.28
C4_04300C_A	3.37	2.55	3.45	2.57	5.72	3.98	0.680169771	0.28
CR_03620C_A	39.31	29.66	31.94	33.59	48.08	50.46	0.329616851	0.28
C4_05570C_A	4.74	4.38	4.00	3.55	7.02	6.60	0.532405941	0.28
C3_07660W_A	5.01	4.89	4.30	6.53	5.45	6.67	0.228092636	0.28
C3_05910W_A	1.03	2.07	0.36	1.34	1.75	1.48	0.749864047	0.28
CR_06970C_A	7.56	3.97	4.29	6.24	5.58	8.84	0.544565529	0.28
CR_08180C_A	24.72	10.49	17.06	21.45	7.13	40.26	0.743118068	0.28
C1_00570C_A	13.05	7.29	8.09	10.07	13.86	13.02	0.456820881	0.28
C1_02510W_A	4.25	5.86	5.04	7.93	5.20	6.85	0.504836347	0.28
CR_07160C_A	26.32	33.52	32.49	42.62	36.30	42.70	0.280469185	0.28
C6_00550W_A	13.17	3.72	11.87	9.96	11.83	15.60	0.677906841	0.28
C2_02260W_A	94.54	65.15	66.17	93.75	95.52	105.27	0.245833384	0.28
C2_04820W_A	4.40	2.90	3.08	4.55	3.85	5.19	0.421045126	0.28
C6_03120W_A	2.48	1.93	0.79	2.21	2.39	2.17	0.649073212	0.28
CR_10820W_A	10.10	28.12	25.14	25.83	33.92	24.13	0.669329494	0.28
C1_04240C_A	868.78	1235.45	1542.96	1732.22	1468.49	1606.50	0.458230111	0.28

C3_07750W_A	9.88	8.46	15.07	14.06	14.14	15.54	0.46333404	0.28
C3_03000W_A	14.00	12.16	11.88	15.13	18.16	16.29	0.320855331	0.28
C2_02840C_A	2.99	2.92	2.55	3.70	3.21	4.15	0.250195733	0.27
C1_08050W_A	130.36	105.27	117.03	175.07	139.05	145.65	0.20399381	0.27
C4_01150W_A	8.85	5.69	6.11	10.32	7.67	8.90	0.395660821	0.27
C6_03140C_A	5.01	5.22	4.78	7.00	6.55	6.08	0.231841773	0.27
C4_01280C_A	14.69	16.16	12.79	15.25	17.05	25.04	0.420059218	0.27
C1_02280C_A	1.83	1.53	1.72	2.07	2.04	2.52	0.31481613	0.27
C4_01120C_A	3.48	3.48	4.18	4.21	4.33	6.03	0.453687998	0.27
CR_06150C_A	51.24	64.95	87.15	104.60	73.56	89.14	0.476970313	0.27
C5_02720W_A	19.62	16.63	20.63	18.60	26.10	29.68	0.388986348	0.27
C2_07390C_A	31.18	30.12	14.14	43.63	34.15	19.87	0.64613499	0.27
C7_00810W_A	10.21	8.50	9.03	0.06	18.12	18.08	0.910712662	0.27
C1_13420C_A	21.82	12.44	12.06	17.79	20.04	22.13	0.4478816	0.27
C2_10840W_A	7.35	5.35	6.52	9.40	6.80	8.83	0.325422944	0.27
CR_03460W_A	35.17	28.77	32.57	43.15	38.74	43.89	0.114074262	0.27
C2_03880C_A	3.85	2.29	3.61	1.00	6.13	5.55	0.800022931	0.27
C1_07280C_A	7.82	7.71	10.80	12.44	11.77	10.14	0.564827412	0.27
C6_04100W_A	63.76	50.91	44.43	57.85	73.28	75.46	0.309624287	0.27
C5_04430C_A	27.70	57.24	57.02	66.64	54.91	65.51	0.577630971	0.27
C1_11340W_A	1.05	1.14	1.15	1.04	1.15	2.18	0.635761722	0.27
C2_08760C_A	66.77	41.05	48.99	69.73	64.91	68.03	0.310294557	0.27
C1_01900C_A	9.23	6.77	6.25	9.99	8.18	10.76	0.359728886	0.27
CR_10300W_A	168.31	178.18	178.93	233.25	221.16	229.73	0.108511335	0.27
C3_02620C_A	7.54	6.36	8.29	4.69	12.56	11.60	0.677137885	0.27
C1_12740W_A	6.58	2.41	3.89	4.31	5.08	7.21	0.632592483	0.27
C3_00840C_A	6.56	7.71	9.54	8.11	9.92	12.98	0.537041883	0.27
C6_02430W_A	3.96	1.85	1.74	3.31	2.72	3.70	0.597764464	0.27
C5_04880C_A	5.29	7.17	8.31	11.83	6.97	8.33	0.549660193	0.27
CR_03240C_A	4.28	1.99	1.79	2.93	2.84	4.66	0.65483529	0.27
C1_00460W_A	90.61	116.43	140.48	148.72	151.75	152.97	0.405375705	0.27
C4_04760C_A	4.74	4.51	4.26	5.82	6.50	5.21	0.428144025	0.27
C6_02040W_A	44.82	77.75	85.12	115.27	71.04	85.94	0.580949242	0.26
C5_05210W_A	18.35	11.77	22.41	26.68	20.95	20.29	0.511664516	0.26
C2_01590W_A	221.21	382.11	449.17	601.66	365.55	410.44	0.601316423	0.26
C4_03920W_A	7.45	5.39	4.78	5.50	8.74	8.51	0.502978832	0.26
CR_07830C_A	78.59	61.44	123.85	109.56	77.45	157.74	0.610598916	0.26
C2_02460W_A	19.54	19.81	31.49	31.27	28.69	32.21	0.455335334	0.26
C4_04100C_A	1.18	1.54	1.36	1.27	2.10	1.84	0.698263137	0.26
C1_08560W_A	20.23	20.06	22.37	29.71	26.53	24.88	0.235537157	0.26
CR_09700W_A	2.42	0.61	1.77	1.98	2.37	1.77	0.727475424	0.26
C3_07120W_A	7.83	4.19	7.07	7.25	8.98	8.37	0.535828761	0.26
C5_04360C_A	41.97	40.90	43.79	64.21	42.96	57.14	0.333323041	0.26
C4_04110W_A	28.69	13.91	9.47	16.38	15.82	35.25	0.740801425	0.26
C3_05860C_A	3.83	1.93	4.17	3.83	2.63	6.46	0.691685586	0.26
C2_02050C_A	9.36	6.86	5.87	10.05	7.93	10.54	0.39721265	0.26
C1_13920W_A	59.09	34.27	50.19	52.38	61.08	71.54	0.429630886	0.26
CR_09750C_A	15.33	14.60	17.64	25.69	16.30	19.69	0.427114639	0.26
C4_06050W_A	23.69	21.43	16.11	28.86	24.99	25.29	0.370061053	0.26
CR_09550C_A	25.08	21.68	45.37	43.94	36.17	39.35	0.580736488	0.26
C6_03930W_A	3.51	4.99	3.84	5.29	4.81	5.97	0.438421939	0.26
C7_00030W_A	207.97	11.12	157.74	155.93	185.01	134.62	0.821609352	0.26
C3_05060W_A	6.78	6.43	5.12	8.79	7.02	7.94	0.357744751	0.26
C3_03720W_A	63.46	55.73	50.51	65.03	80.91	72.87	0.251159334	0.26
C7_00110W_A	248.39	0.99	256.04	215.13	237.09	187.85	0.915008306	0.26
C1_13250W_A	6.48	7.68	9.68	11.35	8.08	11.54	0.502864511	0.26
C2_02830C_A	93.77	97.06	98.86	117.51	117.33	140.53	0.156041441	0.26
CR_09490W_A	4.14	1.62	3.39	2.51	4.95	4.25	0.671479547	0.26
C2_10200W_A	9.25	10.01	10.95	9.85	10.76	18.56	0.568567521	0.26
C6_02920C_A	14.94	13.13	11.99	15.32	17.25	19.11	0.266217957	0.26
C5_03960W_A	211.12	188.05	181.46	232.67	250.77	264.83	0.106350554	0.26
C3_03500W_A	10.02	6.26	12.58	15.07	9.24	12.85	0.578764499	0.25
C1_01670C_A	0.78	0.41	0.58	0.84	0.77	0.68	0.579269153	0.25

CR_04160C_A	4.90	5.15	5.53	8.88	5.21	6.04	0.519581843	0.25
C4_00610W_A	9.71	11.06	10.94	14.64	12.87	13.37	0.312181124	0.25
C6_02110W_A	2.86	2.55	2.51	2.90	3.29	3.95	0.469324295	0.25
C5_03580C_A	8.37	8.30	7.51	8.94	11.00	11.20	0.29939734	0.25
C7_03360W_A	87.07	151.61	105.90	169.82	144.54	132.41	0.529649598	0.25
CR_09210W_A	8.42	13.46	1.06	9.90	6.27	13.72	0.821014901	0.25
C3_03030C_A	25.41	24.80	24.93	34.84	32.40	29.44	0.210369374	0.25
C6_03550C_A	4.93	3.92	4.87	6.16	5.05	6.44	0.349823327	0.25
C2_02960C_A	5.24	5.62	5.87	6.89	6.10	8.62	0.400736775	0.25
C2_01230W_A	2.15	0.59	1.50	0.67	2.97	1.75	0.813296133	0.25
C1_00700W_A	10.99	5.09	5.41	4.54	9.43	13.61	0.739455274	0.25
C7_01270C_A	1.68	1.45	1.66	2.34	1.89	1.95	0.471316818	0.25
C4_03020W_A	25.98	25.71	33.11	37.55	33.68	38.15	0.294080772	0.25
C2_07810W_A	18.94	19.77	10.35	25.85	17.90	19.32	0.576085001	0.25
CR_01290C_A	8.18	8.01	9.55	10.38	11.66	11.06	0.259069812	0.25
C2_02270C_A	10.94	8.84	11.64	11.21	12.34	16.83	0.481882666	0.25
C2_08960C_A	11.03	8.56	9.88	13.34	12.80	11.66	0.284330571	0.25
C1_00790W_A	1.82	1.45	1.10	1.63	1.86	2.15	0.612254696	0.25
C1_05410C_A	26.25	57.26	49.70	72.21	48.11	52.82	0.637882512	0.25
CR_07180W_A	10.47	11.99	12.39	14.61	13.59	16.70	0.308528144	0.25
C4_05260W_A	1.61	1.12	1.78	2.30	1.39	2.14	0.654262166	0.25
CR_01080W_A	1.77	0.84	0.67	0.78	0.99	2.44	0.751256312	0.25
C2_03060W_A	75.87	115.68	116.85	153.87	115.07	129.94	0.501022294	0.25
C3_00300W_A	9.50	10.05	9.50	12.16	8.60	16.78	0.507785685	0.25
C7_03520W_A	13.31	10.08	12.04	10.50	15.03	19.92	0.535729552	0.25
C3_02930W_A	2.07	2.11	1.14	2.18	2.54	2.07	0.707725084	0.25
C2_01350C_A	11.89	10.41	10.53	12.63	15.66	13.77	0.265887247	0.25
C3_03410C_A	85.72	91.05	83.28	104.96	108.85	120.58	0.147838667	0.25
CR_05750W_A	145.06	83.49	103.30	97.88	152.18	173.29	0.546595256	0.25
C4_02630C_A	18.23	20.67	20.38	23.52	20.78	32.11	0.450720668	0.25
C7_03590C_A	2.74	3.26	3.03	3.55	3.57	4.50	0.426617303	0.25
C1_05780W_A	18.88	13.70	16.77	19.69	20.36	23.09	0.25773568	0.25
C3_04760C_A	4.25	3.87	4.79	5.77	5.59	5.18	0.276584957	0.25
CR_01970C_A	138.04	147.96	143.90	179.89	185.38	186.52	0.150679237	0.25
CR_02390W_A	39.09	28.27	31.37	39.57	40.76	45.83	0.233282783	0.25
C4_00240C_A	33.87	38.77	38.91	51.58	43.99	47.78	0.284401036	0.25
CR_00610W_A	2.56	2.51	2.80	3.68	3.03	3.38	0.296357613	0.24
C1_09660W_A	25.62	35.29	33.47	46.13	35.62	39.72	0.435750743	0.24
C6_00710W_A	9.96	6.87	5.97	8.18	9.21	11.70	0.490654356	0.24
CR_06470W_A	31.27	24.22	22.87	29.05	31.96	39.10	0.334586002	0.24
C1_05200C_A	26.61	19.13	18.34	22.71	29.94	28.92	0.420026932	0.24
CR_09070C_A	10.40	5.55	5.75	6.69	10.06	10.81	0.603535711	0.24
C4_05650W_A	9.09	7.23	13.60	14.61	11.47	12.15	0.570769325	0.24
C1_12770W_A	15.02	6.27	8.36	11.50	12.66	13.28	0.59687088	0.24
C5_02620C_A	9.77	6.63	5.40	11.67	6.82	9.21	0.580030237	0.24
C4_01440W_A	7.13	8.89	7.14	11.21	8.48	9.98	0.435795336	0.24
CR_03420C_A	33.49	29.18	26.09	39.56	36.71	36.82	0.216462991	0.24
CR_04280C_A	3.46	4.07	5.15	4.14	6.71	5.37	0.581913088	0.24
C1_00640C_A	2.83	3.45	2.20	5.45	2.59	2.81	0.661102067	0.24
C7_01450C_A	12.08	11.55	0.08	9.72	10.01	10.27	0.916641342	0.24
C6_03470W_A	8.57	7.62	5.68	9.01	9.95	8.84	0.423282513	0.24
CR_06450W_A	0.74	0.92	0.78	1.00	1.05	1.06	0.598980653	0.24
C1_06980C_A	21.20	18.58	19.52	24.46	33.58	16.95	0.577925128	0.24
C3_01090W_A	12.98	4.77	5.10	5.64	11.59	11.65	0.766096756	0.24
C1_08400C_A	20.31	16.05	19.77	26.92	22.19	22.22	0.308294155	0.24
C1_04810W_A	21.75	14.40	20.10	22.17	26.17	22.88	0.403516306	0.24
C1_10470W_A	22.41	21.93	28.27	33.96	28.48	30.10	0.375300746	0.24
C7_04030C_A	13.78	16.21	17.12	16.40	17.06	27.00	0.50893033	0.24
C4_06740C_A	20.79	18.99	19.66	26.51	23.39	25.74	0.14772381	0.24
C1_02290C_A	2.66	1.44	1.93	2.31	2.34	2.98	0.61681761	0.23
CR_03810W_A	38.58	27.37	23.56	32.69	39.50	40.93	0.447650981	0.23
C3_03760W_A	39.19	32.26	36.89	44.06	45.98	47.33	0.153087349	0.23
C5_03800W_A	18.94	30.16	36.87	63.10	22.40	24.28	0.750054831	0.23

C5_02900W_A	4.03	8.09	5.86	8.17	6.94	7.95	0.615652532	0.23
CR_02710W_A	1.31	0.84	1.03	1.47	1.16	1.40	0.620152708	0.23
C4_05110C_A	17.54	21.46	17.72	23.54	20.57	28.30	0.413780764	0.23
C4_03280W_A	96.74	101.87	61.46	160.07	110.86	56.62	0.703760498	0.23
C5_01870W_A	20.86	15.33	15.91	22.87	20.64	22.41	0.283196958	0.23
C6_01380C_A	4.65	1.81	1.50	1.77	4.00	4.26	0.788519164	0.23
C7_02020W_A	14.08	17.02	13.46	15.80	20.10	20.75	0.436764413	0.23
C2_01650W_A	0.37	0.35	0.71	0.54	0.83	0.40	0.789052416	0.23
C6_00230W_A	6.39	7.23	6.15	7.89	9.78	7.40	0.482700504	0.23
C1_02250W_A	9.09	4.13	4.91	3.71	10.02	9.02	0.746894428	0.23
C1_07640C_A	1.05	0.36	0.53	0.67	0.87	0.91	0.743475368	0.23
C7_01930C_A	46.16	37.00	31.01	55.62	41.76	46.84	0.441766185	0.23
C1_11630C_A	0.88	0.61	1.36	1.17	1.55	0.88	0.759983939	0.23
C2_09640W_A	16.58	31.87	30.98	40.27	31.95	29.19	0.637796534	0.23
C6_03130W_A	43.08	80.04	76.32	99.97	71.87	82.70	0.635356797	0.23
C6_02130C_A	1.51	0.99	1.06	0.81	1.50	2.20	0.751777226	0.23
C6_04340W_A	1.88	1.04	0.43	1.70	0.85	1.66	0.796581984	0.23
CR_04050C_A	13.98	14.08	12.63	15.87	16.58	19.05	0.254191663	0.23
CR_07560W_A	66.10	55.80	61.66	70.45	82.49	78.57	0.21311848	0.23
C6_03380W_A	12.97	8.95	11.25	11.89	12.66	17.34	0.481300751	0.23
CR_02120C_A	18.83	15.03	16.48	18.97	22.31	22.17	0.327585698	0.23
C2_08730W_A	5.28	2.64	8.13	7.38	6.21	6.61	0.70580218	0.23
C1_10680C_A	33.95	35.32	36.95	45.09	44.22	44.96	0.190731862	0.22
C2_05790C_A	42.11	32.44	31.23	48.87	43.01	40.93	0.348935452	0.22
C1_03940W_A	23.32	22.85	25.75	30.89	24.37	35.82	0.435213825	0.22
C1_09350W_A	19.82	34.53	39.58	61.66	25.45	32.30	0.731181819	0.22
C1_07920W_A	13.81	12.06	18.50	17.47	20.90	17.38	0.610598916	0.22
C2_09560C_A	7.14	5.06	5.21	7.06	6.82	8.04	0.397122415	0.22
C3_04700W_A	13.18	11.29	8.51	13.40	15.20	12.82	0.489908844	0.22
CR_00220W_A	10.54	11.43	11.81	13.62	14.08	14.94	0.301404329	0.22
C2_10600W_A	43.91	36.96	45.35	52.18	50.91	55.73	0.296402431	0.22
C1_04790W_A	22.05	20.45	22.71	27.58	25.42	29.17	0.262707037	0.22
C6_00210W_A	52.95	52.87	53.50	64.93	66.38	69.39	0.151773657	0.22
C1_11760C_A	48.83	37.57	40.18	56.45	49.16	53.30	0.291789476	0.22
CR_06760C_A	215.93	206.86	223.85	271.50	294.67	245.95	0.294218284	0.22
C1_13120C_A	3.75	1.87	3.08	1.66	4.17	5.06	0.741183013	0.22
C4_06230C_A	11.94	11.66	10.68	17.23	12.07	13.83	0.427462894	0.22
C3_04820C_A	14.25	15.48	11.36	17.36	17.63	16.68	0.387142895	0.22
C1_06530C_A	4.70	5.60	5.64	5.83	5.98	8.36	0.481139377	0.22
C7_01890C_A	11.72	6.97	9.00	9.86	11.56	13.48	0.657985719	0.22
CR_08950W_A	76.12	100.47	99.85	54.60	137.28	158.40	0.714086619	0.22
C1_11590W_A	24.68	20.13	19.13	29.53	30.24	19.88	0.525567482	0.22
CR_03610C_A	23.29	16.07	16.38	21.71	23.18	24.76	0.393743043	0.22
C2_00190C_A	43.23	38.42	42.87	45.17	59.00	51.87	0.354198226	0.22
C4_01460C_A	11.71	9.62	9.70	14.57	8.65	15.78	0.592289418	0.22
C2_01890W_A	4.39	2.54	2.20	3.89	3.59	3.89	0.605776908	0.22
C5_03260C_A	3.28	3.31	2.39	4.58	3.32	3.35	0.550051427	0.22
C1_10730W_A	20.85	17.68	18.53	22.94	23.05	25.55	0.18477642	0.22
C6_00360C_A	27.64	25.88	37.47	44.09	34.66	35.45	0.526696391	0.22
C5_04540C_A	6.69	4.82	4.67	5.57	8.24	6.46	0.590885464	0.22
C1_05170C_A	176.49	151.93	135.30	188.83	193.23	198.08	0.247122951	0.21
C2_05680W_A	7.77	5.92	6.43	8.27	8.47	8.45	0.308788378	0.21
C1_04600C_A	17.34	10.96	12.26	15.53	17.42	17.65	0.48052134	0.21
CR_03730C_A	22.53	29.53	22.35	31.02	30.03	32.53	0.439786912	0.21
C4_00370W_A	14.28	12.23	8.41	16.55	12.45	14.65	0.550051427	0.21
C1_09800C_A	13.41	11.92	18.41	16.46	18.42	19.86	0.552223779	0.21
C1_09460W_A	16.38	17.35	16.54	22.69	20.05	20.20	0.322358243	0.21
C1_13930W_A	34.94	30.90	33.07	41.50	42.71	39.27	0.218968623	0.21
C7_01700W_A	46.83	45.56	57.78	57.73	53.42	77.59	0.479763296	0.21
C2_07550W_A	179.37	140.24	164.98	195.08	216.73	191.65	0.313042142	0.21
C5_02980C_A	1.90	2.99	0.51	1.41	3.11	2.28	0.844125341	0.21
C6_00670W_A	11.64	12.02	12.36	15.40	14.36	15.27	0.281776429	0.21
C5_02390C_A	22.99	17.84	22.03	27.14	20.80	30.74	0.462481012	0.21

C5_04860C_A	3.30	2.01	1.81	2.54	2.61	3.83	0.751627466	0.21
C4_04480C_A	533.18	463.45	493.20	574.01	653.58	630.24	0.206970846	0.21
CR_10670W_A	109.51	112.53	96.66	137.28	132.02	128.65	0.273740453	0.21
C3_07130W_A	11.45	6.28	7.42	6.21	12.38	12.62	0.724286669	0.21
C2_04780W_A	15.38	6.28	4.53	10.29	11.51	10.31	0.74781763	0.21
C3_02190C_A	63.14	76.39	80.76	103.48	81.07	91.50	0.471438328	0.21
C4_02300W_A	8.75	9.18	8.06	10.96	9.63	11.89	0.34900861	0.21
C2_04130W_A	26.02	17.47	19.30	24.79	28.53	24.46	0.45741864	0.21
CR_09670C_A	36.38	42.96	36.09	58.29	38.85	47.30	0.515302795	0.21
CR_04340W_A	9.83	13.34	10.01	11.82	14.32	15.45	0.520937717	0.21
C6_01550C_A	1.52	1.62	1.28	2.08	1.91	1.52	0.568248372	0.21
CR_08190W_A	9.40	4.55	9.65	12.11	3.81	13.67	0.796581984	0.21
C1_10890C_A	45.78	51.53	57.68	65.84	63.87	63.92	0.393785723	0.21
C5_04170W_A	30.87	32.56	31.06	35.78	40.52	41.64	0.290983636	0.21
C3_00380C_A	4.48	3.83	4.36	5.74	4.42	5.62	0.440900619	0.21
CR_05910W_A	35.16	34.10	42.71	62.24	45.65	31.23	0.657985719	0.21
C2_09960W_A	15.80	6.99	7.50	6.77	16.54	13.96	0.757441424	0.21
C1_00710C_A	37.81	72.94	62.44	86.00	63.93	67.98	0.658006668	0.21
C2_06520C_A	3.55	3.83	2.65	4.02	3.42	5.13	0.635761722	0.20
C2_10670W_A	216.64	194.09	189.66	251.72	266.39	227.14	0.30370256	0.20
C4_00840W_A	25.98	25.58	14.29	34.14	23.07	24.58	0.647500881	0.20
C3_03330C_A	14.77	12.75	11.00	13.60	15.15	19.18	0.465252378	0.20
C7_00820W_A	3.90	2.46	2.53	0.02	5.65	5.39	0.904717692	0.20
C1_12660W_A	2.21	0.58	0.32	0.72	1.47	1.61	0.855017206	0.20
CR_00210W_A	8.14	8.40	9.49	11.36	9.33	11.66	0.439615323	0.20
C2_08370C_A	2430.78	2068.01	2173.35	2987.65	3225.87	2007.02	0.563633883	0.20
C1_11540C_A	47.07	38.02	39.33	52.67	58.36	42.31	0.475865755	0.20
C4_04450C_A	18.52	18.13	18.69	15.29	28.43	24.90	0.610598916	0.20
C1_13760W_A	6.00	6.49	5.77	7.93	7.06	7.67	0.411018688	0.20
C3_06070C_A	16.11	11.35	13.73	5.94	22.25	22.81	0.782570019	0.20
C7_02690C_A	4.79	2.17	2.77	3.16	4.79	4.02	0.718831626	0.20
CR_01570W_A	6.95	5.47	3.60	5.18	6.19	8.50	0.704314315	0.20
C4_03810W_A	8.86	5.91	4.72	7.18	7.46	9.39	0.617289506	0.20
C6_03650C_A	14.58	13.96	15.62	19.93	16.38	18.41	0.37129703	0.20
C5_00320W_A	35.21	44.81	49.92	46.04	43.31	72.96	0.645266965	0.20
C4_00730C_A	1.31	2.30	2.30	2.60	2.70	1.96	0.798067283	0.20
C2_01130W_A	16.70	11.90	9.06	14.82	15.01	16.49	0.580087558	0.19
C5_05380W_A	26.94	49.47	48.46	48.20	56.28	51.29	0.663690131	0.19
C1_12650C_A	18.01	9.65	7.02	8.22	14.31	20.10	0.779154959	0.19
C7_03130C_A	4.75	11.29	8.85	11.06	8.65	11.38	0.745858096	0.19
C7_01610W_A	62.76	41.06	90.80	78.30	74.22	87.92	0.67672542	0.19
C1_12730W_A	5.87	4.25	5.53	5.46	6.71	7.11	0.563349409	0.19
CR_05490W_A	3.57	6.13	4.59	6.50	5.26	6.01	0.67163997	0.19
C2_03430W_A	29.14	25.50	26.07	36.34	31.59	31.71	0.342733219	0.19
C1_13550C_A	22.79	21.35	18.88	21.20	30.88	25.58	0.521216294	0.19
C3_00250C_A	34.17	21.85	25.33	29.09	26.70	44.56	0.615805655	0.19
CR_02540W_A	3.61	1.09	1.65	2.34	1.89	3.53	0.78660361	0.19
C2_02730W_A	44.20	60.60	51.72	89.96	75.88	26.38	0.779871131	0.19
C5_00200C_A	42.47	45.79	39.87	49.41	54.59	54.15	0.342186938	0.19
C2_01420C_A	2.77	2.70	2.53	3.11	3.60	3.12	0.544637855	0.19
C1_10070C_A	12.28	9.29	11.46	12.93	12.32	15.41	0.431718971	0.19
C2_08500W_A	27.08	24.70	26.11	38.66	36.72	19.92	0.670812664	0.19
C2_05200C_A	18.34	10.89	12.56	15.75	20.61	14.61	0.631061779	0.19
C1_05700W_A	158.46	152.59	152.18	164.23	188.28	218.85	0.378643092	0.19
C2_04790C_A	4.28	2.96	2.76	3.85	4.15	4.32	0.621074847	0.19
CR_00850C_A	3.41	4.49	4.09	4.29	5.08	5.45	0.543978908	0.19
C2_08470C_A	0.47	0.36	0.24	0.51	0.43	0.37	0.813296133	0.19
CR_02880W_A	9.21	10.01	6.25	8.37	11.50	11.42	0.67889888	0.19
C4_03370C_A	20.01	18.40	19.34	19.63	23.71	27.73	0.510184124	0.19
C6_00910C_A	52.13	55.63	50.70	66.63	59.56	69.17	0.31127565	0.19
C1_14140C_A	29.18	19.72	22.09	24.83	34.00	27.85	0.551297707	0.19
C1_13770C_A	5.26	5.99	8.13	7.44	6.85	9.61	0.667549256	0.19
C4_02060C_A	59.13	44.80	35.42	44.44	57.29	69.15	0.613733736	0.19

C3_04540C_A	4.55	4.95	6.39	11.63	1.79	6.26	0.835134303	0.19
C1_11000C_A	4.10	3.86	5.19	7.41	3.72	5.11	0.752941706	0.19
C4_05670W_A	106.46	99.82	107.30	134.12	123.31	127.61	0.235885064	0.18
C6_00890W_A	21.04	21.40	17.75	20.00	24.59	29.49	0.519793626	0.18
C4_00740W_A	4.84	5.94	7.34	7.09	7.71	7.46	0.671336763	0.18
C7_02990W_A	5.75	5.23	3.96	6.75	5.95	5.60	0.540473363	0.18
C1_02830W_A	8.30	5.98	5.75	5.82	9.37	9.32	0.646308265	0.18
C1_13810W_A	44.94	40.75	35.60	58.24	31.51	59.62	0.649850257	0.18
C4_05060W_A	7.85	6.70	6.09	8.25	7.91	9.12	0.43013635	0.18
CR_04000W_A	80.18	86.33	81.49	113.39	93.02	97.64	0.418794988	0.18
C5_02140C_A	27.56	56.48	59.84	69.51	49.32	59.24	0.740801425	0.18
C5_02210W_A	26.20	30.82	34.12	43.78	29.87	38.34	0.593007731	0.18
C6_00260W_A	63.33	28.06	63.88	56.14	70.17	61.89	0.719789597	0.18
C6_02590C_A	33.16	35.93	29.84	41.45	40.93	38.61	0.442340616	0.18
C2_04560W_A	1.77	2.23	3.03	2.56	2.61	3.43	0.718211851	0.18
C4_05310W_A	0.83	0.18	0.31	0.34	0.60	0.68	0.872320584	0.18
C1_10230C_A	18.73	9.36	9.97	16.82	15.62	13.45	0.687035203	0.18
C1_11800C_A	1.79	1.75	1.76	0.53	2.33	3.68	0.872805746	0.18
C6_02720C_A	8.52	8.44	8.87	8.71	9.43	13.51	0.572780735	0.18
CR_06160C_A	10.22	5.21	8.56	7.80	9.39	11.92	0.732364392	0.18
C1_05430W_A	7.84	8.73	5.92	8.88	10.25	8.25	0.615471268	0.17
CR_04180C_A	38.64	42.41	29.24	46.46	43.82	44.16	0.54355225	0.17
C1_13280C_A	16.99	21.54	25.47	28.81	24.69	24.79	0.632468027	0.17
C4_04460C_A	11.05	9.73	8.25	11.03	12.05	12.22	0.44372551	0.17
C1_00940W_A	11.59	5.23	7.98	10.98	10.59	8.37	0.752942942	0.17
C5_00290W_A	12.05	14.46	14.68	17.90	17.03	15.34	0.557653351	0.17
C2_01330C_A	2.30	1.18	1.54	1.37	2.12	2.59	0.767677371	0.17
C1_00980W_A	15.92	24.67	36.01	31.37	33.12	29.19	0.752942942	0.17
C1_02100W_A	2.69	1.52	1.89	2.56	1.91	2.90	0.716709312	0.17
C2_02760W_A	12.05	11.33	13.95	18.24	15.38	11.69	0.629718886	0.17
CR_07680C_A	2.22	1.37	1.21	1.70	2.12	1.99	0.711948588	0.17
C6_02350C_A	2.07	1.20	1.07	1.80	1.98	1.48	0.752941706	0.17
CR_07320C_A	12.56	11.21	2.58	12.06	9.86	9.94	0.834477486	0.17
C7_00890C_A	64.18	47.32	51.66	62.32	71.18	63.99	0.473464775	0.17
C3_06440W_A	86.11	60.62	55.50	79.89	81.66	82.94	0.535870437	0.17
C3_07920W_A	35.26	31.52	30.78	44.91	38.58	34.83	0.481681189	0.17
C3_03750C_A	9.55	8.50	8.84	10.98	10.28	11.41	0.348935452	0.17
C3_03930W_A	39.76	40.94	37.77	54.09	34.43	56.15	0.610833681	0.17
C3_06640W_A	3.16	2.58	3.05	4.02	3.40	3.28	0.67889888	0.17
C4_07070W_A	22.69	23.92	23.41	27.89	28.50	28.59	0.43310073	0.17
C3_06670C_A	2.14	1.53	1.63	2.02	3.11	1.28	0.806876802	0.17
C1_02710W_A	55.41	64.86	78.96	116.13	60.04	66.31	0.732506877	0.17
CR_04630C_A	6.54	5.34	4.05	6.30	5.90	7.09	0.642028971	0.17
CR_01560W_A	6.22	1.91	0.89	2.44	3.30	5.06	0.87755147	0.17
C2_03100W_A	522.49	562.36	604.53	690.08	704.02	656.53	0.440507663	0.17
C4_01880W_A	28.17	22.54	13.14	19.16	21.94	36.33	0.757866375	0.17
CR_06620W_A	19.15	19.81	19.73	17.87	20.82	32.92	0.669381583	0.17
C2_07950W_A	35.53	28.30	38.58	28.45	48.19	47.28	0.678615239	0.17
CR_06790C_A	1.73	2.08	3.09	3.09	2.83	2.46	0.741183013	0.17
CR_08460W_A	443.70	447.30	428.68	535.39	506.17	559.08	0.283919933	0.16
C2_09350W_A	1.64	1.07	1.74	1.72	1.88	1.76	0.743104654	0.16
C3_04130W_A	29.69	27.37	25.69	36.47	32.28	31.32	0.446512782	0.16
C1_12840W_A	2.46	0.73	1.97	2.36	1.16	2.75	0.872120032	0.16
C3_02670W_A	3.69	3.09	3.63	3.53	5.69	3.32	0.754578325	0.16
C6_00900C_A	12.82	12.26	8.66	5.94	17.54	17.34	0.806876802	0.16
C2_10220C_A	12.92	9.84	12.70	14.32	15.12	13.30	0.538778466	0.16
C2_09910C_A	8.35	4.38	4.34	6.49	7.41	6.66	0.813067902	0.16
C4_05370W_A	2.33	2.89	3.53	2.59	3.09	5.01	0.748413394	0.16
C7_03340C_A	3.15	3.69	3.03	4.33	3.01	4.61	0.707725084	0.16
C1_02950W_A	0.47	9.75	7.40	8.08	7.48	6.24	0.9046316	0.16
C4_03730C_A	2.19	2.15	1.95	1.82	2.70	3.08	0.720266626	0.16
C5_03550W_A	39.76	31.66	34.11	42.90	42.50	41.76	0.371125895	0.16
C1_08860C_A	11.34	14.54	10.11	13.93	15.74	13.91	0.622854944	0.16

C7_03330C_A	54.72	64.11	42.86	70.57	67.43	57.32	0.631431389	0.16
CR_04520W_A	16.87	25.35	32.12	36.94	27.04	26.27	0.751441429	0.16
C5_03560W_A	26.35	24.40	25.34	32.84	30.30	28.67	0.485202604	0.16
C3_02840W_A	2.58	1.50	2.59	2.40	3.26	2.30	0.796581984	0.16
C4_02360W_A	22.24	19.17	19.12	22.72	22.09	28.29	0.445096043	0.16
C2_10190C_A	11.72	9.13	9.67	10.47	13.28	12.99	0.590489434	0.16
CR_05950C_A	8.29	19.25	30.48	25.27	21.12	24.63	0.819789418	0.16
C2_04690C_A	2.29	0.45	1.64	2.04	1.50	1.68	0.854166032	0.16
C7_01820C_A	58.02	52.76	62.67	70.06	67.43	71.57	0.431430665	0.16
C6_00560W_A	5.71	5.32	6.22	6.00	7.08	7.69	0.557644813	0.16
C1_12960C_A	14.75	12.27	13.63	13.97	17.25	17.70	0.485692114	0.16
C1_02430C_A	8.38	7.32	7.53	9.42	9.91	8.59	0.502164061	0.16
C7_04300W_A	94.94	40.21	40.28	65.69	73.25	69.01	0.764963587	0.16
CR_08430W_A	30.55	25.54	25.53	30.72	33.36	33.98	0.385330716	0.16
C2_01850W_A	51.83	43.12	49.00	59.88	59.99	52.85	0.467451309	0.16
C1_06510C_A	5.69	9.19	10.57	10.73	9.19	10.96	0.736368168	0.16
C2_10310C_A	1.83	1.38	1.28	2.00	1.61	1.80	0.69729299	0.16
C2_04720C_A	9.73	2.07	11.93	10.51	9.09	8.60	0.857917177	0.16
C1_02890C_A	40.52	8.51	15.96	34.71	19.10	22.70	0.855137055	0.15
CR_00780C_A	0.56	535.82	663.74	554.68	442.26	483.65	0.956825224	0.15
C2_07460W_A	63.06	47.55	43.03	51.26	61.30	71.72	0.612444807	0.15
CR_04360C_A	17.12	24.82	25.33	22.89	30.94	27.29	0.709435914	0.15
C2_05780C_A	41.99	43.98	41.74	53.92	46.29	53.56	0.437220886	0.15
C5_01180W_A	25.95	19.06	20.98	28.93	24.93	25.09	0.548333356	0.15
CR_04140W_A	2.60	21.59	12.55	16.37	15.81	12.86	0.881093124	0.15
C5_01420W_A	88.80	129.97	131.34	146.72	130.08	146.12	0.671621159	0.15
C1_04140W_A	5.32	6.42	6.91	8.17	6.52	7.74	0.665913513	0.15
C3_00770C_A	109.65	100.40	134.25	115.99	160.54	135.83	0.642536517	0.15
C1_02160W_A	14.89	14.60	8.97	13.76	14.26	18.22	0.723555571	0.15
C3_00730W_A	10.48	5.12	6.73	8.52	10.03	7.89	0.738430119	0.15
C5_03140C_A	19.79	15.62	18.36	22.32	23.39	18.39	0.576666041	0.15
C6_00200C_A	123.79	103.50	142.01	151.67	153.97	135.66	0.592289418	0.15
CR_07070C_A	11.25	13.65	11.49	14.16	14.67	14.89	0.550163518	0.15
CR_05610C_A	16.46	13.17	13.52	10.70	19.33	21.58	0.748214001	0.15
C2_01360C_A	46.94	52.73	44.83	48.63	72.49	51.59	0.676189237	0.15
C1_00650C_A	106.85	177.45	94.60	165.76	134.33	157.02	0.748413394	0.15
C2_01160W_A	6.39	5.33	6.03	6.69	6.55	7.96	0.581482155	0.15
C1_13700W_A	378.64	613.39	624.59	763.37	593.43	590.93	0.7331607	0.15
C1_05630C_A	10.45	10.37	14.78	19.41	9.20	14.13	0.767677371	0.15
C3_07040C_A	11.14	14.04	10.26	17.05	11.17	14.30	0.714219629	0.15
C7_03030W_A	40.59	51.99	18.56	37.44	35.82	60.69	0.820539909	0.15
C6_00120W_A	22.28	16.87	18.36	16.00	27.33	25.25	0.743475368	0.15
CR_04390C_A	16.14	9.57	9.36	15.04	18.71	7.44	0.814782726	0.15
C6_00110C_A	21.62	14.14	15.79	19.60	21.76	19.86	0.641853429	0.15
CR_07360W_A	7.14	7.68	6.86	8.81	8.87	8.24	0.536830985	0.14
C2_02000W_A	32.75	39.99	37.92	45.12	42.73	44.75	0.566948619	0.14
C7_02460C_A	8.90	6.86	6.55	10.35	7.42	8.84	0.663719429	0.14
C4_03560W_A	8.97	8.92	10.15	9.52	10.55	13.52	0.619890586	0.14
C1_12620W_A	12.58	10.27	10.01	14.18	11.95	13.03	0.512056106	0.14
C1_10560C_A	6.08	3.71	5.02	5.75	5.63	6.29	0.667141972	0.14
C5_04730C_A	37.59	15.19	29.92	36.77	29.73	31.10	0.772367216	0.14
CR_08030C_A	47.04	38.02	35.92	44.80	52.12	46.76	0.547816123	0.14
C1_08200W_A	6.67	14.57	12.57	11.11	17.36	12.08	0.813296133	0.14
C6_02740W_A	87.64	111.17	122.91	141.68	107.60	136.65	0.677780224	0.14
C3_07430W_A	32.23	35.24	35.03	39.55	39.28	43.62	0.505573194	0.14
C2_04800C_A	6.89	4.00	3.76	5.50	5.53	6.36	0.74357754	0.14
C3_07050W_A	14.92	7.71	6.56	7.95	9.10	17.75	0.842077386	0.14
C2_05270W_A	1.22	1.51	1.40	1.46	1.73	1.72	0.708064998	0.14
C4_00780C_A	8.50	6.03	5.13	8.89	7.36	7.05	0.698345248	0.14
C7_01320W_A	10.21	7.02	6.38	9.14	8.66	10.23	0.669381583	0.14
C1_08600C_A	21.95	22.26	21.75	26.14	25.42	27.08	0.41125492	0.14
CR_01760C_A	62.41	61.61	67.35	83.78	68.91	75.48	0.514970571	0.14
C2_04960C_A	12.30	9.88	10.50	13.92	11.10	13.90	0.66608856	0.14

CR_03000C_A	47.99	49.87	56.40	60.21	66.55	56.91	0.599690433	0.14
C1_10060C_A	4.44	5.93	6.08	4.55	6.33	8.70	0.79846516	0.14
C4_04980W_A	4.65	3.07	3.47	4.16	4.31	4.82	0.677622674	0.14
C5_01580C_A	116.26	118.32	139.87	163.54	143.17	139.16	0.583210979	0.14
C6_00850W_A	89.93	121.30	135.09	148.66	117.48	148.73	0.701910171	0.14
C5_02170C_A	56.20	54.21	52.32	63.86	66.39	63.12	0.422821575	0.14
C1_07350C_A	42.85	49.08	55.20	57.21	60.87	57.18	0.625686301	0.14
C2_02250C_A	16.11	11.76	12.33	15.00	16.53	16.11	0.596027811	0.14
C5_00040C_A	30.33	28.47	13.00	27.49	30.46	26.94	0.789743703	0.14
C2_09690C_A	7.89	7.56	6.31	7.24	9.52	9.06	0.647763716	0.14
C1_01540W_A	37.90	33.80	25.52	40.80	30.10	44.69	0.68992138	0.14
CR_01520W_A	25.95	18.59	21.29	21.64	29.49	26.69	0.652230162	0.14
CR_09380W_A	48.07	60.31	57.96	70.29	57.52	70.80	0.638295276	0.14
C1_08800W_A	23.07	16.82	15.67	20.08	25.39	20.05	0.68093953	0.14
C5_00240W_A	17.71	9.84	15.35	16.17	17.25	17.20	0.708243852	0.14
C7_00790W_A	6.26	4.25	3.66	2.72	6.88	7.12	0.83242468	0.14
C1_07090C_A	11.21	7.87	9.10	11.25	10.45	11.64	0.592238029	0.14
C7_02870C_A	12.21	15.43	13.74	18.69	16.20	14.38	0.714086619	0.14
C1_06650W_A	91.78	55.60	30.78	70.93	25.66	115.02	0.877288549	0.13
C1_11150W_A	35.32	33.60	29.75	36.42	37.32	43.37	0.543069585	0.13
CR_00010C_A	3.49	2.75	3.28	3.43	4.43	3.39	0.703811255	0.13
C3_04950W_A	126.61	178.55	217.77	252.91	160.53	211.81	0.766007755	0.13
C4_05830W_A	3.24	2.61	1.17	3.78	1.83	2.53	0.885568554	0.13
C2_04620W_A	57.79	30.80	29.56	44.50	43.52	50.74	0.751777226	0.13
C7_00430W_A	23.80	0.00	27.61	12.51	24.98	22.49	0.969787127	0.13
C2_00440W_A	3.62	4.62	4.70	11.66	0.58	3.26	0.920573208	0.13
C4_00820W_A	2.03	1.95	0.94	2.82	1.24	1.82	0.860924675	0.13
CR_02990C_A	64.48	61.31	61.82	66.32	73.35	82.85	0.500420906	0.13
C1_11620W_A	1.81	1.95	3.85	2.67	4.14	2.17	0.859476817	0.13
C2_05040C_A	33.02	34.08	33.48	39.07	41.25	38.80	0.522542642	0.13
C2_06910W_A	20.91	32.54	31.67	36.73	24.76	40.40	0.771518985	0.13
C7_03490W_A	42.94	30.11	25.90	32.26	38.04	46.44	0.714219629	0.13
C6_03150C_A	18.39	19.75	19.43	21.23	22.26	24.85	0.552298185	0.13
C1_02660C_A	17.18	23.51	23.50	29.40	24.36	22.55	0.726299669	0.13
C1_07860W_A	0.93	0.52	0.45	0.47	0.69	1.07	0.87868889	0.13
C7_01250W_A	386.99	356.48	451.98	495.32	472.74	446.57	0.586220229	0.13
C2_03000C_A	1.46	1.52	1.12	1.75	0.92	2.26	0.85001603	0.13
C3_04250W_A	4.63	6.24	6.35	6.79	6.27	7.40	0.726499511	0.13
C3_04110C_A	36.46	32.40	30.30	38.51	38.62	40.09	0.443137965	0.13
C6_01610W_A	642.34	664.59	868.47	957.83	838.93	781.05	0.676189237	0.13
CR_05980W_A	18.31	14.57	13.91	17.12	18.42	19.69	0.553363321	0.13
CR_02160W_A	13.76	10.04	10.73	13.86	12.82	14.06	0.563780419	0.13
C2_08680W_A	3.33	1.14	1.60	1.36	2.19	3.59	0.866262942	0.13
C2_00490W_A	22.81	21.01	23.95	24.13	27.16	28.85	0.53719618	0.13
C7_03530C_A	3.71	4.14	2.46	3.30	4.80	4.07	0.803575596	0.13
CR_00990W_A	11.81	4.80	16.09	11.39	12.13	15.05	0.851659945	0.13
C3_00260C_A	74.30	63.78	68.11	65.77	72.06	106.59	0.692043572	0.13
CR_08330W_A	14.08	12.14	9.50	12.71	13.89	15.64	0.698345248	0.13
C1_10770W_A	15.64	16.62	20.45	23.09	17.04	22.35	0.711082764	0.13
C4_03190W_A	12.18	11.40	12.70	13.61	14.68	14.53	0.556539361	0.13
C6_03560W_A	7.03	13.51	15.53	17.41	9.57	16.25	0.832462269	0.13
C5_03280W_A	41.56	28.04	27.84	36.40	35.76	42.39	0.667965964	0.13
C1_10120C_A	49.38	49.12	50.30	55.24	52.70	68.25	0.570159295	0.13
CR_08040W_A	41.35	44.68	45.28	52.61	50.54	52.15	0.534319246	0.13
C4_05090C_A	3.15	2.78	1.97	2.22	3.46	3.64	0.771070756	0.13
C2_04980C_A	5.07	4.12	4.35	3.70	5.73	6.59	0.7739042	0.13
C7_02180C_A	11.15	18.68	42.17	19.24	27.65	39.41	0.877143463	0.13
C5_02820C_A	11.85	10.11	10.46	10.15	15.53	12.44	0.726792932	0.13
C4_05740C_A	13.41	14.76	5.55	16.11	11.79	11.77	0.834289815	0.13
CR_04920W_A	5.90	4.49	4.38	5.08	5.79	6.49	0.711870401	0.13
C3_00780W_A	4.67	6.63	4.73	7.22	5.76	5.96	0.759245882	0.13
C2_03600W_A	7.16	9.59	7.94	10.75	9.09	9.34	0.70604077	0.12
C1_14270W_A	5.95	7.71	8.88	9.69	8.76	8.13	0.761067222	0.12

C4_02420C_A	3.62	2.72	4.77	3.57	4.50	4.94	0.805379879	0.12
C3_07710W_A	30.92	28.25	25.76	34.92	33.07	31.80	0.556539361	0.12
C5_01760C_A	23.80	19.34	19.32	26.79	22.79	23.73	0.581482155	0.12
C1_04070C_A	1.53	0.85	0.89	1.27	1.40	1.18	0.846990048	0.12
C1_01760W_A	6.62	5.53	5.01	6.22	6.29	7.65	0.65917134	0.12
C6_03530C_A	7.35	4.95	5.66	7.10	5.89	8.14	0.788519164	0.12
CR_10730C_A	23.02	23.79	21.03	32.16	22.59	25.06	0.698521005	0.12
C1_14100W_A	37.69	59.76	60.24	68.23	54.49	64.04	0.770151213	0.12
C1_11240C_A	35.86	27.75	33.46	32.59	39.67	41.53	0.647500881	0.12
C1_04420C_A	46.66	53.08	50.91	63.49	51.61	62.46	0.635356797	0.12
C6_00830C_A	52.13	79.03	72.40	84.79	78.05	77.61	0.740830134	0.12
CR_01610C_A	82.58	65.86	66.05	84.53	81.39	85.16	0.540473363	0.12
C1_03670C_A	36.15	28.51	41.98	45.50	38.83	40.65	0.697322984	0.12
C2_06570C_A	471.98	632.80	971.87	984.31	606.31	869.11	0.821579646	0.12
C2_04100W_A	10.19	9.42	6.38	8.95	9.15	12.44	0.766240243	0.12
CR_00900W_A	0.67	0.52	0.60	1.45	0.45	0.20	0.922089614	0.12
C5_00080C_A	82.39	85.43	44.43	122.38	3.91	125.47	0.951643138	0.12
C3_03780W_A	15.09	8.12	18.09	7.62	12.64	28.40	0.888521365	0.12
CR_08350W_A	23.12	31.63	30.39	33.55	32.64	34.08	0.712921372	0.12
CR_09300C_A	54.28	3.13	45.24	22.88	46.89	48.38	0.925524443	0.12
C7_00770W_A	4.80	2.50	2.34	2.35	4.73	4.16	0.862082597	0.12
C6_02370C_A	2.51	0.53	1.19	1.17	1.88	1.80	0.898947606	0.12
C1_03730C_A	20.49	20.87	17.36	19.83	25.66	23.20	0.679917023	0.12
C4_02570C_A	6.04	29.57	29.95	20.05	16.92	42.05	0.90547257	0.12
C5_04600C_A	32.06	36.98	31.13	38.66	41.55	37.09	0.654985447	0.11
C4_02520C_A	5.50	5.67	5.61	7.80	5.43	6.47	0.732552136	0.11
C5_02230W_A	89.12	131.98	127.91	146.03	125.90	139.18	0.751777226	0.11
C7_01580W_A	5.30	4.58	4.23	6.01	5.08	5.38	0.653230549	0.11
C4_04350W_A	52.71	46.56	50.61	58.03	58.13	58.81	0.526623299	0.11
C3_05560W_A	40.94	41.55	48.25	59.67	39.03	54.60	0.740203165	0.11
C3_03380W_A	3.69	2.30	1.44	2.05	3.33	3.23	0.85530719	0.11
CR_00120C_A	21.54	21.78	3.01	36.63	4.11	13.21	0.932915436	0.11
C5_00950C_A	15.38	15.60	17.32	22.92	19.62	13.77	0.776048888	0.11
C2_10540W_A	30.55	18.18	23.22	30.03	29.22	24.17	0.760099213	0.11
C6_04080W_A	33.39	32.53	32.42	41.80	32.01	41.24	0.643230672	0.11
CR_05260C_A	9.76	7.80	5.96	9.56	7.88	10.01	0.765108924	0.11
C7_01220W_A	20.50	21.43	22.94	23.56	25.66	26.56	0.637451473	0.11
C1_01690C_A	420.89	259.01	311.53	462.70	347.17	337.95	0.759793618	0.11
C1_08080C_A	149.11	118.38	193.00	251.41	191.82	89.61	0.8651394	0.11
C3_07480W_A	22.16	22.72	24.46	28.54	27.23	25.14	0.647405468	0.11
C7_02570C_A	133.31	124.09	116.21	132.34	137.97	165.70	0.611420868	0.11
C1_04670W_A	8.01	6.50	6.88	11.16	2.83	11.09	0.888629845	0.11
C5_01260W_A	0.42	0.22	0.16	0.32	0.23	0.38	0.906275251	0.11
CR_02050C_A	12.41	10.94	10.06	12.92	13.24	12.71	0.611216576	0.11
C3_02790W_A	18.59	19.37	32.40	33.89	23.49	24.81	0.833788509	0.11
C1_09440W_A	12.25	15.49	17.20	18.08	15.44	19.10	0.755099733	0.11
CR_08410W_A	26.59	18.79	20.19	25.77	25.45	24.83	0.6775621	0.11
C2_06150C_A	139.03	41.02	76.95	108.40	67.94	119.46	0.872320584	0.11
C7_02780W_A	47.24	90.10	54.38	105.43	56.45	63.47	0.853890695	0.11
C3_01890C_A	130.82	46.16	110.19	105.46	109.08	116.09	0.85001603	0.11
CR_00860C_A	506.51	544.89	688.96	699.80	702.12	626.14	0.72922184	0.11
C2_07830W_A	4.35	6.99	4.62	7.78	5.92	4.97	0.831903853	0.11
CR_01770C_A	18.68	18.61	16.25	20.64	20.72	20.89	0.596869616	0.11
C6_02440C_A	1.52	2.18	2.39	1.99	2.33	2.75	0.855137055	0.11
C5_01830C_A	8.86	7.64	8.35	8.79	9.01	11.09	0.648447947	0.11
C4_05620C_A	13.14	22.56	15.09	20.62	16.78	22.01	0.825781331	0.11
C3_05920W_A	3.41	2.95	2.08	2.63	3.48	3.68	0.797992492	0.10
C5_04670W_A	29.96	21.75	22.53	29.66	28.40	27.80	0.682728271	0.10
CR_06730W_A	12.54	12.70	12.27	14.59	14.82	14.16	0.622492435	0.10
C4_04870C_A	59.16	47.44	43.78	53.00	60.37	60.64	0.669381583	0.10
C6_02830W_A	0.48	0.22	0.34	0.33	0.46	0.41	0.893054324	0.10
C4_07040W_A	1.88	2.68	2.74	3.38	2.74	2.41	0.833788509	0.10
C5_01410C_A	4.88	3.21	3.65	3.42	4.40	5.76	0.804599789	0.10

C1_06110C_A	4.48	6.54	6.41	5.31	6.48	8.59	0.821539034	0.10
C1_02790W_A	75.04	82.31	91.15	99.71	92.22	97.20	0.674077504	0.10
C2_01560W_A	42.00	42.40	46.84	48.71	48.49	55.19	0.630860893	0.10
C1_14410W_A	42.39	15.65	127.90	91.31	103.89	17.77	0.939353469	0.10
C5_03100C_A	5.73	14.09	21.93	18.14	13.74	17.15	0.896350919	0.10
C3_02450W_A	31.51	42.60	42.41	44.78	40.61	50.28	0.77612475	0.10
C1_07260C_A	18.96	5.69	15.91	16.62	14.95	14.77	0.879240568	0.10
C1_03780C_A	12.49	8.45	11.79	14.66	13.15	9.79	0.799602197	0.10
C1_11960C_A	24.01	24.57	14.45	22.67	22.69	27.73	0.826534415	0.10
C1_11070W_A	1.80	1.25	1.50	1.94	1.49	1.85	0.811378109	0.10
C5_00310C_A	8.72	7.47	9.23	12.02	9.12	8.21	0.81422741	0.10
C7_00100W_A	8.15	0.04	12.59	8.22	7.85	7.68	0.969787127	0.10
C7_00140C_A	64.72	0.38	78.60	52.37	55.11	56.22	0.968386455	0.10
C6_04560W_A	73.45	87.90	72.81	93.03	87.39	91.10	0.701650737	0.10
C1_08980C_A	30.55	17.44	14.58	14.07	27.49	30.23	0.872320584	0.10
CR_07460C_A	13.97	16.60	14.46	18.67	15.29	18.30	0.726320106	0.10
C1_10620W_A	29.93	11.24	11.50	11.40	26.45	21.95	0.896694656	0.10
C7_04010W_A	22.53	18.45	28.06	19.49	19.84	41.10	0.858983738	0.10
C2_03040W_A	27.36	45.08	41.79	44.65	42.13	46.25	0.816185744	0.10
C1_13400C_A	6.45	5.26	5.03	6.32	6.29	6.71	0.703811255	0.10
C6_03050C_A	4.96	2.62	6.13	4.51	6.19	5.03	0.869506995	0.10
C1_02760W_A	82.51	97.57	99.56	112.54	101.68	109.66	0.714219629	0.10
C2_04220C_A	85.67	131.92	138.08	163.88	124.34	124.63	0.830408389	0.09
C6_04280W_A	53.70	51.06	42.48	61.43	41.01	67.86	0.796767204	0.09
C2_09470C_A	1.52	1.28	1.00	1.41	1.26	1.73	0.829353491	0.09
C1_06600W_A	253.30	194.33	173.44	218.56	220.41	275.55	0.748214001	0.09
CR_05680C_A	39.08	67.60	52.20	65.97	63.32	54.89	0.83502457	0.09
C4_05280W_A	4.28	2.88	2.13	3.67	3.38	3.57	0.829579547	0.09
CR_08120C_A	114.82	59.84	55.16	76.63	105.16	78.96	0.855833064	0.09
C7_00990W_A	635.78	635.34	728.76	821.21	774.00	707.05	0.712121416	0.09
C1_06750W_A	6.15	11.00	17.06	15.45	11.76	12.58	0.89565402	0.09
C1_01550W_A	21.74	25.45	6.89	22.41	15.49	24.57	0.903886772	0.09
C7_03070C_A	15.86	22.03	18.04	22.93	16.71	25.29	0.812285242	0.09
CR_10170C_A	3.08	3.31	4.08	3.94	4.17	3.93	0.80809009	0.09
C6_02070C_A	461.06	516.07	700.74	662.61	663.50	609.55	0.798617719	0.09
C2_01210C_A	9.84	12.52	18.75	12.97	16.73	17.90	0.855137055	0.09
C3_06860C_A	47.47	54.04	68.24	70.27	68.03	57.22	0.802869578	0.09
C3_05450C_A	31.59	18.85	16.15	13.65	28.25	34.19	0.888171734	0.09
C2_02300W_A	5.69	7.19	6.65	9.39	5.61	7.52	0.85001603	0.09
CR_06660W_A	3.14	3.86	6.20	6.32	2.72	6.27	0.897779996	0.09
C2_07160W_A	22.16	23.73	26.03	25.12	25.10	32.78	0.756074251	0.09
C6_02410W_A	25.82	23.66	32.00	30.87	33.74	28.87	0.774693866	0.09
C2_10820C_A	7.39	0.33	6.52	5.54	4.38	6.17	0.946417414	0.09
C2_03450W_A	5.38	6.47	5.89	6.85	5.76	7.85	0.800073398	0.09
CR_00670C_A	0.28	1.46	0.95	0.36	1.54	1.23	0.946325703	0.09
C4_07190W_A	31.96	29.22	30.29	27.16	38.82	39.01	0.772976554	0.09
C1_10320W_A	5.29	1.76	1.84	2.29	3.88	3.83	0.916340993	0.09
C5_03300C_A	7.13	3.35	3.50	4.18	5.72	5.98	0.875565652	0.09
C4_04150C_A	118.40	148.03	151.44	228.03	123.50	129.23	0.859969639	0.09
C2_04240C_A	20.95	18.76	22.42	24.25	22.77	24.20	0.707198016	0.09
C4_00060W_A	32.24	25.82	27.10	37.56	33.31	26.36	0.801109063	0.09
C2_07040W_A	5.27	4.61	4.80	5.95	5.66	5.23	0.784410836	0.09
C6_02020C_A	33.30	24.50	23.21	31.94	36.53	23.57	0.833357665	0.09
C2_00370W_A	5.73	4.25	6.66	7.22	4.55	7.32	0.84758131	0.08
C1_03220C_A	649.79	763.34	773.59	905.88	835.53	768.54	0.763641841	0.08
C1_13020C_A	27.53	34.00	41.37	46.89	34.61	36.74	0.836301341	0.08
C1_07680W_A	15.08	9.94	8.65	14.16	12.33	11.82	0.835914261	0.08
C2_01680C_A	25.20	19.90	17.45	29.01	18.47	23.97	0.821609352	0.08
C2_00300C_A	28.45	27.19	26.50	36.84	33.79	23.07	0.829772912	0.08
C4_03590C_A	15.89	14.46	20.63	24.13	15.78	18.51	0.833633141	0.08
CR_10710C_A	12.84	12.89	16.22	17.14	15.39	15.52	0.777996892	0.08
CR_07700W_A	6.86	4.65	5.54	7.09	7.58	4.68	0.859430483	0.08
CR_04540C_A	7.57	6.84	7.66	8.37	8.52	8.24	0.822703418	0.08

C2_07510W_A	14.65	13.64	11.52	18.50	11.16	16.01	0.834755676	0.08
C6_01760W_A	25.81	27.61	25.33	31.36	30.14	28.66	0.712493422	0.08
C2_05060C_A	40.63	48.37	38.09	42.98	47.85	55.03	0.792014557	0.08
C2_00480C_A	22.39	37.84	34.72	21.42	43.49	44.66	0.893857522	0.08
C3_05270C_A	10.92	4.57	6.73	6.99	9.30	8.84	0.877421969	0.08
C4_02160C_A	4.24	5.07	3.60	4.62	4.57	5.61	0.819789418	0.08
C6_04240W_A	1.60	1.25	0.39	0.93	1.35	1.47	0.941950349	0.08
C2_04680W_A	22.05	17.84	25.53	29.43	22.13	23.14	0.83216027	0.08
C1_01200W_A	12.83	8.13	13.35	11.98	14.82	12.14	0.841295527	0.08
C1_13680C_A	76.62	103.00	123.84	152.52	92.14	103.62	0.871967889	0.08
C7_02370W_A	26.47	74.15	75.53	24.45	81.00	99.39	0.934316527	0.08
CR_09560C_A	2.65	1.60	3.15	3.24	2.89	2.27	0.890578878	0.08
C5_02510C_A	23.84	21.96	21.61	18.42	23.24	35.57	0.854261455	0.08
CR_01490C_A	91.65	93.92	87.67	102.77	114.65	93.79	0.772976554	0.08
C1_08360C_A	1936.00	1549.89	1646.82	1954.21	1973.43	1903.61	0.691711369	0.08
C5_00330C_A	4.60	3.05	2.71	3.23	3.74	4.79	0.852707902	0.08
C2_00850W_A	12.49	13.80	17.36	19.23	15.21	15.38	0.835134303	0.08
C2_01570W_A	0.97	2.17	2.31	2.75	2.14	1.30	0.936099688	0.08
C1_13240W_A	22.86	22.54	20.96	22.83	25.15	27.65	0.738006038	0.08
CR_10060W_A	115.52	46.25	49.04	88.99	69.52	77.78	0.897306275	0.08
C1_12490W_A	10.04	20.13	9.65	18.37	13.58	13.80	0.897757675	0.08
C1_08550C_A	127.21	137.01	137.69	171.90	140.04	146.39	0.769046081	0.08
C1_12820C_A	6.73	1.55	3.17	4.44	3.37	5.08	0.932915436	0.07
C1_03900W_A	2.93	0.56	0.15	0.36	2.27	1.47	0.966708209	0.07
C5_04000W_A	5.68	8.05	6.74	8.11	8.48	6.73	0.860924675	0.07
C7_02480W_A	26.27	30.63	28.57	37.06	31.21	29.10	0.821071493	0.07
C3_05140C_A	4.67	5.54	4.79	6.37	4.79	5.94	0.836558745	0.07
C5_01140C_A	4.69	3.92	4.06	4.61	4.14	5.66	0.809328412	0.07
CR_03200C_A	3.38	3.61	3.57	3.50	4.24	4.24	0.813296133	0.07
C5_02500C_A	20.80	18.81	15.62	11.46	24.68	26.65	0.896161594	0.07
C1_05940W_A	32.30	24.51	26.93	29.86	26.94	38.32	0.803654333	0.07
C1_03720C_A	12.82	14.19	14.26	12.33	16.57	18.07	0.833134811	0.07
C3_03260W_A	12.30	13.70	13.14	16.70	9.73	18.31	0.871142903	0.07
C5_02030W_A	54.49	74.57	81.13	89.20	68.19	82.73	0.855584293	0.07
C1_12430W_A	9.03	9.08	7.46	10.84	7.97	10.23	0.820672324	0.07
C5_05460C_A	33.64	33.93	34.82	36.09	43.88	35.97	0.815051944	0.07
C1_01980W_A	7.62	3.97	5.99	5.70	6.61	7.49	0.878019059	0.07
CR_08050C_A	83.29	83.24	92.51	92.49	100.56	100.81	0.754578325	0.07
C7_02790C_A	5.75	11.75	6.05	15.36	4.95	6.64	0.934297253	0.07
C4_01870C_A	29.42	31.99	28.67	34.31	32.60	35.33	0.757441424	0.07
C2_03360W_A	16.16	25.38	22.10	27.70	28.78	15.69	0.901358861	0.07
CR_02860W_A	118.06	103.40	24.90	133.64	14.00	132.70	0.957565316	0.07
CR_04740C_A	16.31	15.43	18.92	20.12	18.77	18.50	0.785877321	0.07
C2_06420C_A	35.63	32.27	33.71	39.11	35.55	40.44	0.697322984	0.07
CR_09950C_A	26.33	15.49	24.97	32.27	20.32	22.66	0.884691851	0.07
C4_06140C_A	3.86	4.66	3.17	5.17	3.47	4.66	0.881704824	0.07
C6_04590C_A	30.96	29.47	25.83	28.63	33.69	35.24	0.798171177	0.07
C3_06210C_A	11.63	9.63	2.91	5.78	10.18	11.25	0.936543184	0.07
CR_04580W_A	32.05	28.21	28.68	31.79	34.91	33.78	0.765108924	0.07
C1_00850W_A	11.17	16.18	12.62	12.00	13.61	19.99	0.889859702	0.07
C2_02330W_A	1.72	1.92	3.70	2.30	1.93	4.17	0.940675182	0.07
C1_08390C_A	20.39	22.79	25.40	27.76	24.52	25.44	0.819789418	0.07
C6_02420W_A	2.01	0.70	0.76	0.68	1.40	1.78	0.947158759	0.07
C2_06100W_A	41.68	40.75	41.31	60.26	24.20	56.25	0.907592919	0.07
C3_04470W_A	39.78	35.94	33.94	43.71	38.10	41.98	0.748813651	0.06
CR_01300W_A	50.53	49.25	52.80	64.07	47.46	60.96	0.830394878	0.06
C1_11010C_A	12.93	14.33	11.59	13.27	15.82	14.79	0.833788509	0.06
C1_06690W_A	21.22	16.73	12.29	17.44	17.43	21.74	0.872428157	0.06
C4_03390W_A	100.76	85.24	91.70	96.87	107.05	109.12	0.751163032	0.06
C1_11330C_A	441.77	547.70	498.06	523.35	573.58	588.62	0.821350749	0.06
C1_13490C_A	0.27	1.24	1.31	0.97	1.08	1.16	0.951605544	0.06
C3_05980C_A	7.29	4.85	7.47	8.31	6.19	7.56	0.872320584	0.06
C1_09620C_A	9.56	5.73	3.63	6.72	6.14	8.41	0.925560258	0.06

C2_09580W_A	14.18	17.60	18.88	20.65	19.10	17.56	0.859675066	0.06
C1_08120W_A	25.65	24.94	16.84	20.32	34.21	21.04	0.902507668	0.06
C1_03380W_A	261.64	248.85	279.20	310.14	267.27	314.09	0.771518985	0.06
C6_00880W_A	18.16	19.03	19.01	15.42	22.88	25.18	0.874222566	0.06
C4_04550C_A	12.80	16.53	18.72	17.38	16.46	20.54	0.877288549	0.06
C2_09040W_A	82.56	64.69	67.93	76.25	77.84	87.73	0.793087671	0.06
C5_05280C_A	6.41	5.88	11.21	10.80	8.25	7.36	0.92408822	0.06
C3_00420W_A	7.99	5.69	6.58	8.39	6.70	7.63	0.850312936	0.06
C3_02160C_A	33.42	27.35	26.18	31.96	35.36	30.03	0.829353491	0.06
C7_01710W_A	15.26	12.23	25.45	16.67	16.43	26.78	0.921073381	0.06
C3_03420C_A	214.59	250.37	240.34	291.62	260.67	241.86	0.84114891	0.06
C2_00760C_A	228.06	332.23	412.00	506.13	142.38	459.53	0.943239977	0.06
CR_05890C_A	28.15	18.13	22.59	18.41	31.63	26.84	0.903183401	0.06
C1_14260C_A	86.59	67.53	62.36	75.20	83.83	83.12	0.836301341	0.06
C1_03300C_A	28.51	27.01	26.85	34.94	29.42	28.00	0.834538704	0.06
CR_05650W_A	23.42	30.60	20.81	30.41	26.87	26.92	0.883655361	0.05
C6_01850W_A	18.21	14.46	14.70	20.09	16.90	15.95	0.853890695	0.05
CR_08020C_A	114.22	67.11	110.74	94.30	117.17	114.48	0.898380512	0.05
C2_00680C_A	6.99	6.05	9.74	9.61	5.92	10.09	0.92229637	0.05
C4_00950C_A	12.91	7.87	10.14	9.87	11.95	12.65	0.887598133	0.05
C6_04300W_A	4.27	1.04	1.01	1.99	2.09	2.88	0.956825224	0.05
C1_03710C_A	4.00	4.50	7.20	4.43	7.72	5.46	0.927747537	0.05
C6_01770W_A	6.76	6.85	6.67	7.75	7.21	7.71	0.853890695	0.05
CR_06830C_A	9.97	9.28	8.02	7.91	10.02	12.58	0.898051452	0.05
C4_00770C_A	37.99	24.90	23.48	30.30	28.56	37.53	0.894322801	0.05
C1_09120W_A	1.59	0.92	1.68	0.99	1.66	2.02	0.941641944	0.05
C1_01040W_A	42.86	49.39	47.00	47.58	54.54	53.88	0.859095661	0.05
C6_01640W_A	29.15	27.49	27.26	32.83	29.93	31.00	0.835134303	0.05
C1_08700W_A	24.80	16.81	17.52	22.07	20.81	22.86	0.869298447	0.05
C1_11250W_A	9.17	50.05	58.05	42.68	56.21	34.02	0.962339103	0.05
C2_01370C_A	10.40	6.61	4.79	6.69	10.48	6.93	0.934316527	0.05
C6_00990W_A	19.44	16.08	18.74	18.82	18.81	23.01	0.854166032	0.05
C4_03070W_A	26.56	23.15	35.23	31.62	34.60	28.48	0.901425208	0.05
C4_04520W_A	7.33	6.00	3.93	5.78	5.79	7.67	0.924765326	0.05
C2_02320C_A	109.68	165.92	170.76	207.61	135.69	158.78	0.920573208	0.05
C3_07940W_A	37.43	32.18	36.31	42.46	39.98	35.49	0.860924675	0.05
CR_03340C_A	13.11	9.33	9.59	10.14	12.67	12.84	0.893054324	0.05
C4_03360C_A	54.14	53.39	56.10	60.82	58.41	63.52	0.803729762	0.05
CR_08660W_A	5.88	1.75	6.10	5.59	7.89	1.47	0.966801513	0.05
C1_08270C_A	30.87	25.97	24.24	30.18	31.13	28.79	0.848990622	0.05
C2_09820W_A	28.73	46.48	28.64	40.37	42.53	33.37	0.92499113	0.04
C4_05760W_A	12.97	17.71	11.07	19.17	15.03	12.40	0.927747537	0.04
C2_05470W_A	29.19	30.00	30.43	35.52	31.18	33.33	0.85221945	0.04
C5_03670C_A	4.84	8.17	7.46	7.81	9.60	5.39	0.942994807	0.04
C2_06890C_A	4.68	6.49	4.63	8.02	3.43	6.27	0.945982341	0.04
C2_09050C_A	6.36	5.57	6.61	5.84	6.42	8.39	0.903165038	0.04
C4_04660C_A	41.09	24.83	17.14	28.38	31.67	31.41	0.936099688	0.04
C6_01620W_A	11.61	9.76	7.82	9.00	11.42	11.97	0.907592919	0.04
C1_10220C_A	11.25	7.36	7.35	10.96	9.24	8.49	0.918951104	0.04
C1_04450C_A	21.22	22.60	25.71	25.93	25.72	25.71	0.885568554	0.04
CR_03130W_A	3.46	2.66	4.09	3.61	2.16	5.62	0.957225171	0.04
C7_01960W_A	21.50	7.84	7.36	18.76	11.04	10.16	0.957565316	0.04
C1_06710W_A	8.13	12.73	16.63	19.34	10.61	12.05	0.948556551	0.04
C6_04030W_A	7.19	6.99	6.73	7.29	6.92	9.06	0.891689213	0.04
C1_06020W_A	10.34	8.74	7.69	10.07	9.72	9.91	0.882383504	0.04
C3_03400C_A	26.17	12.14	17.71	14.09	21.69	26.11	0.944490537	0.04
C5_02160W_A	1.98	1.88	2.24	2.22	2.10	2.42	0.913087806	0.04
C2_03500W_A	29.54	25.75	21.83	32.00	25.08	28.41	0.89391971	0.04
C1_05420W_A	9.30	15.24	14.44	14.79	15.97	12.72	0.939569692	0.04
C4_04360W_A	1.48	1.28	1.46	1.60	1.32	1.75	0.931587179	0.04
CR_01720W_A	5.69	5.46	5.66	6.18	4.94	7.60	0.908122109	0.04
C4_01090C_A	1.52	0.79	1.33	1.24	0.92	1.87	0.959989925	0.04
C4_00320C_A	15.00	16.39	18.03	21.15	16.73	17.02	0.91534601	0.04

C1_02900C_A	6.22	3.77	4.10	6.65	4.65	4.17	0.942931708	0.04
C2_09670C_A	45.20	55.92	56.17	52.51	59.56	62.93	0.909696945	0.04
C4_00340W_A	73.11	70.33	66.77	77.59	76.36	78.77	0.849615542	0.04
C7_01630W_A	7.56	12.01	10.42	12.19	11.14	10.04	0.939433187	0.04
C7_03080W_A	5.58	9.14	8.47	8.87	6.75	10.30	0.944435383	0.04
C3_03880C_A	77.33	67.89	68.06	77.04	77.50	81.28	0.84864478	0.03
CR_03410W_A	17.46	19.43	21.55	22.08	21.80	20.90	0.910164559	0.03
C5_02760W_A	16.41	13.27	15.26	14.54	17.63	17.42	0.907592919	0.03
C7_00930W_A	147.57	193.70	210.71	219.49	189.21	205.41	0.924818504	0.03
C4_05400C_A	1.95	0.56	1.29	0.57	1.43	2.18	0.975525479	0.03
C6_03260W_A	154.76	157.32	162.10	192.55	176.51	155.08	0.903165038	0.03
C1_12630C_A	4.96	2.96	3.03	4.17	4.13	3.74	0.952057613	0.03
C5_00440C_A	53.18	42.24	45.74	47.18	47.27	61.56	0.908122109	0.03
C6_00700C_A	29.14	33.98	38.62	38.10	40.22	34.26	0.926220787	0.03
C4_06400C_A	41.26	54.17	55.91	42.72	60.98	64.41	0.941143358	0.03
C2_00290W_A	13.40	10.89	11.68	14.08	14.73	10.82	0.936099688	0.03
CR_09980W_A	17.09	11.55	11.21	14.26	14.26	15.25	0.924043235	0.03
C6_02880W_A	16.00	15.06	16.78	19.77	16.88	16.15	0.920573208	0.03
C5_00180W_A	23.10	49.49	36.05	42.07	41.58	37.42	0.956163322	0.03
C4_01100C_A	2.27	1.26	2.47	2.02	1.80	2.79	0.959715776	0.03
C1_01360C_A	297.28	253.23	268.50	290.66	306.30	305.06	0.869642019	0.03
C1_06960W_A	63.66	56.15	61.59	74.05	77.34	47.60	0.943239977	0.03
C3_06400C_A	15.52	9.90	9.44	13.48	11.56	13.24	0.941004224	0.03
C2_05450C_A	6.83	4.70	4.75	7.70	5.28	4.79	0.959715776	0.03
C1_04010C_A	2.78	1.68	2.23	2.77	1.84	2.78	0.958328178	0.03
C7_03890C_A	31.12	42.48	21.70	31.96	30.77	43.15	0.954114294	0.03
C3_06780C_A	2.84	1.73	2.49	3.45	2.07	2.24	0.962062792	0.03
C7_03000C_A	4.03	6.76	9.39	11.15	4.93	6.30	0.972772309	0.03
C2_10470C_A	27.45	34.93	38.39	40.27	34.11	37.08	0.940095415	0.03
C1_03450C_A	24.09	15.46	16.72	22.98	21.10	17.36	0.943192491	0.03
C1_02470W_A	2.27	2.72	2.75	1.10	3.75	3.61	0.976127036	0.03
C1_05840W_A	4.16	5.79	7.38	5.61	5.54	8.08	0.961881976	0.03
C1_02040C_A	10.88	8.16	8.93	9.75	8.86	12.14	0.932915436	0.03
C7_00170W_A	31.59	0.12	29.32	19.66	22.73	23.50	0.991224937	0.03
C7_04340C_A	20.55	68.96	38.00	30.94	44.91	67.41	0.976025941	0.03
C2_02770W_A	44.51	44.85	52.07	55.01	59.89	40.23	0.948556551	0.03
C6_01320W_A	6.00	5.18	5.34	6.72	5.41	6.02	0.917598607	0.03
C5_01840C_A	4.04	2.37	3.41	3.05	2.86	4.88	0.961400174	0.03
C1_10080W_A	16.47	21.14	19.48	24.81	16.52	21.75	0.954114294	0.02
C7_04020C_A	164.39	173.49	218.50	102.30	202.55	312.69	0.972048394	0.02
C2_09000C_A	5.50	5.30	4.70	3.76	6.32	6.91	0.965376707	0.02
C3_05210C_A	4.46	4.57	4.27	5.37	4.33	4.95	0.925524443	0.02
C1_09860C_A	33.50	24.80	24.14	22.90	41.87	24.93	0.963812492	0.02
C3_02040C_A	2.38	2.46	3.49	3.46	2.09	3.63	0.972772309	0.02
C7_03160W_A	16.79	19.59	19.57	20.31	18.51	22.87	0.938831187	0.02
CR_04290W_A	16.63	10.47	16.57	19.00	14.56	14.41	0.96373297	0.02
C4_02050W_A	3.31	2.36	3.49	1.55	4.86	3.62	0.976643999	0.02
C1_02090C_A	2.15	1.48	2.32	2.52	2.21	1.77	0.968299711	0.02
CR_00640W_A	71.58	81.37	35.90	76.51	64.50	66.13	0.968650005	0.02
C2_02810C_A	29.32	20.94	40.10	32.98	43.40	22.08	0.973018859	0.02
C2_09720W_A	86.14	52.94	54.58	75.81	74.79	59.53	0.959481492	0.02
C6_03740W_A	1.57	1.29	0.92	1.15	1.85	1.10	0.97984704	0.02
C2_09870W_A	41.05	31.57	29.08	28.21	48.05	34.40	0.964473589	0.02
C1_02940C_A	41.62	83.61	76.83	67.63	77.78	78.33	0.972048394	0.02
C1_14080W_A	2.82	3.04	3.60	3.69	3.41	3.26	0.95290672	0.02
C1_04080W_A	8.74	5.26	8.05	5.70	8.22	10.15	0.973293982	0.02
C6_00350W_A	23.22	19.50	24.52	25.17	27.46	20.71	0.955896819	0.02
CR_04260W_A	8.60	16.18	17.20	18.66	14.69	12.74	0.977076057	0.02
C1_04890W_A	96.54	87.57	93.64	104.81	115.45	82.37	0.958328178	0.02
C1_06770W_A	9.22	4.79	10.82	5.48	11.00	10.58	0.979105121	0.02
CR_07090W_A	6.68	5.71	5.18	5.77	6.21	7.22	0.951502475	0.02
C5_05270C_A	37.20	19.46	37.59	35.71	34.90	31.69	0.973293982	0.02
C1_09380W_A	8.88	4.16	12.30	10.58	5.32	11.85	0.982390503	0.02

C1_04410C_A	115.33	124.23	130.54	137.04	125.01	143.36	0.951714643	0.02
C6_00190W_A	26.75	25.44	22.17	25.80	26.21	29.17	0.951714643	0.02
C1_07390W_A	36.28	28.09	45.25	43.38	44.28	31.44	0.974337943	0.02
C1_05280W_A	7.13	8.30	7.22	9.88	7.18	7.71	0.972772309	0.01
C7_01160C_A	1.54	22.74	28.84	47.07	9.74	1.90	0.995011819	0.01
C2_04700C_A	0.70	0.34	0.79	0.51	0.25	1.22	0.990955908	0.01
C2_08020C_A	23.19	14.54	14.93	14.34	22.15	20.54	0.977947266	0.01
C2_08810C_A	480.54	715.25	523.36	605.00	704.08	572.25	0.976078368	0.01
C1_14120C_A	45.20	56.06	57.33	61.80	54.19	57.46	0.972307802	0.01
CR_06250W_A	8.84	5.83	6.33	5.82	8.45	8.51	0.979105121	0.01
CR_07010W_A	68.03	96.34	181.99	150.26	114.00	116.01	0.985323048	0.01
C1_10540C_A	14.37	7.11	8.84	12.66	9.78	10.26	0.980846784	0.01
C1_01560W_A	20.26	17.51	15.04	19.76	17.99	19.68	0.966827308	0.01
C2_06270W_A	1.46	1.18	1.69	1.14	1.84	1.71	0.985976426	0.01
C1_07710C_A	17.24	11.47	12.73	18.87	11.69	14.36	0.979808309	0.01
C1_01770W_A	6.16	5.05	7.88	8.08	6.16	6.52	0.98170768	0.01
CR_08090W_A	529.78	417.72	512.88	495.92	613.88	470.90	0.976127036	0.01
C1_04560W_A	11.49	12.26	11.87	10.31	13.69	14.83	0.977715976	0.01
C1_09670C_A	16.99	38.57	44.12	50.56	26.39	32.72	0.990623452	0.01
C4_02400C_A	2.37	1.25	1.09	1.65	1.66	1.79	0.988644451	0.01
C1_00870W_A	10.50	10.72	12.55	12.88	10.54	13.36	0.97835702	0.01
C4_05970W_A	13.25	13.45	13.51	15.02	14.30	14.39	0.974337943	0.01
C6_01280W_A	15.19	10.32	10.73	11.93	13.16	14.09	0.981312015	0.01
C1_01170C_A	1.80	2.52	2.34	3.25	2.01	2.00	0.989209675	0.01
C6_01140C_A	5.55	1.31	1.58	1.45	2.49	5.09	0.994196059	0.01
C2_07370W_A	17.27	12.50	2.77	34.44	0.21	0.02	0.997595534	0.01
CR_00980C_A	52.47	25.85	52.44	36.57	48.19	56.63	0.989113741	0.01
C1_12050W_A	97.63	34.95	56.99	77.24	46.08	80.31	0.991821499	0.01
C5_03090W_A	29.10	33.38	44.64	37.50	37.79	41.46	0.987570718	0.01
C5_05190W_A	27.90	2.13	33.82	26.36	20.21	21.71	0.995956876	0.01
C2_05800C_A	18.90	14.69	12.77	18.01	14.59	17.56	0.987990456	0.01
C3_04720C_A	5.79	6.41	7.10	6.74	7.16	7.05	0.986813265	0.01
C4_00200C_A	313.92	263.87	306.99	339.86	318.15	298.77	0.983736594	0.01
C2_05640W_A	39.15	25.70	25.64	28.69	35.18	33.56	0.989706384	0.00
C1_06700W_A	10.28	7.86	9.33	10.61	7.66	11.51	0.989988023	0.00
C5_02930C_A	14.21	45.46	45.37	45.40	25.75	44.84	0.996486759	0.00
CR_09830W_A	0.36	0.09	2.88	1.86	1.46	0.28	0.998292067	0.00
C4_02140C_A	8.87	3.31	3.72	4.04	5.03	8.08	0.996957916	0.00
C4_06080W_A	5.84	12.38	12.90	10.74	10.52	12.43	0.997595534	0.00
C2_06610C_A	10.60	9.45	9.41	8.38	10.08	13.30	0.998955121	0.00
C6_01910W_A	224.43	179.80	229.00	228.75	228.54	225.21	0.998102639	0.00
C7_04140C_A	18.09	22.73	19.89	11.63	26.33	27.95	0.997316277	0.00
C3_05970C_A	67.62	42.10	62.81	57.75	67.34	59.85	0.995011819	0.00
C4_05420C_A	1.81	1.48	0.76	1.02	1.44	1.89	0.995902423	0.00
CR_00630W_A	2.51	4.71	2.89	4.19	3.22	3.56	0.995138719	0.00
C2_01170C_A	12.97	10.35	10.64	11.34	12.03	13.16	0.989706384	0.00
C2_05260W_A	29.88	25.12	23.89	28.68	28.61	27.49	0.988530661	0.00
CR_09230C_A	11.04	10.91	2.34	8.05	4.27	14.02	0.996635121	0.00
C1_11190W_A	31.18	25.08	29.46	30.33	29.95	31.91	0.986823291	0.00
C1_08810C_A	3.71	4.03	3.12	4.43	3.42	3.88	0.990347473	0.00
C1_07820W_A	3.14	1.34	4.17	3.32	2.48	3.49	0.994473032	0.00
CR_04460C_A	15.59	16.31	13.47	20.48	18.61	9.57	0.99280761	0.00
C2_07080C_A	7.71	10.99	10.49	13.58	7.08	11.03	0.992616104	0.00
C5_01350W_A	10.92	11.90	13.12	12.20	12.37	14.24	0.987107746	0.00
C2_03620W_A	2.94	9.64	8.84	9.58	8.51	5.19	0.994141372	-0.01
C4_04440W_A	55.29	47.99	51.31	40.59	67.89	57.45	0.989108609	-0.01
C3_03100C_A	24.58	15.93	12.52	17.11	18.67	20.88	0.989962673	-0.01
C1_03540C_A	3.92	4.92	8.76	6.69	5.97	6.26	0.992456493	-0.01
C7_01990C_A	5.44	6.21	9.24	8.64	6.86	7.04	0.989745848	-0.01
C3_06430W_A	60.30	53.63	51.31	64.17	51.91	61.62	0.979746966	-0.01
C3_01750C_A	11.22	7.03	6.81	4.81	11.77	10.17	0.991224937	-0.01
C1_12220W_A	1.30	0.94	0.99	1.01	1.27	1.20	0.989745848	-0.01
C1_06070W_A	4.58	5.11	6.71	8.10	4.24	5.40	0.990319506	-0.01

CR_09880W_A	119.08	44.69	63.99	77.51	129.79	30.60	0.992456493	-0.01
C2_03380W_A	82.62	75.59	78.23	91.55	93.13	68.26	0.980846784	-0.01
CR_08450C_A	12.64	10.75	9.28	11.86	11.33	11.84	0.977389059	-0.01
C4_05770C_A	9.31	7.14	5.64	7.38	7.43	8.84	0.982173422	-0.01
C2_10060C_A	1.53	0.78	0.70	0.63	1.01	1.61	0.992241622	-0.01
C1_01910W_A	11.38	12.31	14.70	18.30	11.00	12.01	0.985251464	-0.01
C7_00040C_A	15.44	1.29	18.40	12.00	13.17	12.07	0.993464655	-0.01
C4_04310W_A	27.54	35.15	32.78	35.08	41.76	25.57	0.984068008	-0.01
C2_00450C_A	20.10	19.81	17.81	28.92	4.66	29.03	0.991870169	-0.01
CR_09970W_A	605.02	317.24	297.28	364.00	463.54	466.54	0.98454832	-0.01
C2_09150W_A	30.64	24.45	20.81	25.33	25.32	30.64	0.975719935	-0.01
C2_09780C_A	58.19	64.29	64.23	71.26	59.49	70.17	0.972412246	-0.01
C5_04130C_A	27.36	20.77	29.30	31.21	29.21	22.24	0.979105121	-0.01
CR_05250C_A	2.96	3.69	2.01	2.87	3.14	3.32	0.983165006	-0.01
C2_02890W_A	12.19	13.41	13.86	14.86	12.11	15.51	0.972772309	-0.01
C1_11280W_A	13.23	11.46	11.29	13.51	12.44	12.62	0.968508611	-0.01
C6_02810C_A	8.40	7.91	6.81	8.25	8.73	7.77	0.970509404	-0.01
CR_05530C_A	22.82	32.37	18.40	16.40	32.21	30.72	0.985286101	-0.01
CR_02670C_A	27.16	22.84	27.39	34.26	20.36	28.27	0.97608574	-0.01
C7_03410C_A	5.50	3.66	3.28	4.28	4.00	5.01	0.976782741	-0.01
C7_01330C_A	1.90	1.44	1.11	1.52	1.55	1.69	0.97619694	-0.01
CR_09960C_A	35.48	25.91	22.11	28.44	28.04	32.58	0.972406307	-0.01
C3_03370C_A	16.65	16.57	20.46	18.04	19.37	20.04	0.970445041	-0.01
C2_09270C_A	68.00	62.09	64.43	79.34	61.31	67.54	0.962850764	-0.01
C1_11120C_A	72.77	71.84	67.01	89.23	77.08	59.62	0.973178066	-0.01
CR_08480C_A	199.05	231.90	294.25	296.04	221.86	262.06	0.974738933	-0.01
C6_01430C_A	33.72	59.22	64.29	63.00	48.89	58.08	0.979297441	-0.01
CR_02190C_A	7.91	6.81	4.11	8.07	5.72	6.27	0.978124015	-0.01
C2_01670C_A	14.19	19.97	19.08	23.23	13.09	21.16	0.976944828	-0.01
C3_04460W_A	9.65	10.83	10.74	11.66	10.54	11.25	0.95810075	-0.01
C4_05190C_A	2.98	3.87	3.84	4.45	3.26	3.73	0.974337943	-0.02
C1_00160C_A	90.76	51.59	72.38	77.49	71.27	79.35	0.969016137	-0.02
C7_02490W_A	4.14	2.85	3.37	3.42	3.95	3.64	0.965648101	-0.02
CR_03050C_A	16.30	13.74	10.79	15.68	13.75	14.08	0.9657418	-0.02
C3_02590W_A	8.35	6.12	7.08	7.43	7.61	7.93	0.955614506	-0.02
C3_00110C_A	58.76	41.40	43.59	56.58	57.83	37.68	0.972412246	-0.02
C4_05050C_A	5.55	5.15	8.63	6.23	6.68	7.78	0.974337943	-0.02
C3_00500C_A	8.04	8.89	7.98	8.01	5.29	13.58	0.977947266	-0.02
C5_00300C_A	8.30	7.04	7.59	9.02	7.41	8.03	0.949307958	-0.02
C2_02990C_A	32.05	27.13	24.26	26.97	32.28	29.55	0.95313596	-0.02
C2_08600W_A	3.86	1.94	3.08	3.63	2.90	2.91	0.976045063	-0.02
C5_03460C_A	4.51	3.39	4.77	3.07	5.11	5.33	0.974530816	-0.02
C1_05120W_A	15.59	14.21	15.56	16.09	14.07	18.29	0.948642171	-0.02
CR_01620C_A	622.80	679.30	823.27	848.63	740.30	682.31	0.959495279	-0.02
C1_05190C_A	17.39	25.39	22.52	21.01	22.34	26.73	0.969787127	-0.02
C4_06450W_A	8.49	6.18	4.74	5.44	7.30	7.90	0.974337943	-0.02
CR_00750C_A	0.31	17.20	19.44	13.58	13.68	13.08	0.992456493	-0.02
C3_01030W_A	8.77	3.40	4.16	3.56	5.41	8.31	0.979105121	-0.02
C2_00630C_A	16.81	19.60	16.24	21.75	17.36	17.10	0.956597438	-0.02
C1_09340C_A	6.47	6.66	5.66	7.46	5.70	6.90	0.948642171	-0.02
CR_09610C_A	34.77	29.50	23.68	32.90	28.76	31.77	0.947630459	-0.02
C4_02390W_A	2818.42	2184.31	1993.84	2277.73	2758.80	2366.95	0.94653191	-0.02
C2_07980W_A	25.05	32.62	32.64	25.99	35.26	35.24	0.955615734	-0.02
C1_00580W_A	15.03	15.18	18.26	16.16	16.58	18.87	0.941480092	-0.02
C1_00030C_A	19.37	16.99	16.33	15.22	20.68	19.96	0.952286476	-0.02
C7_02080W_A	11.87	9.49	1.39	10.83	7.88	5.27	0.983678542	-0.02
CR_08420W_A	15.56	14.15	12.66	13.85	16.41	14.68	0.934297253	-0.02
C7_02390W_A	11.82	15.67	21.09	13.31	16.06	22.67	0.966708209	-0.02
CR_05880W_A	29.94	5.35	17.48	7.86	25.76	21.24	0.980846784	-0.02
CR_09340W_A	184.39	192.34	199.06	196.34	210.80	204.77	0.913784062	-0.03
C5_03680W_A	8.77	8.98	10.34	13.71	6.99	9.21	0.959989925	-0.03
CR_05500C_A	4.85	5.42	3.39	3.43	4.27	6.86	0.964821295	-0.03
CR_08690C_A	26.57	3.79	27.69	1.60	55.44	2.27	0.991821499	-0.03

CR_07240C_A	54.50	15.21	49.97	34.37	54.33	36.22	0.974337943	-0.03
C7_03620C_A	15.52	11.90	9.09	14.58	15.71	7.97	0.963817533	-0.03
C2_06030W_A	5.24	2.91	4.47	3.01	4.46	5.87	0.963609494	-0.03
C3_04530C_A	12.71	14.37	12.30	15.96	13.32	12.52	0.934932695	-0.03
C6_02670C_A	42.41	31.68	30.69	34.31	42.96	33.02	0.939175721	-0.03
C5_03210C_A	0.68	0.42	1.20	1.00	1.08	0.34	0.978213107	-0.03
C1_04850C_A	7.83	8.96	8.10	11.37	8.25	6.89	0.966313094	-0.03
C1_07140C_A	14.91	13.87	20.99	24.32	21.61	6.30	0.973293982	-0.03
C2_08320C_A	3.36	2.89	1.56	3.27	2.44	2.56	0.966830495	-0.03
C4_04800W_A	67.99	89.03	96.27	92.85	104.07	71.37	0.945836563	-0.03
C3_02780W_A	75.51	67.43	76.43	88.15	74.00	69.56	0.915423778	-0.03
CR_01210C_A	46.40	25.16	31.47	34.30	37.68	35.95	0.940053626	-0.03
C1_04400C_A	82.61	104.06	98.23	114.87	89.76	97.90	0.925524443	-0.03
C5_04680W_A	9.91	5.56	5.69	7.20	6.77	8.23	0.942753314	-0.03
CR_10500C_A	13.69	11.57	23.37	14.12	17.33	20.12	0.954730413	-0.03
C6_01190C_A	3.71	2.05	2.24	2.64	2.60	3.16	0.942931708	-0.03
C4_05590W_A	4.72	5.30	3.63	5.99	3.62	4.84	0.942099851	-0.03
C1_08040W_A	23.32	22.96	20.89	25.92	24.43	20.53	0.931819973	-0.03
C6_00480C_A	17.25	21.81	30.54	31.88	28.99	12.43	0.962932593	-0.03
C4_06680C_A	17.41	16.28	13.31	15.91	16.94	16.70	0.901133527	-0.03
C3_07000W_A	56.24	36.96	28.81	35.42	51.40	40.65	0.945531627	-0.03
C1_11260C_A	1.32	5.11	18.62	13.30	6.49	6.65	0.98170768	-0.03
C3_05100C_A	841.66	655.29	749.54	792.46	794.61	775.25	0.877143463	-0.03
CR_04090C_A	134.87	217.71	266.64	207.54	207.62	244.90	0.945481791	-0.03
C5_00580W_A	7.72	7.94	8.13	2.49	10.94	11.74	0.968386455	-0.04
C2_00180C_A	19.18	22.21	28.00	28.55	25.79	18.78	0.940163224	-0.04
C2_04880C_A	67.90	62.06	53.21	62.85	62.55	67.55	0.88314809	-0.04
C7_01860W_A	4.17	2.35	2.73	4.09	2.35	3.29	0.959989925	-0.04
C4_02610C_A	2.79	4.46	3.26	4.57	3.63	2.91	0.947101111	-0.04
C3_07010W_A	43.99	26.79	24.50	26.33	42.35	30.53	0.943239977	-0.04
C1_08320W_A	229.45	242.45	224.06	257.85	245.79	229.65	0.878079501	-0.04
C6_03730C_A	3.94	2.17	3.15	3.15	3.38	3.14	0.939814881	-0.04
C4_03200C_A	57.89	37.67	36.96	70.01	25.03	43.82	0.955201566	-0.04
CR_08620C_A	10.41	18.23	19.02	10.29	20.77	19.55	0.954114294	-0.04
C2_00710W_A	28.04	22.68	24.75	31.41	19.19	28.84	0.921768365	-0.04
C4_04340C_A	21.19	29.68	32.23	41.20	22.31	24.39	0.947826029	-0.04
C6_02270C_A	1.23	0.97	0.67	1.01	0.93	1.07	0.950013868	-0.04
C7_01020C_A	2.00	2.80	9.51	6.93	4.41	3.72	0.973293982	-0.04
C1_03350C_A	11.10	10.87	6.80	13.81	4.53	12.05	0.956825224	-0.04
C2_05020W_A	30.40	26.46	30.36	28.04	34.18	29.29	0.896350919	-0.04
C3_01360C_A	31.10	24.98	27.01	31.58	28.05	27.40	0.872351779	-0.04
C1_03960C_A	8.51	4.50	4.07	5.95	5.70	6.12	0.938517587	-0.04
C1_14170W_A	35.57	37.71	52.75	43.58	46.75	42.34	0.916197737	-0.04
C2_10130W_A	0.87	0.33	0.75	0.60	0.68	0.76	0.955318312	-0.04
C5_04560C_A	1117.79	1412.85	1172.67	1237.62	1578.29	1074.88	0.920573208	-0.04
C6_00650C_A	238.57	207.17	370.19	372.74	217.36	268.85	0.940095415	-0.04
C2_02280W_A	11.59	14.65	18.21	16.14	14.60	16.16	0.922530493	-0.04
C7_00200W_A	36.82	27.85	30.48	31.84	32.71	35.18	0.867836511	-0.04
C2_10660W_A	70.46	54.20	50.26	52.15	69.89	60.72	0.904264237	-0.04
CR_07940W_A	2.56	2.93	2.38	3.20	2.50	2.59	0.917498829	-0.04
C1_00610W_A	97.05	111.48	103.96	110.67	105.60	112.77	0.863192665	-0.04
C1_04750W_A	1.11	5.04	5.18	4.94	3.38	3.79	0.966801513	-0.04
C2_04520C_A	6.51	2.34	1.29	2.71	3.63	4.15	0.966827308	-0.04
C1_06850W_A	73.45	52.40	54.30	66.27	63.59	57.92	0.885627198	-0.04
C1_07890C_A	66.25	59.37	44.47	64.70	64.34	48.31	0.912255247	-0.04
C7_00020C_A	241.40	345.88	316.22	324.22	307.06	322.28	0.903183401	-0.04
C5_03850W_A	7.37	3.89	3.88	5.89	3.72	6.21	0.950766838	-0.04
C2_01920C_A	14.23	10.65	11.37	11.66	12.03	14.24	0.873354218	-0.04
C4_03090W_A	42.72	26.64	38.52	40.06	33.71	38.90	0.902914511	-0.04
CR_08930C_A	63.88	59.35	74.46	35.85	82.30	89.67	0.942099851	-0.04
CR_05770W_A	20.87	10.84	13.63	10.48	19.93	16.58	0.937646027	-0.04
C5_02490C_A	3.28	3.52	2.43	2.86	3.86	2.94	0.926925427	-0.04
C5_01210W_A	86.18	78.17	66.21	79.81	81.38	79.73	0.851736441	-0.05

C2_02920W_A	120.49	131.89	140.81	130.45	135.57	146.87	0.858112158	-0.05
C6_01830W_A	79.54	57.88	60.88	69.33	69.14	68.23	0.863192665	-0.05
C6_03910C_A	7.20	7.83	8.11	9.23	7.71	7.26	0.902208173	-0.05
C2_00330C_A	39.56	33.81	30.91	38.61	40.10	29.79	0.889966691	-0.05
C3_02430W_A	5.23	4.68	5.23	5.65	4.24	5.99	0.886093761	-0.05
C1_02410C_A	5.03	3.00	3.50	3.03	5.52	3.39	0.939390768	-0.05
CR_10680W_A	38.95	28.30	36.37	32.01	39.25	36.71	0.873311131	-0.05
C1_02350W_A	7.91	4.09	9.44	7.01	7.08	8.25	0.936070755	-0.05
C5_02060W_A	8.35	9.43	12.04	10.91	7.99	12.48	0.916661263	-0.05
C1_03820W_A	8.84	6.56	5.62	6.16	6.71	9.06	0.912981312	-0.05
CR_05440W_A	105.44	107.92	68.80	48.19	66.36	184.43	0.953549642	-0.05
C2_02290C_A	2.99	2.34	4.08	3.61	3.70	2.51	0.937206248	-0.05
CR_08200C_A	7.20	5.60	7.66	7.38	5.21	8.85	0.906574704	-0.05
C5_02150C_A	12.29	12.32	11.53	12.44	12.54	12.79	0.830254635	-0.05
C4_05160C_A	4.26	1.51	2.27	1.84	2.88	3.59	0.948703006	-0.05
C3_00130C_A	93.96	27.02	68.88	117.84	45.52	31.02	0.959989925	-0.05
C4_04140W_A	3.76	2.97	2.31	3.49	2.89	3.04	0.916384558	-0.05
C1_03070C_A	29.03	23.02	19.29	24.43	25.07	24.64	0.868684473	-0.05
C2_07090C_A	18.63	16.93	20.80	18.69	20.07	20.03	0.843086719	-0.05
C2_07180W_A	1.83	2.33	2.03	2.00	3.11	1.29	0.948642171	-0.05
C7_03660C_A	70.81	98.49	99.20	127.24	97.82	54.84	0.932304719	-0.05
C3_06990W_A	4.47	2.75	3.21	3.12	3.09	4.64	0.917598607	-0.05
C1_14110C_A	812.64	627.22	734.46	735.11	822.44	697.93	0.842370402	-0.05
C3_03920W_A	138.23	151.68	148.91	134.06	135.62	189.87	0.872913619	-0.05
C5_05100C_A	2.44	4.12	5.68	5.48	3.17	4.20	0.938979213	-0.05
C5_03380W_A	3.17	4.06	4.25	4.53	3.64	3.81	0.904272049	-0.05
CR_00160C_A	93.42	105.03	58.65	71.23	84.96	112.62	0.912481917	-0.05
C4_06370C_A	1.11	0.92	4.76	1.67	2.07	3.40	0.962339103	-0.05
C4_00520W_A	5.56	4.56	5.53	4.78	3.77	7.75	0.927007305	-0.05
C2_03240C_A	28.37	24.56	20.38	24.30	23.85	28.05	0.839384464	-0.05
C1_04630C_A	5.48	6.38	6.23	6.96	5.47	6.44	0.85948853	-0.05
C7_04330C_A	30.80	42.17	33.97	34.85	34.31	42.77	0.881020409	-0.05
C6_00410C_A	17.90	17.95	18.48	22.03	20.11	14.16	0.883859576	-0.06
C1_08450C_A	48.28	57.45	65.79	70.25	53.16	55.33	0.881399469	-0.06
CR_00760C_A	0.10	11.69	10.98	8.63	7.44	8.21	0.980364699	-0.06
C4_00790C_A	1.16	0.58	0.79	0.83	0.82	0.98	0.925524443	-0.06
C2_09790W_A	6.54	2.83	3.39	3.94	3.95	5.26	0.922501507	-0.06
C5_02310C_A	2.18	2.69	3.67	3.45	2.57	2.88	0.909252495	-0.06
CR_09530C_A	34.25	20.80	35.87	30.13	36.62	26.91	0.896161594	-0.06
C1_01470W_A	44.07	35.72	36.00	49.14	27.59	43.48	0.887898649	-0.06
C4_03440C_A	0.92	0.91	0.37	0.72	0.56	1.01	0.942753314	-0.06
C2_00540W_A	29.84	13.56	28.24	13.14	30.34	30.34	0.932915436	-0.06
CR_00260W_A	66.69	52.38	49.44	55.15	61.53	57.32	0.819789418	-0.06
C3_03980C_A	4.96	6.28	5.27	6.22	4.79	6.17	0.875816517	-0.06
C6_00520W_A	2.55	2.68	2.07	4.18	2.04	1.38	0.952286476	-0.06
C5_00100C_A	100.71	118.02	52.82	134.97	4.74	145.70	0.975860881	-0.06
C1_09960W_A	37.16	33.67	38.96	40.77	33.56	39.52	0.829353491	-0.06
C1_01700W_A	27.32	12.92	12.67	19.06	16.60	18.49	0.90746523	-0.06
CR_03800C_A	8.97	11.40	13.55	15.80	9.97	9.48	0.907601776	-0.06
C4_04650W_A	13.00	12.77	13.61	12.96	15.74	11.95	0.859020857	-0.06
CR_04190W_A	120.19	161.08	125.36	142.24	142.66	137.61	0.8455024	-0.06
C7_03940C_A	17.03	29.29	19.75	21.59	16.15	31.61	0.910558709	-0.06
C1_03440C_A	33.18	27.47	24.06	39.59	25.56	22.00	0.892755242	-0.06
C5_02180C_A	14.08	8.80	10.34	12.39	10.47	11.29	0.849883294	-0.06
C1_12860C_A	7.68	5.12	5.31	5.50	7.59	5.49	0.884691851	-0.06
C3_03300C_A	37.73	33.57	25.67	20.04	27.50	53.35	0.91664166	-0.06
CR_00810W_A	2.28	3.44	2.70	2.31	3.50	3.02	0.927995748	-0.06
C1_05320C_A	6.20	8.61	6.98	7.52	8.63	6.38	0.878019059	-0.06
C4_02880C_A	3.23	2.65	5.14	4.22	4.61	2.50	0.925524443	-0.07
C4_06090C_A	3.43	4.66	4.47	4.52	4.61	3.87	0.869516583	-0.07
C2_01310W_A	11.20	5.53	4.70	5.62	7.56	8.72	0.912481917	-0.07
C3_03190C_A	7.04	6.35	4.76	7.48	5.31	5.90	0.872320584	-0.07
C2_10760C_A	21.93	13.42	16.43	20.40	10.35	22.67	0.903165038	-0.07

C6_03590C_A	15.66	19.62	18.74	17.78	19.03	19.01	0.836558745	-0.07
C4_01920W_A	38.07	22.40	26.68	29.11	20.63	40.06	0.898705856	-0.07
C5_00280C_A	24.55	23.94	25.03	24.18	27.08	24.61	0.779871131	-0.07
C2_06820C_A	48.95	59.88	55.55	58.64	48.94	62.93	0.824637803	-0.07
C6_04360C_A	21.28	29.05	28.39	28.60	23.86	29.20	0.849811081	-0.07
C4_01050C_A	1433.31	1500.81	1004.84	1552.59	1250.05	1255.22	0.852705515	-0.07
C5_02580W_A	23.89	20.49	23.38	21.72	15.69	32.83	0.892306096	-0.07
C6_02800W_A	17.95	27.51	35.88	35.89	21.93	26.73	0.906275251	-0.07
CR_10840C_A	795.89	1041.30	1120.63	954.52	1164.00	940.01	0.855101095	-0.07
C5_00450C_A	32.44	36.73	42.04	40.09	32.32	42.80	0.833788509	-0.07
CR_09330C_A	48.12	32.04	36.79	32.50	43.73	43.66	0.840591551	-0.07
CR_09270C_A	31.20	7.16	31.20	22.16	13.81	35.49	0.940095415	-0.07
C2_04410W_A	5.28	4.38	4.30	6.35	2.52	5.53	0.906967921	-0.07
C2_04210W_A	4.48	6.92	7.94	6.37	6.29	7.39	0.885627198	-0.07
CR_04780W_A	9.46	6.56	6.06	6.37	8.34	7.90	0.883846219	-0.07
C6_02690C_A	2.15	4.29	3.82	3.97	3.74	2.93	0.906275251	-0.07
C2_09160W_A	1.31	1.06	1.21	0.99	1.52	1.17	0.91154023	-0.07
CR_04960C_A	58.34	34.61	33.22	30.84	46.98	51.16	0.885627198	-0.07
CR_02970C_A	19.58	18.74	17.63	19.63	18.31	19.63	0.711018354	-0.07
C5_04770W_A	7.23	6.26	6.33	7.40	6.32	6.69	0.823746191	-0.07
C2_08610W_A	9.83	4.54	4.37	4.10	6.48	8.54	0.91661373	-0.07
C5_05290C_A	54.36	40.48	110.45	85.42	71.15	54.13	0.916641342	-0.07
C4_06690C_A	105.84	147.85	140.83	141.80	132.86	132.91	0.833788509	-0.07
CR_09860W_A	3.00	0.39	11.02	5.08	8.50	1.01	0.974337943	-0.07
C3_03820C_A	5.08	8.07	8.06	5.93	9.17	6.74	0.890107093	-0.07
C1_12790C_A	7.44	5.58	7.36	4.93	7.75	8.18	0.869516583	-0.07
C2_07410W_A	32.76	35.94	15.85	39.47	38.78	7.36	0.941211604	-0.07
CR_00050W_A	3.57	1.92	3.25	2.62	3.81	2.51	0.898947606	-0.07
C2_10780C_A	1.26	0.22	1.79	1.38	0.76	1.19	0.949711938	-0.07
C1_08680C_A	49.15	51.36	54.32	53.14	54.43	51.32	0.756074251	-0.07
C1_08990C_A	13.11	12.32	15.24	13.81	12.82	15.15	0.767064866	-0.08
C1_09610W_A	36.53	41.77	44.68	40.45	39.65	46.49	0.783948366	-0.08
CR_09110C_A	2.15	2.86	2.00	1.88	2.12	3.19	0.897325178	-0.08
C1_02590C_A	200.33	195.09	221.01	220.25	207.08	203.79	0.718455587	-0.08
C1_06940C_A	23.18	21.43	22.05	23.76	26.04	18.13	0.811991015	-0.08
C2_07320W_A	61.79	102.28	103.30	97.32	108.23	69.06	0.878690453	-0.08
CR_04370W_A	4.56	6.80	4.76	4.49	7.00	5.00	0.885539652	-0.08
C2_00810C_A	10.50	16.51	10.56	22.04	5.06	11.65	0.925524443	-0.08
C7_01720W_A	29.37	22.27	35.93	28.23	26.52	34.90	0.833476097	-0.08
C2_04750W_A	8.32	5.15	4.38	5.43	6.09	6.64	0.859476817	-0.08
C5_00880C_A	17.07	24.47	19.21	21.12	23.68	17.45	0.844125341	-0.08
C2_05140W_A	4.00	5.80	5.45	4.19	8.00	3.35	0.902018718	-0.08
CR_07340C_A	29.12	18.35	19.79	29.29	17.47	21.62	0.85733855	-0.08
C7_00300W_A	50.71	49.77	53.28	41.95	59.02	56.13	0.79089759	-0.08
C2_06650C_A	2.08	0.93	1.16	1.46	1.28	1.47	0.896350919	-0.08
CR_02400W_A	1.13	1.44	0.98	1.81	0.79	1.03	0.913405382	-0.08
C7_02850W_A	6.09	9.01	22.10	17.81	11.23	9.17	0.923726479	-0.08
C4_02320C_A	172.56	385.67	282.46	339.08	240.84	289.94	0.885568554	-0.08
C7_00340C_A	42.36	49.04	42.43	48.78	43.69	44.52	0.748413394	-0.08
C7_00670C_A	8.11	5.89	6.17	6.30	6.93	7.30	0.765567238	-0.08
C1_14400C_A	6.31	7.54	9.56	9.58	11.77	2.36	0.927995748	-0.08
CR_02040W_A	44.95	39.19	40.71	44.92	40.72	41.65	0.66258915	-0.08
C4_02220C_A	1.39	0.97	0.54	0.98	0.85	1.13	0.903308204	-0.08
C7_01690W_A	17.13	20.55	21.53	18.78	16.20	25.74	0.845518284	-0.08
C5_01220W_A	12.14	8.00	9.87	9.47	10.22	10.86	0.793215992	-0.08
C6_01920C_A	20.45	15.45	16.19	16.79	18.34	17.87	0.743118068	-0.08
C2_09170W_A	22.97	15.62	12.75	16.93	15.47	19.80	0.83977317	-0.08
C5_01970C_A	18.73	15.60	16.87	20.36	13.99	17.88	0.781545173	-0.08
CR_07770C_A	22.30	19.84	20.95	22.39	23.01	18.77	0.75829345	-0.08
C5_04260W_A	17.75	16.59	18.28	19.86	17.72	16.02	0.760321996	-0.08
C1_14300C_A	24.23	25.86	26.69	26.59	25.72	26.14	0.698185685	-0.08
C3_07630C_A	37.19	46.27	24.95	33.32	37.14	40.47	0.848880927	-0.08
C5_01620C_A	2.95	1.70	1.75	1.85	2.34	2.35	0.891312464	-0.08

C3_07600W_A	10.17	14.49	14.50	15.07	11.00	14.15	0.84700868	-0.08
CR_03220C_A	12.66	8.71	9.53	10.37	9.73	11.35	0.780025516	-0.08
CR_05380C_A	334.82	326.08	89.56	199.61	202.30	365.60	0.916779138	-0.08
C3_00650W_A	0.99	1.13	1.97	1.68	1.07	1.44	0.903183401	-0.08
C2_03530W_A	11.02	14.27	13.81	13.93	11.72	14.42	0.796301667	-0.08
C3_00900C_A	25.49	34.10	33.76	40.34	30.49	24.51	0.851049947	-0.08
C2_07010W_A	27.73	34.14	31.98	35.35	27.45	33.21	0.780539091	-0.09
C1_02920W_A	34.91	63.87	57.28	62.07	45.30	53.19	0.859675066	-0.09
C2_01520W_A	12.62	15.61	17.01	16.89	14.30	15.00	0.802514009	-0.09
C2_02780C_A	5.80	2.26	2.93	3.85	4.52	2.63	0.904394834	-0.09
C3_06800C_A	6.40	4.15	4.59	4.67	3.69	6.98	0.868490205	-0.09
C5_02970W_A	4.00	5.02	6.04	5.15	5.11	5.10	0.819789418	-0.09
C3_06330W_A	1030.24	1033.89	934.74	907.70	839.67	1319.97	0.787986825	-0.09
CR_10600C_A	82.72	84.02	76.04	69.91	93.25	83.71	0.753517531	-0.09
C2_07680W_A	7.53	10.14	12.00	9.68	11.00	9.54	0.841082791	-0.09
C2_03420C_A	4.96	4.88	4.59	5.46	4.21	5.03	0.758533838	-0.09
C3_03200C_A	14.13	13.76	7.01	11.41	10.99	12.96	0.88126583	-0.09
C7_04280C_A	138.83	68.18	62.99	84.71	92.19	94.29	0.860924675	-0.09
C3_03290C_A	6.86	5.60	4.04	4.68	5.21	6.86	0.83502457	-0.09
CR_05050W_A	594.77	743.04	826.11	888.44	611.69	710.76	0.815588377	-0.09
C1_00480C_A	14.80	16.12	14.03	17.42	14.70	13.58	0.763554705	-0.09
C4_07220C_A	13.78	9.02	8.97	8.48	12.78	10.77	0.828260582	-0.09
CR_09640C_A	46.61	75.45	69.42	78.99	49.41	67.76	0.857484445	-0.09
C7_01340W_A	9.66	5.99	5.69	8.33	5.84	7.40	0.83502457	-0.09
C2_02350C_A	6.22	5.20	4.99	6.62	4.81	5.20	0.774913805	-0.09
C6_02540C_A	6.17	4.65	3.41	4.25	5.24	4.92	0.85728193	-0.09
C1_00960C_A	8.62	6.91	5.95	7.60	6.61	7.54	0.757365037	-0.09
C7_02890C_A	17.67	40.37	25.33	25.93	30.89	28.78	0.872320584	-0.09
C6_01840C_A	30.34	33.95	34.66	33.58	33.35	33.63	0.710390557	-0.09
C5_01940W_A	14.21	13.52	15.90	17.37	13.38	13.52	0.765882216	-0.09
C1_07410C_A	64.57	140.94	96.97	130.68	102.34	76.02	0.879341877	-0.09
C2_06330C_A	5.48	3.42	5.15	3.31	6.31	4.51	0.861395103	-0.09
CR_04150W_A	2.32	3.76	2.81	3.47	3.11	2.44	0.869840157	-0.09
C3_00820W_A	16.08	19.14	19.29	20.50	17.45	17.38	0.751256312	-0.09
C6_02380W_A	7.48	4.39	7.81	7.22	6.80	5.77	0.833041658	-0.09
C2_03050W_A	13.96	12.51	13.96	13.88	13.96	13.05	0.6744447	-0.09
C1_08930C_A	37.82	32.27	41.58	26.38	54.91	30.92	0.866734716	-0.09
C2_09710C_A	215.54	112.57	183.62	155.09	189.29	168.73	0.816595077	-0.09
CR_00100C_A	63.72	45.44	60.29	19.03	92.07	59.02	0.906967921	-0.10
CR_00310C_A	34.24	11.91	5.50	3.48	12.46	35.91	0.94304642	-0.10
CR_04040C_A	12.10	23.58	14.66	19.76	15.60	15.98	0.853890695	-0.10
C2_07610C_A	22.06	21.00	19.13	21.08	20.29	21.53	0.612444807	-0.10
CR_05390W_A	488.20	529.25	310.53	302.37	292.26	764.35	0.885814895	-0.10
C3_00450C_A	42.51	41.72	38.95	58.36	8.23	59.28	0.921673012	-0.10
C4_02260C_A	1.09	0.96	0.64	1.25	0.48	0.99	0.907727455	-0.10
C7_02330W_A	7.13	11.54	7.29	10.33	7.48	8.62	0.83156898	-0.10
C5_04270C_A	267.86	318.73	362.97	323.38	338.37	300.49	0.755829725	-0.10
C3_04610W_A	0.70	0.69	0.36	0.23	0.96	0.56	0.924818504	-0.10
C5_02200W_A	0.87	1.07	1.15	1.10	0.79	1.21	0.883846219	-0.10
C2_07110C_A	8.52	11.37	8.97	8.85	10.42	9.93	0.767677371	-0.10
C1_04970W_A	3.15	2.13	1.60	1.94	2.32	2.65	0.833476097	-0.10
C2_09810C_A	10.74	10.72	9.31	10.51	9.13	11.46	0.712921372	-0.10
C2_05730C_A	7.02	7.47	7.38	8.47	5.67	7.98	0.774913805	-0.10
C1_08340C_A	36.04	44.16	36.51	37.73	34.42	46.01	0.752942942	-0.10
C4_01080W_A	4.34	4.44	5.06	5.64	4.85	3.43	0.81179163	-0.10
C6_03160C_A	76.88	61.96	75.13	62.04	73.10	80.37	0.687035203	-0.10
CR_10280W_A	29.68	32.06	28.96	31.29	28.84	31.22	0.707645013	-0.10
C3_00570C_A	21.03	17.77	20.34	15.83	21.32	22.41	0.73977486	-0.10
C1_09950C_A	44.23	44.73	44.73	43.14	48.09	43.34	0.661090907	-0.10
C3_06810W_A	5.40	4.91	4.07	4.50	4.67	5.30	0.717664247	-0.10
C3_01850W_A	3.03	1.75	3.55	2.48	2.90	2.98	0.853890695	-0.10
C2_07750W_A	18.86	12.42	9.74	18.40	10.15	12.49	0.853028034	-0.10
C6_00780W_A	8.27	8.30	7.74	8.38	7.85	8.25	0.61021944	-0.10

C4_04000W_A	11.65	16.83	12.99	17.28	9.96	14.80	0.824555588	-0.10
CR_10620C_A	39.18	53.14	40.80	22.56	56.56	55.62	0.865042792	-0.10
C3_01790C_A	3.75	3.45	3.85	3.21	5.68	2.11	0.869516583	-0.10
C1_11400C_A	6.02	5.59	6.00	6.78	5.28	5.64	0.692309832	-0.10
C7_00230W_A	7.73	7.55	9.28	7.84	8.91	7.93	0.727357591	-0.10
C1_03760C_A	18.77	9.84	23.08	20.52	15.66	15.49	0.845710161	-0.10
C3_07260C_A	8.95	10.32	9.12	9.13	9.74	9.72	0.661933844	-0.10
C5_01390C_A	4.74	4.21	2.94	3.15	4.03	4.74	0.841022043	-0.10
C2_06220C_A	21.30	16.42	17.20	17.71	21.06	16.15	0.740032415	-0.10
C2_03200W_A	4.95	3.18	3.80	4.18	3.36	4.46	0.78750966	-0.10
C4_07230C_A	31.86	21.32	18.69	20.39	31.75	19.19	0.827505705	-0.10
C5_03600W_A	17.78	14.33	11.77	10.89	17.40	15.56	0.794365923	-0.11
C1_12180C_A	17.80	22.63	19.29	20.27	19.68	20.21	0.713927474	-0.11
C4_03900C_A	1.41	2.32	1.55	1.49	1.22	2.65	0.872861048	-0.11
C7_02520W_A	5.29	2.26	3.52	3.47	3.82	3.71	0.853652483	-0.11
C1_07690C_A	4.39	1.69	2.65	3.32	2.70	2.63	0.851527151	-0.11
C7_00320C_A	16.15	16.28	16.25	15.22	17.11	16.47	0.598119068	-0.11
C1_12170C_A	12.60	17.04	17.38	12.40	17.32	17.63	0.78468268	-0.11
C3_01480C_A	10.79	4.92	11.21	6.13	10.40	10.27	0.86183583	-0.11
C2_06230W_A	20.88	20.49	19.35	19.97	20.31	20.56	0.536679563	-0.11
C1_00320W_A	32.89	29.64	25.90	29.72	27.83	30.94	0.631757976	-0.11
C1_07020C_A	12.63	9.68	10.92	10.11	13.47	9.50	0.748561905	-0.11
C5_02770W_A	6.13	4.66	5.30	4.74	5.26	6.08	0.73369471	-0.11
C6_03340C_A	74.31	69.97	84.88	55.61	88.20	85.87	0.763016139	-0.11
C2_03230C_A	12.89	17.97	17.04	15.85	10.19	22.47	0.833788509	-0.11
C1_05850W_A	25.26	24.02	13.41	22.01	20.48	20.08	0.798432646	-0.11
CR_04720C_A	9.83	6.83	7.01	7.65	7.60	8.33	0.687688703	-0.11
C4_06460C_A	8.29	5.61	4.19	5.72	6.87	5.35	0.832462269	-0.11
C1_00750C_A	50.10	44.23	31.32	40.70	40.45	44.31	0.726909443	-0.11
CR_07570W_A	21.00	16.91	18.06	17.27	17.49	21.16	0.655343968	-0.11
CR_10490W_A	73.88	105.46	236.01	121.17	136.63	162.20	0.872320584	-0.11
C2_03410W_A	18.07	25.12	22.27	24.33	22.16	19.20	0.751777226	-0.11
C1_12080W_A	12.46	13.68	13.69	14.16	12.11	13.58	0.650919261	-0.11
C5_03120W_A	3.32	3.10	3.78	2.46	4.29	3.42	0.8095412	-0.11
C1_12450C_A	1.83	2.16	1.67	2.08	1.55	2.04	0.736967998	-0.11
C6_00090W_A	17.12	13.42	15.51	14.11	15.67	16.10	0.713581314	-0.11
C1_03030W_A	712.48	622.26	769.39	736.94	692.82	668.30	0.608964038	-0.11
C3_04580C_A	125.16	102.36	127.24	100.41	160.09	90.97	0.790602447	-0.11
C1_04740W_A	30.45	26.12	28.93	24.10	26.99	34.35	0.676189237	-0.11
C4_00500W_A	8.87	3.13	3.51	3.06	3.72	8.61	0.888938131	-0.11
C1_02930C_A	5.85	11.52	10.36	8.83	9.54	9.53	0.821322431	-0.11
C5_03830C_A	11.94	7.21	10.94	8.02	10.84	10.96	0.783591659	-0.12
C5_00730W_A	39.35	38.00	41.21	40.32	38.31	39.64	0.502972928	-0.12
C1_01050C_A	16.80	17.10	18.09	18.27	14.67	19.06	0.664129737	-0.12
CR_06510W_A	9.97	5.95	6.11	4.35	6.89	10.64	0.857484445	-0.12
C4_00110C_A	13.89	12.11	11.78	13.25	17.35	6.64	0.842303228	-0.12
CR_03070W_A	36.10	37.78	38.51	42.17	34.35	35.51	0.63597279	-0.12
C3_07410C_A	84.91	84.88	90.02	94.68	84.07	79.84	0.600222697	-0.12
C2_03320W_A	14.22	12.00	9.98	11.19	11.83	12.94	0.644522865	-0.12
C2_09250W_A	16.23	17.72	15.67	20.61	15.08	13.73	0.752941706	-0.12
C5_02840C_A	12.64	7.98	9.30	9.48	9.62	10.50	0.687009307	-0.12
C7_03730C_A	13.81	15.99	8.53	11.47	12.96	13.74	0.777016343	-0.12
C2_03030W_A	4.80	7.89	6.54	8.43	4.50	6.35	0.817558775	-0.12
CR_10360C_A	150.34	120.59	132.22	106.16	156.06	137.20	0.695413259	-0.12
C5_03690W_A	5.63	5.38	6.11	7.24	4.64	5.16	0.761067222	-0.12
C2_00960C_A	87.06	118.22	111.83	128.85	93.55	94.25	0.751256312	-0.12
CR_02690W_A	9.73	5.94	8.99	9.94	2.93	11.79	0.879113853	-0.12
CR_06050W_A	12.95	15.99	15.74	15.92	13.63	14.97	0.695848411	-0.12
CR_07530C_A	14.70	11.64	11.25	9.70	13.95	13.59	0.712036614	-0.12
CR_02110W_A	8.61	10.02	7.79	9.61	8.14	8.57	0.67842811	-0.12
C2_03180C_A	26.86	21.96	22.84	20.60	22.21	28.43	0.631415419	-0.12
CR_04420C_A	9.63	7.47	3.53	7.97	9.20	3.01	0.89110748	-0.12
C5_04350C_A	13.89	12.95	17.69	15.15	13.67	15.44	0.698345248	-0.12

C1_12520W_A	3.96	1.94	1.71	2.15	2.28	3.06	0.833998519	-0.12
CR_10290C_A	4.30	4.26	4.43	4.93	3.66	4.33	0.65815551	-0.12
C6_00320C_A	2.67	2.25	2.55	2.35	2.39	2.66	0.610044354	-0.12
C2_08010W_A	10.41	10.03	9.86	11.03	8.89	10.15	0.557653351	-0.12
C2_06010W_A	10.83	4.45	8.73	7.09	7.16	9.40	0.808216419	-0.12
C5_02640W_A	15.74	11.85	12.99	12.97	13.55	13.58	0.654365709	-0.12
C1_06130C_A	16.79	33.79	33.74	25.53	28.83	30.19	0.811533978	-0.12
C1_10940C_A	67.08	87.77	100.24	91.68	73.04	89.79	0.728687779	-0.12
CR_03060W_A	131.13	94.04	95.89	97.63	110.16	108.73	0.626207521	-0.12
C1_11650W_A	464.22	384.36	545.69	697.11	342.83	337.37	0.816398755	-0.12
C1_06920C_A	5.77	6.39	6.87	6.06	5.83	6.99	0.67766743	-0.13
CR_08470W_A	8.21	10.77	9.66	10.97	9.65	7.78	0.744931047	-0.13
C1_08130C_A	4.72	6.59	8.13	7.09	7.09	5.11	0.788291787	-0.13
C5_00690C_A	7.89	3.44	4.12	4.47	5.18	5.53	0.818161174	-0.13
C3_04320W_A	12.91	13.15	11.80	14.53	10.78	12.18	0.674077504	-0.13
C1_03990W_A	13.01	7.96	5.66	6.86	9.04	10.27	0.815588377	-0.13
C4_02590C_A	3.68	5.98	3.76	5.77	3.53	4.07	0.804783189	-0.13
C5_03590W_A	21.50	22.30	21.04	20.46	20.98	22.72	0.505489737	-0.13
C1_10510W_A	7.25	6.28	8.81	6.34	8.62	7.08	0.763230412	-0.13
C4_01660W_A	2.76	2.95	2.67	2.86	2.43	3.00	0.641853429	-0.13
CR_04310C_A	24.60	25.35	30.54	23.10	26.78	29.84	0.671250887	-0.13
C1_13330C_A	21.13	29.09	28.32	41.30	25.47	10.68	0.864879132	-0.13
C4_00170W_A	385.26	528.40	510.52	489.13	464.13	461.22	0.679379765	-0.13
CR_09790W_A	38.08	40.30	44.87	49.62	28.53	44.20	0.736858858	-0.13
C3_02580C_A	9.76	8.18	7.27	7.75	8.44	8.69	0.677150749	-0.13
C1_02000W_A	10.88	13.67	13.29	14.47	10.64	12.39	0.709506112	-0.13
C2_08910C_A	11.24	8.48	9.66	8.63	9.35	11.02	0.6292726	-0.13
C2_10430C_A	2.97	1.97	2.09	0.48	2.05	4.45	0.903153622	-0.13
C2_02520W_A	8.05	7.85	6.38	5.73	7.96	8.27	0.701910171	-0.13
C4_05960W_A	32.19	29.29	30.99	34.08	25.31	31.87	0.603626576	-0.13
C2_07400C_A	163.86	233.58	167.62	217.71	194.01	146.58	0.749285583	-0.13
C2_05000C_A	10.54	8.93	10.98	9.24	11.09	9.58	0.663061214	-0.13
C3_01860C_A	38.59	25.75	41.27	41.80	33.48	28.24	0.743045508	-0.13
C4_06940C_A	37.49	34.07	30.45	33.92	34.79	31.67	0.551615068	-0.13
C2_07250C_A	3.42	5.41	1.48	4.61	4.75	0.70	0.91250151	-0.13
C1_07900W_A	41.89	36.24	41.79	32.39	44.51	40.97	0.642029234	-0.13
C2_08990C_A	14.09	13.40	12.82	15.26	11.01	13.44	0.604950341	-0.13
C5_05030C_A	9.85	7.68	6.70	7.48	7.64	8.65	0.640100882	-0.13
C3_06970W_A	3.98	2.50	2.32	2.11	3.15	3.35	0.779871131	-0.14
C1_14310W_A	25.62	25.65	26.85	23.95	27.66	25.10	0.550617489	-0.14
CR_08070W_A	35.73	29.93	31.29	29.94	33.09	32.08	0.484662977	-0.14
C3_07500W_A	35.42	38.57	38.29	53.38	29.80	27.16	0.772936322	-0.14
C7_01290W_A	4.14	1.50	1.24	0.54	1.91	4.26	0.915904988	-0.14
C4_04920W_A	22.12	21.82	20.54	22.08	22.03	19.17	0.563803695	-0.14
C7_00780W_A	4.72	2.83	2.62	2.26	3.61	4.08	0.790645162	-0.14
C2_03900C_A	26.95	20.77	19.46	20.63	19.96	25.28	0.60162417	-0.14
C7_02670W_A	3.23	1.85	2.03	2.10	2.19	2.67	0.742042563	-0.14
C1_07780W_A	12.74	8.33	7.35	9.37	8.66	9.70	0.69614493	-0.14
C4_02010C_A	0.65	0.53	0.72	0.69	0.43	0.74	0.834538704	-0.14
C6_01860C_A	16.71	23.10	24.35	21.30	17.37	24.61	0.732362304	-0.14
C1_08140W_A	9.15	11.25	10.13	10.58	12.67	6.57	0.777876204	-0.14
C7_00190W_A	101.74	57.42	68.58	66.29	78.58	76.64	0.695322038	-0.14
CR_10720W_A	11.38	11.35	11.88	12.34	9.99	11.66	0.547593539	-0.14
C6_00510C_A	9.66	7.02	7.83	9.09	8.06	6.77	0.668890573	-0.14
C6_00600C_A	30.98	31.71	44.61	38.21	34.03	33.16	0.681862398	-0.14
C4_04370C_A	4.93	4.10	4.40	4.30	4.43	4.40	0.550327473	-0.14
C1_13520C_A	4.85	6.05	9.61	5.99	6.98	7.26	0.788054834	-0.14
C4_00470C_A	38.57	21.14	20.16	23.19	20.62	34.05	0.766867625	-0.14
C1_11360W_A	1522.43	1314.70	1404.19	1415.03	1515.14	1208.58	0.556950862	-0.14
C6_01370W_A	9.31	6.01	5.73	5.32	5.96	9.29	0.760390733	-0.14
C3_07350W_A	54.95	59.86	57.36	49.72	59.99	59.18	0.55116661	-0.14
C5_01780W_A	28.27	29.36	28.35	27.11	27.88	29.29	0.438652747	-0.14
CR_00370W_A	9.88	8.23	8.52	8.91	8.42	8.73	0.468536817	-0.14

C4_02980W_A	60.43	65.40	74.15	73.48	58.04	64.79	0.600736389	-0.14
C5_00980W_A	22.46	16.48	21.80	21.62	20.36	17.19	0.636240978	-0.14
C6_03080C_A	54.10	40.69	52.83	43.54	49.52	51.06	0.562001572	-0.14
C2_03700W_A	24.71	21.07	16.82	20.68	20.16	20.25	0.605776908	-0.14
C2_00530W_A	7.90	2.14	8.17	0.94	8.90	7.81	0.913793991	-0.14
C2_03800C_A	21.78	18.22	19.22	19.17	18.10	20.58	0.415428574	-0.14
C2_08040C_A	1133.27	1029.07	1079.12	1056.77	1056.54	1049.83	0.311774832	-0.15
C6_00300C_A	36.70	28.33	30.88	30.87	31.16	31.34	0.444666595	-0.15
C1_09940W_A	10.37	8.56	8.95	6.52	11.00	9.65	0.764159369	-0.15
CR_08570W_A	30.35	27.33	27.26	26.62	27.61	28.63	0.328249784	-0.15
C2_06040C_A	2.84	2.93	2.52	3.38	2.17	2.58	0.736967998	-0.15
C7_00210C_A	6.56	6.42	5.79	5.85	6.29	6.19	0.512447035	-0.15
C1_11040W_A	892.83	657.48	902.24	765.32	896.67	719.06	0.618617961	-0.15
C2_07650C_A	12.99	18.88	20.04	14.55	19.47	16.81	0.715872549	-0.15
C2_09300W_A	16.54	18.71	18.64	16.79	16.35	19.52	0.555807332	-0.15
C3_07730W_A	73.67	64.14	58.03	59.16	70.36	60.69	0.53947733	-0.15
C1_02440C_A	7.19	7.07	8.97	8.33	6.55	7.81	0.65568262	-0.15
CR_01930C_A	229.45	351.47	256.17	257.01	284.76	279.20	0.6730093	-0.15
C3_01680C_A	7.03	4.19	4.08	4.10	5.23	5.47	0.740883388	-0.15
C3_00520W_A	37.17	35.25	31.13	25.20	29.88	46.19	0.706276212	-0.15
C5_04710W_A	14.58	10.43	12.45	13.73	10.38	12.31	0.610598916	-0.15
C7_03380W_A	12.51	11.02	7.56	8.45	8.37	13.42	0.773188104	-0.15
C3_07140C_A	2.38	1.40	1.84	2.31	1.40	1.76	0.767875751	-0.15
C4_04900W_A	281.53	382.58	462.47	433.39	324.34	346.03	0.710863726	-0.15
C1_13260W_A	52.74	66.93	82.25	74.63	54.96	68.22	0.697535817	-0.15
C1_03270W_A	22.24	23.95	29.26	24.42	24.75	24.35	0.590489434	-0.15
C5_04980W_A	1.04	2.09	1.54	1.37	1.94	1.23	0.817839698	-0.15
C1_03390W_A	53.72	56.91	61.89	59.11	53.28	55.84	0.487424201	-0.15
C2_06390C_A	27.13	24.63	35.35	32.43	24.85	27.51	0.646111412	-0.15
C3_04420W_A	26.35	24.86	22.72	24.17	24.68	22.97	0.432620884	-0.15
C1_07130C_A	10.04	5.91	8.68	10.30	9.53	3.78	0.819258743	-0.15
C1_03110W_A	550.27	585.62	618.82	615.70	589.77	501.27	0.552150976	-0.15
C1_00890W_A	10.21	7.11	7.46	8.00	6.73	9.34	0.627140534	-0.15
C6_04650W_A	95.10	66.07	86.25	27.75	106.87	105.39	0.83502457	-0.15
C7_03970C_A	26.02	66.80	38.78	39.14	26.24	65.35	0.831110678	-0.15
C2_03310C_A	78.57	63.07	65.56	73.99	56.64	70.29	0.525642363	-0.15
C1_07210C_A	19.94	18.60	19.15	13.51	23.27	19.14	0.67557731	-0.15
C1_01530C_A	69.61	33.41	67.49	57.24	52.24	54.82	0.726909443	-0.15
C2_01300C_A	8.80	5.35	6.24	5.99	6.52	7.18	0.645399423	-0.15
C4_06060W_A	9.89	7.98	6.75	8.14	6.37	9.38	0.621907968	-0.15
C2_07600C_A	26.51	26.50	27.99	28.38	26.82	23.33	0.517823695	-0.16
C4_04240C_A	24.29	18.19	32.32	26.37	24.40	21.61	0.691606769	-0.16
C3_03340C_A	79.36	58.28	85.89	62.57	75.35	78.38	0.621937985	-0.16
C5_00500W_A	11.95	10.83	10.31	9.95	12.43	9.60	0.567521711	-0.16
C6_03430C_A	4.76	4.84	4.55	4.54	4.08	5.11	0.473033633	-0.16
C4_02200C_A	8.60	11.64	7.03	9.11	7.50	9.90	0.738559563	-0.16
C1_02680C_A	4.91	5.07	6.02	7.06	5.39	2.98	0.774913805	-0.16
CR_07370W_A	24.47	36.80	45.92	44.84	28.29	31.33	0.760695035	-0.16
C2_09100C_A	2.30	2.82	2.47	2.69	1.81	2.88	0.70031165	-0.16
C3_04670C_A	728.93	707.30	827.11	757.23	750.48	683.31	0.472786156	-0.16
C2_09900C_A	3.85	4.45	7.00	5.45	3.14	6.25	0.79921257	-0.16
C4_05630W_A	58.17	57.28	50.53	72.72	18.22	71.10	0.828850625	-0.16
C1_08620W_A	31.46	39.73	39.95	36.83	37.83	33.05	0.612859053	-0.16
C2_08590W_A	383.88	450.37	504.93	500.15	354.66	447.85	0.616487208	-0.16
CR_03330W_A	30.05	24.42	23.30	28.55	23.57	22.88	0.552004669	-0.16
C7_02980C_A	8.74	6.89	8.95	8.19	7.77	7.76	0.4995327	-0.16
C2_01470W_A	8.04	6.45	7.16	6.85	7.18	6.85	0.416854736	-0.16
C1_12920C_A	19.06	19.85	23.77	18.37	19.54	22.88	0.570996853	-0.16
C2_00220C_A	58.01	46.59	43.15	44.39	50.15	47.84	0.490450014	-0.16
C1_05520W_A	12.20	8.24	8.03	8.67	8.96	9.80	0.598775665	-0.16
C1_06910C_A	13.56	11.43	10.16	12.09	10.13	11.69	0.503826314	-0.16
C1_05810W_A	15.04	19.83	24.85	22.46	17.96	17.52	0.712921372	-0.16
C4_05680W_A	22.38	24.15	20.58	20.42	18.90	25.80	0.538961242	-0.16

C2_10750C_A	50.86	30.77	34.86	36.97	30.10	45.10	0.652706972	-0.16
C5_05070W_A	5.45	4.12	3.99	4.08	4.46	4.52	0.563449865	-0.16
CR_03990C_A	36.00	32.17	34.06	33.39	31.00	34.28	0.304427824	-0.16
C1_11430W_A	1.64	1.89	1.90	1.43	2.39	1.41	0.755178621	-0.16
C6_01000C_A	3.32	2.43	3.02	3.14	2.35	3.00	0.67163997	-0.16
C1_03620C_A	25.72	4.13	45.64	29.10	19.50	23.81	0.879985789	-0.16
C7_03150W_A	1.09	0.80	0.78	1.03	0.67	0.87	0.754373201	-0.16
C2_09260C_A	57.08	70.76	64.78	62.31	63.30	60.67	0.522291864	-0.16
C4_02720C_A	9.50	30.22	8.99	4.67	20.35	22.85	0.876948192	-0.16
C6_00790C_A	183.64	115.96	239.03	184.47	170.68	162.35	0.702235402	-0.16
C4_05350W_A	12.85	11.53	10.55	8.61	10.59	14.48	0.629953434	-0.17
C1_06720C_A	9.04	10.82	13.55	10.46	11.29	10.51	0.640510902	-0.17
C4_05100C_A	60.86	43.81	42.25	41.18	50.06	49.50	0.547220766	-0.17
CR_03020C_A	22.79	20.07	24.60	21.41	20.08	23.38	0.466217816	-0.17
C2_03680W_A	3.86	12.81	14.40	12.79	8.75	8.77	0.83216027	-0.17
C5_04970C_A	0.68	0.49	0.32	0.30	0.53	0.60	0.82313902	-0.17
CR_06520C_A	5.51	2.56	0.96	1.77	3.25	3.57	0.868378273	-0.17
C7_03800W_A	2.10	3.08	1.15	2.21	1.48	2.47	0.83242468	-0.17
C7_02350C_A	1.88	2.88	2.63	3.26	1.72	2.16	0.756726185	-0.17
CR_02560C_A	7.90	5.04	5.34	5.72	5.86	5.90	0.586283556	-0.17
C5_00830C_A	32.45	36.17	34.86	34.23	30.45	35.08	0.432814008	-0.17
C4_05860W_A	20.46	19.77	34.12	21.49	23.06	27.12	0.707198016	-0.17
C1_06040W_A	3.03	1.91	1.26	1.76	1.81	2.36	0.758215047	-0.17
C1_04340C_A	3.08	3.59	2.75	1.82	2.92	4.31	0.773913736	-0.17
CR_08060C_A	146.89	151.82	168.04	144.34	161.72	142.34	0.460495493	-0.17
C1_11600W_A	23.40	15.46	22.62	13.40	24.95	20.36	0.713305457	-0.17
C2_08850C_A	34.04	37.29	35.44	33.59	31.40	37.82	0.426500142	-0.17
CR_10250C_A	663.71	769.51	731.91	707.35	709.15	666.94	0.443630491	-0.17
C7_00800C_A	19.71	12.86	13.21	1.99	19.71	22.15	0.87868889	-0.17
C7_03480W_A	6.56	12.47	6.35	7.50	8.67	8.41	0.736902074	-0.17
C3_06630W_A	1.46	1.31	0.93	1.26	1.00	1.29	0.710863726	-0.17
C2_06760C_A	4.89	2.92	3.37	3.56	3.42	3.73	0.663719429	-0.17
C2_09120C_A	1.33	0.74	0.90	1.16	0.68	1.02	0.82244987	-0.17
CR_02770C_A	15.51	14.24	13.25	13.58	13.75	13.87	0.399479643	-0.17
C2_04060C_A	12.67	20.14	10.71	10.33	14.13	17.69	0.734765139	-0.17
C2_00230W_A	22.56	19.67	18.79	18.36	21.93	18.04	0.490187801	-0.17
C6_03620C_A	13.34	11.62	12.10	11.84	10.48	13.21	0.422720342	-0.17
C5_02400W_A	46.38	78.02	87.23	86.81	55.70	62.05	0.729125471	-0.17
C5_01880C_A	32.96	27.56	18.03	21.77	19.86	33.67	0.707725084	-0.17
C7_04150W_A	3.61	4.11	3.73	1.94	4.61	4.44	0.752273279	-0.17
C2_08900W_A	54.89	141.12	165.93	190.66	80.46	79.33	0.830498559	-0.17
C5_04590C_A	638.22	928.36	857.75	901.41	766.39	663.39	0.636548416	-0.17
C1_12240C_A	7.15	6.08	7.28	5.34	7.46	6.77	0.581482155	-0.17
C2_05210W_A	18.35	15.18	21.98	19.84	16.35	16.85	0.619096753	-0.17
CR_05020W_A	2.85	2.09	2.62	2.22	2.05	2.98	0.65483529	-0.17
C3_06470W_A	25.32	23.79	20.69	24.46	21.71	20.49	0.455001322	-0.18
C1_09180W_A	2.94	2.44	2.05	2.34	2.64	2.11	0.615652532	-0.18
CR_04200W_A	16.71	32.02	28.73	29.81	24.49	20.32	0.726909443	-0.18
CR_00140W_A	6.33	5.81	0.70	7.12	3.98	0.98	0.893857522	-0.18
CR_06070W_A	30.43	29.33	28.23	29.01	26.40	28.65	0.298970719	-0.18
C7_02680W_A	14.96	6.13	8.11	6.67	9.98	10.97	0.769268255	-0.18
C1_02370C_A	4.98	3.30	5.07	2.96	4.54	5.22	0.680174491	-0.18
CR_04640W_A	2.86	1.87	1.30	1.72	1.74	2.26	0.7487803	-0.18
C2_01870C_A	5.44	3.56	3.60	3.70	3.38	4.94	0.661577566	-0.18
C4_01680W_A	15.12	8.20	7.88	6.38	9.74	13.50	0.767875751	-0.18
CR_06300C_A	19.79	16.19	18.69	16.86	18.51	16.72	0.453752359	-0.18
C3_05880C_A	39.67	56.12	30.26	35.46	35.15	50.75	0.688225228	-0.18
C2_09740W_A	71.65	63.95	66.59	67.75	62.82	62.14	0.294158507	-0.18
C5_01640W_A	6.01	5.43	5.69	4.68	4.29	7.41	0.639124587	-0.18
C1_04780C_A	9.41	8.66	8.09	8.10	9.08	7.72	0.436692845	-0.18
C1_14380C_A	4.66	6.74	6.11	7.17	7.62	1.80	0.827505705	-0.18
C1_00120C_A	44.06	48.15	61.04	44.72	48.34	53.52	0.544436215	-0.18
C2_06080C_A	19.75	27.46	21.73	23.01	20.20	22.80	0.566948619	-0.18

C1_00390W_A	6.63	6.89	7.11	5.54	6.86	7.26	0.474167762	-0.18
C2_09430W_A	1231.18	1046.94	1082.26	1003.28	1098.30	1093.13	0.249458324	-0.18
C5_02830W_A	30.04	23.95	27.86	25.79	27.52	24.41	0.423224299	-0.18
C3_07850W_A	29.15	30.09	35.80	36.90	26.95	26.72	0.573861293	-0.18
C4_04670C_A	36.13	20.09	12.97	16.37	17.63	31.45	0.767297272	-0.18
C3_02570W_A	3.97	4.21	3.58	3.61	3.18	4.41	0.544010297	-0.18
C3_06650C_A	8.52	8.68	13.46	10.41	9.26	9.53	0.631415419	-0.18
C4_04210C_A	7.90	2.43	5.12	7.73	3.17	3.72	0.829619822	-0.18
C4_01270W_A	527.71	671.87	779.00	650.75	407.90	845.17	0.689169113	-0.18
C2_05300C_A	7.04	11.09	12.54	10.25	8.89	10.24	0.678559173	-0.18
C1_07330W_A	3.04	1.63	2.12	3.20	1.69	1.49	0.772976554	-0.19
C1_04520C_A	66.40	70.05	76.55	70.85	68.21	63.38	0.409191075	-0.19
C2_10230W_A	2.46	1.16	1.52	1.46	1.17	2.24	0.762878483	-0.19
C6_02680W_A	10.73	4.13	4.57	4.31	7.27	6.64	0.77693342	-0.19
C2_08510W_A	1.34	1.56	0.81	1.20	1.73	0.59	0.839079895	-0.19
C1_11990W_A	49.14	55.14	51.34	59.07	21.97	68.12	0.757441424	-0.19
C1_05140W_A	78.45	67.75	53.04	49.33	83.76	54.54	0.653518731	-0.19
C2_10330C_A	7.07	7.61	7.34	8.32	5.93	6.61	0.629657996	-0.19
C3_00690C_A	11.58	13.78	13.81	14.02	11.26	11.96	0.514636188	-0.19
C1_00070W_A	159.33	130.17	121.68	116.30	134.27	138.19	0.382670556	-0.19
C3_06020W_A	21.28	21.98	21.24	31.77	14.40	14.86	0.724130233	-0.19
C1_00730C_A	22.48	20.94	15.59	17.56	17.74	20.60	0.487424201	-0.19
C3_02170C_A	52.00	64.91	59.88	56.25	51.80	60.18	0.441300195	-0.19
C1_07010W_A	13.05	8.53	7.46	7.55	13.56	5.99	0.752188648	-0.19
C2_06110W_A	51.79	14.17	26.51	36.40	10.74	39.59	0.826124698	-0.19
C3_07740W_A	46.20	34.03	40.20	39.33	38.31	35.85	0.385986562	-0.19
C2_06170C_A	32.73	17.46	31.40	24.40	21.25	31.34	0.642940721	-0.19
C3_02760C_A	14.70	11.49	11.04	12.18	11.39	11.52	0.378990767	-0.19
C5_03160W_A	2.25	1.61	1.31	1.21	1.94	1.71	0.674276721	-0.19
CR_04030W_A	12.86	10.90	11.16	12.31	9.42	11.30	0.401489742	-0.19
C2_01240C_A	1.71	1.46	1.59	1.21	1.65	1.61	0.595296903	-0.19
C1_11060C_A	1274.55	1360.93	1460.31	1283.49	1330.95	1262.84	0.338298258	-0.19
C1_09810W_A	11.92	16.69	6.32	11.17	11.36	10.56	0.728664586	-0.19
C2_00920W_A	23.30	14.15	14.67	14.89	14.47	19.64	0.606966264	-0.19
C4_00600C_A	64.97	50.28	41.69	44.98	52.31	50.06	0.488911904	-0.20
C1_13690C_A	215.09	217.15	216.12	194.20	198.04	220.33	0.238062961	-0.20
C1_10280C_A	89.88	118.53	13.89	105.87	20.93	84.57	0.867736307	-0.20
C6_00620W_A	5.01	12.04	11.71	10.95	7.17	9.26	0.766137988	-0.20
C1_11550W_A	85.47	50.11	91.77	69.71	81.23	61.80	0.631415419	-0.20
C4_07160W_A	5.70	7.34	12.62	8.46	9.81	5.68	0.794146923	-0.20
C2_07560W_A	11.13	5.51	7.26	7.19	5.76	9.47	0.657288045	-0.20
C1_09520C_A	11.52	9.99	10.36	11.85	6.56	11.72	0.616487208	-0.20
C6_00720C_A	57.19	60.22	71.07	63.40	59.12	55.24	0.432274068	-0.20
C4_07110C_A	659.41	539.99	493.19	213.64	685.41	693.25	0.75829345	-0.20
C1_01800W_A	20.78	17.00	18.85	18.59	18.27	16.37	0.406542659	-0.20
C2_05610C_A	1250.29	1020.86	1185.56	1109.51	1112.03	1019.13	0.288015921	-0.20
CR_05640C_A	5.20	5.30	5.94	4.98	5.57	4.90	0.596706857	-0.20
C3_00860W_A	51.84	47.34	56.38	37.91	34.51	75.11	0.673161698	-0.20
CR_01360W_A	14.34	5.70	7.67	9.70	7.09	9.01	0.723555571	-0.20
C1_01280C_A	2.90	3.23	3.64	0.73	4.74	3.58	0.843936103	-0.20
C1_03120W_A	95.19	86.98	88.43	86.34	79.50	88.55	0.154995748	-0.20
CR_06680C_A	2.58	2.11	1.88	2.32	1.84	2.01	0.518082342	-0.20
C5_05000C_A	11.34	12.61	11.13	11.80	10.57	10.68	0.377867952	-0.20
C7_02110W_A	38.49	30.08	4.84	25.85	27.14	14.88	0.835914261	-0.20
C4_00460C_A	13.99	8.30	8.33	8.34	9.06	11.21	0.58253824	-0.20
CR_06230W_A	4.80	8.12	6.57	5.86	5.62	7.00	0.624437078	-0.20
CR_08840C_A	14.51	22.39	21.58	19.25	19.06	16.86	0.598661218	-0.20
C4_01670C_A	10.97	5.63	7.51	5.81	7.24	9.47	0.659824972	-0.20
C4_03860C_A	3.77	2.95	2.80	2.55	1.46	4.97	0.772936322	-0.20
C1_06680W_A	5.89	5.00	4.48	4.84	4.24	5.35	0.415451844	-0.20
CR_00490W_A	16.41	16.43	20.65	4.02	22.61	23.80	0.818404244	-0.20
C2_08160C_A	36.17	19.57	20.68	15.86	22.89	32.59	0.698345248	-0.20
C2_05660W_A	104.15	112.66	104.18	100.71	102.83	97.87	0.272064856	-0.20

C5_01550C_A	240.21	203.56	232.41	214.84	223.13	194.43	0.311070296	-0.20
C4_06010C_A	13.62	13.57	10.98	12.66	11.91	11.20	0.423282513	-0.20
C5_05260W_A	14.98	15.28	16.60	17.10	13.46	13.42	0.491753936	-0.20
C5_02480W_A	46.03	40.57	36.22	38.20	38.78	37.93	0.301750438	-0.20
C1_08530W_A	20.90	12.59	15.97	13.65	17.48	14.88	0.581307271	-0.21
C4_00190W_A	3.03	3.41	4.29	3.21	3.41	3.41	0.618201358	-0.21
CR_06030C_A	5.59	4.11	5.22	4.62	4.62	4.68	0.413388604	-0.21
C2_08740W_A	3.58	1.55	4.80	2.83	2.62	3.84	0.752188648	-0.21
C1_09230C_A	17.26	20.63	17.67	21.37	13.79	17.02	0.545464675	-0.21
C4_04780W_A	21.84	43.99	36.99	34.88	31.59	30.72	0.653437599	-0.21
C1_13860C_A	576.55	821.07	782.08	735.17	677.05	638.38	0.513893181	-0.21
C2_02490C_A	3.47	4.01	7.82	2.54	5.25	6.68	0.76228877	-0.21
C2_01030W_A	28.29	32.38	31.93	31.35	27.64	27.76	0.403638577	-0.21
C7_01970C_A	96.60	43.98	46.27	55.13	60.16	56.94	0.654365709	-0.21
C2_08240W_A	28.71	23.97	30.62	25.72	26.95	25.04	0.327007791	-0.21
C1_01790W_A	14.37	11.46	11.64	12.25	11.39	11.28	0.298970719	-0.21
C7_00180W_A	2.31	0.00	1.74	1.15	1.44	1.10	0.940095415	-0.21
C2_02670C_A	10.93	4.36	10.42	12.15	2.46	9.34	0.821539034	-0.21
C2_09130C_A	18.30	18.25	15.87	16.52	16.88	15.53	0.295521236	-0.21
C5_02570W_A	2.28	1.05	2.07	1.40	0.66	2.99	0.806286789	-0.21
C5_04290C_A	11.44	14.97	14.69	13.57	11.09	13.90	0.496384514	-0.21
C1_03130C_A	64.85	60.47	61.53	57.46	56.36	60.51	0.113618735	-0.21
C6_00180C_A	5.31	7.05	8.13	8.39	4.74	6.00	0.68093953	-0.21
C4_00040W_A	40.77	32.75	31.08	33.61	34.15	29.31	0.365753338	-0.21
C1_10650W_A	1.59	1.25	0.97	1.23	1.63	0.69	0.761265511	-0.21
C6_04050W_A	1.75	1.90	1.59	1.32	2.02	1.54	0.664325155	-0.21
C3_04410C_A	17.93	21.36	20.48	19.74	17.38	18.77	0.356491105	-0.21
C1_02720W_A	16.00	13.36	3.97	12.34	8.88	9.61	0.767875751	-0.21
C4_06160W_A	4.97	3.91	4.14	3.79	3.72	4.62	0.449446786	-0.21
C1_08660C_A	8.27	11.45	12.29	11.69	8.43	9.82	0.5951257	-0.21
CR_08700C_A	109.49	29.47	110.50	74.00	147.79	3.30	0.917598607	-0.21
C5_05220W_A	0.48	0.34	1.10	0.47	0.60	0.71	0.820847888	-0.22
C4_00850C_A	0.41	0.78	0.55	0.54	0.19	0.91	0.826350516	-0.22
C2_09650W_A	243.46	216.88	234.51	229.93	190.78	225.98	0.221399826	-0.22
C2_03820C_A	519.51	618.63	666.94	621.36	519.46	545.04	0.416854736	-0.22
C4_06660W_A	65.02	34.67	36.98	36.05	43.03	46.93	0.580386603	-0.22
C1_11170W_A	43.39	65.52	58.25	60.18	46.89	49.40	0.543791448	-0.22
C1_14550C_A	35.33	28.22	29.28	43.58	17.44	25.20	0.689091422	-0.22
C4_00750C_A	3.32	2.49	4.24	1.54	2.46	5.36	0.791357494	-0.22
C4_05080C_A	5.27	5.26	4.23	4.76	4.32	4.63	0.403286376	-0.22
C1_01640W_A	118.03	133.42	151.09	144.17	114.70	116.18	0.442654561	-0.22
C4_01830C_A	18.26	21.45	15.93	23.13	19.38	8.92	0.705058473	-0.22
C1_08630W_A	7.95	6.61	6.04	6.50	6.17	6.44	0.390672089	-0.22
C5_01340W_A	13.99	12.33	12.67	11.67	12.15	12.38	0.227745947	-0.22
C1_03200C_A	3.54	3.04	2.80	3.39	2.97	2.34	0.514636188	-0.22
C3_01880W_A	4.44	2.06	2.52	2.68	2.27	3.39	0.671621159	-0.22
C3_03770C_A	44.57	45.35	36.88	45.08	39.11	33.31	0.446512782	-0.22
C6_02240C_A	346.19	566.48	71.66	338.86	275.34	304.73	0.81276417	-0.22
C6_00660C_A	40.75	47.14	48.23	44.61	38.33	43.82	0.384865354	-0.22
C7_00740W_A	18.11	14.38	12.90	12.79	14.50	14.69	0.345769399	-0.22
C5_03740W_A	10.60	7.93	9.25	7.14	8.01	10.55	0.503228722	-0.22
C1_10750C_A	37.59	40.97	42.34	36.74	37.04	38.48	0.328655546	-0.22
C1_12270W_A	2.29	1.58	2.33	1.78	2.23	1.70	0.663719429	-0.22
C6_01260W_A	19.33	14.58	17.90	17.24	15.19	15.55	0.438652747	-0.22
CR_03860C_A	5.95	5.98	4.87	5.34	4.49	5.79	0.430545092	-0.22
C2_10590W_A	216.35	175.61	179.70	156.50	198.50	172.90	0.33389939	-0.22
C2_06730W_A	125.20	22.41	133.25	59.90	5.42	198.08	0.916340993	-0.22
C6_00430C_A	18.14	17.34	15.84	19.96	14.88	12.60	0.510324269	-0.22
C4_04580W_A	17.06	4.49	11.61	15.79	2.44	12.23	0.824393957	-0.22
C5_04700C_A	60.97	23.67	29.60	35.92	32.37	36.00	0.658584368	-0.22
C4_00760W_A	26.86	26.92	36.96	32.52	24.75	26.93	0.486254529	-0.22
C1_13650C_A	15.53	11.84	12.25	11.87	11.00	13.77	0.35035169	-0.22
C6_01820C_A	28.78	29.81	27.57	26.74	26.26	26.82	0.216085808	-0.22

C1_04150C_A	2.22	1.45	1.01	0.82	1.74	1.70	0.786477168	-0.22
C1_12410C_A	41.25	28.11	27.06	30.32	26.43	32.11	0.422562624	-0.22
C6_03520C_A	7.55	7.94	5.08	4.47	6.13	8.51	0.618617961	-0.22
CR_09440C_A	49.27	90.09	54.75	67.08	57.29	56.74	0.609809495	-0.22
C5_01540W_A	548.72	696.84	773.70	764.63	563.05	547.79	0.525567482	-0.22
C2_00770W_A	54.40	66.25	84.68	84.68	17.46	90.81	0.785116743	-0.22
CR_06060W_A	30.17	22.18	29.80	21.34	29.01	25.27	0.45922845	-0.22
C2_08570W_A	6.46	4.43	5.83	6.27	3.76	5.40	0.57166533	-0.22
CR_00690C_A	3.06	11.47	8.14	1.87	10.63	8.80	0.830214397	-0.22
C2_00930C_A	59.13	50.53	48.85	48.56	46.43	51.40	0.193440528	-0.23
C4_00210W_A	8.52	7.30	6.31	6.24	8.33	5.84	0.563449865	-0.23
C2_10620W_A	737.40	640.04	753.17	698.48	662.85	601.80	0.257892659	-0.23
C4_02450W_A	6.31	2.95	2.41	3.42	3.42	3.81	0.707198016	-0.23
C1_08480C_A	49.89	62.39	68.03	56.46	52.73	57.89	0.420059218	-0.23
C7_04050W_A	50.48	44.92	36.16	27.05	44.65	49.70	0.564527447	-0.23
C3_03530W_A	14.76	2.76	7.11	7.45	7.42	7.49	0.782195072	-0.23
C2_08710W_A	12.93	6.51	10.71	8.62	7.05	12.08	0.637727046	-0.23
CR_04660C_A	169.56	207.51	219.14	208.19	154.79	189.50	0.428034209	-0.23
CR_00390W_A	0.56	0.87	0.89	0.78	0.65	0.72	0.61384795	-0.23
C1_05090W_A	10.47	12.64	6.08	6.57	9.10	11.23	0.654985447	-0.23
C3_00010C_A	16.44	11.07	9.86	9.95	12.59	11.66	0.512320235	-0.23
C5_00110C_A	85.56	76.73	47.20	99.07	2.49	92.82	0.903545921	-0.23
C2_00200W_A	41.39	31.55	31.35	31.97	35.44	27.93	0.394542012	-0.23
C5_00610C_A	7.56	5.70	5.33	6.66	4.23	6.21	0.516259365	-0.23
C5_00560W_A	98.19	97.47	102.04	79.53	88.66	106.11	0.28526406	-0.23
CR_00820C_A	14.31	35.45	30.35	25.38	24.71	24.46	0.672172602	-0.23
C6_04040C_A	177.89	147.87	150.22	126.83	172.50	136.23	0.371486151	-0.23
CR_03660C_A	0.34	0.32	0.58	0.49	0.16	0.50	0.807444908	-0.23
C3_03790W_A	20.97	20.67	24.90	21.05	20.52	19.59	0.322088687	-0.23
CR_05830C_A	5.40	5.97	4.11	4.15	4.92	5.19	0.499646771	-0.23
C2_10460C_A	29.70	30.12	27.52	10.33	35.77	34.26	0.720876414	-0.23
C2_03400C_A	12.23	17.39	17.28	17.33	14.50	11.34	0.55787618	-0.24
C1_03190C_A	247.92	369.02	372.21	406.21	248.20	258.24	0.589841165	-0.24
C4_04640C_A	15.48	11.16	8.53	10.08	11.24	10.80	0.52549973	-0.24
CR_03080C_A	9.82	11.23	11.54	9.32	9.59	10.88	0.427350745	-0.24
CR_00660W_A	0.85	1.94	0.76	0.80	1.16	1.26	0.766096756	-0.24
C1_11270W_A	1522.79	1775.10	1476.66	1419.57	1495.68	1466.33	0.259271414	-0.24
CR_03520C_A	56.09	67.06	64.70	58.63	58.49	55.30	0.294310896	-0.24
C7_02540W_A	3.85	3.62	2.36	3.13	2.73	3.11	0.534925257	-0.24
C1_07850C_A	1.86	1.47	1.87	1.22	1.75	1.76	0.586484287	-0.24
C3_00950C_A	5.19	4.93	4.30	4.52	3.06	5.65	0.557332719	-0.24
C2_10140W_A	0.99	0.59	0.56	0.57	0.54	0.84	0.706392583	-0.24
C6_01250W_A	9.35	8.14	8.07	7.35	6.70	9.17	0.53096468	-0.24
C5_01560C_A	22.12	20.47	24.87	21.15	21.13	19.39	0.314039531	-0.24
CR_07440W_A	25.65	17.67	17.01	17.61	17.57	19.75	0.362877463	-0.24
C5_04050W_A	19.01	12.98	13.37	11.94	13.67	15.69	0.3885326	-0.24
C4_03330W_A	8.57	6.25	5.94	4.29	6.37	8.26	0.584068337	-0.24
C6_00640C_A	5.24	7.04	7.82	7.18	5.24	6.02	0.544557355	-0.24
CR_07190W_A	88.47	80.70	72.75	83.88	76.90	59.21	0.390695902	-0.24
C7_02860C_A	5.36	10.17	10.14	10.73	7.26	5.61	0.667141972	-0.24
C2_10710W_A	83.60	79.13	103.11	89.90	71.23	81.83	0.35686213	-0.24
CR_01500W_A	17.00	14.29	13.92	16.10	13.68	11.30	0.391622808	-0.24
C2_01860C_A	1.86	0.88	1.28	1.73	0.53	1.43	0.783591659	-0.24
CR_07080W_A	31.06	51.72	51.25	50.85	34.37	38.00	0.580131445	-0.24
C1_06900C_A	494.82	372.01	419.67	364.17	409.57	395.37	0.21355526	-0.24
C3_00490W_A	38.25	28.92	28.77	27.41	8.53	52.60	0.763230412	-0.24
CR_06690C_A	44.36	64.69	73.85	66.44	48.46	53.10	0.53947733	-0.24
C2_04970W_A	1.04	0.94	0.93	0.57	0.78	1.28	0.67889888	-0.24
C7_03120W_A	11.68	14.19	13.02	11.84	11.20	12.55	0.328889308	-0.24
C1_01060W_A	563.83	690.74	726.27	598.34	618.93	595.33	0.324569275	-0.24
C1_08260C_A	18.93	48.27	60.05	49.18	36.09	32.00	0.715872549	-0.25
C5_00940C_A	16.29	13.58	14.11	14.00	16.48	9.30	0.536703287	-0.25
C1_07120W_A	61.52	60.62	77.53	67.82	60.49	53.59	0.37311625	-0.25

CR_06180W_A	58.45	81.35	79.23	73.53	60.54	66.64	0.42424164	-0.25
C1_02600W_A	37.75	54.44	62.97	67.10	34.19	40.95	0.625934412	-0.25
C3_02440C_A	6.45	6.46	6.26	5.11	5.85	6.45	0.381776008	-0.25
CR_04590C_A	315.71	320.96	336.68	284.99	305.33	296.53	0.141561013	-0.25
C1_01090C_A	39.90	24.88	30.01	32.22	27.87	25.77	0.46333404	-0.25
C6_03420W_A	1.62	1.33	1.02	0.88	1.21	1.53	0.61456057	-0.25
C1_07290W_A	1.93	2.87	4.13	2.75	3.01	2.33	0.72154429	-0.25
C2_03640W_A	4.40	8.48	8.25	7.43	5.16	6.80	0.626421456	-0.25
C3_03390C_A	11.44	12.32	13.37	12.93	10.18	10.62	0.432372938	-0.25
C2_06480W_A	2.82	4.48	4.00	4.79	1.90	3.68	0.692751602	-0.25
C2_01980C_A	24.27	20.35	20.84	18.28	20.93	20.08	0.17694917	-0.25
CR_01920W_A	46.72	91.14	61.64	56.79	61.39	64.76	0.556758056	-0.25
C6_00070C_A	16.28	13.89	11.14	12.97	12.54	11.80	0.323175762	-0.25
C2_05720C_A	84.00	80.77	70.07	73.28	68.68	70.77	0.150260381	-0.25
C1_13430C_A	13.08	11.97	13.19	9.92	10.10	14.69	0.364824967	-0.25
C1_09310C_A	6.52	7.68	6.82	6.83	6.77	5.44	0.372161935	-0.25
C7_01680C_A	8.93	9.03	5.30	6.38	6.75	7.97	0.539798515	-0.25
C3_06160C_A	2.67	1.39	2.99	5.23	0.60	0.45	0.855833064	-0.25
C3_01080W_A	10.06	7.28	4.81	2.64	10.05	7.20	0.760154673	-0.26
C5_02810W_A	6.93	4.64	5.89	4.03	7.77	3.97	0.67163997	-0.26
C1_12950W_A	2.80	2.18	1.89	1.94	1.80	2.47	0.427668295	-0.26
C7_00510W_A	6.60	1.63	6.54	2.45	6.21	4.49	0.765567238	-0.26
C5_01650C_A	2.72	1.03	0.67	0.97	1.14	1.83	0.742973065	-0.26
C4_00480W_A	6.28	2.69	2.83	3.43	3.60	3.49	0.626421456	-0.26
C1_01340C_A	10.50	10.45	9.04	7.98	8.71	10.43	0.33447544	-0.26
C1_08770W_A	8.23	9.58	11.16	10.17	7.24	8.86	0.450935369	-0.26
C6_00800C_A	15.09	14.35	12.34	11.54	11.77	14.48	0.242158071	-0.26
CR_02410W_A	7.30	7.77	7.86	8.38	5.92	6.43	0.361196229	-0.26
C1_07910C_A	3.71	1.84	1.13	2.84	1.58	1.56	0.740832578	-0.26
C6_00770C_A	8.90	7.41	8.50	7.54	7.00	7.83	0.194775598	-0.26
C2_01750C_A	15.59	11.04	13.18	9.50	13.39	12.92	0.449577425	-0.26
C4_03750C_A	2.78	2.07	1.59	2.29	1.65	1.84	0.595284159	-0.26
C1_13960W_A	9.54	7.69	9.49	7.65	7.76	8.64	0.219550657	-0.26
C1_07060C_A	13.97	10.87	13.66	1.94	14.27	18.56	0.798432646	-0.26
C5_03570W_A	108.53	79.95	82.82	70.96	90.98	81.18	0.306230874	-0.26
C5_02530W_A	5.44	4.56	3.05	3.85	1.48	6.50	0.732362304	-0.26
C4_02940W_A	204.05	159.00	149.06	27.86	173.00	262.78	0.780025516	-0.26
C1_01210W_A	36.14	25.12	43.09	26.43	32.72	34.69	0.448752693	-0.26
C2_07880W_A	8.65	9.39	11.78	8.28	7.73	10.96	0.425351775	-0.26
C6_00500C_A	7.46	9.60	11.02	8.38	7.35	9.68	0.460721683	-0.26
C4_00070C_A	79.92	85.29	72.49	82.00	71.24	60.53	0.317850423	-0.26
C3_02000W_A	8.06	5.93	7.24	7.16	5.88	6.02	0.284156611	-0.26
C7_00690W_A	1.03	1.70	1.71	1.22	1.32	1.45	0.680023077	-0.26
CR_07330W_A	73.90	63.36	24.09	63.26	40.75	40.12	0.679095255	-0.26
C4_03690C_A	8.71	11.80	9.30	10.88	6.50	9.56	0.556950862	-0.26
C2_03160C_A	46.10	66.35	58.36	27.68	52.19	75.61	0.626000782	-0.27
C1_14060W_A	62.32	79.79	68.35	57.68	60.58	72.06	0.302113238	-0.27
C4_00350W_A	20.52	16.03	14.76	12.89	14.34	18.87	0.365778352	-0.27
C2_08030W_A	16.52	9.93	10.92	8.50	14.04	10.72	0.552617108	-0.27
C4_05140C_A	27.19	34.92	25.68	25.55	28.60	24.86	0.34513331	-0.27
C2_04490W_A	86.31	75.26	69.94	63.36	74.73	69.25	0.185387706	-0.27
C5_02450W_A	7.86	7.45	6.39	5.09	5.85	8.58	0.427991386	-0.27
C3_02370C_A	17.30	14.53	16.22	10.83	14.89	17.41	0.358538651	-0.27
C1_11210C_A	43.31	36.65	36.03	33.58	33.48	36.83	0.088852534	-0.27
C1_08710W_A	10.66	6.97	10.00	11.39	5.83	7.43	0.574970467	-0.27
C2_01740C_A	6.67	2.67	4.82	3.93	4.40	4.33	0.67163997	-0.27
C6_01030W_A	3.06	3.83	2.71	2.17	2.93	3.49	0.548653781	-0.27
C1_10860C_A	4.31	2.93	3.94	2.96	3.15	3.87	0.436692845	-0.27
CR_03030C_A	1688.57	1475.25	1615.96	1386.24	1433.09	1460.45	0.044338983	-0.27
C1_05610W_A	15.54	12.72	14.89	13.55	12.06	13.01	0.168108508	-0.27
C4_02970C_A	15.77	16.14	17.20	14.54	13.96	15.60	0.110167234	-0.27
C1_04230W_A	40.10	40.55	45.28	39.85	31.60	41.69	0.247170252	-0.27
C6_03440W_A	0.50	0.56	0.54	0.46	0.40	0.57	0.627912131	-0.27

C7_04100C_A	18.91	25.16	21.69	11.81	24.97	22.24	0.573068975	-0.27	
CR_00040C_A	23.54	21.27	20.33	18.70	20.79	18.63	0.167430605	-0.27	
CR_05360C_A	9.42	5.54	3.67	4.99	3.85	7.74	0.644784207	-0.27	
C5_01300C_A	2.08	3.94	3.84	2.90	3.01	2.91	0.603807499	-0.27	
C1_05510C_A	19.35	8.31	10.17	6.84	12.88	13.62	0.676997771	-0.27	
C5_04190W_A	9.31	6.59	6.30	8.87	6.33	4.46	0.58037552	-0.27	
C7_01350C_A	2.88	0.70	0.53	0.70	1.48	1.37	0.805777887	-0.27	
C2_03690C_A	0.89	9.78	6.55	9.33	0.85	5.66	0.88314809	-0.28	
C7_02120C_A	6.22	8.73	0.89	4.49	5.41	4.26	0.797241915	-0.28	
C6_03760C_A	203.55	195.01	206.74	227.69	152.23	159.92	0.338780633	-0.28	
C4_05980C_A	2.61	3.36	2.41	2.74	2.50	2.25	0.447500628	-0.28	
C6_02950C_A	1.51	2.38	2.24	1.61	2.05	1.79	0.593165621	-0.28	
C2_02740C_A	19.10	32.83	36.89	35.14	25.37	19.09	0.605826077	-0.28	
CR_02100C_A	38.74	37.23	40.12	31.72	35.80	35.85	0.152859497	-0.28	
C2_10210C_A	82.32	88.95	111.73	76.46	89.05	87.30	0.300181262	-0.28	
C3_03320W_A	48.88	64.94	52.62	28.42	28.44	94.12	0.700969957	-0.28	
C7_02770W_A	3.10	9.85	6.84	6.56	6.70	4.56	0.673894716	-0.28	
C6_00540W_A	7.14	6.14	6.94	4.98	6.76	6.20	0.29523343	-0.28	
C5_00380W_A	8.00	3.66	2.13	4.52	3.66	3.90	0.675854824	-0.28	
C4_00310C_A	38.77	38.74	36.12	31.43	35.68	34.06	0.128862545	-0.28	
C1_09970C_A	5.10	5.29	4.60	4.62	4.29	4.43	0.20377455	-0.28	
CR_09840C_A	0.51	0.08	3.06	1.23	1.63	0.34	0.875283067	-0.28	
C4_02660W_A	3.09	2.49	2.38	3.11	2.15	1.83	0.560353914	-0.28	
C1_03170C_A	3.68	1.60	1.75	1.56	1.92	2.71	0.632397069	-0.28	
C1_11940C_A	1.16	1.22	2.28	1.28	1.41	1.45	0.620574837	-0.28	
C5_01290C_A	86.42	82.76	92.05	83.62	73.85	74.97	0.128286964	-0.28	
C1_08940C_A	31.12	30.77	28.67	32.96	26.44	20.88	0.365225217	-0.28	
C4_02680C_A	32.92	10.96	21.52	21.21	24.28	11.44	0.68315221	-0.28	
C1_12290C_A	15.23	14.00	14.64	11.97	13.24	13.75	0.101190317	-0.28	
C6_02760W_A	2.60	2.51	1.29	1.52	2.11	2.03	0.657192765	-0.28	
C5_05090W_A	2.15	1.77	1.66	1.48	2.14	1.26	0.576316367	-0.28	
C1_06580W_A	1024.98	1372.60	1452.52	1114.62	907.95	1434.01	0.414400179	-0.28	
C3_04620C_A	6.19	4.92	6.58	6.65	5.45	3.54	0.59687088	-0.28	
C2_01530C_A	39.08	41.48	40.95	42.61	30.30	35.22	0.256244825	-0.28	
C7_00710W_A	989.13	825.86	1089.96	882.73	870.29	820.57	0.184274408	-0.28	
C2_10790C_A	42.29	1.76	42.81	26.37	20.76	28.89	0.820672324	-0.28	
C1_07440W_A	16.13	14.25	11.97	15.21	12.44	9.71	0.389625274	-0.28	
C2_02620W_A	141.18	82.11	73.80	75.67	76.86	109.30	0.480048543	-0.29	
C4_03300C_A	4.52	4.82	2.54	3.07	4.34	3.01	0.720146917	-0.29	
C2_06540C_A	8.02	9.06	8.51	8.31	5.77	8.71	0.322394245	-0.29	
C1_09980C_A	22.63	22.63	24.00	18.45	20.29	22.67	0.161310515	-0.29	
C7_03290C_A	7.61	9.19	7.55	8.73	6.27	6.62	0.337716968	-0.29	
C1_00470C_A	17.84	20.80	19.60	17.38	17.99	16.20	0.218154639	-0.29	
C6_01200W_A	4.22	5.50	3.64	4.34	5.05	2.34	0.633100596	-0.29	
C5_02670W_A	6.71	5.05	4.69	4.68	4.86	4.98	0.293635105	-0.29	
C6_04440C_A	11.88	8.06	12.06	11.72	9.36	7.01	0.458861418	-0.29	
C6_04140C_A	7.96	6.72	6.28	6.47	6.46	5.54	0.233282783	-0.29	
C2_05440W_A	3.02	2.24	3.23	2.74	2.38	2.36	0.330952463	-0.29	
C1_11230W_A	2.81	1.77	4.86	2.72	2.59	3.00	0.672848344	-0.29	
CR_05570C_A	1.22	0.97	0.60	0.91	0.89	0.66	0.603535711	-0.29	
C1_03250C_A	14.44	13.60	14.19	12.31	12.07	12.91	0.09899972	-0.29	
C4_02550C_A	3.01	4.89	4.69	3.39	4.47	3.26	0.51416326	-0.29	
C4_02960W_A	2.09	1.87	2.64	1.39	1.39	3.10	0.579003852	-0.29	
C1_12340C_A	12.34	13.18	14.03	12.94	9.96	12.06	0.254790755	-0.29	
C5_01810W_A	10.55	13.18	12.56	12.02	9.62	10.49	0.304835066	-0.29	
C4_01430C_A	7.22	1.86	2.23	2.10	3.68	4.01	0.723555571	-0.29	
C3_05950W_A	2.42	2.02	3.74	1.85	3.41	1.92	0.606508734	-0.29	
C4_07240W_A	57.94	58.86	83.10	55.26	62.24	58.98	0.336039634	-0.29	
C6_00760W_A	22.36	20.63	20.35	16.89	18.46	20.43	0.126687081	-0.29	
C3_01710C_A	0.77	0.40	0.20	0.33	0.43	0.44	0.752551935	-0.29	
C3_07570C_A	5.43	7.72	7.05	6.43	5.29	6.13	0.423282513	-0.29	
C1_00550W_A	1.57	5.63	5.68	3.09	3.95	4.49	0.696757093	-0.29	
CR_05240C_A	13.35	9.50	9.21	7.55	10.32	10.23	0.32309142	-0.29	

C7_01550W_A	5.66	6.76	5.92	4.81	5.57	5.83	0.209038348	-0.30
C4_03230C_A	8.66	7.29	7.10	9.81	4.71	5.73	0.496300139	-0.30
C3_07150C_A	199.77	214.95	303.10	214.94	193.51	226.55	0.334486476	-0.30
C4_00660W_A	8.29	7.07	7.10	7.54	6.71	5.48	0.36577669	-0.30
CR_03760W_A	10.86	17.51	16.04	14.80	10.72	13.81	0.458687337	-0.30
CR_06590C_A	7.45	3.48	4.17	3.53	4.43	5.19	0.539002712	-0.30
C1_10100C_A	68.95	139.76	160.37	174.93	44.77	109.54	0.699225814	-0.30
C1_13510C_A	5.88	4.91	5.96	2.96	5.02	6.73	0.514934006	-0.30
C1_05980W_A	8.26	5.66	5.61	4.90	5.86	6.31	0.31836383	-0.30
C4_02430W_A	2.76	1.77	1.36	1.42	1.47	2.27	0.526481529	-0.30
C6_04370W_A	2.47	2.07	1.90	1.91	1.65	2.10	0.306326179	-0.30
C1_04020C_A	44.84	73.08	72.42	64.23	48.07	56.32	0.438421939	-0.30
C1_06790C_A	28.50	25.37	26.31	20.32	24.58	25.29	0.188597122	-0.30
C2_07480W_A	25.52	30.29	25.47	16.25	26.12	29.25	0.412575377	-0.30
C3_05740C_A	4.62	2.35	1.92	2.26	2.89	2.57	0.587428957	-0.30
C1_11510C_A	13.25	17.40	15.12	14.45	11.46	14.39	0.3058719	-0.30
C1_06780W_A	55.61	53.54	62.28	39.00	55.40	55.88	0.26246902	-0.30
C4_00390W_A	127.88	114.88	103.43	112.01	86.06	104.83	0.161310515	-0.30
C3_01530C_A	3.78	4.62	3.74	3.33	3.67	3.66	0.205251358	-0.30
C1_01680C_A	2.98	5.30	5.12	4.61	2.83	4.36	0.608516869	-0.30
C1_00760W_A	59.70	81.85	59.11	63.38	51.73	61.27	0.307487435	-0.31
C2_05870W_A	13.50	12.09	19.77	15.87	9.45	14.49	0.527493727	-0.31
C7_02660C_A	3.78	1.16	1.23	1.35	2.08	1.87	0.719789597	-0.31
C3_00890C_A	3.95	5.16	4.94	3.96	4.07	4.28	0.295497973	-0.31
C1_03040W_A	11.92	10.19	9.62	10.66	8.15	8.91	0.267606345	-0.31
C1_04640W_A	38.23	27.87	30.63	29.26	25.12	29.93	0.142469321	-0.31
CR_07790C_A	99.22	94.07	116.31	102.93	88.01	79.39	0.226147111	-0.31
CR_05990C_A	12.05	10.52	9.89	8.37	10.68	9.19	0.187564025	-0.31
C2_00720C_A	7.98	8.24	6.96	7.38	4.43	8.51	0.426216069	-0.31
C3_05000W_A	109.91	150.09	174.43	127.22	122.57	131.70	0.332459051	-0.31
C5_03760C_A	6.41	5.37	5.21	5.01	4.92	4.85	0.120774269	-0.31
C3_02210C_A	3.62	2.94	2.46	1.97	2.64	3.22	0.406280846	-0.31
C6_03040C_A	3.13	2.70	2.57	2.53	2.99	1.75	0.424778094	-0.31
CR_09720W_A	2.29	3.15	2.58	3.84	1.21	2.02	0.691360154	-0.31
C1_11640C_A	10.90	7.29	13.25	9.41	7.98	9.94	0.412092221	-0.31
C5_05250C_A	11.00	11.80	20.75	16.22	10.94	10.86	0.532841213	-0.31
C1_14050C_A	10.88	12.42	14.13	10.74	12.64	9.18	0.310786494	-0.31
C1_05290W_A	85.67	159.08	112.74	163.22	73.32	76.87	0.592289418	-0.31
C1_07930C_A	3.08	1.86	1.03	1.43	1.11	2.63	0.676462946	-0.31
CR_07860C_A	9.46	9.40	8.21	6.40	9.05	8.03	0.313948856	-0.31
C4_04890C_A	633.22	728.18	757.97	667.82	601.94	575.73	0.164316879	-0.31
C4_01520C_A	322.46	306.51	312.81	289.78	252.07	276.80	0.035464657	-0.31
C2_00750W_A	6.66	6.06	4.30	4.49	2.88	7.46	0.579451395	-0.31
C2_02660W_A	29.21	9.10	24.41	32.26	4.75	17.02	0.747608332	-0.31
CR_03270W_A	26.49	69.53	51.15	46.77	41.13	41.61	0.554112543	-0.32
C1_12110C_A	2.50	1.50	1.23	0.22	2.18	2.00	0.789093411	-0.32
CR_08290W_A	73.33	38.78	31.04	43.77	37.30	41.77	0.477275381	-0.32
C7_01190W_A	9.18	8.50	7.92	7.36	6.56	8.32	0.199626412	-0.32
CR_01480W_A	6.12	4.60	5.00	4.98	4.53	4.12	0.239405677	-0.32
C3_06360C_A	315.30	277.85	290.24	240.70	259.35	265.60	0.021930206	-0.32
C3_01510W_A	13.93	12.56	15.37	11.13	13.01	12.12	0.146285864	-0.32
C2_03970W_A	17.00	9.83	14.22	9.79	14.31	11.21	0.42008083	-0.32
CR_05100W_A	2.15	1.71	1.42	2.18	0.98	1.42	0.524636732	-0.32
C4_00120W_A	22.65	17.82	16.39	11.33	28.10	9.13	0.632423795	-0.32
C5_01740C_A	30.03	31.15	31.08	31.80	23.77	24.46	0.222722133	-0.32
C3_04690C_A	13.76	7.51	8.22	7.16	9.18	8.95	0.418080591	-0.32
C3_02630C_A	36.51	28.56	30.77	27.51	29.18	25.97	0.110239764	-0.32
C1_07510W_A	5.11	2.28	2.88	1.78	3.65	3.42	0.618565804	-0.32
C2_01550W_A	44.45	36.98	36.56	39.45	23.09	39.76	0.332699213	-0.32
C1_04220C_A	32.23	35.12	35.61	28.31	28.35	32.62	0.127939638	-0.32
CR_07720C_A	5.53	5.53	5.52	5.10	5.04	4.18	0.217712321	-0.32
C1_00420W_A	97.85	92.91	102.96	89.18	78.41	86.62	0.049577989	-0.32
C1_06420C_A	8.71	6.33	7.26	7.06	5.60	6.57	0.190234396	-0.32

C2_04480W_A	15.51	27.16	29.10	22.63	17.98	22.04	0.45231575	-0.32
C1_02990C_A	4.47	5.62	5.02	4.73	4.46	3.91	0.315750597	-0.32
CR_01130W_A	11.27	7.29	2.95	0.92	9.27	8.22	0.771385347	-0.32
C2_10030C_A	91.46	179.12	209.01	120.08	153.87	144.78	0.509272978	-0.32
C7_00980W_A	7.51	11.31	11.01	10.43	7.79	7.66	0.396089461	-0.32
C2_02630W_A	5.31	4.15	2.93	1.27	3.89	5.50	0.669211719	-0.32
CR_10270C_A	25.65	22.69	22.52	19.33	19.69	22.06	0.05538606	-0.33
CR_06430W_A	4.13	0.67	2.47	1.86	1.89	2.39	0.72484293	-0.33
C2_05290C_A	8.46	3.21	2.79	2.04	4.79	5.47	0.694314638	-0.33
C1_08690W_A	18.59	19.44	23.67	18.56	15.42	19.34	0.253542867	-0.33
C7_02450W_A	26.01	28.27	26.97	25.29	19.88	25.06	0.128043429	-0.33
C1_06610C_A	98.92	138.64	106.40	116.10	83.78	98.04	0.295810421	-0.33
C1_11130W_A	7.01	2.96	3.40	2.33	5.46	3.50	0.640456429	-0.33
C6_01210W_A	68.38	72.01	84.94	68.28	64.55	61.37	0.170054442	-0.33
CR_08940W_A	11.66	12.11	10.21	4.20	13.39	11.64	0.591182627	-0.33
C4_07140W_A	7.24	6.09	7.47	3.51	6.74	7.52	0.498967305	-0.33
C1_13220C_A	35.09	31.17	23.49	22.55	26.13	28.29	0.207721542	-0.33
C5_04180W_A	4.22	6.10	5.80	4.02	5.36	4.51	0.378643092	-0.33
C1_00920W_A	5.14	4.29	3.86	3.82	3.09	4.50	0.215978208	-0.33
C2_10690W_A	109.58	108.35	96.97	89.48	93.12	87.46	0.043846427	-0.33
C7_01670W_A	4.86	4.70	4.58	4.41	3.98	3.74	0.175185049	-0.33
C1_13940W_A	134.99	164.83	182.17	140.69	135.47	138.96	0.176570351	-0.33
CR_03480W_A	0.84	0.42	0.18	0.31	0.48	0.43	0.726320106	-0.33
C5_04630W_A	15.14	33.63	49.55	31.80	24.75	28.53	0.601316423	-0.33
C3_01990W_A	32.83	36.07	38.33	42.45	23.81	25.72	0.381987462	-0.33
C7_04120W_A	12.70	18.53	13.36	5.50	19.20	13.65	0.628519251	-0.33
C2_03840C_A	36.58	28.09	35.82	26.10	28.86	30.90	0.156177749	-0.33
C3_01690W_A	10.17	13.64	12.57	10.57	9.14	11.57	0.250709924	-0.34
C3_07380W_A	49.07	70.82	59.50	50.44	47.05	56.99	0.250821142	-0.34
C4_03710C_A	6.90	10.15	7.05	9.26	5.04	6.42	0.451644546	-0.34
C1_03370W_A	133.24	81.85	102.74	91.90	81.25	97.23	0.191811404	-0.34
CR_00440C_A	7.30	7.16	6.36	5.91	5.18	6.75	0.099584043	-0.34
C3_07890W_A	34.20	30.39	37.29	33.25	29.56	24.11	0.266981766	-0.34
C3_07470W_A	4.59	4.26	4.02	4.03	3.15	3.85	0.23094529	-0.34
CR_06260W_A	9.41	8.61	11.67	9.02	8.49	7.84	0.227687336	-0.34
C7_01180W_A	14.80	13.00	10.63	9.52	11.42	11.81	0.188597122	-0.34
C2_08080C_A	12.89	15.03	16.56	12.48	11.78	13.75	0.203972612	-0.34
C2_05070W_A	2.98	4.82	3.73	3.26	3.11	3.53	0.357744751	-0.34
C5_04950C_A	2.66	2.86	1.49	1.49	2.04	2.45	0.489482701	-0.34
CR_08340W_A	66.46	51.60	55.59	52.55	49.03	46.05	0.081038236	-0.34
C7_01230C_A	43.76	34.09	36.48	30.99	34.20	32.07	0.068284403	-0.34
C6_04520W_A	105.37	140.24	104.35	113.61	94.77	91.26	0.238317954	-0.34
C5_03990W_A	7.72	7.55	7.59	5.76	6.18	7.56	0.133484449	-0.34
C1_13050W_A	396.88	357.95	430.43	349.29	303.71	358.90	0.059277447	-0.34
C5_01240W_A	20.68	18.06	17.22	15.30	17.47	14.77	0.108614843	-0.34
CR_02580W_A	7.63	6.04	3.83	4.64	5.16	5.00	0.415150676	-0.34
C5_04520W_A	11.57	13.31	11.89	11.39	10.35	9.65	0.166816725	-0.34
C1_09130W_A	10.25	8.77	7.74	7.00	8.19	7.55	0.134921033	-0.34
C3_02770C_A	29.52	26.95	25.43	24.74	19.70	25.39	0.088104103	-0.34
C4_04120C_A	6.61	5.14	4.82	3.41	5.55	5.08	0.332459051	-0.34
C7_04230W_A	416.27	238.24	352.70	259.21	313.24	278.60	0.262227464	-0.34
C2_08000C_A	6.64	7.54	9.04	1.41	11.72	6.59	0.711145919	-0.34
C6_01630W_A	42.97	35.73	34.88	33.59	31.55	31.35	0.04923674	-0.34
C3_07370W_A	34.13	29.98	28.36	25.55	27.03	25.97	0.072204875	-0.34
C4_03620C_A	5.57	4.51	4.51	4.41	3.92	4.06	0.195674262	-0.34
C7_03710C_A	5.85	2.72	3.00	2.96	4.49	2.21	0.54355225	-0.34
C1_12870C_A	3.27	3.43	3.14	3.46	1.97	2.94	0.462068069	-0.35
C4_01310W_A	1.13	0.87	0.57	0.73	0.99	0.44	0.62185304	-0.35
C5_03490C_A	20.33	21.92	31.37	24.31	18.97	19.49	0.309656971	-0.35
C5_02780W_A	4.71	3.01	2.93	3.05	3.44	2.50	0.358643236	-0.35
C5_01530C_A	23.38	18.63	16.74	16.40	16.42	16.98	0.108295622	-0.35
CR_06900C_A	5.52	3.40	5.85	4.18	4.09	4.21	0.324762636	-0.35
CR_08370W_A	40.77	45.92	43.04	35.90	33.64	41.03	0.077995262	-0.35

CR_08580C_A	1.78	2.35	2.86	1.10	2.54	2.30	0.592534388	-0.35
C1_04280C_A	1.76	0.88	1.35	0.86	1.34	1.14	0.556758056	-0.35
C2_08060W_A	39.86	43.02	41.36	35.08	39.69	30.46	0.221399826	-0.35
C4_04810C_A	0.98	1.99	1.60	1.48	1.19	1.19	0.583210979	-0.35
C7_03810W_A	21.70	29.57	13.84	17.65	16.25	21.61	0.412493973	-0.35
CR_08830W_A	78.71	176.31	150.84	141.66	101.67	104.19	0.486507391	-0.35
C4_00570C_A	61.81	48.46	49.30	45.89	41.93	47.06	0.045156713	-0.35
C4_06040W_A	4.76	3.63	4.11	3.46	2.87	4.22	0.278332245	-0.35
C2_09620W_A	8.46	12.11	15.33	9.56	10.27	10.76	0.348012544	-0.35
C2_03980C_A	4.65	5.77	5.42	4.59	4.44	4.39	0.1906825	-0.35
C1_05720W_A	659.68	657.15	788.50	679.94	579.96	520.70	0.155664126	-0.35
CR_10370W_A	181.69	99.76	181.36	113.91	156.20	117.96	0.36194376	-0.35
C2_01440C_A	4.96	3.24	5.56	2.13	3.18	6.28	0.644034297	-0.35
C1_04120C_A	2.67	4.48	3.69	3.96	3.03	2.21	0.507914297	-0.35
C2_03990W_A	16.13	18.95	18.65	17.82	13.26	14.45	0.195233351	-0.35
C2_00320W_A	43.26	46.55	48.96	39.85	45.39	31.80	0.221461537	-0.36
C7_01420W_A	14.01	9.41	8.54	11.70	0.98	14.49	0.748913226	-0.36
CR_03830C_A	3.52	3.16	2.63	2.57	2.55	2.74	0.155374742	-0.36
CR_04100C_A	1186.46	1273.39	1421.66	1004.60	1145.23	1133.86	0.081517374	-0.36
C5_01450W_A	15.46	21.59	19.51	15.12	15.17	17.73	0.208563499	-0.36
C7_03870W_A	30.81	69.41	33.98	43.34	24.49	47.31	0.544436215	-0.36
CR_09320C_A	47.67	16.54	35.38	14.61	35.21	33.18	0.585455759	-0.36
CR_09990W_A	10.51	9.59	9.70	8.27	8.21	8.63	0.010511422	-0.36
C2_04540C_A	2.82	3.70	3.51	2.89	1.99	3.56	0.424533358	-0.36
C3_01160W_A	26.65	22.48	19.32	17.57	18.89	21.19	0.09716984	-0.36
CR_01140C_A	9.07	8.99	6.32	1.07	9.44	10.08	0.720904972	-0.36
C7_00840C_A	1.91	0.97	0.84	0.25	1.38	1.47	0.705692236	-0.36
C3_02990C_A	15.66	12.97	11.45	11.96	10.41	11.42	0.125602808	-0.36
C1_11750W_A	5.34	5.22	5.88	5.06	4.88	3.89	0.198715297	-0.36
C3_03050C_A	14.13	12.48	12.85	10.68	11.77	10.73	0.042660632	-0.36
CR_08650C_A	51.03	25.26	61.68	30.53	41.45	43.78	0.448877558	-0.36
C2_06260W_A	4.40	4.28	3.70	4.18	2.87	3.36	0.405761222	-0.36
C2_07440C_A	9.49	11.74	10.22	11.40	10.20	4.77	0.487424201	-0.36
C1_09530W_A	9.34	4.31	5.32	5.74	4.64	5.41	0.435213825	-0.36
C4_01470W_A	3.54	2.14	2.01	1.49	3.19	1.71	0.550605517	-0.36
C1_00910W_A	8.22	6.20	6.14	6.08	5.78	5.41	0.233388472	-0.36
C5_00420C_A	7.83	8.00	9.79	7.10	7.47	6.95	0.323438998	-0.36
CR_00400C_A	25.87	19.22	30.07	22.73	19.55	20.76	0.191672172	-0.36
C6_02460C_A	9.04	6.31	12.32	7.55	5.96	9.80	0.418794988	-0.36
C6_01100W_A	50.23	32.28	30.46	33.49	29.51	31.19	0.209870637	-0.36
C2_10770W_A	25.94	1.35	31.23	16.39	10.57	21.79	0.769923659	-0.36
C6_03450C_A	6.09	7.11	5.72	4.89	4.51	6.56	0.207674201	-0.36
C6_04290W_A	30.99	19.40	16.74	18.95	17.21	19.77	0.269911233	-0.36
C3_04090W_A	466.74	481.41	468.60	410.05	366.43	414.79	0.015721787	-0.36
C3_04990W_A	33.14	21.55	29.93	21.17	23.38	26.21	0.163949246	-0.36
C1_12700W_A	3.22	4.31	3.84	2.84	3.42	3.30	0.249560437	-0.36
C6_01790C_A	10.91	14.56	9.50	10.70	8.82	9.92	0.254419419	-0.37
C3_00120W_A	10.41	9.38	7.26	6.00	11.81	4.67	0.537862878	-0.37
CR_08680C_A	14.05	1.93	11.93	1.52	20.41	0.54	0.875283067	-0.37
C5_01820W_A	18.61	19.44	22.86	20.03	15.32	15.77	0.167061471	-0.37
C4_00410W_A	17.84	26.04	29.81	16.16	24.88	20.86	0.381721295	-0.37
C2_07210C_A	106.42	68.89	124.21	121.59	52.10	76.58	0.464083649	-0.37
C5_03070W_A	317.62	494.58	595.56	475.73	330.03	383.17	0.370954913	-0.37
C1_10720C_A	9.02	9.16	8.27	7.71	7.75	6.67	0.104760264	-0.37
C3_01470W_A	26.54	23.32	28.36	21.66	22.25	21.40	0.084987884	-0.37
C1_08210C_A	6.24	11.74	10.97	7.39	9.20	7.82	0.382627354	-0.37
C3_06740W_A	2.68	2.31	2.40	1.87	2.21	2.08	0.187235705	-0.37
CR_07480W_A	152.25	179.13	176.04	141.81	137.09	146.98	0.05931053	-0.37
C7_00750W_A	96.08	63.44	64.47	21.10	87.82	77.15	0.599770716	-0.37
C2_03260W_A	40.10	36.80	38.02	29.17	26.76	40.37	0.142376959	-0.37
C3_05830W_A	16.56	18.87	19.84	19.14	9.85	17.50	0.333983925	-0.37
CR_06610W_A	1.11	0.84	0.65	0.80	0.83	0.53	0.55199061	-0.37
C1_08840W_A	3.90	3.78	3.73	2.67	3.72	3.11	0.268845258	-0.37

C2_09180W_A	2.75	2.53	1.49	1.88	1.53	2.20	0.458305122	-0.37
C5_01110W_A	16.88	14.36	12.92	12.74	11.42	12.66	0.085440793	-0.37
C2_06240W_A	2.96	3.21	4.02	1.98	1.94	4.48	0.602372176	-0.37
C2_00820W_A	5.94	11.96	6.88	10.96	4.67	5.23	0.558976743	-0.37
CR_07110C_A	1.43	1.08	0.77	0.79	0.76	1.16	0.461807226	-0.37
C4_06880C_A	47.34	60.84	70.21	54.13	44.12	51.12	0.235308679	-0.37
CR_01540W_A	8.32	4.50	4.79	4.95	3.93	5.72	0.337899779	-0.37
CR_03390C_A	12.38	15.03	13.52	12.52	11.09	10.54	0.130212358	-0.37
C2_04530W_A	4.80	2.06	2.07	2.58	1.96	2.85	0.551778589	-0.38
C2_04930C_A	23.97	20.32	21.83	17.61	17.15	20.30	0.018437313	-0.38
C7_00860W_A	41.85	37.21	46.24	36.66	33.18	34.46	0.060954838	-0.38
C2_00160C_A	49.82	47.56	49.88	38.05	38.20	46.52	0.037620302	-0.38
C2_06920C_A	6.41	30.60	28.22	18.68	9.86	27.28	0.668057638	-0.38
C4_02280W_A	25.64	26.75	37.90	26.42	26.15	22.55	0.233602751	-0.38
C2_00780W_A	23.78	23.41	28.80	30.86	6.78	26.09	0.592288302	-0.38
C3_05940C_A	5.01	2.50	7.51	4.44	4.71	3.20	0.570996853	-0.38
CR_03040C_A	14.40	20.07	21.04	15.86	14.09	16.49	0.212848645	-0.38
C3_04970C_A	16.22	10.95	17.42	10.83	12.33	13.82	0.193093102	-0.38
C3_04840C_A	5.63	6.79	5.64	5.84	5.73	3.41	0.366919269	-0.38
C5_05160C_A	16.41	25.99	38.28	26.11	18.03	23.53	0.453785668	-0.38
CR_01370C_A	17.84	8.45	11.95	10.27	8.42	12.94	0.368163212	-0.38
C1_14590C_A	68.37	52.35	55.75	92.78	25.02	27.66	0.608906763	-0.38
C2_04380C_A	10.37	14.42	7.34	10.37	2.25	14.49	0.657288045	-0.38
C3_04500C_A	1716.92	1573.46	1670.50	1362.41	1320.43	1435.54	0.001275333	-0.38
C2_05590C_A	6.87	9.17	7.74	8.23	5.22	6.35	0.309160202	-0.38
C1_01370C_A	706.12	1029.49	1139.76	976.16	745.90	672.76	0.307097137	-0.38
C3_01500C_A	26.81	19.09	21.91	16.93	16.87	22.32	0.113184899	-0.38
CR_10430C_A	5.83	4.37	10.63	6.67	4.73	5.79	0.503072422	-0.38
CR_01230C_A	4.94	3.22	2.98	2.71	3.20	3.28	0.246974348	-0.38
C7_00580C_A	22.82	24.53	17.74	15.74	18.15	20.03	0.165593374	-0.38
C5_05440C_A	167.06	127.09	106.11	94.95	127.82	106.33	0.188097865	-0.38
C2_05810W_A	13.25	14.48	11.10	10.14	10.75	11.26	0.094448459	-0.38
C2_07060W_A	42.24	39.12	38.41	33.89	33.61	31.48	0.022977153	-0.38
C1_14430C_A	48.43	9.33	63.42	37.47	51.35	9.11	0.722718122	-0.38
C1_06380C_A	18.20	22.06	17.80	17.08	11.80	19.26	0.287073743	-0.39
CR_03490W_A	7.85	8.53	6.76	6.47	5.76	6.95	0.105470543	-0.39
C3_02410C_A	11.88	7.65	9.51	8.44	5.99	9.50	0.24700984	-0.39
C1_02300W_A	7.75	5.33	9.75	4.84	7.46	6.51	0.438146363	-0.39
CR_01320C_A	8.21	8.02	8.09	6.36	6.99	6.70	0.025959831	-0.39
C1_03050W_A	8.36	8.23	6.63	6.53	6.31	6.32	0.063402968	-0.39
CR_08400C_A	66.95	58.16	72.58	52.59	60.38	49.72	0.101444155	-0.39
C5_04410C_A	28.96	24.74	33.21	25.15	23.76	22.71	0.088574174	-0.39
C6_00530C_A	4.01	5.89	6.78	5.83	4.79	3.11	0.554768156	-0.39
C2_08770C_A	24.54	22.46	26.27	15.38	22.49	22.57	0.207365618	-0.39
C3_02600C_A	28.62	22.11	22.76	16.78	22.90	20.69	0.128853623	-0.39
C4_04330C_A	118.58	76.10	149.36	101.64	80.83	100.82	0.28388768	-0.39
CR_06000W_A	14.71	11.09	10.15	8.86	10.07	10.61	0.103747332	-0.39
C6_01970C_A	652.96	736.01	1372.99	782.96	583.60	931.58	0.407366686	-0.39
C7_03920C_A	390.75	609.71	378.18	308.88	325.70	514.30	0.338491523	-0.39
C5_02920W_A	2.32	5.47	3.92	4.00	2.18	3.57	0.519097311	-0.39
C1_13560W_A	4.87	3.81	3.54	2.47	3.77	3.78	0.268368773	-0.39
CR_04800W_A	1.59	0.69	1.00	0.77	0.97	0.93	0.471463456	-0.39
C3_02330C_A	1.14	0.82	0.55	0.72	0.40	0.94	0.62860851	-0.39
CR_09420C_A	13.38	18.23	10.21	10.42	12.73	11.35	0.279402656	-0.39
C5_01720C_A	1.91	1.21	1.20	0.83	1.36	1.36	0.394439108	-0.39
C2_02010C_A	2.45	4.45	3.31	3.05	2.38	3.05	0.40340839	-0.39
C2_04330C_A	11.75	8.03	9.08	7.34	7.75	8.60	0.134424978	-0.39
C4_03400W_A	32.25	34.59	60.77	45.98	28.40	30.79	0.426989764	-0.39
CR_05130C_A	5.36	4.06	6.01	3.60	5.46	3.55	0.354226684	-0.39
C3_00610W_A	10.55	10.59	12.00	8.71	9.94	8.60	0.134729778	-0.39
C7_00250C_A	53.03	57.76	58.65	45.97	82.72	8.60	0.677651439	-0.39
C5_03810C_A	1.74	1.13	0.66	0.49	0.92	1.47	0.667965964	-0.39
C1_00180W_A	1228.80	995.10	1076.32	964.44	837.55	900.89	0.012500414	-0.40

C6_03580W_A	4.60	4.40	4.01	3.52	3.04	4.14	0.113069021	-0.40
C7_00830C_A	42.64	24.17	25.16	0.01	39.59	35.28	0.888758564	-0.40
C2_07720C_A	16.36	14.13	13.90	12.61	12.82	10.88	0.05374646	-0.40
C3_03210W_A	15.68	9.88	5.37	9.53	8.66	6.87	0.468473059	-0.40
C3_02880W_A	9.14	5.92	9.56	6.99	8.04	5.06	0.395301718	-0.40
C1_03090W_A	405.71	485.55	602.36	482.17	358.37	390.25	0.197798514	-0.40
C3_07860C_A	25.06	16.42	17.53	18.05	15.71	14.30	0.153377436	-0.40
C6_03660C_A	30.36	35.96	46.08	29.42	26.79	36.61	0.203972612	-0.40
C3_07400W_A	5.43	8.96	8.18	7.68	5.17	5.77	0.36194376	-0.40
C2_07860W_A	12.24	9.81	13.29	8.50	6.46	14.13	0.347344173	-0.40
C3_04710W_A	3.16	2.55	2.10	1.72	1.60	3.10	0.453752359	-0.40
C2_10830W_A	2.88	2.52	8.97	6.06	2.24	3.45	0.697915242	-0.40
C3_05240C_A	368.66	185.70	355.18	184.28	337.22	214.64	0.399555964	-0.40
C3_04880W_A	324.91	293.76	202.40	187.40	272.67	208.38	0.233602751	-0.40
C1_05350W_A	20.31	17.98	13.75	12.08	13.82	16.66	0.178431358	-0.40
C1_09990W_A	9.10	5.23	4.85	3.77	5.37	6.42	0.354519292	-0.40
C3_04810C_A	1390.53	1446.48	860.76	830.54	1145.39	1041.35	0.24237306	-0.40
C3_04140C_A	244.23	220.97	268.69	265.85	82.47	255.49	0.480048543	-0.40
C2_01610C_A	1732.88	1606.56	1934.04	1549.69	1548.00	1196.96	0.101024838	-0.40
C2_04600C_A	325.41	248.55	325.54	238.13	281.93	210.10	0.121235397	-0.40
C1_06520C_A	5.37	3.99	4.32	3.53	3.79	3.81	0.038901343	-0.40
C7_00680W_A	2.41	2.11	1.96	1.18	1.64	2.36	0.472259804	-0.41
C6_01960W_A	2.84	3.45	4.45	3.19	2.53	3.05	0.277502241	-0.41
CR_02460W_A	26.32	23.02	28.92	21.94	23.23	18.48	0.089705557	-0.41
C3_00140W_A	26.53	7.62	20.70	15.48	27.71	0.00	0.896161594	-0.41
C6_01160W_A	4.14	4.28	3.83	3.80	2.74	3.47	0.288481554	-0.41
C1_01650W_A	36.62	34.49	41.57	32.01	27.38	32.54	0.037944667	-0.41
C3_04750W_A	7.89	6.42	11.52	7.65	6.63	6.77	0.258244027	-0.41
C7_01660C_A	3.82	4.11	4.74	3.65	3.10	3.59	0.114530826	-0.41
CR_00430C_A	23.21	19.62	36.61	18.01	24.20	22.47	0.345956761	-0.41
C3_00030C_A	10.33	7.00	6.42	6.23	6.69	6.29	0.144042348	-0.41
C7_04130C_A	80.19	87.40	86.02	33.48	81.89	92.12	0.420773123	-0.41
C7_04260W_A	97.56	46.53	43.11	46.82	51.03	52.45	0.337439676	-0.41
C1_07250C_A	17.80	13.08	18.94	7.36	17.84	15.19	0.399578359	-0.41
C2_02720W_A	13.74	14.62	14.42	17.00	11.07	6.55	0.403516306	-0.41
CR_07760W_A	24.99	23.01	26.70	23.73	26.79	9.68	0.476970313	-0.41
C2_02800W_A	28.51	25.42	63.06	33.45	44.56	16.30	0.563265957	-0.41
C1_02330C_A	34.00	11.80	28.48	12.11	21.61	26.10	0.507931821	-0.41
C4_04850C_A	6.80	8.29	8.00	5.97	6.86	5.94	0.117221393	-0.41
CR_07730W_A	6.70	6.76	6.81	7.73	4.74	3.94	0.314273198	-0.41
C3_00050C_A	28.11	23.07	25.47	21.38	20.99	19.67	0.077023753	-0.41
C7_03580C_A	15.83	15.19	15.30	11.35	13.14	13.04	0.016859828	-0.41
C2_09140C_A	16.76	14.15	17.01	13.51	13.30	11.94	0.03230769	-0.41
C3_05530W_A	14.44	19.15	16.48	9.94	14.72	16.16	0.224856263	-0.41
C3_00530C_A	4.19	4.92	5.70	4.45	3.24	4.36	0.214248416	-0.41
C4_05820W_A	9.04	2.41	5.71	3.31	5.29	5.13	0.601316423	-0.42
C7_00600C_A	20.90	21.92	14.91	14.11	15.69	16.98	0.128421301	-0.42
C2_09220W_A	473.44	720.07	648.83	544.66	452.52	506.51	0.16957725	-0.42
C1_07070C_A	7.62	5.50	7.12	4.54	6.13	5.67	0.14209881	-0.42
C4_01220C_A	0.90	1.21	1.06	0.74	0.84	0.97	0.334131448	-0.42
C2_07330W_A	8.40	13.63	13.78	13.01	10.29	5.74	0.422211715	-0.42
CR_04650W_A	25.75	17.11	18.06	14.62	22.42	11.68	0.374207058	-0.42
CR_03980W_A	16.24	18.03	15.78	13.89	13.35	13.18	0.061061728	-0.42
C3_01070C_A	1.94	1.05	0.60	0.65	0.94	1.30	0.54355225	-0.42
C1_04910C_A	29.74	26.31	27.85	23.24	23.11	21.33	0.016482341	-0.42
CR_01650W_A	8.25	11.88	15.26	10.36	11.98	6.28	0.42711838	-0.42
C4_02040W_A	13.36	16.35	18.20	7.92	15.13	15.83	0.327133109	-0.42
C1_06560W_A	9.40	10.38	10.51	6.79	7.87	9.85	0.12010373	-0.42
C7_03570W_A	19.58	17.95	20.14	13.96	13.63	19.05	0.071192646	-0.42
C2_10010C_A	36.98	40.42	55.07	22.97	38.30	46.30	0.321024318	-0.42
C1_05670W_A	71.17	80.07	86.03	70.76	60.61	60.54	0.058711704	-0.42
C1_12150C_A	7.80	6.97	7.06	4.26	6.92	6.32	0.244902138	-0.42
CR_06910W_A	5.07	4.55	4.07	4.28	3.21	3.54	0.076822314	-0.42

C6_01570C_A	3.50	2.06	1.76	1.88	1.98	1.99	0.292894471	-0.42
C3_00470W_A	26.87	25.81	5.16	22.18	3.95	20.68	0.681052586	-0.42
C4_03680C_A	4.68	2.83	2.54	2.47	2.59	2.99	0.285887191	-0.42
C1_05330C_A	2.82	4.36	4.16	2.85	2.71	3.62	0.290865189	-0.42
C2_03940C_A	4.05	4.40	4.17	3.56	2.93	3.68	0.076021725	-0.42
C2_08090W_A	6.73	8.44	7.83	6.19	5.90	6.36	0.191052967	-0.42
C3_05850W_A	15.71	9.55	9.53	6.77	7.38	13.82	0.352253857	-0.43
C3_01490W_A	58.98	30.84	44.87	28.68	38.48	40.38	0.235308679	-0.43
CR_08360C_A	724.46	590.10	754.86	564.63	601.48	491.05	0.056689024	-0.43
C4_06440C_A	2.78	2.68	2.93	1.95	2.47	2.32	0.195390416	-0.43
C6_03490C_A	1.06	1.57	1.26	1.44	0.78	0.90	0.479703216	-0.43
C1_05860W_A	10.58	14.36	4.56	7.79	7.78	8.19	0.456878639	-0.43
C4_04160W_A	48.07	25.76	28.13	30.51	25.49	25.03	0.21917248	-0.43
C4_03990C_A	8.50	10.44	8.98	8.02	7.31	7.13	0.108905717	-0.43
C3_05120C_A	44.11	40.90	34.92	34.85	29.28	32.12	0.041300839	-0.43
C2_03670W_A	2.28	9.42	7.58	7.40	4.11	4.20	0.588170536	-0.43
C7_04110W_A	14.22	20.41	13.29	6.76	16.10	15.85	0.421415878	-0.43
C1_04840C_A	6.42	9.82	7.79	7.00	6.13	6.11	0.314097651	-0.43
C4_00030C_A	32.29	31.41	31.38	25.19	25.58	25.42	0.005119753	-0.43
C3_02220W_A	52.96	57.24	59.37	49.99	41.01	45.24	0.030206159	-0.43
C1_03630W_A	3.45	3.07	3.15	2.33	3.06	2.32	0.106930389	-0.43
CR_06290C_A	47.48	32.67	35.42	30.82	30.74	30.56	0.049121106	-0.43
C6_03100W_A	8.82	6.54	6.55	6.18	5.62	5.74	0.07472003	-0.43
C4_06260W_A	9.48	10.21	7.13	6.57	8.01	6.88	0.186896287	-0.43
C3_06480C_A	127.05	161.03	153.33	134.08	102.71	118.25	0.096278823	-0.43
C2_10860C_A	88.48	89.72	89.61	74.41	59.87	80.66	0.026600092	-0.43
C3_07610W_A	12.45	18.30	14.29	11.59	10.87	13.84	0.140315988	-0.43
C2_01820C_A	4.47	1.88	2.07	2.35	1.89	2.43	0.402485681	-0.43
C6_04600W_A	16.73	18.30	16.19	10.64	16.04	14.26	0.128853623	-0.43
C4_06070C_A	7.54	6.75	5.61	7.06	4.23	4.55	0.252894538	-0.44
C1_11450C_A	74.51	55.29	86.32	48.90	72.15	50.61	0.203455751	-0.44
C1_11500C_A	7.25	9.55	7.30	10.25	8.48	0.28	0.740830134	-0.44
C2_08070C_A	12.76	13.38	15.33	11.31	10.30	11.49	0.055096347	-0.44
C3_06610W_A	31.86	25.27	35.34	27.44	25.54	20.59	0.119948403	-0.44
C5_02700W_A	17.42	13.54	14.31	12.32	12.38	11.28	0.023713531	-0.44
C1_13440C_A	0.54	0.70	0.38	0.54	0.58	0.15	0.635356797	-0.44
CR_08010W_A	36.65	38.14	43.85	31.81	31.50	31.29	0.073807877	-0.44
C7_00660W_A	24.47	31.25	10.21	26.22	3.67	23.29	0.646975601	-0.44
C6_04420W_A	699.01	763.01	751.71	563.71	621.32	583.46	0.013345175	-0.44
C2_03960W_A	262.59	406.33	423.99	398.09	244.18	234.01	0.308646927	-0.44
C6_01390W_A	12.82	7.83	10.28	7.02	7.38	10.19	0.178431358	-0.44
C1_00770C_A	3.60	1.86	2.03	1.64	3.05	1.19	0.540350859	-0.44
C2_03150C_A	7.23	7.15	8.49	3.77	5.70	8.85	0.312036194	-0.44
C2_08930W_A	14.88	1.46	3.97	7.47	1.82	6.52	0.70500737	-0.44
C2_02700C_A	24.31	16.25	20.16	18.98	11.49	17.76	0.187823351	-0.44
C1_11920W_A	29.20	29.16	41.54	20.66	27.78	31.34	0.178431358	-0.44
C6_01150W_A	4.65	8.07	3.73	4.57	4.15	4.45	0.342641768	-0.44
C2_05540C_A	16.54	16.83	13.21	12.27	12.78	11.97	0.054367655	-0.44
CR_00620C_A	106.07	129.16	165.84	130.73	91.97	97.61	0.192357881	-0.44
C2_04500W_A	16.45	16.49	8.23	9.86	11.51	11.29	0.259069812	-0.44
C1_03550C_A	59.46	49.87	72.05	51.61	47.47	44.92	0.074920736	-0.44
C3_06240C_A	3.83	3.31	4.46	4.25	3.46	1.38	0.595215264	-0.44
C1_09770W_A	32.60	42.46	61.59	53.14	31.78	23.77	0.419124063	-0.44
C7_01600W_A	7.71	6.06	7.46	5.69	5.66	5.51	0.065539274	-0.44
C7_03170W_A	5.20	7.33	5.92	4.64	4.14	5.98	0.170165801	-0.44
C1_02390W_A	7.93	3.77	4.82	3.42	3.49	6.14	0.346977241	-0.45
C3_00660W_A	1.09	0.98	1.05	0.86	0.66	0.96	0.169945263	-0.45
C3_01220W_A	5.12	2.83	2.96	1.66	3.97	2.93	0.445361518	-0.45
C1_00500C_A	50.94	97.88	97.84	75.42	56.05	66.33	0.295521236	-0.45
C1_05030C_A	17.36	23.85	23.14	18.85	15.58	16.82	0.140315988	-0.45
C4_04010W_A	15.85	11.45	9.33	8.26	11.34	9.20	0.170026954	-0.45
C2_04250W_A	11.69	10.16	8.28	7.86	7.53	8.46	0.04742202	-0.45
CR_01150C_A	6.54	7.19	2.14	0.84	6.04	5.70	0.657410559	-0.45

C1_12400C_A	46.89	37.69	44.49	36.89	29.04	36.17	0.042785939	-0.45
CR_02240C_A	6.18	5.63	7.68	4.98	4.80	5.65	0.070100307	-0.45
C5_05130C_A	7.01	27.28	27.36	20.45	12.57	16.64	0.540749679	-0.45
CR_07780W_A	17.16	21.39	15.93	15.16	11.89	16.26	0.101699606	-0.45
C6_02090C_A	3.01	2.26	2.06	1.98	1.68	2.13	0.11551425	-0.45
C2_00620C_A	1.81	2.87	3.71	2.73	1.51	2.48	0.443595011	-0.45
C5_02340C_A	17.86	14.43	11.21	9.10	13.01	12.11	0.140760129	-0.45
C4_05810W_A	127.44	106.09	114.79	122.62	70.34	81.57	0.157570822	-0.45
C2_06770W_A	2.90	2.90	2.10	1.76	1.99	2.43	0.233700456	-0.45
CR_08170C_A	14.65	13.83	13.99	13.62	3.59	16.63	0.50737014	-0.45
C2_04770W_A	5.72	2.55	1.99	3.09	2.58	2.33	0.464490645	-0.45
C1_06100C_A	18.60	17.12	19.34	16.76	11.52	15.24	0.058961384	-0.45
C4_04790W_A	0.24	0.45	0.63	0.36	0.39	0.30	0.547403992	-0.45
C5_03540C_A	1440.44	1330.75	1676.18	1226.13	1224.62	1047.55	0.037800802	-0.46
CR_05010W_A	5.26	2.84	3.09	2.94	2.41	3.41	0.24413131	-0.46
C4_06000W_A	7.29	7.98	7.40	6.31	5.15	6.46	0.05538606	-0.46
C1_06450C_A	846.97	764.22	916.04	694.60	627.05	667.74	0.005308008	-0.46
C4_02480C_A	9.09	5.69	5.05	4.42	5.28	5.86	0.343059481	-0.46
C6_00140C_A	15.07	21.39	19.85	16.03	15.57	12.78	0.181205109	-0.46
CR_10000C_A	34.82	20.79	16.61	19.98	15.57	20.84	0.268864995	-0.46
C2_01880C_A	5.73	2.96	2.71	3.27	2.44	3.18	0.347063232	-0.46
C6_04210C_A	14.10	8.64	8.06	7.86	7.02	9.22	0.159299941	-0.46
C2_04940C_A	16.58	20.07	22.74	15.89	16.96	13.88	0.103105175	-0.46
C3_02970C_A	40.67	38.45	40.30	30.85	30.80	32.17	0.001456496	-0.46
C3_05370C_A	709.29	703.20	812.37	680.80	535.42	531.47	0.043473981	-0.46
C2_09920W_A	3.12	1.98	1.72	1.32	2.73	1.28	0.536679563	-0.46
C1_02050C_A	26.19	27.50	25.51	20.62	19.76	21.88	0.002756781	-0.46
C2_04650C_A	11.13	2.43	3.29	3.44	3.89	5.63	0.570171429	-0.46
C3_05460W_A	3.40	3.32	3.00	2.73	2.06	2.83	0.083410728	-0.46
C3_04330C_A	190.99	257.42	241.78	202.16	168.79	172.76	0.082000781	-0.46
C7_00940W_A	20.81	23.09	22.89	17.18	18.17	17.07	0.014603411	-0.46
C4_06760W_A	79.93	10.20	55.71	29.98	46.07	35.76	0.581482155	-0.46
CR_09890C_A	24.93	20.39	26.11	29.94	21.50	3.78	0.598172611	-0.46
C6_03010W_A	2.89	3.19	3.15	2.68	2.33	2.23	0.033745141	-0.46
CR_03430W_A	11.57	7.56	11.76	9.76	6.39	7.90	0.26214262	-0.46
C1_08520C_A	10.43	7.33	10.84	7.74	8.36	6.21	0.214695376	-0.47
CR_01000C_A	5.01	2.56	4.25	3.30	2.41	3.49	0.219334301	-0.47
CR_03910C_A	20.17	18.60	10.33	10.44	17.44	10.24	0.318253367	-0.47
C5_04790C_A	4.93	5.16	5.11	3.60	3.13	5.16	0.224476189	-0.47
C3_07390C_A	12.22	11.34	11.80	9.39	7.38	10.88	0.07749022	-0.47
C7_00720W_A	28.09	16.74	16.27	13.56	15.23	18.63	0.156213754	-0.47
C6_04480C_A	203.00	83.94	253.17	206.07	132.63	77.47	0.476560035	-0.47
C2_00120C_A	39.89	32.46	30.18	27.16	24.56	28.07	0.00902833	-0.47
CR_10440W_A	1.52	0.98	2.24	1.08	1.06	1.55	0.35552759	-0.47
C1_14280C_A	251.62	225.09	265.26	197.86	194.42	184.90	0.003905859	-0.47
C2_03570C_A	8.67	10.08	8.57	7.42	7.04	6.82	0.082000781	-0.47
C2_00500W_A	11.52	14.55	18.02	11.34	10.94	12.21	0.10966024	-0.47
C1_00690W_A	2.78	2.56	2.06	1.51	1.81	2.44	0.152958615	-0.47
C2_05570C_A	7.80	9.76	10.55	7.14	7.04	7.71	0.093535806	-0.47
C2_04760W_A	30.75	20.41	20.75	24.87	16.29	14.24	0.202502163	-0.47
C2_01390W_A	38.19	46.99	41.00	12.34	43.56	42.71	0.440900619	-0.48
CR_07100W_A	18.00	21.75	20.59	15.47	15.12	16.44	0.020183988	-0.48
C1_02620C_A	125.46	129.88	174.42	135.45	101.18	97.53	0.119023643	-0.48
C3_05960W_A	5.79	3.19	8.04	3.87	5.48	3.76	0.342698327	-0.48
C1_10400C_A	127.57	122.92	110.50	92.77	98.22	88.71	0.005112676	-0.48
C5_01930W_A	7.03	5.14	7.62	5.59	4.29	5.43	0.106494068	-0.48
C1_09210C_A	12.82	10.50	11.18	10.64	6.85	9.26	0.074387291	-0.48
C1_08830C_A	1.79	1.81	1.21	1.67	1.29	0.82	0.536830985	-0.48
CR_03590C_A	4.96	5.01	5.93	3.72	4.22	4.35	0.124643364	-0.48
C2_09760W_A	50.60	27.79	29.47	25.58	24.67	32.79	0.171286357	-0.48
C1_03930W_A	1.73	1.22	1.14	0.63	0.84	1.68	0.441766185	-0.48
C5_00170W_A	54.81	84.56	76.50	57.24	56.59	54.34	0.110246082	-0.48
C1_13160W_A	60.37	83.19	62.81	52.77	45.23	62.95	0.090465811	-0.48

C3_00350W_A	20.55	29.79	25.88	20.27	16.52	22.59	0.125716418	-0.48
C2_09490W_A	6.80	7.26	7.80	5.10	6.95	4.82	0.142969898	-0.48
C5_01170W_A	9.17	9.27	7.05	8.11	6.29	5.22	0.189158071	-0.48
C3_05150W_A	115.35	85.06	77.19	67.37	77.49	68.16	0.044057964	-0.48
C1_06570W_A	6.16	8.05	8.79	6.07	3.43	8.42	0.322983017	-0.48
C4_03670W_A	4.07	8.27	3.58	6.33	3.25	2.80	0.448758779	-0.48
C2_03810C_A	889.71	999.33	1205.92	909.72	791.95	690.93	0.076014223	-0.48
C1_12130C_A	4.46	3.52	3.69	2.75	2.94	3.31	0.033286549	-0.49
C1_08420W_A	15.45	11.70	11.66	9.81	9.78	10.27	0.011400923	-0.49
C1_05070C_A	2.39	2.09	2.29	1.61	1.76	1.84	0.047822989	-0.49
C4_01860C_A	8.71	7.99	6.79	5.07	5.71	7.34	0.077260369	-0.49
C1_07880C_A	12.15	14.49	7.96	7.83	8.28	10.70	0.177684504	-0.49
C3_01570W_A	3.21	3.23	4.07	2.51	2.35	3.24	0.125008019	-0.49
CR_05900W_A	4.79	5.14	4.52	3.31	4.10	3.69	0.066424439	-0.49
CR_04120C_A	1.38	5.11	4.85	2.78	3.01	3.09	0.475616917	-0.49
C6_03800C_A	18.10	15.60	17.54	14.41	11.32	13.78	0.011400923	-0.49
C3_02100W_A	16.02	14.50	14.66	11.84	11.79	11.13	0.004488142	-0.49
C3_00310C_A	32.16	26.03	27.38	17.95	15.60	32.52	0.225708226	-0.49
CR_01200W_A	1.78	0.93	2.13	1.39	1.27	1.04	0.40361642	-0.49
C3_07490W_A	49.03	55.66	52.38	47.65	34.11	39.35	0.041163656	-0.49
C3_02690C_A	4.36	4.59	3.18	2.97	2.55	3.83	0.146173236	-0.49
CR_01820W_A	11.14	6.02	10.14	8.74	6.22	5.85	0.198916985	-0.49
CR_10450C_A	26.99	26.55	56.71	25.13	30.40	29.45	0.288218402	-0.49
CR_09480W_A	6.60	6.00	5.48	3.95	4.97	4.95	0.050615958	-0.49
C5_03400C_A	38.48	47.77	47.02	34.82	30.92	36.92	0.047000231	-0.49
C2_05360C_A	7.92	6.98	8.06	5.30	5.38	6.92	0.034636807	-0.49
C2_10850C_A	37.88	27.43	23.57	22.71	20.28	24.98	0.057999093	-0.49
C7_03610C_A	3.49	3.29	2.63	2.69	2.46	2.04	0.082533095	-0.49
C5_05020C_A	19.65	15.83	15.64	11.76	14.35	12.97	0.022236486	-0.49
C5_03010W_A	7.83	2.52	2.60	3.14	2.89	3.72	0.425295788	-0.50
C4_03550W_A	6.75	7.66	5.93	5.15	5.07	5.37	0.025013693	-0.50
C5_00430W_A	20.72	23.11	26.79	21.00	17.04	16.02	0.082950119	-0.50
C1_10390C_A	704.77	1149.34	1304.95	1069.35	723.21	638.35	0.255137137	-0.50
C7_03670W_A	152.48	193.12	177.75	159.34	157.33	82.57	0.224900778	-0.50
CR_01670W_A	4.53	2.64	3.01	2.35	2.79	2.63	0.168047036	-0.50
C1_01460W_A	9.69	10.76	13.77	9.36	6.58	10.40	0.121159174	-0.50
CR_03470W_A	16.93	18.61	16.78	12.59	14.36	13.05	0.013861792	-0.50
C3_03430C_A	9.77	11.53	14.26	8.83	8.23	10.18	0.121865966	-0.50
C3_03740W_A	9.05	12.09	15.30	9.71	8.55	9.53	0.222770765	-0.50
C4_00180W_A	12.83	14.79	14.27	11.18	10.51	10.30	0.017912169	-0.50
C1_13010W_A	2.14	1.46	1.91	1.28	1.30	1.60	0.085017892	-0.50
C1_05820C_A	12.13	12.36	12.29	8.03	10.91	9.04	0.049106282	-0.50
C4_06020C_A	13.38	13.05	11.72	10.10	9.19	9.80	0.001776082	-0.50
C2_07940C_A	8.45	10.73	10.32	7.01	7.16	8.39	0.050800911	-0.50
C5_00230C_A	10.71	7.41	11.25	7.65	8.07	6.54	0.11066959	-0.51
CR_01380W_A	3.14	2.94	3.32	3.05	2.02	2.08	0.108950746	-0.51
C3_03670W_A	8.36	9.43	10.44	7.47	5.90	8.19	0.052176576	-0.51
C3_06710W_A	16.82	20.36	19.84	15.53	14.44	13.43	0.027503313	-0.51
C3_06090C_A	17.01	4.64	11.67	1.04	10.89	13.11	0.645279519	-0.51
C6_02660C_A	6.42	6.60	5.87	5.17	4.15	5.01	0.024752878	-0.51
C7_02960C_A	9.55	8.94	17.72	9.31	8.95	9.25	0.225163325	-0.51
C3_07340W_A	184.99	239.56	189.22	150.56	158.54	157.97	0.025136613	-0.51
C3_05180W_A	10.17	7.71	6.70	6.14	5.57	6.84	0.115136317	-0.51
CR_05970C_A	17.11	18.94	20.20	13.61	14.77	14.29	0.013765512	-0.51
C3_04780C_A	49.38	76.55	42.87	40.37	42.13	46.23	0.144333011	-0.51
C7_01870W_A	9.95	8.46	8.94	8.00	6.79	5.83	0.041275504	-0.51
C3_03060W_A	9.95	8.18	9.14	6.05	7.23	7.25	0.07346183	-0.51
C1_12070C_A	2.97	1.94	3.19	2.17	1.92	2.03	0.138193072	-0.51
C1_08470W_A	82.95	66.22	80.04	48.93	56.21	68.15	0.021620683	-0.51
CR_10020C_A	29.54	19.66	24.90	24.61	15.91	15.14	0.142730155	-0.51
C6_03950C_A	27.79	19.56	20.46	14.44	20.12	16.38	0.066323368	-0.51
C4_04130W_A	15.14	14.13	17.77	10.16	12.07	13.33	0.047804846	-0.52
C2_07520C_A	19.42	12.66	17.09	8.87	14.74	13.29	0.129034091	-0.52

C3_05800W_A	3.22	2.65	1.94	2.33	1.22	2.36	0.283701436	-0.52
C1_04350C_A	2.70	2.67	1.13	1.27	1.84	1.79	0.33447544	-0.52
C5_04160W_A	20.80	23.67	26.90	17.11	14.58	22.18	0.095888915	-0.52
CR_09410W_A	7.71	8.45	5.37	4.46	5.62	6.17	0.108905717	-0.52
C4_06420W_A	8.17	6.05	5.57	4.06	4.04	6.79	0.13475826	-0.52
C3_05280C_A	15.89	8.48	9.90	7.89	8.47	9.29	0.126022007	-0.52
CR_08610W_A	34.51	70.70	98.19	15.63	68.20	71.67	0.528675818	-0.52
C2_01910W_A	10.02	4.81	5.61	4.88	4.28	6.07	0.241019105	-0.52
CR_07510W_A	9.88	5.83	13.39	5.54	7.65	8.62	0.315735244	-0.52
C1_09160W_A	8.47	7.93	8.27	6.19	6.15	6.20	0.00972027	-0.52
C1_08460C_A	92.52	81.30	87.12	63.39	68.10	64.44	0.000195124	-0.52
C4_00620C_A	6.64	9.72	18.70	10.00	6.83	9.71	0.38130273	-0.52
C2_04040C_A	4.99	4.63	4.85	3.43	3.60	3.83	0.000779903	-0.52
C2_10110W_A	56.49	44.53	61.62	31.44	63.79	25.73	0.311836946	-0.52
C5_00460C_A	1.93	1.49	1.59	1.36	1.21	1.19	0.116478831	-0.52
C7_04200C_A	2.31	2.33	2.45	0.58	2.19	2.57	0.446008267	-0.53
C4_01140C_A	3.40	2.30	2.63	1.77	1.90	2.49	0.28388768	-0.53
C6_01900C_A	31.11	39.02	52.60	34.83	28.29	29.39	0.121063912	-0.53
CR_09910W_A	4.05	3.27	5.37	4.23	2.37	2.92	0.215978208	-0.53
C1_08910C_A	1.92	0.52	0.99	0.26	1.00	1.26	0.575922439	-0.53
C5_04420W_A	25.99	35.31	39.07	28.28	22.75	24.63	0.081945364	-0.53
C4_06280C_A	3.07	6.49	8.60	4.82	3.59	5.37	0.362414337	-0.53
C4_04880W_A	3.62	5.06	4.17	4.03	2.34	3.32	0.210141834	-0.53
C1_11030W_A	82.60	68.80	94.30	64.08	63.24	56.37	0.019838974	-0.53
C2_00090W_A	19.48	21.66	17.72	13.77	18.30	11.94	0.104877955	-0.53
C4_03410W_A	10.35	7.76	8.01	6.78	4.76	8.05	0.109292298	-0.53
C2_06780C_A	7.38	4.61	4.84	4.48	3.70	4.37	0.085017892	-0.53
C2_09060C_A	147.94	200.83	184.17	154.64	124.22	121.68	0.053356274	-0.53
C5_04530W_A	7.25	6.92	5.98	4.69	5.99	4.33	0.100521193	-0.53
C3_02910W_A	7.84	10.14	11.73	7.10	10.85	4.14	0.345075276	-0.53
C6_02570C_A	15.97	17.33	19.92	14.50	12.02	13.30	0.025531023	-0.53
C7_01830W_A	7.55	4.16	6.41	5.99	4.32	3.17	0.278300618	-0.53
C3_03070W_A	2.89	2.23	3.90	2.72	1.70	2.31	0.218011248	-0.53
C1_08490W_A	59.76	62.43	62.46	47.52	44.64	45.86	0.000552971	-0.53
C7_00960W_A	948.23	981.40	1203.73	851.71	793.45	694.13	0.023919876	-0.53
CR_09290W_A	7.05	0.54	5.84	1.47	3.84	4.54	0.638295276	-0.53
C4_00380W_A	12.34	16.95	15.14	8.42	12.07	12.82	0.108762369	-0.53
C1_05530C_A	4.90	2.17	2.78	1.43	2.78	3.04	0.343361175	-0.53
C6_00870C_A	440.84	450.78	449.63	331.43	330.82	339.62	6.74E-05	-0.53
C4_06950W_A	13.14	12.42	11.37	12.26	7.12	8.17	0.120774269	-0.53
CR_02180W_A	7.11	9.05	9.71	7.69	6.44	5.21	0.117122502	-0.54
C1_01580W_A	16.54	26.79	28.61	23.88	14.76	15.41	0.22771888	-0.54
C7_03840W_A	15.38	18.02	11.95	10.27	9.68	13.99	0.093248753	-0.54
C1_00970W_A	13.02	9.55	11.08	9.61	8.33	7.00	0.097766889	-0.54
C2_05710C_A	530.19	669.32	801.12	521.57	513.03	461.03	0.057229056	-0.54
C1_10380C_A	18.16	14.90	16.71	12.73	12.26	11.94	0.001073737	-0.54
C1_10760W_A	14.60	10.79	12.90	7.54	10.43	10.42	0.040851343	-0.54
C2_09750W_A	160.35	149.78	141.39	118.18	96.10	121.97	0.001461791	-0.54
C1_01290C_A	11.00	8.52	7.02	6.26	6.61	6.80	0.033278441	-0.54
C2_02690W_A	54.22	18.32	38.47	43.13	11.97	26.64	0.441048966	-0.54
C5_05140W_A	7.33	10.92	11.31	8.83	6.65	6.61	0.14544041	-0.54
C3_06980W_A	2.59	2.39	1.55	1.47	1.21	2.15	0.27878466	-0.54
C6_01700W_A	637.74	584.06	730.19	443.61	546.90	457.59	0.014290818	-0.54
CR_03750C_A	7.91	8.93	6.14	5.07	6.44	5.52	0.083043369	-0.54
C3_07420W_A	19.05	21.27	22.49	19.15	12.81	14.81	0.056423154	-0.54
C1_12390C_A	757.16	588.87	669.83	463.18	541.86	485.84	0.002977349	-0.54
C1_01930W_A	320.99	323.37	382.89	251.72	269.04	242.26	0.005715762	-0.54
C1_02460W_A	753.49	861.09	1059.96	714.50	632.29	644.39	0.02855127	-0.54
CR_08130W_A	21.89	14.98	16.70	13.45	12.69	13.43	0.014984193	-0.54
C1_03020C_A	1603.61	1279.00	1424.49	1075.48	1092.34	1013.59	0.000589622	-0.54
C2_01260W_A	4.09	3.20	5.69	3.17	2.83	3.60	0.125674946	-0.54
C5_01200W_A	45.12	43.27	44.02	28.95	35.63	33.49	0.005499125	-0.54
CR_06140W_A	55.93	44.41	46.68	35.61	37.40	35.56	0.00077722	-0.54

C1_08000W_A	2.07	0.97	1.59	0.97	1.20	1.24	0.227100589	-0.54
C1_06470W_A	674.00	788.02	976.93	655.16	558.74	600.55	0.037947451	-0.54
C1_06400C_A	25.57	18.12	14.94	9.16	16.89	17.14	0.178431358	-0.54
C1_05900W_A	11.90	17.97	7.37	8.10	9.36	10.28	0.227556592	-0.54
C2_05560W_A	17.17	22.69	25.62	22.40	14.28	11.93	0.194281364	-0.54
C2_09930W_A	7.73	7.41	6.83	3.93	7.57	4.69	0.161726244	-0.54
C6_00390W_A	25.66	23.11	26.56	21.88	20.44	13.17	0.089871493	-0.55
CR_05160C_A	2.38	1.80	1.96	1.50	1.55	1.45	0.135351139	-0.55
CR_05720W_A	156.46	186.65	181.64	121.00	105.85	164.14	0.039338094	-0.55
C2_04400W_A	101.65	95.10	92.78	76.69	62.84	74.59	0.000564771	-0.55
C2_01250W_A	67.54	78.75	101.63	57.90	58.22	67.99	0.048259557	-0.55
C2_01950C_A	2.79	0.53	0.92	0.58	1.52	0.97	0.553339673	-0.55
C3_01100W_A	10.27	9.47	10.66	6.64	8.26	7.43	0.03366835	-0.55
C1_02310C_A	7.22	10.19	7.72	4.48	5.69	8.40	0.243557744	-0.55
C4_06850C_A	35.12	34.69	35.91	25.50	27.48	24.98	0.001190602	-0.55
C1_05100W_A	4.96	5.09	4.68	3.67	3.53	3.67	0.05515923	-0.55
C3_00580W_A	12.60	13.73	11.57	8.20	9.89	9.89	0.007359439	-0.55
C6_02860W_A	5.11	4.24	5.45	3.18	2.50	5.27	0.151688854	-0.55
C2_03950W_A	9.90	4.85	8.21	6.43	5.25	5.08	0.17359445	-0.55
C2_02200W_A	9.99	7.39	7.16	5.13	5.58	7.31	0.047234115	-0.55
C5_01520C_A	31.50	31.36	33.62	23.68	23.46	23.82	0.000313573	-0.56
C3_00340W_A	12.76	13.69	11.00	6.98	9.03	11.47	0.118920471	-0.56
C1_09840C_A	25.63	27.37	18.57	16.58	16.64	19.39	0.033358671	-0.56
C6_02310W_A	13.56	8.44	11.35	9.05	9.84	5.31	0.172341056	-0.56
CR_10010C_A	25.20	11.16	10.97	11.77	10.77	11.75	0.206273368	-0.56
CR_00560W_A	40.92	35.98	21.73	12.21	29.77	30.28	0.285495201	-0.56
C1_05260C_A	21.40	23.08	22.93	17.60	16.58	15.28	0.003734328	-0.56
C2_08620W_A	19.85	14.25	12.55	10.50	10.33	13.29	0.040573515	-0.56
CR_01550C_A	2.39	1.47	0.42	0.56	0.87	1.69	0.564827412	-0.56
C1_04960C_A	15.93	8.56	8.62	7.30	6.47	10.34	0.153998026	-0.56
CR_10470C_A	5.46	4.73	9.47	3.46	5.38	5.59	0.229902228	-0.56
C3_02310W_A	32.20	25.11	37.95	21.69	23.52	24.47	0.021645052	-0.56
C2_05700W_A	1076.20	988.78	1013.02	695.83	766.11	791.42	1.82E-05	-0.56
C3_03690W_A	3.29	2.03	2.18	1.38	1.63	2.44	0.258842043	-0.56
CR_08320W_A	8.84	9.32	9.26	7.09	6.26	6.68	0.01413527	-0.56
C1_00530C_A	15.24	23.29	23.77	15.18	15.61	15.10	0.071192646	-0.56
C1_12530C_A	4.42	3.15	3.56	2.97	2.19	2.94	0.070960042	-0.56
CR_08980C_A	12.61	11.68	10.92	2.82	11.21	11.77	0.389671193	-0.56
C3_01620W_A	9.98	9.30	7.98	7.49	4.39	8.03	0.138909996	-0.56
C3_00790W_A	4.49	4.27	3.84	2.28	3.93	2.96	0.092686597	-0.56
C7_04090C_A	13.08	19.71	13.26	10.14	10.72	13.03	0.082512658	-0.56
C4_02580W_A	6.11	22.75	20.72	11.99	7.38	17.60	0.463101296	-0.57
C2_08720W_A	16.66	9.92	11.19	10.38	6.40	10.69	0.12288963	-0.57
C3_06150W_A	9.40	5.58	5.26	4.19	5.26	5.20	0.089540992	-0.57
C1_07630W_A	5.89	14.93	18.90	5.65	10.47	13.22	0.418341416	-0.57
C2_09030W_A	20.06	23.23	21.97	12.34	18.73	16.53	0.061027846	-0.57
C2_04310W_A	88.85	94.79	100.04	69.25	71.24	66.58	0.001303812	-0.57
C1_04700C_A	8.25	8.69	8.56	5.26	6.04	7.32	0.010453121	-0.57
C7_01940C_A	44.02	30.07	25.36	29.46	21.07	21.39	0.08162365	-0.57
C7_02430C_A	2.87	4.25	1.68	2.41	1.39	2.66	0.342420316	-0.57
C6_00690W_A	2.72	2.33	2.94	2.30	1.67	1.82	0.086661872	-0.57
C1_08650C_A	10.24	12.54	12.33	11.53	5.29	8.88	0.178431358	-0.57
C2_00940W_A	292.75	307.71	333.35	224.17	226.64	230.42	0.000544405	-0.57
C7_01850C_A	164.84	75.18	74.74	74.70	73.70	77.56	0.157941815	-0.57
C2_02950W_A	63.65	97.38	82.35	61.29	77.17	38.61	0.184244182	-0.57
C4_06510C_A	18.26	16.44	15.63	10.34	11.81	14.45	0.010950246	-0.57
C7_01950W_A	9.60	3.99	4.65	5.74	3.68	3.62	0.292930921	-0.57
C7_00590W_A	19.82	17.57	10.80	8.64	12.97	13.31	0.138264326	-0.57
C5_00350C_A	28.83	12.59	4.66	10.61	11.03	11.18	0.440086386	-0.57
C5_03170C_A	8.10	8.54	6.38	6.43	5.24	5.05	0.030478328	-0.57
C1_07480C_A	0.63	0.56	0.12	0.25	0.44	0.25	0.570296578	-0.57
C2_09630C_A	1.83	1.17	2.15	0.86	1.15	1.63	0.417399969	-0.57
C2_08860W_A	60.20	39.34	36.74	26.21	32.15	40.02	0.062844863	-0.58

C5_05050W_A	8791.09	11729.75	10637.45	8023.56	7571.60	7082.52	0.016249905	-0.58
C1_09240C_A	1.37	3.03	1.61	2.02	0.78	1.60	0.403286376	-0.58
C3_05720C_A	4.95	3.01	2.83	2.54	3.02	2.16	0.11814457	-0.58
C3_02960C_A	4.81	4.94	4.98	4.28	2.77	3.64	0.027505016	-0.58
C2_00270C_A	45.72	42.67	43.67	30.70	39.29	25.10	0.04322188	-0.58
C4_04170C_A	6.98	4.23	3.68	2.99	4.41	3.25	0.14144624	-0.58
C3_02110W_A	1110.37	1206.02	1402.79	1069.10	851.82	771.67	0.027074284	-0.58
CR_01690C_A	75.93	75.86	62.07	48.76	47.99	58.19	0.001785133	-0.58
C4_01650C_A	9.92	7.93	9.29	6.08	6.96	6.56	0.005215943	-0.58
C7_02380C_A	448.45	926.29	734.23	496.36	503.13	541.75	0.134950832	-0.58
C3_03040W_A	54.91	58.16	62.29	46.98	40.05	39.76	0.003204808	-0.58
C3_05900W_A	8.23	9.92	5.24	4.73	6.44	5.66	0.151203592	-0.58
C3_05250C_A	11.13	5.85	8.52	4.44	7.94	5.84	0.16170464	-0.58
C2_04160W_A	7.65	14.04	14.77	9.54	8.18	8.82	0.166816725	-0.58
CR_04890W_A	2.42	1.81	0.77	1.02	1.08	1.48	0.390061185	-0.58
C1_04530C_A	13.99	20.16	16.62	12.45	12.40	11.95	0.033769301	-0.58
C2_09360W_A	1.08	1.18	0.90	0.62	0.82	0.83	0.12699066	-0.58
C3_04490W_A	19.58	15.40	15.94	11.65	11.84	13.09	0.000870705	-0.58
C4_00160C_A	85.22	89.65	100.00	64.21	64.85	69.35	0.000751017	-0.59
CR_06500C_A	8.07	3.41	1.95	1.86	3.22	4.43	0.478238467	-0.59
C5_04300C_A	44.78	83.49	84.39	49.95	53.11	51.52	0.125915425	-0.59
C2_05890C_A	178.74	179.00	167.17	127.16	130.99	119.55	0.000182135	-0.59
C2_08540C_A	4.15	1.95	2.34	1.66	1.81	2.58	0.24897453	-0.59
C3_01350C_A	6.05	5.27	6.50	4.51	3.73	4.51	0.039387466	-0.59
C1_13790C_A	15.40	16.85	14.14	13.01	6.24	14.27	0.189982743	-0.59
C1_02230W_A	5.96	5.94	5.10	2.85	4.13	5.23	0.076822314	-0.59
C3_05710W_A	996.97	1501.59	1070.46	849.43	949.68	777.56	0.05226015	-0.59
C3_03310C_A	9.86	13.93	9.04	4.40	6.37	13.10	0.308646927	-0.59
C1_08820C_A	7.68	10.54	12.31	8.99	7.74	5.26	0.180322948	-0.59
C1_04820C_A	3.95	5.65	14.21	10.65	1.97	4.57	0.561909303	-0.59
C2_10720C_A	27.72	31.76	32.88	21.20	20.71	24.61	0.004251569	-0.59
C5_05150C_A	6.25	7.84	7.99	6.19	5.05	4.61	0.055622484	-0.59
C1_14560C_A	64.38	63.69	64.34	47.27	46.72	43.90	0.000141213	-0.59
C2_03560C_A	8.01	7.98	8.42	7.70	3.72	6.10	0.11930636	-0.59
CR_07000C_A	4.26	4.10	3.21	3.37	2.60	2.29	0.040025508	-0.60
C3_00620C_A	10.35	24.88	31.49	20.65	12.03	15.58	0.33389939	-0.60
C1_01570C_A	23.35	23.13	32.23	19.54	17.67	19.08	0.02998006	-0.60
C7_03060C_A	6.68	7.94	6.76	5.87	3.85	5.59	0.038807766	-0.60
C7_03260C_A	17.63	18.77	24.75	15.00	14.06	14.65	0.027572786	-0.60
CR_07220C_A	2.56	2.16	2.49	2.04	1.64	1.46	0.036616358	-0.60
C7_02970W_A	7.12	7.32	11.46	7.67	5.16	5.67	0.112738471	-0.60
C3_05700W_A	11.25	8.75	8.52	6.73	6.08	7.47	0.003543089	-0.60
C1_02260C_A	40.30	25.70	33.60	23.12	29.65	17.52	0.086884989	-0.60
C5_04060C_A	3.25	3.48	2.98	2.37	2.45	2.11	0.004894374	-0.60
CR_04490C_A	23.69	23.28	24.35	19.85	12.66	18.46	0.023824861	-0.60
C4_00590C_A	72.48	53.42	60.44	41.74	45.53	44.98	0.000652225	-0.60
CR_10130W_A	5.13	7.07	12.74	8.16	4.09	5.64	0.312119474	-0.60
C2_00650W_A	6.97	7.10	8.46	6.53	4.99	4.52	0.032270701	-0.60
C2_02160W_A	12.20	8.59	7.78	4.49	7.23	8.55	0.123651572	-0.60
C6_01670W_A	57.56	54.25	68.40	41.64	47.65	38.85	0.007543228	-0.60
C1_05750C_A	6.43	5.30	5.62	4.15	4.06	4.13	0.000134091	-0.60
C1_03460C_A	5.53	3.94	3.43	3.62	2.46	3.07	0.060641107	-0.60
C6_04510C_A	15.66	19.97	9.38	10.30	11.73	10.02	0.131914225	-0.60
CR_10760C_A	19.45	14.42	14.48	13.68	11.77	8.83	0.081627408	-0.60
C1_13950C_A	7.11	9.11	7.89	6.70	5.49	4.97	0.092782031	-0.60
CR_00190W_A	6.92	10.24	5.64	1.04	7.45	7.84	0.48948122	-0.60
C1_03950C_A	3.78	1.72	2.62	1.54	1.12	2.98	0.399479643	-0.60
C1_08030W_A	474.10	343.39	344.51	281.30	257.51	283.22	0.001802191	-0.60
CR_01040C_A	15.93	10.56	9.45	9.24	0.21	16.42	0.662253102	-0.61
C5_01470C_A	2.89	2.62	2.42	1.79	1.13	2.70	0.184350059	-0.61
C2_09460C_A	11.37	9.53	7.94	6.83	6.83	6.76	0.0045121	-0.61
C1_13620W_A	73.70	60.80	67.92	46.62	48.06	48.78	7.43E-06	-0.61
C2_09530W_A	25.66	18.44	17.43	13.39	15.85	14.17	0.011806457	-0.61

C6_02280W_A	25.89	22.73	13.85	12.64	17.70	13.69	0.097761348	-0.61
C6_00460C_A	8.90	11.08	11.04	7.66	8.17	6.19	0.032570302	-0.61
C6_02470W_A	8.57	5.52	6.48	4.00	4.39	6.16	0.051729613	-0.61
CR_01590C_A	46.13	49.25	46.20	33.93	31.36	35.26	9.65E-05	-0.61
C6_01070C_A	6.32	3.72	4.44	3.04	4.08	3.08	0.11954924	-0.61
C1_02740C_A	9.85	9.62	10.07	7.07	7.21	6.67	0.004766811	-0.61
C6_02140W_A	11.80	9.38	8.79	5.64	6.67	8.89	0.024021853	-0.61
C1_05800C_A	4.01	4.56	6.82	3.65	3.30	3.94	0.14772381	-0.61
C5_02350C_A	4.09	2.39	2.90	1.97	2.58	2.04	0.150617252	-0.61
CR_00590W_A	5.79	1.93	1.41	1.94	1.94	2.45	0.35479897	-0.61
C6_02990W_A	8.12	8.49	9.33	5.36	5.76	7.24	0.009922678	-0.61
C5_01400W_A	1.09	1.55	0.92	1.07	0.58	0.87	0.244889658	-0.61
C4_02180C_A	1.41	1.33	1.56	0.88	1.09	1.06	0.055818958	-0.61
C6_02560W_A	45.87	76.87	72.35	50.98	45.67	41.98	0.071831263	-0.61
C2_02680W_A	8.34	3.49	5.67	6.27	2.77	3.13	0.296622658	-0.61
CR_04010C_A	28.60	21.84	20.00	16.13	17.13	16.21	0.005308008	-0.61
C6_03170C_A	4.46	3.56	3.00	2.14	2.74	2.84	0.046286317	-0.61
C5_00130C_A	60.54	63.39	35.31	54.93	1.76	56.93	0.715643183	-0.61
C7_01590C_A	4.27	3.84	3.98	2.43	2.71	3.39	0.026139638	-0.62
C2_02020W_A	15.26	16.13	16.67	13.16	9.56	11.25	0.005810906	-0.62
C4_01360W_A	64.45	86.97	107.38	69.31	57.08	56.89	0.060970505	-0.62
C1_12470W_A	1.60	1.73	0.35	0.59	0.75	1.22	0.526111216	-0.62
C6_02170C_A	2.19	3.36	3.91	2.39	2.61	1.67	0.210581775	-0.62
C3_06190C_A	1.34	1.89	1.70	1.44	1.03	1.00	0.092196159	-0.62
C3_00390W_A	5.27	3.85	4.89	2.36	3.48	3.94	0.099584043	-0.62
CR_08960C_A	2.41	2.72	2.70	0.81	2.56	2.12	0.279969997	-0.62
C7_01800C_A	1652.03	937.43	927.14	854.21	808.41	784.93	0.042154288	-0.62
C7_00620W_A	32.72	36.67	26.24	6.53	30.92	30.02	0.370417611	-0.62
C3_05200W_A	676.92	631.07	775.58	523.62	484.57	455.85	0.001190429	-0.62
C1_09400C_A	7.37	7.61	10.84	6.07	4.58	7.56	0.081805598	-0.62
C5_01230C_A	38.91	46.17	45.39	33.36	28.91	29.72	0.003806028	-0.62
C5_02660C_A	5.84	5.56	5.15	2.18	1.82	7.80	0.401070169	-0.62
C3_06270C_A	10.88	8.32	19.93	11.83	6.75	8.93	0.233619124	-0.62
C2_00640W_A	4.78	7.37	7.59	5.10	4.52	4.30	0.064257524	-0.62
C5_01980C_A	27.09	29.86	39.78	24.72	18.40	25.07	0.039947136	-0.62
C5_00190C_A	88.19	222.81	173.85	119.54	117.04	107.47	0.176461828	-0.62
C1_09920W_A	6.00	6.25	6.41	3.45	4.66	4.96	0.011645946	-0.63
C1_06820W_A	2.69	13.57	21.18	7.78	8.57	10.36	0.465496893	-0.63
C6_03270C_A	46.27	50.38	66.05	35.99	39.47	38.52	0.013616347	-0.63
C5_00750C_A	31.87	39.98	46.63	30.08	25.81	27.29	0.017775845	-0.63
CR_10390W_A	2.90	1.14	3.53	1.28	1.70	2.29	0.365326862	-0.63
C3_05680W_A	12.30	11.99	13.80	9.90	7.35	9.35	0.012951971	-0.63
C3_01670W_A	77.14	81.81	77.17	54.12	49.72	61.44	0.000207272	-0.63
C2_03910C_A	5.38	4.69	4.80	3.70	2.90	3.77	0.003087273	-0.63
C2_02580W_A	7.59	11.31	12.38	9.65	6.78	5.46	0.134928647	-0.63
C1_11160C_A	13.91	19.28	31.21	15.54	14.46	15.16	0.177867786	-0.63
CR_06170W_A	22.68	19.85	22.93	13.40	14.01	18.25	0.004211733	-0.63
C2_04580W_A	29.38	13.15	15.88	6.87	11.57	22.13	0.296289404	-0.63
C6_03230W_A	21.54	8.77	10.60	5.82	10.57	11.74	0.231669796	-0.63
C1_04570C_A	2.21	1.49	2.02	1.30	1.21	1.48	0.089437714	-0.63
C1_09050W_A	8.01	4.60	4.88	3.56	4.09	4.46	0.0537671	-0.63
CR_07800W_A	2.50	4.19	5.09	3.81	1.97	2.47	0.241934504	-0.63
CR_06010W_A	19.45	14.62	15.19	12.31	11.20	10.70	0.002749843	-0.63
C1_03260W_A	8.49	8.38	7.38	6.36	5.17	5.36	0.003209371	-0.63
C2_07170C_A	10.44	5.38	5.93	4.69	4.76	5.57	0.069219191	-0.63
CR_10610C_A	21.37	20.28	20.80	6.08	17.41	20.06	0.257168319	-0.63
C6_03690W_A	2.21	2.93	2.79	1.92	1.95	1.63	0.030385527	-0.64
C1_06890C_A	1375.44	804.75	905.89	717.69	729.93	672.88	0.016649395	-0.64
C3_04830C_A	350.53	291.00	249.25	200.86	249.29	162.88	0.026644841	-0.64
C1_10200C_A	2.69	3.13	2.81	1.69	1.81	2.46	0.044629353	-0.64
CR_08150W_A	1515.78	1087.50	1177.56	975.25	860.46	766.71	0.004010164	-0.64
C2_05550W_A	19.26	47.10	48.32	37.49	17.20	25.91	0.281776429	-0.64
C2_00140W_A	18.59	21.69	20.68	11.27	16.92	14.06	0.020148503	-0.64

C7_01400C_A	8.11	2.45	2.32	3.68	0.25	4.91	0.595183999	-0.64
C2_02900W_A	5.66	2.62	4.57	1.42	3.48	3.91	0.372122815	-0.64
CR_10530W_A	7.06	8.39	16.13	8.41	6.43	7.13	0.170134286	-0.64
C5_05040W_A	6.54	9.96	10.86	7.19	6.07	5.74	0.073212634	-0.64
C1_10870W_A	331.37	477.26	567.00	408.63	254.79	293.65	0.085459584	-0.65
C1_02030C_A	13.56	12.94	15.30	6.25	11.48	11.03	0.091020581	-0.65
C3_04550C_A	166.79	135.01	269.97	202.50	88.06	103.86	0.210063419	-0.65
C7_02940C_A	7.47	1.23	11.03	7.95	0.36	5.28	0.724748793	-0.65
C2_02930C_A	96.98	101.24	90.98	76.21	64.35	58.37	0.00214305	-0.65
C1_12540W_A	9.83	3.25	3.01	3.73	2.40	4.74	0.372902415	-0.65
C6_01880W_A	15.46	13.46	11.41	8.69	9.50	9.51	0.007343919	-0.65
C1_07240W_A	59.54	51.19	66.65	15.84	64.67	40.85	0.284663152	-0.65
C6_02940C_A	16.60	18.31	19.81	13.44	12.07	12.16	0.001977165	-0.65
C6_01690W_A	12.89	10.80	9.64	5.72	8.90	8.20	0.021924835	-0.65
C4_01790W_A	4.45	3.34	2.39	2.63	2.84	1.45	0.158238841	-0.65
CR_02960W_A	14.54	11.58	11.33	7.27	8.75	9.60	0.006446571	-0.65
C2_04420W_A	1.46	1.14	0.77	0.68	0.49	1.12	0.254790755	-0.65
C1_03920C_A	0.85	1.41	1.82	0.82	0.76	1.20	0.258331303	-0.66
C4_05800C_A	25.48	32.84	29.56	24.34	15.58	20.62	0.039542868	-0.66
C1_00740C_A	34.00	37.27	25.32	17.77	23.18	25.38	0.021072575	-0.66
C7_02760W_A	7.83	2.52	3.53	2.05	2.72	4.70	0.379904685	-0.66
C1_01400C_A	29.69	31.91	36.18	21.00	22.20	23.84	0.000755318	-0.66
C7_03200C_A	64.93	39.24	52.31	27.70	40.80	37.76	0.031050307	-0.66
C1_09680W_A	52.73	75.02	78.02	51.32	39.58	50.98	0.024661254	-0.66
C4_03290W_A	25.07	18.15	11.89	13.88	14.31	9.04	0.110246082	-0.66
C2_04320W_A	4.95	3.55	2.50	2.35	2.23	2.93	0.083970459	-0.66
C4_04430W_A	65.55	72.15	81.55	54.55	49.85	45.59	0.00339599	-0.66
C1_02010C_A	6.40	5.53	5.48	3.99	4.57	3.28	0.011923034	-0.66
CR_04870C_A	4.42	4.70	1.32	5.76	0.71	0.60	0.59123834	-0.66
C5_03620W_A	6.79	15.60	8.71	3.55	9.64	8.23	0.294057184	-0.66
C4_06560W_A	1.71	1.90	2.60	2.50	0.41	1.29	0.469883906	-0.66
C2_06550W_A	17.17	22.07	16.80	14.50	10.63	13.21	0.019564678	-0.66
C3_00150W_A	27.55	8.52	20.31	17.32	19.97	0.00	0.819034026	-0.66
C1_08730W_A	13.02	11.69	14.32	10.38	5.70	10.61	0.055564445	-0.66
C6_00020W_A	39.38	44.66	40.51	26.94	30.83	27.08	0.002791287	-0.66
C4_06120W_A	409.51	403.48	350.40	292.94	267.79	229.69	0.001190602	-0.67
CR_01270C_A	2.41	2.13	2.52	1.32	1.63	1.84	0.039805065	-0.67
C4_03470C_A	1.00	1.00	0.80	1.03	0.41	0.46	0.400364548	-0.67
C3_03550C_A	18.81	7.59	7.54	6.82	7.52	8.39	0.170165801	-0.67
C7_02420C_A	5.37	5.48	4.36	3.85	2.13	4.41	0.076859928	-0.67
C2_09770C_A	8.00	5.64	5.34	3.92	4.18	4.75	0.006954702	-0.67
CR_03510W_A	0.91	0.64	1.02	0.47	0.73	0.51	0.214089036	-0.67
C4_04180C_A	5.20	3.98	2.73	2.27	2.20	3.62	0.141561013	-0.67
C1_05990C_A	14.80	4.06	10.98	6.44	7.30	6.24	0.236537587	-0.67
C3_04120C_A	5.12	6.02	7.69	4.09	4.61	4.11	0.047656166	-0.67
C3_05320W_A	4.35	3.17	0.69	1.66	1.82	2.06	0.378362195	-0.67
CR_04410W_A	1.11	0.72	0.61	0.56	0.71	0.37	0.311557921	-0.67
C3_00720W_A	64.03	54.32	75.56	42.08	43.21	46.39	0.001018858	-0.67
C3_07670W_A	13.74	20.35	19.81	14.98	10.75	10.99	0.054392042	-0.67
C1_01510W_A	0.92	1.00	0.60	0.73	0.50	0.46	0.197688522	-0.67
C5_01000C_A	55.79	59.31	55.71	54.12	54.79	5.28	0.470769462	-0.67
C1_08920W_A	3.01	3.55	2.49	1.40	3.06	1.64	0.223761421	-0.67
C5_05010W_A	3.50	4.29	4.86	3.14	2.59	2.75	0.187293947	-0.67
C5_04920C_A	7.56	8.35	7.31	6.57	4.27	4.90	0.01189812	-0.67
C2_02880C_A	11.77	19.52	19.88	15.18	11.06	8.54	0.118784356	-0.68
C6_02300C_A	16.45	10.82	14.56	8.97	13.81	5.16	0.21621926	-0.68
C1_06840C_A	17.66	51.19	86.39	30.91	31.45	44.49	0.337640201	-0.68
C1_14530W_A	91.46	132.48	135.44	98.07	61.47	85.38	0.048363056	-0.68
C1_03010W_A	760.70	527.29	633.16	396.47	479.62	414.90	0.001877116	-0.68
CR_00710C_A	4.74	9.88	5.09	4.87	3.93	4.58	0.178929734	-0.68
C1_04470C_A	11.80	9.49	10.28	7.58	6.25	7.41	0.009184665	-0.68
C1_02870W_A	13.94	5.32	8.71	9.92	3.65	5.04	0.275219256	-0.68
C4_00880W_A	5.05	8.16	4.53	4.23	3.79	4.03	0.076822314	-0.68

C3_04360W_A	13.96	14.21	14.66	10.39	8.98	9.55	8.66E-05	-0.68
CR_00470W_A	34.19	32.12	34.57	6.77	30.21	31.18	0.302199845	-0.68
C4_02510W_A	6.79	5.56	7.94	4.32	3.55	5.81	0.032956458	-0.68
C3_02560W_A	2.63	1.28	0.86	0.96	0.70	1.52	0.327007791	-0.68
C1_13900C_A	13.11	14.83	14.62	6.74	13.88	7.91	0.092491146	-0.68
C1_11970C_A	8.46	14.02	7.92	9.01	5.34	6.24	0.117256404	-0.68
C1_14500C_A	36.94	238.44	254.83	245.95	17.59	101.27	0.686882017	-0.68
C4_00260W_A	51.26	47.10	45.18	36.22	29.64	30.47	9.55E-05	-0.69
C1_02910C_A	19.30	8.47	9.64	9.85	6.97	7.98	0.110239764	-0.69
C3_05380W_A	0.84	0.37	1.01	0.39	0.37	0.71	0.336364324	-0.69
C4_05840W_A	1.39	2.36	2.81	1.40	1.79	1.20	0.165534681	-0.69
C3_05760W_A	6.76	4.46	3.12	3.96	1.95	3.67	0.18181718	-0.69
C2_05410W_A	610.86	1021.29	1165.51	911.25	491.89	485.43	0.143149324	-0.69
C1_14350W_A	128.07	109.39	107.01	91.41	109.83	26.23	0.274252493	-0.69
C1_09320C_A	7.71	16.08	10.11	8.00	7.49	7.45	0.094187006	-0.69
C1_00090W_A	18.50	10.67	13.40	8.30	10.13	9.73	0.03281087	-0.69
C1_02820W_A	42.88	41.62	43.53	31.16	27.06	27.21	1.23E-05	-0.69
C4_03580W_A	5.38	6.76	7.53	3.83	4.02	5.34	0.034410396	-0.70
C1_09600C_A	11.08	10.84	10.89	6.79	6.96	8.10	0.000160264	-0.70
C4_03110W_A	18.53	10.51	12.74	9.52	8.07	10.08	0.011609741	-0.70
C1_02240W_A	5.61	6.04	6.82	2.09	3.97	6.34	0.158292149	-0.70
CR_02220C_A	81.43	70.31	89.34	56.86	49.07	54.74	9.42E-05	-0.70
CR_00870C_A	1.01	1.68	1.99	1.32	0.94	0.89	0.243142657	-0.70
C5_05370C_A	9.32	9.09	6.45	3.99	5.25	7.35	0.042923047	-0.70
C1_00490C_A	125.17	243.43	238.97	176.57	119.23	112.26	0.112811888	-0.70
C1_10250C_A	0.92	1.40	2.48	1.01	1.09	1.10	0.31306545	-0.70
C3_00360W_A	30.67	28.56	23.25	16.54	22.09	16.01	0.011556528	-0.70
C1_05470W_A	6.04	7.10	5.75	3.52	3.56	5.54	0.03817944	-0.70
C3_04770C_A	15.14	12.98	8.05	6.88	8.59	8.49	0.035064677	-0.70
C1_01600W_A	8.38	9.56	12.88	8.55	5.58	6.37	0.055482028	-0.70
C2_07030C_A	17.56	16.86	21.17	14.19	10.81	11.91	0.005094493	-0.70
C3_03810W_A	4.01	11.64	10.43	5.55	6.38	5.61	0.194506589	-0.70
C3_05390C_A	3.78	1.44	1.97	1.16	1.76	1.80	0.18527108	-0.70
C3_02280C_A	46.57	15.10	25.83	15.52	25.28	16.08	0.178762168	-0.70
C5_00120W_A	14.00	13.23	10.64	10.16	0.38	14.93	0.57166533	-0.70
C1_10800C_A	3.34	4.52	5.92	3.44	2.99	2.72	0.070419704	-0.70
C2_01380W_A	15.81	23.13	18.81	8.68	15.29	14.57	0.057050146	-0.70
CR_02430C_A	24.44	20.14	23.80	16.08	13.23	15.98	6.03E-05	-0.70
C3_04650W_A	2.96	2.82	3.42	2.24	1.84	1.94	0.030129964	-0.71
C5_02730C_A	11.53	16.68	21.32	13.86	9.67	9.44	0.078611788	-0.71
C3_02950C_A	160.64	191.83	186.35	118.73	128.87	109.46	0.000883997	-0.71
C5_01070C_A	7.86	6.87	7.40	5.15	5.73	3.71	0.018035414	-0.71
C4_04560C_A	58.08	61.54	51.95	37.43	39.98	35.91	0.000167832	-0.71
C2_06950C_A	9.72	11.71	12.65	7.49	7.58	7.46	0.008637144	-0.71
C1_05560W_A	169.02	155.56	162.43	99.80	114.67	106.29	2.96E-06	-0.71
C1_07770W_A	8.25	10.73	9.64	5.93	5.03	8.01	0.019369789	-0.71
C3_06700C_A	54.66	35.59	51.98	28.51	29.95	34.98	0.002892413	-0.71
C7_00010C_A	0.34	44.83	38.85	23.62	9.55	23.99	0.740164917	-0.71
C5_03030W_A	6.49	4.94	3.01	3.50	2.69	3.27	0.06882157	-0.71
C3_02340W_A	2.97	2.36	2.33	1.27	1.87	1.83	0.053625593	-0.72
C4_03770W_A	15.03	9.49	6.77	5.27	6.38	8.85	0.125699071	-0.72
C2_07590W_A	59.08	76.53	115.33	80.35	45.48	39.75	0.141789738	-0.72
CR_10520C_A	2.65	1.82	3.54	1.52	1.64	2.07	0.157390138	-0.72
C5_01700W_A	5.95	8.40	8.36	4.03	5.08	5.78	0.052207791	-0.72
C2_02940W_A	9.03	15.26	13.03	10.38	9.30	4.84	0.133237673	-0.72
CR_04230W_A	26.58	29.65	38.25	22.56	21.16	18.40	0.011197678	-0.72
C5_03650C_A	1.21	2.27	1.78	1.96	0.94	0.58	0.337716968	-0.72
C1_11770C_A	20.17	18.74	14.96	11.96	11.57	11.75	0.000191733	-0.72
C1_07050C_A	4.39	4.51	4.18	2.89	2.74	2.92	0.000500676	-0.72
C5_05410C_A	219.75	186.41	155.21	89.45	175.46	100.04	0.062400113	-0.72
C3_00090W_A	1176.79	902.92	991.27	712.87	749.50	536.20	0.003272708	-0.72
C4_04820C_A	5.09	6.45	4.79	2.76	3.92	4.05	0.048793548	-0.72
C3_00510W_A	3.50	5.28	5.99	2.57	3.06	4.11	0.089815052	-0.72

CR_10340W_A	19.11	19.78	18.49	12.40	11.81	13.43	1.71E-06	-0.72
C4_04630C_A	1.93	3.38	4.68	3.63	1.10	1.90	0.386266605	-0.72
C1_05690C_A	2.24	2.28	2.41	1.61	1.20	1.67	0.093959944	-0.72
C1_00670C_A	7.01	9.20	6.15	4.93	3.79	6.04	0.030470181	-0.72
C7_02470C_A	16.18	13.90	17.57	9.59	10.55	10.97	0.000792822	-0.72
C2_00730C_A	6.82	7.46	6.51	5.13	3.39	5.15	0.006986883	-0.72
C3_02480C_A	272.71	302.24	298.83	198.50	180.27	194.55	9.33E-06	-0.72
C5_00050W_A	24.38	17.16	7.02	6.96	11.55	12.92	0.220836204	-0.72
C3_00540C_A	23.74	20.43	25.19	17.58	9.32	18.50	0.041819742	-0.73
C1_01940C_A	4.69	1.58	3.72	1.96	1.56	2.91	0.282233586	-0.73
C1_10410W_A	10.72	11.79	9.83	6.59	6.80	7.74	0.001334817	-0.73
C7_00550C_A	16.89	21.98	17.50	4.21	16.89	15.89	0.241062477	-0.73
C2_02040W_A	9.46	9.27	7.40	6.69	5.06	5.29	0.002640371	-0.73
C5_00160W_A	8.15	15.59	13.21	10.64	6.86	6.84	0.110915062	-0.73
C1_00100C_A	33.06	20.96	26.92	15.08	19.50	17.85	0.006477832	-0.73
C7_04320W_A	13.10	7.15	8.69	6.01	7.28	5.40	0.074444462	-0.73
C2_09600C_A	25.31	41.31	49.32	26.37	22.23	27.69	0.056689024	-0.73
CR_07390C_A	11.90	17.93	23.56	13.14	9.90	12.02	0.066563281	-0.73
C4_00250W_A	16.62	12.16	9.82	7.61	6.94	10.55	0.038083118	-0.73
C3_00180C_A	18.22	7.88	14.83	12.31	1.89	12.48	0.374854506	-0.73
C2_03870W_A	6.63	9.03	10.52	6.27	3.32	7.64	0.147822954	-0.73
CR_05760C_A	20.00	10.82	16.91	8.24	12.35	10.21	0.039753333	-0.73
C2_00580C_A	41.08	36.27	45.69	29.82	23.48	26.83	0.000318192	-0.73
C4_05460C_A	27.92	19.03	19.24	12.54	13.85	16.49	0.002608156	-0.73
C7_02800W_A	24.51	23.79	15.87	17.91	11.32	12.38	0.026914761	-0.73
C3_02470C_A	245.99	314.26	364.76	227.90	179.17	197.08	0.007207357	-0.73
C6_03990C_A	4.76	5.60	5.47	4.43	2.60	3.27	0.030479019	-0.73
C1_11860W_A	40.32	38.69	38.39	24.93	24.82	26.48	6.86E-10	-0.73
C3_00480C_A	245.39	244.79	174.65	137.03	39.82	260.63	0.304722004	-0.74
CR_00280C_A	16.49	13.66	16.45	10.27	9.22	10.66	6.54E-05	-0.74
C3_06080W_A	3.54	1.27	2.63	0.29	2.38	2.09	0.423282513	-0.74
C6_03960W_A	63.24	61.77	61.09	40.49	36.16	44.15	3.11E-07	-0.74
C3_03230C_A	12.61	7.91	26.52	10.71	9.45	10.36	0.176580072	-0.74
C5_00840W_A	5.53	6.29	7.14	4.07	4.51	3.70	0.010831017	-0.74
CR_06860C_A	5.45	7.71	5.53	3.98	4.14	4.03	0.01151612	-0.74
C1_05110C_A	177.03	200.97	206.38	143.66	118.94	116.58	0.000509626	-0.74
C1_10420C_A	24.01	20.03	22.26	15.57	13.36	13.92	9.98E-06	-0.74
C7_04290W_A	109.48	48.85	58.72	48.56	45.21	44.94	0.050688983	-0.74
C1_01480C_A	714.71	649.02	1039.97	524.28	534.71	497.63	0.007641699	-0.74
C4_06270C_A	28.63	36.79	28.28	7.22	27.41	26.28	0.221617186	-0.74
C3_06120C_A	9.74	13.96	3.32	6.61	5.60	5.27	0.235067769	-0.74
C2_05330C_A	5.83	5.31	5.62	3.63	3.43	3.74	0.000313573	-0.74
C1_13800C_A	3.35	4.99	3.12	3.25	1.69	2.50	0.08823059	-0.74
C5_05080W_A	3.61	6.09	6.63	3.11	3.50	4.02	0.056689024	-0.74
CR_05300C_A	46.95	26.97	18.43	26.34	25.99	5.89	0.318175152	-0.74
C1_14330W_A	6.91	5.66	6.63	3.69	4.28	4.40	0.001319951	-0.74
C4_07170C_A	8.25	6.63	12.46	4.51	7.02	5.92	0.146050747	-0.74
CR_05310W_A	20.35	8.44	7.97	9.45	10.81	2.76	0.334994439	-0.74
C1_09080C_A	45.68	65.74	59.55	41.00	35.30	34.47	0.007049041	-0.74
C6_03480W_A	4.76	2.92	2.43	1.48	2.39	2.60	0.090904529	-0.74
C1_03180W_A	2.65	2.53	3.90	1.82	1.86	2.17	0.073241396	-0.75
CR_05800C_A	40.06	39.95	41.98	21.67	28.70	28.18	0.000956186	-0.75
C7_01620C_A	5.65	6.68	5.91	4.31	3.97	3.47	0.003175587	-0.75
C6_01520W_A	2.02	2.44	2.76	1.47	1.10	2.07	0.073012964	-0.75
C2_08100W_A	62.46	57.95	62.41	39.06	40.02	38.59	1.11E-07	-0.75
C1_07100C_A	6.40	3.52	4.55	2.08	2.41	4.76	0.142785319	-0.75
CR_01060W_A	8.60	6.47	6.99	6.02	0.06	8.28	0.709786192	-0.75
C6_02000W_A	3.23	2.99	3.97	2.80	2.34	1.38	0.082850892	-0.75
C4_01560C_A	1.88	1.07	1.29	0.73	0.84	1.15	0.063054598	-0.75
C3_06850W_A	23.68	30.55	24.63	17.77	16.30	16.85	0.000934649	-0.75
C1_04550W_A	19.74	23.08	30.05	16.63	14.45	15.72	0.019869354	-0.75
C4_01020C_A	26.49	13.89	21.68	14.28	11.55	13.81	0.013132885	-0.75
C5_03370C_A	2.35	3.45	2.89	1.80	1.88	1.84	0.130217561	-0.75

CR_04330W_A	23.39	14.50	15.46	11.01	12.57	10.36	0.007902326	-0.75
C3_07650C_A	0.61	0.94	0.67	0.49	0.21	0.70	0.284401036	-0.75
CR_10590W_A	17.43	15.20	13.45	10.73	10.19	8.52	0.000646692	-0.75
CR_10180W_A	3.64	2.43	3.31	1.91	1.88	2.18	0.069156104	-0.75
CR_01330W_A	8.68	8.21	11.59	7.44	5.37	5.47	0.010404309	-0.75
C1_10500W_A	18.92	20.72	18.56	14.27	13.26	9.68	0.011092999	-0.75
CR_00070W_A	13.35	11.57	8.39	5.51	10.98	4.60	0.116570784	-0.75
C7_01790C_A	1763.94	838.05	1063.70	859.19	748.67	711.07	0.025933872	-0.75
C4_00710W_A	20.90	21.11	23.23	13.10	13.97	14.76	7.21E-06	-0.75
C1_14030W_A	30.88	19.16	38.78	13.94	23.27	19.38	0.061270292	-0.75
CR_02300C_A	15.72	15.82	15.78	11.36	7.63	11.42	0.001733905	-0.76
C4_02270C_A	65.96	69.67	106.39	57.16	55.37	42.33	0.023246988	-0.76
C4_02700W_A	2.60	3.15	2.40	1.44	1.74	2.00	0.019978937	-0.76
C5_02520W_A	9.97	3.84	3.24	1.78	3.33	5.66	0.300014037	-0.76
C6_00080C_A	10.94	11.13	10.00	6.14	8.21	6.09	0.004431794	-0.76
C3_06920W_A	67.65	69.37	67.82	38.15	45.94	46.85	1.71E-05	-0.76
C6_01680C_A	2.16	2.40	3.14	1.98	1.33	1.60	0.072361251	-0.76
C1_03400W_A	31.49	23.02	21.14	16.42	16.35	15.23	0.000689782	-0.76
CR_03950W_A	4.69	8.52	6.31	5.00	3.20	4.32	0.084721265	-0.76
C6_01710C_A	16.78	15.72	15.20	9.45	10.24	10.73	2.99E-07	-0.76
C3_04680W_A	807.67	714.62	815.34	490.44	541.51	455.41	1.67E-05	-0.76
C2_06590C_A	9.30	5.70	5.60	4.25	3.57	5.24	0.021893338	-0.76
C4_04270W_A	15.91	13.23	20.79	8.43	14.72	8.46	0.06802715	-0.76
C5_04310W_A	2.65	2.00	1.97	1.29	1.54	1.38	0.020511522	-0.76
C4_04860W_A	13.36	16.24	12.60	9.43	9.89	7.53	0.005848629	-0.76
C3_01660W_A	73.21	76.11	75.16	47.00	44.60	51.39	4.79E-06	-0.76
CR_03690W_A	9.15	14.15	15.46	8.68	6.80	9.42	0.025809488	-0.76
CR_02260C_A	11.19	14.05	14.50	9.22	8.08	8.02	0.003451366	-0.77
C1_11110C_A	4.15	4.09	6.69	4.61	2.53	2.37	0.197335041	-0.77
C1_09570W_A	7.40	3.87	5.50	2.74	4.13	3.71	0.041066148	-0.77
CR_02950C_A	14.37	33.58	25.45	27.11	12.14	7.57	0.268368773	-0.77
C4_04600C_A	25.10	18.73	23.46	16.33	2.79	24.06	0.339574129	-0.77
C7_01920W_A	585.80	254.44	273.60	226.69	228.16	241.66	0.049506115	-0.77
C1_00170W_A	153.00	142.65	105.44	86.79	85.47	81.40	0.000433055	-0.77
C2_06200C_A	7.05	7.94	6.24	3.40	4.84	5.23	0.007967566	-0.77
C7_02750W_A	2.62	5.32	4.31	2.71	3.23	1.85	0.125089243	-0.77
C1_13980C_A	4.26	2.23	5.87	2.75	2.45	2.61	0.095883248	-0.77
C3_03120C_A	4.09	4.89	1.55	1.99	2.35	2.31	0.172869142	-0.77
C1_10700C_A	89.91	107.05	109.72	71.79	59.04	64.10	0.000326065	-0.77
C1_10960W_A	5.17	8.45	10.67	5.90	4.56	4.97	0.086370903	-0.77
C7_00950W_A	14.71	9.85	12.92	7.44	8.50	7.65	0.002880591	-0.77
C2_04390W_A	4.22	2.85	4.74	2.76	0.88	3.89	0.273219344	-0.77
C2_07020C_A	2.21	3.70	3.23	2.12	1.85	1.83	0.064499047	-0.77
C1_00220W_A	333.38	328.57	353.81	231.98	196.31	213.84	6.81E-07	-0.77
C3_06180C_A	538.32	546.04	550.68	381.57	575.64	57.82	0.364734239	-0.78
C4_05530W_A	9.40	12.76	10.42	6.82	6.76	7.00	0.002116814	-0.78
C1_11880W_A	11.25	13.32	13.41	8.49	7.24	8.19	0.006349144	-0.78
C5_02740W_A	1.99	1.14	3.18	0.95	1.26	1.74	0.179781641	-0.78
C3_02360C_A	0.53	0.48	0.96	0.26	0.30	0.68	0.312724075	-0.78
C4_01190W_A	25.21	20.67	15.12	12.23	10.73	15.33	0.005673588	-0.78
C5_03840W_A	4.67	3.04	3.68	2.68	1.89	2.51	0.026448747	-0.78
C1_08750W_A	10.93	14.53	13.65	7.92	8.94	7.68	0.004704033	-0.78
C4_07180W_A	1476.86	1285.70	1548.80	535.84	1178.39	987.47	0.036945351	-0.78
C4_04390W_A	153.49	238.75	332.99	213.19	117.54	127.20	0.093956567	-0.79
CR_06570C_A	6.00	3.74	2.68	2.22	2.53	2.98	0.052939256	-0.79
C6_02610C_A	16.93	20.61	19.86	15.76	10.62	9.62	0.013794172	-0.79
C1_01920W_A	3.44	3.46	3.97	2.50	2.20	2.10	0.000481737	-0.79
C1_11980W_A	7.24	14.82	7.81	11.44	2.20	5.17	0.324064684	-0.79
C7_00910C_A	12.19	9.87	10.59	5.90	7.26	7.19	0.000222944	-0.79
C4_02250C_A	172.55	185.81	225.88	129.91	111.25	125.42	0.000211866	-0.79
C1_13030C_A	27.36	25.05	25.88	18.92	16.47	13.41	0.000503498	-0.79
C1_02770W_A	112.84	105.77	87.72	59.71	69.16	62.33	0.000208078	-0.79
C1_08500C_A	7173.96	7162.00	5967.29	3820.45	4386.18	4489.15	5.93E-06	-0.79

C3_05110W_A	23.42	17.60	17.44	12.20	11.44	12.76	9.34E-05	-0.79
C3_05010C_A	5.69	5.66	6.85	3.77	3.78	3.82	0.000189076	-0.79
CR_00270C_A	26.19	20.17	21.29	14.49	12.77	14.87	0.000243845	-0.79
C6_04550C_A	25.55	27.40	30.28	19.66	14.30	18.16	0.000407343	-0.79
C1_10660W_A	57.18	54.78	65.52	30.67	52.37	27.08	0.035208382	-0.79
CR_08310C_A	3.32	2.55	3.60	2.26	1.88	1.74	0.021898976	-0.79
C3_00800W_A	18.60	20.78	25.84	10.18	7.34	23.79	0.134769103	-0.79
C1_09390W_A	3.91	2.49	4.10	1.96	1.69	2.85	0.043939442	-0.79
C4_03960W_A	207.28	91.31	131.72	57.99	84.81	123.39	0.082153152	-0.79
C2_00260C_A	93.80	104.45	80.13	64.15	58.00	51.10	0.000523664	-0.80
C7_02910W_A	17.41	20.98	23.37	6.85	9.76	22.42	0.133484449	-0.80
C2_04110W_A	0.87	1.24	1.26	0.54	0.56	1.01	0.081667047	-0.80
CR_03230W_A	24.94	17.81	18.15	13.38	11.83	12.49	0.000111487	-0.80
C1_10000C_A	18.44	18.71	14.75	10.68	11.40	10.16	0.000142354	-0.80
C5_00570W_A	9.28	8.43	8.33	2.99	5.81	7.43	0.04651361	-0.80
C5_02320C_A	8.10	11.42	9.87	7.80	5.02	5.49	0.020132949	-0.80
C1_03240W_A	36.30	36.14	38.45	20.76	21.34	26.73	0.000107046	-0.80
CR_02210W_A	6.37	6.70	5.85	4.66	3.84	3.24	0.00197696	-0.80
C1_02480W_A	22.57	9.09	10.87	4.86	11.90	9.17	0.159685627	-0.80
C6_00490W_A	4.39	3.22	3.16	2.14	2.43	2.10	0.00739847	-0.80
C2_03220C_A	33.78	35.39	33.83	26.21	12.65	25.26	0.024816829	-0.80
C1_10710C_A	2.65	2.84	1.78	1.23	1.81	1.42	0.04651361	-0.81
C1_11580W_A	61.44	77.76	89.24	46.89	51.54	42.46	0.003680094	-0.81
C3_05870C_A	10.00	6.92	9.80	4.48	4.46	7.48	0.012951971	-0.81
C4_05940W_A	2.51	2.40	2.16	1.28	1.53	1.52	0.00065485	-0.81
C1_00810W_A	0.39	2.32	0.91	0.49	1.24	0.49	0.463101296	-0.81
C4_04770C_A	39.65	61.08	44.00	36.26	25.09	28.12	0.011003358	-0.81
C7_02840C_A	18.91	46.71	101.93	66.57	19.54	17.75	0.364268794	-0.81
C7_01100C_A	13.11	15.99	12.67	10.08	8.38	7.21	0.002975047	-0.82
C2_00800C_A	1.90	3.15	1.60	1.87	0.40	1.85	0.292477886	-0.82
C1_09290C_A	29.84	37.47	43.06	24.25	23.55	20.06	0.002258801	-0.82
CR_10510W_A	7.61	7.36	12.04	4.87	5.54	6.21	0.009981511	-0.82
C2_07070W_A	30.14	36.79	34.42	20.00	17.85	24.60	0.000418064	-0.82
C4_06290W_A	5.36	6.40	4.30	1.78	3.58	4.45	0.167969755	-0.82
C1_12930C_A	2.06	1.18	0.91	0.84	0.76	0.93	0.112537051	-0.82
C1_12200W_A	2.44	2.30	1.90	0.27	1.64	2.16	0.308295303	-0.82
C5_00550C_A	19.24	21.88	21.38	14.42	10.90	12.97	0.002473163	-0.82
C1_12970C_A	33.23	31.48	41.86	19.15	17.88	28.57	0.003782014	-0.82
C2_03750W_A	32.96	36.78	42.31	27.86	20.87	19.95	0.00238256	-0.82
C3_02890C_A	13.39	11.98	11.69	8.43	9.69	4.33	0.073447826	-0.82
C2_10420W_A	5.69	10.85	9.94	5.80	5.31	5.20	0.034705042	-0.82
C3_02140C_A	6.81	5.74	6.28	3.11	3.85	4.50	0.001326557	-0.82
C2_07230C_A	7.23	8.94	5.73	3.99	4.93	4.46	0.004643446	-0.82
C4_05030C_A	10.03	17.15	14.83	8.44	8.72	8.67	0.008515826	-0.82
C3_04790W_A	9.94	14.49	6.74	6.65	5.96	6.50	0.035883022	-0.82
C3_05780C_A	33.39	27.43	17.01	20.71	7.02	19.79	0.124290865	-0.83
C3_03990C_A	3.97	4.32	4.56	2.50	2.68	2.64	0.000462236	-0.83
C4_05850C_A	10.64	14.28	15.35	5.07	10.63	8.86	0.04651361	-0.83
C6_01940W_A	18.56	10.14	21.47	12.26	7.57	10.50	0.081323207	-0.83
C3_02710W_A	333.86	344.89	326.50	195.54	190.18	227.22	9.49E-08	-0.83
C6_01350W_A	10.89	7.75	5.67	4.21	4.95	5.55	0.011620745	-0.83
C1_04580C_A	10.86	13.58	15.17	9.92	7.82	6.37	0.014914162	-0.83
C6_04430W_A	10.81	8.69	8.94	7.97	6.60	2.57	0.120815164	-0.83
C1_14210C_A	61.02	71.45	64.70	61.51	3.05	56.98	0.565672591	-0.83
C1_10640C_A	7.16	6.13	4.87	2.43	4.53	4.01	0.022274693	-0.83
C4_01260W_A	9.50	5.94	4.97	3.62	3.76	4.94	0.01718431	-0.83
C1_10210C_A	9.08	6.42	4.67	5.10	3.94	3.09	0.030917019	-0.83
C2_07240C_A	3.52	6.38	1.58	3.25	2.19	1.54	0.243162614	-0.83
CR_10190C_A	5.06	3.59	4.52	2.39	2.82	2.73	0.002640371	-0.83
C3_06420C_A	7.52	9.34	10.03	6.03	4.75	5.53	0.002648946	-0.83
C1_02570C_A	1.91	2.10	1.82	1.10	1.28	1.15	0.009939031	-0.83
C7_02500C_A	7.02	3.79	3.79	2.18	3.00	3.58	0.057793431	-0.83
C7_02510W_A	12.81	7.18	9.77	4.34	7.20	6.19	0.046909382	-0.83

C4_04930C_A	12.62	10.17	12.09	8.45	4.94	7.68	0.006309219	-0.84
C2_10250C_A	5.67	4.53	4.59	2.74	2.46	3.76	0.001274063	-0.84
C1_07180W_A	207.88	215.74	272.31	199.04	23.84	203.86	0.307278883	-0.84
C1_02980W_A	35.13	105.47	140.85	72.09	48.73	51.68	0.171171177	-0.84
C1_00860W_A	8.36	7.69	4.63	2.94	3.67	5.86	0.060737466	-0.84
C6_03330C_A	6.47	4.27	3.99	2.10	3.21	3.54	0.016329825	-0.84
C3_03870C_A	121.09	127.26	122.05	75.39	76.27	71.88	3.12E-08	-0.84
C2_07270W_A	78.14	116.61	70.61	46.49	70.53	43.15	0.028804146	-0.84
C1_13910C_A	11.46	12.58	12.79	7.41	6.81	8.02	1.38E-05	-0.84
C3_00640W_A	10.72	16.07	15.32	10.13	7.42	7.96	0.008489209	-0.84
C4_00360C_A	5.52	5.59	4.88	3.43	2.84	3.36	0.00059075	-0.84
C5_02370C_A	12.10	8.59	9.22	5.31	6.08	6.54	0.000308563	-0.84
C1_03510C_A	287.81	377.08	389.76	224.45	204.03	209.80	0.00032026	-0.84
C1_09000W_A	6.21	4.86	8.46	4.11	3.73	3.91	0.00808361	-0.84
C4_05320W_A	195.68	113.56	109.29	77.39	81.04	91.03	0.003573283	-0.85
C2_08800C_A	58.35	64.82	60.05	36.51	35.62	38.15	8.15E-08	-0.85
C1_00510W_A	25.05	27.94	31.18	17.86	16.61	16.10	8.89E-05	-0.85
CR_01260W_A	4.47	3.85	3.38	1.82	2.29	2.88	0.003239991	-0.85
C1_09640W_A	225.38	266.28	267.01	162.97	145.81	147.84	1.21E-05	-0.85
C1_09150W_A	16.75	8.33	19.87	8.89	8.91	9.02	0.036131218	-0.85
C2_10630W_A	16.30	13.96	14.98	8.59	9.04	9.46	4.44E-08	-0.85
C2_06440C_A	7.28	11.69	11.53	7.51	5.04	5.89	0.030013449	-0.85
CR_00510C_A	104.20	149.58	173.92	25.16	118.59	114.90	0.209362105	-0.85
C1_12030W_A	212.01	67.63	133.75	108.88	50.25	85.29	0.109944927	-0.85
C7_00310C_A	22.39	28.36	22.50	15.01	15.51	13.35	0.000610412	-0.85
C1_00630W_A	18.17	23.20	15.26	14.41	9.46	10.08	0.008822865	-0.85
C1_06500W_A	9.33	8.73	10.79	6.26	5.34	5.59	0.000914781	-0.86
C6_02750C_A	2.59	2.91	2.34	1.28	1.79	1.58	0.007531187	-0.86
C7_03440W_A	8.06	5.75	9.02	5.48	5.21	2.82	0.04112059	-0.86
C3_05930W_A	44.70	30.54	46.18	17.10	33.55	21.18	0.023809401	-0.86
C2_00350W_A	11.31	11.16	9.84	7.78	6.93	4.46	0.006717699	-0.86
C2_08270C_A	10.10	9.41	13.77	4.69	11.61	3.32	0.165613889	-0.86
C1_14130W_A	279.05	330.13	359.43	203.73	182.12	193.64	3.10E-05	-0.86
C5_03610W_A	33.45	44.34	27.18	9.10	30.36	23.18	0.117908522	-0.86
C4_07010C_A	14.96	26.46	24.72	15.95	11.33	12.40	0.027866813	-0.86
CR_06700C_A	95.27	126.33	118.22	70.02	67.21	65.79	0.000147611	-0.86
C5_00090C_A	69.53	72.14	30.92	51.62	1.63	50.49	0.603535711	-0.86
C6_01340C_A	12.99	10.96	10.05	7.79	5.36	7.07	0.002026539	-0.86
C1_10450W_A	61.19	82.41	89.83	61.30	32.35	46.19	0.016898673	-0.86
C2_09390W_A	33.69	23.55	26.67	6.48	18.52	24.85	0.120774269	-0.86
C2_09950W_A	69.85	38.84	29.26	15.14	33.96	31.94	0.086134821	-0.86
CR_00180C_A	6.41	8.19	5.98	1.04	5.39	5.84	0.236085794	-0.86
C1_04940C_A	4.89	3.34	5.00	3.74	2.34	1.71	0.10244393	-0.86
C1_12550C_A	2.11	1.26	2.20	1.00	0.82	1.47	0.040475443	-0.87
CR_01050C_A	4.91	3.52	3.30	3.06	0.00	3.98	0.736395033	-0.87
C1_05540C_A	126.92	91.49	129.94	67.69	62.73	75.50	3.52E-05	-0.87
C3_02290W_A	10.01	6.55	7.72	4.83	3.93	5.53	0.001957062	-0.87
C1_14450C_A	41.45	41.43	73.19	54.43	24.42	13.20	0.182808656	-0.87
C4_05380C_A	5.71	6.99	12.70	3.27	4.77	6.98	0.169686294	-0.87
C1_08150C_A	2.92	2.66	1.75	1.85	1.42	1.02	0.118497914	-0.87
C3_06410C_A	5.88	7.41	7.19	3.57	4.41	4.17	0.002592511	-0.87
CR_10380C_A	3.85	1.39	3.12	1.88	1.62	1.37	0.120079015	-0.87
C1_03860C_A	6.48	3.77	3.72	1.71	2.76	3.75	0.039866825	-0.87
CR_09310W_A	10.37	3.74	7.40	2.71	4.58	5.27	0.087819877	-0.87
CR_06380C_A	13.78	16.37	13.09	8.46	8.48	8.65	3.90E-05	-0.87
C1_01750W_A	7.67	10.55	9.62	8.07	4.81	3.55	0.048197733	-0.87
C4_04570W_A	14.40	19.90	22.11	10.60	10.97	11.85	0.002905104	-0.87
C1_05300C_A	112.11	152.30	101.71	97.41	55.43	63.78	0.013515795	-0.87
C6_01470W_A	3.12	1.94	1.59	1.45	1.27	1.17	0.037739487	-0.88
C3_01900C_A	25.69	15.64	16.65	12.29	9.92	11.70	0.001088823	-0.88
C4_03180W_A	60.55	50.69	58.96	32.55	31.69	35.88	5.77E-10	-0.88
C1_14460W_A	53.88	137.26	163.30	167.17	10.27	33.65	0.576819399	-0.88
C1_08570C_A	5.26	7.40	5.64	2.90	4.06	3.77	0.015452102	-0.88

CR_02820W_A	106.94	108.16	123.86	80.94	37.94	81.94	0.018443623	-0.88
C4_03520C_A	6.00	4.55	6.14	3.33	3.20	3.26	0.000110666	-0.88
C4_00890W_A	41.48	64.31	55.42	35.73	29.34	30.37	0.002055773	-0.88
C1_11560C_A	7.99	8.00	7.17	6.00	4.96	2.55	0.046362624	-0.88
C1_05600W_A	4.96	5.02	3.91	2.55	2.44	3.17	8.83E-05	-0.88
C4_04380C_A	3.27	1.70	5.25	2.21	1.09	2.50	0.275385823	-0.88
C4_06670W_A	15.24	18.57	18.30	9.91	10.04	10.70	4.89E-05	-0.88
CR_03780C_A	5.98	5.71	5.96	3.77	3.57	3.01	0.00261234	-0.88
C4_05450C_A	31.53	24.70	29.64	12.51	14.31	23.59	0.006031215	-0.88
C3_06690C_A	42.65	39.53	39.77	23.40	23.55	24.46	2.20E-07	-0.88
C2_07290W_A	55.26	101.14	72.90	46.89	61.53	26.08	0.071190709	-0.88
C4_05170C_A	10.71	11.54	18.38	9.19	6.58	8.14	0.013087378	-0.88
C5_02690W_A	49.95	57.46	67.95	31.90	30.01	41.49	0.00047088	-0.88
C1_00210C_A	259.24	208.47	255.84	154.69	121.12	147.61	1.78E-06	-0.88
C1_12010C_A	3.20	2.52	2.66	2.08	0.69	2.13	0.092310172	-0.88
C2_06810C_A	231.46	237.21	298.96	152.96	141.77	155.76	1.21E-05	-0.88
CR_05290W_A	1.96	1.46	1.87	0.65	0.76	1.66	0.123651572	-0.88
CR_10260W_A	14.03	13.93	13.43	7.61	8.91	7.64	4.13E-05	-0.89
C5_03060C_A	9.30	10.81	10.49	5.43	6.17	6.26	4.57E-05	-0.89
C4_05560C_A	7.19	5.90	8.22	3.97	3.91	4.55	0.000252468	-0.89
C2_09610W_A	2.92	3.25	6.12	2.39	2.52	2.28	0.060890552	-0.89
C3_04180W_A	99.52	104.19	94.18	62.42	51.09	60.90	2.18E-07	-0.89
C1_02380C_A	4.83	2.75	2.36	1.52	0.88	3.37	0.224145474	-0.89
C4_06550C_A	6.38	6.41	7.53	4.37	3.33	4.17	5.28E-05	-0.89
C1_12380C_A	17.07	14.47	15.34	8.54	8.62	10.14	2.64E-07	-0.89
C7_03640C_A	11.36	13.88	15.30	9.62	8.53	5.44	0.012966189	-0.89
C7_02320W_A	1.05	3.67	3.94	1.34	1.41	2.38	0.191866208	-0.89
CR_05040W_A	3.97	2.73	2.29	1.04	1.57	2.34	0.197202	-0.90
C1_09480W_A	23.92	33.35	34.26	16.11	17.86	19.35	0.002184742	-0.90
C5_02650C_A	12.91	12.52	13.44	8.75	6.55	7.27	1.07E-05	-0.90
C3_06680C_A	24.19	14.75	15.14	9.28	10.21	11.70	0.001807605	-0.90
C1_01190C_A	26.43	28.99	31.59	15.47	18.40	16.68	1.60E-05	-0.90
C2_05280C_A	8.10	6.87	6.46	3.16	5.38	3.81	0.008045759	-0.90
C2_00060C_A	16.85	52.75	91.78	39.81	14.85	41.05	0.246783134	-0.90
C1_03910C_A	3.57	2.26	2.24	1.23	1.35	2.07	0.020101757	-0.90
C1_10020W_A	16.47	11.05	11.78	8.25	7.41	6.99	0.000236762	-0.90
CR_02380C_A	4.38	3.40	5.74	3.88	1.98	1.94	0.126337887	-0.90
C1_10830W_A	13.32	7.39	10.24	4.27	7.55	5.93	0.01939684	-0.90
C5_02290W_A	8.93	9.50	10.24	5.18	5.21	6.22	3.97E-05	-0.90
C5_00770C_A	8.39	6.05	7.41	3.96	4.21	4.43	1.24E-05	-0.90
C2_05120C_A	0.85	3.57	3.24	0.17	3.67	0.47	0.546475107	-0.90
C4_03930C_A	9.11	7.42	4.90	4.12	3.72	4.48	0.026644841	-0.90
C1_00150C_A	121.95	90.28	83.96	48.67	60.95	61.09	5.84E-05	-0.90
C3_01020W_A	24.19	25.47	15.80	12.68	12.17	13.05	0.000809957	-0.90
C7_01280C_A	9.11	9.01	8.96	5.19	5.15	5.30	1.60E-06	-0.90
C1_10630C_A	3.12	1.80	1.65	1.11	1.56	1.08	0.029992701	-0.90
C2_00550W_A	7.31	8.20	9.18	4.95	5.16	4.16	0.000463074	-0.90
C3_07620C_A	6.95	13.26	8.39	4.50	5.91	6.23	0.024657421	-0.90
C3_00170C_A	33.87	43.22	94.35	61.92	8.43	29.52	0.295521236	-0.90
C1_09010W_A	15.82	9.18	11.35	7.84	6.53	6.48	0.001324746	-0.90
C3_07180C_A	2.97	1.39	1.17	1.26	1.02	0.86	0.089482269	-0.91
CR_03630W_A	22.99	22.20	18.75	11.32	14.02	11.35	1.92E-05	-0.91
C2_04300C_A	9.50	6.39	8.62	4.92	5.53	3.60	0.009088904	-0.91
C2_01970C_A	6.35	5.93	5.09	3.54	3.22	3.20	6.16E-06	-0.91
C4_03950C_A	27.08	18.11	25.75	14.89	13.86	11.94	0.002195043	-0.91
C7_02150C_A	44.76	55.41	51.62	35.89	28.18	23.14	0.001547963	-0.91
C1_02780W_A	149.40	171.66	171.39	94.84	88.90	99.90	1.86E-07	-0.91
C5_03860W_A	25.25	22.59	21.90	16.79	10.19	13.04	0.000932986	-0.91
C2_06210C_A	150.24	159.39	142.83	42.88	109.61	107.80	0.036597246	-0.91
C5_01310W_A	9.88	8.11	6.49	4.75	5.00	4.30	0.003140354	-0.91
C4_05300W_A	51.55	41.40	32.25	24.01	21.95	25.53	7.73E-05	-0.92
C7_03680W_A	11.25	13.40	12.21	6.74	8.90	5.43	0.003303651	-0.92
C2_09860C_A	5.55	8.32	2.91	2.98	3.83	2.81	0.078221107	-0.92

C4_06780C_A	75.01	35.11	60.93	24.50	37.43	34.99	0.013265808	-0.92
C5_02240W_A	2.64	2.76	1.15	0.94	0.39	2.38	0.285264045	-0.92
CR_09160C_A	59.65	64.93	78.97	40.45	36.55	39.47	1.32E-05	-0.92
C2_04990W_A	7.41	7.17	5.85	4.01	4.55	3.04	0.00500609	-0.92
C5_03870C_A	90.49	90.47	116.88	76.32	38.89	55.21	0.00726626	-0.92
C2_00520W_A	67.60	43.13	77.55	10.32	48.40	48.56	0.168936763	-0.92
CR_00170W_A	19.79	29.39	16.77	4.64	16.65	16.58	0.127045407	-0.92
C1_14320C_A	146.72	157.52	161.04	87.37	89.12	89.04	3.44E-09	-0.92
C2_01090C_A	10.94	3.92	6.41	3.59	3.77	4.56	0.083970459	-0.92
C3_06040W_A	4.66	4.28	4.55	4.07	1.50	2.09	0.056896942	-0.92
C6_00740W_A	13.69	16.13	16.58	10.45	8.14	7.87	0.000222786	-0.92
CR_04810W_A	267.57	348.85	470.38	261.32	181.34	179.20	0.008687004	-0.92
C1_04030W_A	6.28	5.47	4.56	2.90	2.87	3.49	7.62E-05	-0.92
C2_08420W_A	5.73	6.83	5.54	2.71	3.13	4.40	0.004272694	-0.93
CR_02000C_A	42.04	32.43	41.67	20.25	22.74	22.64	3.71E-07	-0.93
C1_05790W_A	26.73	30.91	33.33	18.51	14.26	18.70	0.001242503	-0.93
C7_03470W_A	2.34	4.73	3.73	2.55	2.33	1.27	0.118920471	-0.93
C3_00980W_A	4.32	5.04	5.54	2.52	3.19	2.72	0.00047899	-0.93
C1_07370C_A	6.81	6.38	5.73	2.77	4.94	2.92	0.008629727	-0.93
CR_07630C_A	391.24	408.29	499.00	286.01	217.21	231.59	6.75E-05	-0.94
C5_01960C_A	135.60	101.54	141.19	91.79	54.59	66.80	0.001416851	-0.94
C4_03500C_A	1.83	3.16	4.32	2.20	1.94	1.06	0.165065876	-0.94
CR_00700W_A	0.88	3.67	2.35	0.43	1.36	2.18	0.304792835	-0.94
C1_05270C_A	21.10	21.57	22.35	11.61	12.44	12.61	3.79E-07	-0.94
CR_02740W_A	35.03	29.77	22.81	18.46	13.62	17.30	0.000199718	-0.94
CR_01960C_A	5.07	4.84	5.52	3.01	2.53	3.13	9.01E-05	-0.94
CR_06020W_A	4.27	5.38	6.14	3.29	2.74	2.84	0.003563775	-0.94
CR_04470W_A	4.98	3.94	4.36	3.34	3.39	0.60	0.221682556	-0.94
C2_03830W_A	5.90	7.12	9.53	4.18	3.61	4.95	0.00323079	-0.94
C1_14490C_A	8.86	14.41	16.62	16.93	1.28	4.37	0.346391524	-0.94
C4_05290W_A	8.66	5.94	4.77	3.00	3.83	4.00	0.002937518	-0.94
C2_03440W_A	64.80	65.76	60.35	39.61	34.74	32.84	2.50E-07	-0.94
C5_04620C_A	4.64	6.82	5.41	3.55	2.62	3.29	0.007296724	-0.94
C2_00390C_A	63.55	71.91	75.54	53.01	22.52	43.66	0.014563113	-0.95
C6_01650C_A	8.69	6.02	7.97	4.66	3.80	4.21	9.70E-06	-0.95
C6_02640C_A	10.32	16.67	14.47	12.78	7.86	2.52	0.169314123	-0.95
CR_03010C_A	39.13	36.75	34.45	21.76	19.35	20.60	2.53E-08	-0.95
CR_01810C_A	3.46	2.00	2.67	2.16	1.89	0.44	0.216153	-0.95
C3_04200W_A	7.67	5.05	4.64	3.21	3.07	3.35	0.001849837	-0.95
C2_02610C_A	8.00	5.13	6.92	4.37	3.09	3.70	0.001031983	-0.95
C4_03910W_A	3.70	4.33	4.69	1.79	2.18	3.13	0.004798394	-0.95
C5_02270W_A	87.08	98.17	105.91	56.17	44.62	62.57	1.56E-05	-0.95
C2_10120W_A	4.63	4.67	4.25	1.68	2.88	2.94	0.01070091	-0.95
C1_05870W_A	7.55	16.44	6.16	3.96	5.97	7.07	0.08695846	-0.95
C7_01750W_A	3.50	3.03	3.62	1.86	1.83	1.95	0.002960379	-0.96
C3_06310C_A	6.22	3.66	3.45	2.58	2.34	2.44	0.00238256	-0.96
C3_06870W_A	20497.50	24540.93	20483.28	11541.09	14126.82	10798.41	4.33E-05	-0.96
C3_02510C_A	0.58	0.16	1.79	0.75	0.18	0.46	0.443661075	-0.96
C7_03090C_A	4.34	4.87	6.44	3.91	1.52	3.31	0.047629815	-0.96
C1_13450W_A	5.92	6.08	4.87	2.64	3.78	2.91	0.00039897	-0.96
C1_10270C_A	14.51	18.50	21.22	13.41	7.05	9.81	0.00932147	-0.96
CR_00240W_A	11.01	23.84	46.65	29.51	4.17	11.92	0.312440273	-0.97
C7_00700W_A	50.17	41.17	44.47	21.81	26.65	26.31	1.03E-06	-0.97
C4_04940W_A	2.10	1.64	3.23	1.52	1.05	1.21	0.061246924	-0.97
C5_02630C_A	26.78	21.53	16.03	13.97	9.84	11.55	0.000670969	-0.97
C3_07580W_A	106.10	171.40	190.02	71.02	96.32	92.47	0.005872936	-0.97
C7_00970C_A	6.47	7.63	7.77	5.30	3.00	3.79	0.005082193	-0.97
CR_03870W_A	30.53	22.70	24.18	15.23	9.25	18.02	0.005302533	-0.97
CR_07470W_A	36.55	33.84	34.42	18.49	19.54	19.57	1.22E-08	-0.97
C3_05340W_A	1.56	1.09	0.68	0.72	0.64	0.44	0.115614077	-0.97
CR_06440C_A	20.06	15.65	13.63	8.41	9.17	9.48	4.58E-06	-0.97
C7_03320C_A	6.69	4.89	2.26	2.49	2.73	2.33	0.037894363	-0.97
C2_03070C_A	1.22	1.38	0.92	0.55	0.73	0.62	0.038901343	-0.97

C3_07820W_A	87.13	70.00	75.19	50.68	42.53	34.08	0.000132819	-0.97
C3_06600C_A	6.02	3.64	4.74	2.51	1.74	3.66	0.013664945	-0.98
C3_02080W_A	10.16	8.68	7.69	4.06	4.52	5.96	4.40E-05	-0.98
C2_00340C_A	252.10	281.79	239.02	158.70	154.13	110.38	0.000138682	-0.98
C1_06480C_A	29.62	25.78	26.80	14.02	11.59	19.52	0.00026878	-0.98
C1_12900W_A	6.51	6.19	7.07	3.59	3.47	3.77	2.06E-07	-0.98
C6_02580W_A	26.99	27.03	23.98	14.86	16.19	11.58	4.39E-05	-0.98
C5_03940C_A	5.28	2.75	3.84	1.81	2.21	2.43	0.007010178	-0.98
C4_06310C_A	1.61	2.80	1.81	1.16	1.37	0.87	0.071698599	-0.98
C6_03710W_A	6.37	7.37	6.80	3.88	3.88	3.51	6.11E-07	-0.98
C4_00580W_A	33.74	29.95	31.13	13.83	18.13	19.82	1.67E-05	-0.98
CR_10640W_A	9.52	10.88	10.27	2.85	7.11	6.88	0.026884567	-0.98
C3_05420W_A	0.58	4.31	3.37	0.12	0.05	4.57	0.683881016	-0.98
CR_00410W_A	4.69	6.26	10.83	4.38	3.59	4.00	0.030646436	-0.98
C1_02640C_A	26.73	68.30	71.13	45.35	22.73	23.79	0.085636622	-0.98
C4_01370W_A	89.53	122.29	141.96	64.58	61.37	68.62	0.000296317	-0.98
C4_01380W_A	5.02	3.72	4.00	2.18	2.30	2.45	1.29E-06	-0.99
C2_08980C_A	8.11	10.35	8.45	5.03	4.92	4.79	6.47E-06	-0.99
C7_03040W_A	3.63	8.63	1.11	2.41	1.20	3.70	0.311495696	-0.99
C1_04980C_A	17.86	12.00	21.65	9.54	9.78	8.64	0.001102205	-0.99
C3_05300C_A	3.97	6.72	3.85	1.66	4.35	1.90	0.088295165	-0.99
CR_01030W_A	1.49	1.55	1.23	1.24	0.00	1.12	0.512447035	-0.99
C3_04510W_A	4.30	3.88	4.24	2.31	1.12	3.26	0.113808579	-0.99
C2_02360C_A	5.74	9.06	7.09	3.09	5.43	3.39	0.022252638	-0.99
C7_04240C_A	178.05	88.48	87.71	63.80	63.45	62.99	0.003537009	-0.99
C3_05730C_A	4.75	2.29	2.24	1.20	2.19	1.58	0.053932634	-0.99
C5_02540C_A	2.15	1.71	1.26	0.75	0.80	1.19	0.035465331	-0.99
C4_05920C_A	4.24	6.91	6.45	3.66	2.68	3.30	0.003883923	-0.99
C1_02420C_A	13.53	15.18	15.29	7.74	8.40	7.78	3.64E-08	-0.99
C4_05730W_A	17.32	22.79	12.70	10.55	10.12	7.92	0.005582727	-0.99
C1_07200W_A	30.00	40.84	45.28	25.98	20.68	16.43	0.003028776	-0.99
C3_07330W_A	26.01	39.87	36.69	22.04	15.81	18.13	0.001371654	-0.99
C3_06590W_A	58.63	115.43	132.65	76.17	35.12	57.22	0.042590442	-0.99
CR_03280W_A	49.24	53.11	57.96	30.94	28.53	27.51	1.49E-07	-1.00
C5_03040W_A	13.38	9.84	7.68	6.98	5.13	4.49	0.00355055	-1.00
C6_03610W_A	8.20	5.54	5.97	2.89	2.85	4.89	0.002813495	-1.00
CR_03930C_A	9.54	11.27	10.25	6.58	4.40	5.83	0.000192195	-1.00
C5_02940C_A	43.25	28.15	32.87	21.04	17.37	17.59	1.62E-05	-1.00
C1_00780C_A	1.92	2.49	2.26	0.95	1.01	1.64	0.007453263	-1.00
C1_09730W_A	12.77	17.02	21.35	22.55	1.14	3.91	0.365534884	-1.00
CR_06340C_A	763.12	1182.43	1038.68	635.03	528.56	448.42	0.001071659	-1.01
C1_14200W_A	23.07	28.09	47.82	8.48	10.02	35.86	0.151064208	-1.01
C3_04960W_A	81.74	17.35	43.46	13.88	26.58	34.94	0.134826287	-1.01
C7_02810W_A	82.72	72.57	59.54	87.30	11.99	15.27	0.243473563	-1.01
CR_07890W_A	205.03	186.61	163.78	105.97	88.71	103.33	9.47E-10	-1.01
C2_07690W_A	1.92	1.99	2.06	0.88	1.07	1.24	0.004851358	-1.01
C2_02370C_A	245.27	300.58	320.12	171.04	147.93	147.16	5.72E-06	-1.01
CR_04760C_A	6.42	6.30	6.98	4.37	2.94	3.28	0.000145511	-1.01
C1_09750W_A	21.31	21.70	23.29	31.40	1.41	2.50	0.55517551	-1.01
C1_01420C_A	48.82	51.24	39.07	25.90	23.39	25.11	2.89E-07	-1.02
C7_02160W_A	2.05	1.25	2.02	0.90	0.84	1.09	0.00191967	-1.02
C1_09220W_A	52.58	60.27	56.46	35.81	29.00	25.71	1.75E-05	-1.02
C1_05770C_A	29.40	35.10	35.70	18.49	17.08	18.00	2.67E-07	-1.02
C3_06110C_A	28.84	29.88	9.73	7.19	14.01	15.26	0.074240967	-1.02
C2_04460W_A	59.12	64.25	57.30	36.10	23.15	37.51	8.81E-05	-1.02
C2_06320W_A	12.18	11.58	10.07	6.11	5.98	5.92	7.30E-10	-1.02
C1_11700C_A	109.05	145.70	196.94	85.64	73.09	83.54	0.001080344	-1.02
C7_00260C_A	18.12	14.62	17.68	7.67	16.88	1.75	0.213069504	-1.02
C2_10450W_A	28.33	38.78	35.71	2.93	27.03	25.02	0.222263424	-1.02
C2_04350C_A	1.79	1.51	1.21	1.11	0.61	0.67	0.018195056	-1.02
C6_03410C_A	43.40	33.09	36.84	19.19	17.05	23.96	7.72E-07	-1.02
C6_01080C_A	6.38	3.42	3.44	1.78	2.78	2.42	0.018685843	-1.02
C6_02870W_A	57.39	68.77	63.09	33.00	31.97	35.84	6.81E-08	-1.02

CR_03540W_A	2.87	1.78	2.76	1.55	1.29	1.05	0.019652779	-1.02
C2_03540W_A	37.96	26.69	31.86	18.01	15.47	17.59	7.61E-08	-1.03
C1_09470C_A	31.10	31.95	31.11	18.21	15.52	16.19	3.84E-09	-1.03
C7_03270W_A	14.08	17.40	14.67	10.52	7.43	6.52	0.000899353	-1.03
CR_03400W_A	4.79	4.81	4.96	3.16	1.66	2.89	0.001403489	-1.03
C4_05360C_A	0.40	0.78	0.87	0.11	0.13	0.84	0.390695902	-1.03
C1_12060C_A	3.96	3.85	3.37	1.62	2.18	2.03	0.003686166	-1.03
C2_07790C_A	2.27	3.63	1.80	1.61	0.90	1.51	0.07703147	-1.03
C4_02410C_A	293.73	328.16	370.38	172.78	158.58	194.56	1.81E-07	-1.03
C4_04050C_A	208.40	440.05	262.35	205.04	125.79	155.18	0.013953101	-1.03
C1_06670W_A	20.23	10.96	15.24	8.76	7.41	8.13	0.000215113	-1.03
C4_00140C_A	133.13	135.74	133.23	72.71	82.71	55.90	1.99E-05	-1.04
C1_02020W_A	12.23	12.92	15.69	6.78	7.34	7.37	5.55E-05	-1.04
C3_07910W_A	7.65	8.50	9.77	5.29	6.77	1.43	0.120701432	-1.04
C6_01980C_A	40.73	35.66	56.99	22.82	17.18	30.62	0.002277046	-1.04
C4_01530C_A	193.79	152.11	163.42	80.73	93.66	92.80	1.92E-10	-1.04
C7_01040C_A	3.27	5.98	5.87	2.76	2.65	2.59	0.006953807	-1.04
C1_13140C_A	356.28	430.09	303.62	171.36	184.14	220.52	9.13E-06	-1.04
C2_06640C_A	27.87	23.53	20.61	12.24	12.54	13.00	1.48E-09	-1.04
C3_01730C_A	45.91	58.33	61.67	16.32	34.49	37.04	0.010037441	-1.04
C1_02450C_A	3.77	1.68	2.13	1.20	1.08	1.67	0.088889621	-1.04
C5_04320C_A	18.08	13.12	22.51	10.15	5.68	12.50	0.008692539	-1.04
C5_02330W_A	44.90	55.72	64.41	30.23	29.04	27.62	8.73E-05	-1.04
C5_04580C_A	9.10	6.15	9.18	3.71	4.40	4.59	0.001236295	-1.04
C1_03490W_A	56.22	63.56	73.67	35.91	31.41	34.53	1.02E-06	-1.04
CR_02270C_A	7.14	7.08	8.96	3.65	4.19	4.27	0.000289351	-1.04
C1_04290C_A	233.64	284.09	303.35	167.48	137.37	126.60	2.05E-05	-1.04
C2_07260C_A	338.68	526.48	325.78	244.74	233.19	147.01	0.005505369	-1.04
C6_03350C_A	11.87	10.33	10.22	3.89	6.38	6.68	0.000388211	-1.04
CR_07060C_A	131.58	115.43	92.90	55.80	60.95	60.66	5.57E-08	-1.05
C1_11020W_A	10.62	8.61	7.96	4.53	4.55	5.09	9.60E-08	-1.05
C6_02650C_A	3.86	5.09	5.95	3.94	2.29	1.53	0.038607977	-1.05
C2_02570W_A	6.69	4.40	4.92	3.56	2.77	1.97	0.004258742	-1.05
C6_01750C_A	18.13	15.89	13.73	8.41	7.37	9.12	9.84E-07	-1.05
CR_03110W_A	5.47	5.93	7.20	3.42	3.78	2.51	0.001112329	-1.05
C6_01330C_A	1.83	2.12	1.48	0.81	1.24	0.73	0.072540066	-1.05
C2_01270W_A	5.79	3.69	6.40	1.66	3.17	3.38	0.018019968	-1.05
C7_03820C_A	11.70	12.51	5.07	3.59	5.53	6.01	0.041482034	-1.05
CR_08300C_A	64.38	39.99	38.53	27.90	19.66	26.44	0.000244174	-1.05
C2_00210W_A	315.65	772.77	992.31	704.59	226.14	158.33	0.169788593	-1.06
CR_02330C_A	1.67	0.75	1.84	0.43	0.96	0.78	0.108950746	-1.06
C2_03920C_A	1.52	0.81	1.30	0.49	0.65	0.70	0.009176245	-1.06
C1_02800W_A	2.46	3.37	1.58	2.00	1.20	0.66	0.138126988	-1.06
C7_03420C_A	12.08	9.92	8.29	4.92	5.90	4.78	2.07E-05	-1.06
C5_01080C_A	31.88	27.38	31.60	18.99	13.22	14.71	2.12E-06	-1.06
C1_04320W_A	109.96	85.15	102.39	45.49	50.17	57.76	3.31E-09	-1.07
C1_14180W_A	14.04	11.57	18.34	5.97	8.47	8.23	0.0004142	-1.07
C1_06010W_A	9.59	7.12	6.56	2.49	4.25	5.22	0.005987059	-1.07
C5_01250W_A	17.53	17.51	20.01	9.87	9.52	8.97	2.93E-09	-1.07
C1_08430W_A	223.40	191.99	222.19	111.82	102.76	113.18	1.95E-15	-1.07
C4_06100W_A	10.89	13.46	11.53	7.24	6.14	5.11	4.87E-05	-1.07
C3_07560W_A	55.12	55.91	60.78	24.01	33.60	30.75	7.70E-07	-1.07
C3_01980C_A	62.07	96.63	129.57	71.49	40.03	37.33	0.024816829	-1.07
C3_01830C_A	4.46	3.65	4.15	1.49	2.18	2.58	0.000860199	-1.07
C3_05510W_A	3.72	4.00	2.09	1.55	1.72	1.75	0.003737548	-1.07
C1_07530W_A	16.49	14.98	16.26	6.39	10.05	8.24	0.021620683	-1.07
C3_00100W_A	25.27	16.59	13.28	8.64	11.35	7.93	0.002733538	-1.07
C5_01050C_A	6.04	8.65	10.86	6.91	3.21	2.98	0.052302392	-1.08
CR_03960C_A	6.03	5.33	4.91	3.04	2.68	2.60	2.17E-06	-1.08
C3_07450C_A	19.00	21.32	21.13	10.96	11.05	9.34	8.71E-07	-1.08
CR_03440W_A	5.21	4.43	3.85	2.01	2.72	2.06	0.002003886	-1.09
C6_03540W_A	19.60	33.51	40.56	15.59	12.72	19.95	0.005810906	-1.09
C6_00750C_A	3503.74	5461.53	4803.08	2462.25	2315.04	2272.76	5.31E-05	-1.09

C2_04190C_A	41.28	59.22	39.92	24.06	25.01	22.50	6.11E-05	-1.09
CR_10350C_A	356.05	344.21	508.25	154.59	214.50	245.31	0.000283921	-1.09
C2_10360C_A	4.69	6.24	7.54	2.85	2.38	4.11	0.005249596	-1.10
C3_02150C_A	9.44	7.36	11.73	3.97	4.85	5.54	0.000289388	-1.10
C4_04530C_A	5.14	4.78	6.79	3.27	2.64	2.50	0.000229773	-1.10
C4_06860C_A	49.66	52.70	64.48	29.66	33.29	20.78	0.000388197	-1.10
C3_00160C_A	31.44	14.12	32.64	26.68	4.08	8.24	0.163949246	-1.10
CR_05510W_A	5.60	10.77	5.40	3.69	3.65	3.69	0.005425306	-1.11
C2_09590C_A	15.15	24.83	28.63	13.94	10.81	9.87	0.003504777	-1.11
C5_01500C_A	4.42	4.01	3.30	1.61	1.93	2.34	1.52E-05	-1.11
C6_02160W_A	6.26	4.01	5.05	2.53	1.94	3.19	0.000479135	-1.11
C4_05390W_A	28.53	65.68	70.69	19.05	22.17	43.05	0.038475628	-1.11
C6_00470C_A	145.29	197.29	181.79	88.83	92.09	82.26	1.68E-06	-1.11
C3_00550C_A	148.13	118.01	121.21	63.97	63.52	65.26	1.78E-15	-1.11
C1_10810W_A	1.55	1.88	1.47	0.96	0.79	0.59	0.064239362	-1.11
C2_10440C_A	14.19	14.58	13.17	0.75	10.86	9.29	0.218757557	-1.12
C3_02320W_A	119.95	81.80	114.13	50.66	53.68	52.21	4.07E-08	-1.12
C2_10320C_A	1.53	0.77	1.54	0.28	0.32	1.27	0.175940439	-1.12
C1_00290W_A	16.53	17.54	25.45	14.92	10.14	4.41	0.039072785	-1.12
C7_04040C_A	64.16	85.34	93.82	38.60	35.34	48.08	2.61E-05	-1.12
CR_04480C_A	49.06	57.71	67.11	34.62	29.39	22.17	0.000148706	-1.13
C1_03810C_A	1.57	2.35	0.96	1.49	0.55	0.37	0.171171177	-1.13
C3_03440C_A	12.22	10.07	11.48	6.87	5.09	4.72	6.94E-06	-1.13
C1_08760W_A	7.79	10.81	10.51	5.80	4.25	4.37	0.000362353	-1.13
C6_01930W_A	13.33	15.08	25.10	9.16	6.86	10.62	0.003428343	-1.13
C5_03390C_A	38.88	49.85	50.67	22.16	25.15	21.62	4.26E-06	-1.13
C3_04060C_A	1421.06	1344.05	1475.13	726.08	687.05	679.18	7.23E-17	-1.13
C2_10870W_A	35.32	49.06	40.58	19.99	17.25	24.92	2.07E-05	-1.13
CR_07640C_A	21.57	20.61	20.16	9.24	11.19	10.20	3.27E-08	-1.13
C3_05350C_A	1.38	0.97	1.19	0.59	0.65	0.49	0.007953742	-1.14
CR_04500C_A	30.14	28.99	29.48	13.43	14.55	15.55	7.90E-14	-1.14
C2_07580W_A	10.96	13.53	17.31	6.87	6.30	7.44	8.79E-05	-1.14
C2_07910C_A	6.86	16.82	23.24	11.36	3.90	7.95	0.129420626	-1.14
C5_04330W_A	131.71	140.91	168.42	56.86	78.54	81.17	7.32E-06	-1.14
C1_12370W_A	31.05	20.40	28.90	9.79	15.52	13.75	0.000238934	-1.14
C5_05510C_A	6.74	7.46	7.21	4.85	3.33	2.25	0.018362552	-1.14
C1_04880C_A	7.23	6.40	6.14	2.77	3.30	3.36	0.001606486	-1.14
C2_05050C_A	10.19	12.75	11.69	4.93	6.01	6.00	2.27E-06	-1.14
C3_05130C_A	2.87	2.26	2.02	0.88	1.52	1.06	0.007147121	-1.15
C1_13720W_A	1.80	2.56	1.52	1.05	0.86	0.96	0.003744087	-1.15
CR_10630W_A	11.92	14.77	12.40	4.15	7.11	7.78	0.000805501	-1.15
C1_04190C_A	8.06	9.09	9.00	3.90	3.23	5.60	0.000135646	-1.15
C5_00140C_A	25.18	47.03	21.28	23.54	0.89	22.07	0.296289404	-1.15
C6_03310W_A	25.35	23.21	19.94	10.76	11.77	10.66	1.66E-10	-1.15
C1_08900W_A	6.66	5.67	7.25	2.69	3.69	3.08	8.84E-06	-1.16
C5_01270W_A	5.98	7.06	7.77	3.46	3.54	3.09	4.96E-06	-1.16
CR_07580C_A	3.11	2.22	2.42	1.11	1.28	1.34	1.50E-06	-1.16
C5_04800W_A	37.62	31.28	32.52	15.79	16.66	16.49	1.33E-15	-1.16
C3_07160W_A	1.73	2.11	2.51	1.29	0.87	0.87	0.006828863	-1.16
C1_06320W_A	4.40	4.65	3.75	1.93	1.89	2.34	6.43E-07	-1.16
C4_06990W_A	0.46	0.83	1.21	0.44	0.34	0.42	0.064093726	-1.16
C4_01690C_A	47.43	52.10	46.85	23.64	22.58	24.27	1.64E-13	-1.17
C2_01540W_A	18.27	22.25	24.13	14.97	3.81	12.57	0.035791272	-1.17
C2_09480W_A	4.30	3.25	2.80	1.22	1.91	1.76	0.004425702	-1.18
C2_04510W_A	6.65	3.61	1.18	0.92	1.74	2.74	0.140326716	-1.18
C2_09290W_A	5.52	4.81	13.27	4.73	3.82	2.59	0.0911897	-1.18
C1_05080W_A	4.61	3.18	3.20	1.58	1.63	2.03	5.81E-05	-1.18
C7_03790W_A	4.97	5.38	3.24	2.25	1.79	2.40	0.000863043	-1.18
C4_02950C_A	13.57	18.39	18.45	5.97	3.98	14.48	0.047197093	-1.18
C2_01430W_A	62.31	76.93	114.18	42.61	39.71	39.02	0.000246023	-1.18
C1_09430W_A	1.60	2.43	2.67	1.14	0.83	1.21	0.002151534	-1.18
C1_00270W_A	51.91	42.22	52.69	24.80	24.74	20.12	2.23E-08	-1.18
C4_06480C_A	15.52	12.86	13.20	5.89	7.99	5.75	2.29E-06	-1.19

C2_02030W_A	221.89	271.22	270.87	131.45	109.76	123.34	5.26E-09	-1.19
C3_06340W_A	36.35	29.58	24.09	11.24	14.71	16.55	1.45E-05	-1.19
C3_02300W_A	3.30	2.83	3.00	1.41	1.25	1.64	2.80E-08	-1.19
C2_02590W_A	203.61	145.85	160.81	99.69	71.88	68.31	2.61E-06	-1.19
C3_06000W_A	6.56	7.03	5.74	4.30	1.99	2.85	0.001407779	-1.19
C1_11140W_A	14.22	17.91	14.34	7.90	6.75	7.34	1.92E-07	-1.19
C1_06210W_A	1.30	1.78	1.56	0.97	0.20	1.01	0.107580493	-1.19
C1_02580W_A	1.92	2.07	2.91	1.10	0.85	1.28	0.002455141	-1.20
C4_05880W_A	21.46	20.19	36.98	9.30	23.32	3.90	0.101374984	-1.20
C3_02680C_A	39.27	36.01	37.82	17.20	17.55	18.44	2.41E-21	-1.20
C7_03050W_A	5.94	15.49	19.49	10.83	2.96	5.66	0.094881842	-1.20
C7_02600C_A	21.17	20.85	19.24	9.87	9.21	9.66	9.04E-19	-1.20
CR_01640C_A	1.83	2.81	1.85	1.33	1.03	0.67	0.022090171	-1.20
C2_02420C_A	24.59	27.98	22.95	10.03	11.44	14.06	5.25E-08	-1.20
C3_04160W_A	7.11	6.27	4.93	2.98	2.45	3.09	3.05E-06	-1.21
C6_01780C_A	10.60	12.35	9.64	6.61	4.25	4.37	7.59E-05	-1.21
C2_09700W_A	3.56	1.90	3.16	1.11	1.80	1.07	0.012204913	-1.21
C5_05200C_A	27.84	2.55	51.87	16.40	10.86	10.77	0.21311848	-1.21
C4_06470W_A	59.10	55.62	57.30	22.65	33.51	23.63	1.66E-06	-1.21
C3_00910W_A	3.86	4.08	3.13	1.76	1.42	1.98	3.30E-06	-1.21
C1_10440W_A	4.30	3.46	3.89	1.98	2.04	1.38	1.95E-05	-1.21
C1_12360C_A	299.58	305.19	367.93	161.96	147.72	142.84	2.38E-10	-1.22
C2_08450W_A	8.76	8.73	9.05	4.22	4.12	3.99	3.30E-15	-1.22
C1_06460C_A	231.14	379.23	507.89	244.22	148.27	130.02	0.00682401	-1.22
C1_14390W_A	23.39	77.41	96.34	44.89	36.73	10.25	0.131262787	-1.22
C3_02860W_A	24.72	23.54	26.83	9.82	14.97	9.92	1.18E-05	-1.22
C1_14040W_A	36.73	22.17	33.86	11.01	17.86	13.77	0.000111198	-1.22
C5_05480W_A	236.99	259.59	292.39	124.05	128.41	113.49	4.60E-10	-1.22
C5_00820W_A	7.53	13.15	14.26	6.32	4.78	5.17	0.001902832	-1.22
C3_07230W_A	8.57	7.80	7.87	5.31	4.81	0.94	0.070776521	-1.22
C1_11570W_A	5.89	6.80	5.34	2.70	4.18	1.39	0.026993233	-1.22
C4_00650W_A	72.64	97.34	99.75	49.52	37.07	38.61	4.59E-06	-1.23
C1_07570C_A	2.22	2.82	3.06	1.20	1.11	1.38	0.000755318	-1.23
CR_03740C_A	71.99	83.88	78.49	38.68	33.47	36.16	9.19E-11	-1.23
C2_06430C_A	17.50	14.54	13.58	6.46	6.60	7.82	9.45E-08	-1.23
C4_03880W_A	2.52	3.30	1.99	1.11	0.90	1.54	0.003190239	-1.23
C6_03000C_A	4.25	7.65	4.20	1.97	3.15	2.26	0.009184665	-1.23
C3_01770C_A	4.82	6.44	6.93	2.82	2.94	2.61	1.95E-05	-1.24
C1_09190C_A	438.47	374.72	442.66	209.83	158.33	208.37	2.41E-11	-1.24
C1_12780W_A	40.86	24.80	35.75	15.07	15.61	15.50	2.38E-07	-1.24
CR_00480W_A	10.07	7.67	8.84	0.49	6.08	5.50	0.157242173	-1.24
CR_01630C_A	2.66	3.82	2.72	1.35	1.26	1.60	0.00015921	-1.24
C2_08870C_A	595.31	650.89	567.58	306.30	260.76	261.46	2.80E-12	-1.24
C2_00080C_A	4.64	17.75	17.64	8.72	3.06	6.85	0.074991022	-1.25
C3_05090C_A	9.65	10.18	10.05	4.82	4.45	4.33	6.97E-11	-1.25
C4_03080W_A	9.33	4.47	6.16	2.63	2.18	4.22	0.002065784	-1.25
C3_05330C_A	1.92	1.32	1.19	0.66	0.30	1.01	0.053871593	-1.25
CR_04880W_A	12.03	11.62	7.28	8.12	3.20	2.64	0.02668543	-1.25
CR_06980W_A	5.40	1.97	3.00	0.77	1.94	1.85	0.073162059	-1.25
CR_10400W_A	35.08	40.96	74.29	23.76	23.05	21.65	0.001119134	-1.25
C6_03630W_A	7.99	5.08	6.06	3.46	1.86	3.31	0.001388137	-1.25
C1_13590W_A	2.10	1.60	1.02	0.04	1.03	1.03	0.222662435	-1.25
C6_04260C_A	13.00	13.38	13.09	8.48	5.13	4.27	0.000681762	-1.25
C4_04090C_A	15.53	13.76	11.33	4.72	6.82	6.66	7.86E-07	-1.26
CR_02650C_A	26.82	17.51	14.20	7.52	7.86	10.80	8.45E-05	-1.26
C4_00290C_A	1.08	0.58	0.66	0.49	0.36	0.17	0.090173508	-1.26
C4_04470W_A	15.02	20.84	19.82	8.17	8.51	8.40	4.24E-07	-1.27
C1_14360C_A	37.69	53.18	52.75	24.01	32.36	7.60	0.033080225	-1.27
C1_08880W_A	14.85	9.94	10.88	5.46	5.16	5.27	1.73E-08	-1.27
C7_01150W_A	0.75	6.96	4.90	4.80	0.74	0.15	0.374864156	-1.27
C1_12120W_A	9.05	6.57	6.16	1.97	3.60	4.10	0.000450266	-1.28
C1_02670C_A	0.30	0.64	0.67	0.29	0.30	0.14	0.075365255	-1.28
C1_13570W_A	7.07	4.47	10.33	3.11	3.62	2.97	0.000932636	-1.28

C4_06530C_A	2.72	2.64	2.57	1.71	0.69	1.11	0.003146177	-1.28
C5_00870C_A	1.98	2.61	3.18	1.05	1.03	1.37	0.000833478	-1.28
CR_01790C_A	4.10	3.16	4.56	1.99	2.19	1.04	0.001933149	-1.28
C3_06140W_A	15.45	12.60	17.26	6.86	6.43	6.83	2.12E-09	-1.28
C4_03000C_A	30.02	25.78	39.23	12.89	14.84	14.35	6.07E-07	-1.28
CR_03770C_A	191.06	651.76	637.22	389.43	138.49	137.58	0.065858855	-1.28
C5_04220W_A	1.20	0.72	1.36	0.03	1.37	0.00	0.498110805	-1.28
CR_10040W_A	116.90	87.08	92.25	50.84	41.10	38.69	1.18E-08	-1.29
C7_02060W_A	26.35	21.34	17.49	10.39	10.05	8.24	1.07E-07	-1.29
C2_00280C_A	17.17	21.57	17.78	10.23	8.43	6.22	6.15E-05	-1.29
C5_01440C_A	12.28	16.08	17.08	6.74	6.69	6.64	3.84E-07	-1.29
C1_13110C_A	22.79	24.07	19.73	9.20	8.80	11.33	8.43E-12	-1.30
CR_07690W_A	18.80	18.10	18.17	8.49	5.52	10.11	3.63E-05	-1.30
C2_10640C_A	67.16	69.02	64.62	28.35	28.39	31.17	1.84E-17	-1.30
C3_03270W_A	9.78	17.77	15.68	5.01	5.11	8.97	0.003569929	-1.30
CR_00090C_A	93.54	199.31	130.23	39.58	114.18	31.18	0.043939442	-1.30
C2_08970C_A	5.32	9.83	9.96	3.07	3.69	4.26	0.001619523	-1.30
C4_01390W_A	2.76	3.32	2.52	1.78	0.19	1.77	0.128043429	-1.31
C2_09940W_A	230.01	234.47	259.70	124.40	94.74	96.91	2.43E-10	-1.31
C1_09280W_A	9.20	8.17	6.36	3.30	3.08	3.92	3.02E-08	-1.31
C3_04260W_A	34.82	31.75	25.98	13.93	12.50	13.76	2.71E-12	-1.31
C1_06620C_A	6.57	5.45	9.46	3.01	1.71	4.62	0.007833236	-1.31
CR_05790C_A	65.12	43.28	42.98	10.20	35.50	19.17	0.010897592	-1.32
C3_07700W_A	40.20	39.77	45.04	18.13	18.17	17.97	1.47E-14	-1.32
C3_02460C_A	4.63	2.93	2.37	1.95	0.76	1.55	0.013817231	-1.32
C2_07490W_A	117.60	106.90	125.19	27.46	63.42	60.83	0.0005249	-1.32
C6_00340C_A	8.63	9.67	8.69	4.41	4.12	3.13	1.90E-07	-1.32
CR_09020C_A	5.65	18.19	5.17	3.23	4.52	4.94	0.031153659	-1.33
C1_09270W_A	3.91	6.61	3.58	1.83	1.76	2.47	0.000889453	-1.33
CR_02790C_A	4.26	5.76	7.23	3.06	1.81	2.55	0.000997865	-1.33
C1_12190W_A	6.72	6.59	5.69	0.37	3.94	3.80	0.106264788	-1.34
C3_02850C_A	7.28	4.24	3.70	1.92	2.85	1.61	0.002155416	-1.34
C5_00700C_A	15.28	15.54	12.25	7.49	4.87	6.03	5.45E-07	-1.34
C1_08240C_A	39.29	39.62	43.16	18.57	16.17	17.35	2.19E-15	-1.34
C6_04490W_A	27.15	35.21	30.59	13.82	12.67	13.22	3.87E-10	-1.34
C1_10570C_A	0.98	0.77	0.88	0.40	0.25	0.44	0.005161545	-1.35
CR_08520C_A	18.65	26.94	24.10	12.60	5.84	11.40	0.001129352	-1.35
C1_13130C_A	3.50	2.06	2.88	0.87	1.41	1.25	0.000341545	-1.35
C1_14220C_A	2.69	2.12	1.96	1.00	0.21	1.65	0.074001392	-1.35
C1_04510W_A	1.04	2.11	1.56	0.69	0.56	0.73	0.009176245	-1.36
C4_01010C_A	7.05	5.73	9.52	3.14	2.50	3.72	0.000157399	-1.36
C2_08790W_A	9.15	10.38	8.84	4.18	3.47	4.33	1.79E-10	-1.36
C1_11870W_A	13.73	14.18	16.97	6.08	6.44	6.38	2.94E-11	-1.36
CR_08000C_A	151.50	154.96	154.12	67.38	63.14	63.59	3.76E-23	-1.36
C5_04810W_A	775.21	609.58	568.76	344.81	289.99	179.11	3.41E-05	-1.36
C1_10930C_A	28.66	33.06	36.19	15.86	12.10	13.31	3.16E-09	-1.36
C2_08390W_A	51.80	47.78	52.54	21.74	18.14	24.11	1.65E-15	-1.36
C3_03140C_A	9.75	5.21	10.73	1.44	3.86	5.46	0.017971467	-1.36
C2_04810W_A	3.31	1.71	4.11	1.90	0.89	0.95	0.029708119	-1.36
C1_10580C_A	56.85	25.16	43.36	11.54	19.72	20.72	0.001145369	-1.36
C1_04000C_A	12.09	8.12	7.13	3.49	3.78	4.08	1.22E-06	-1.37
C4_00230W_A	33.36	47.15	46.66	18.12	16.49	18.67	1.36E-07	-1.37
C4_06960W_A	15.28	16.68	14.25	7.05	8.82	3.32	0.003357916	-1.37
C2_02400W_A	10.40	13.41	12.73	4.66	5.95	4.57	3.00E-07	-1.38
C1_09930W_A	2.71	1.69	3.23	0.68	1.02	1.38	0.008794943	-1.38
C1_13600W_A	1.90	1.18	1.08	0.52	0.71	0.45	0.019457343	-1.38
C1_10820C_A	45.37	45.41	48.11	20.23	18.92	18.47	5.52E-19	-1.38
C4_03250C_A	7.44	13.32	7.65	5.80	2.54	3.48	0.004852432	-1.38
C7_03350C_A	19.95	34.27	13.87	11.59	8.83	7.84	0.001995513	-1.39
C2_07200W_A	19.36	22.09	37.37	11.31	5.15	16.35	0.010831017	-1.39
C2_08280W_A	5.82	4.28	8.39	1.41	5.45	0.62	0.118920471	-1.39
C5_03780C_A	14.31	25.46	18.43	10.90	6.48	6.73	0.000670969	-1.39
C1_06440C_A	2.86	1.70	1.85	0.82	0.77	1.02	3.95E-05	-1.39

C3_03560W_A	21.79	11.52	10.76	5.92	6.03	5.97	3.97E-05	-1.39
C2_02910W_A	9.18	12.66	12.26	4.86	3.78	5.38	0.000444757	-1.40
C1_12810W_A	13.85	7.74	10.82	4.50	3.17	5.51	5.90E-05	-1.40
C2_05110W_A	20.10	70.32	59.56	11.71	36.70	13.41	0.046896121	-1.40
CR_03880W_A	32.98	25.99	26.48	13.92	10.83	10.02	3.19E-10	-1.40
C6_04130C_A	46.96	43.41	44.86	17.83	20.21	17.01	3.97E-18	-1.40
C3_05810C_A	0.60	0.52	0.40	0.18	0.13	0.30	0.020845704	-1.41
C2_01000W_A	3379.10	3429.78	3704.07	1506.23	1481.34	1282.52	6.93E-16	-1.41
C7_03460W_A	1.45	2.92	2.12	1.46	0.81	0.33	0.042346825	-1.41
CR_04070W_A	59.18	65.23	68.25	29.53	23.48	25.25	5.44E-12	-1.41
CR_03920C_A	10.13	13.70	13.47	4.50	4.81	5.89	4.35E-08	-1.41
CR_00880W_A	4.52	2.51	3.61	2.22	1.41	0.62	0.017087783	-1.42
C2_00110W_A	38.72	33.94	37.32	16.41	10.34	17.65	6.99E-08	-1.42
C3_03450C_A	3.72	1.69	2.56	1.04	0.76	1.34	0.001021721	-1.43
C2_05580W_A	16.15	12.11	11.43	5.42	5.13	5.30	8.04E-14	-1.43
C4_04190C_A	0.42	0.65	0.52	0.29	0.17	0.17	0.013784469	-1.43
C3_03610W_A	19.15	13.16	14.81	8.09	5.68	4.97	1.38E-06	-1.43
C6_00380C_A	11.19	16.38	15.90	7.41	6.48	3.46	0.000664049	-1.43
CR_01340W_A	0.65	0.84	0.86	0.56	0.14	0.22	0.05765423	-1.43
CR_02060W_A	95.63	92.58	89.38	40.27	36.72	33.75	2.85E-18	-1.44
C2_07360W_A	5.78	4.57	3.15	3.79	0.76	0.77	0.067244572	-1.44
C6_01410C_A	24.63	27.55	32.62	13.76	9.32	10.85	6.15E-08	-1.44
C4_03570W_A	5.38	4.90	6.04	2.61	2.04	1.85	8.15E-08	-1.44
C4_00700C_A	20.61	18.81	32.35	9.29	9.66	9.67	2.46E-06	-1.44
C3_05580C_A	24.71	18.25	43.16	13.71	10.26	10.32	0.000351207	-1.44
C1_10110W_A	2.02	1.20	1.27	0.53	0.65	0.59	0.000271893	-1.44
C4_05000W_A	2.78	1.20	1.37	0.35	0.95	0.76	0.023976271	-1.44
CR_02940C_A	3.84	5.23	7.78	2.30	3.08	1.28	0.006540054	-1.44
C1_11480W_A	1.35	3.18	3.20	1.19	0.62	1.25	0.011084127	-1.45
CR_01800C_A	12.24	8.83	11.85	2.56	6.56	3.76	0.001004368	-1.45
C2_10300C_A	3.58	2.48	1.94	0.52	0.91	1.70	0.007453263	-1.45
C1_06000W_A	4.86	3.32	2.69	1.18	2.11	0.94	0.002550372	-1.45
CR_02640W_A	23.18	21.05	20.99	7.77	8.42	9.56	1.56E-20	-1.45
C1_05880W_A	15.50	18.32	18.15	7.54	6.82	6.20	4.30E-10	-1.45
C3_00440W_A	6.92	6.66	8.01	4.23	0.41	3.90	0.079857982	-1.45
CR_10580W_A	116.54	159.66	142.78	58.11	57.80	49.95	9.34E-10	-1.45
C1_08590C_A	204.55	199.99	237.84	87.22	84.77	81.10	4.79E-18	-1.46
C4_04830W_A	14.80	13.44	15.12	5.26	6.42	5.28	1.88E-13	-1.46
C3_03460C_A	1.17	1.21	0.93	0.41	0.45	0.38	0.017215192	-1.46
CR_10570C_A	69.00	70.87	69.82	24.67	29.49	28.11	5.65E-19	-1.46
CR_00580W_A	142.30	125.75	80.13	26.06	48.59	61.54	0.000322022	-1.47
C5_05060C_A	2.89	2.46	2.62	1.54	1.04	0.43	0.030184622	-1.47
C7_03860W_A	70.57	167.13	113.24	49.92	37.25	52.03	0.000287024	-1.47
C4_05430C_A	22.28	24.80	24.58	8.52	9.67	9.78	3.79E-16	-1.47
C2_00840W_A	6.04	7.24	7.41	2.95	2.41	2.72	1.96E-09	-1.47
C7_04210C_A	290.68	187.39	271.23	28.15	120.83	142.27	0.01500929	-1.47
C2_06720W_A	12.75	7.83	7.75	2.04	2.52	6.44	0.005848629	-1.48
C1_08170C_A	33.19	25.91	22.19	10.72	9.44	11.23	1.20E-12	-1.48
C1_10350C_A	21.29	20.36	18.22	9.77	6.00	7.44	4.03E-09	-1.48
C2_04360W_A	11.71	16.39	10.53	6.56	0.60	8.06	0.084119457	-1.48
C4_01230C_A	10.66	10.51	11.69	4.30	3.60	4.72	4.20E-07	-1.48
C1_14160W_A	4.28	2.23	3.61	1.01	1.24	1.62	0.000120181	-1.48
C7_01650W_A	271.47	228.56	241.21	88.21	101.52	96.29	9.22E-25	-1.48
C4_06570C_A	4701.34	6430.82	6291.25	2590.49	1996.45	2191.28	1.96E-09	-1.48
C3_05590C_A	165.87	465.16	327.35	77.38	155.63	142.97	0.003449537	-1.48
C2_00690W_A	8.27	6.10	13.56	4.40	2.84	3.49	0.000537917	-1.49
C3_07220C_A	20.61	16.78	17.52	10.26	10.18	0.27	0.330952463	-1.49
C1_02810W_A	34.40	31.56	31.18	13.04	13.33	10.75	5.35E-16	-1.50
C7_03690W_A	8.71	6.17	6.76	2.47	3.01	2.73	4.98E-07	-1.50
C4_05070C_A	29.61	24.35	28.07	9.40	10.48	11.38	3.50E-19	-1.50
C4_02900C_A	75.45	88.83	87.03	32.36	36.23	27.42	3.72E-11	-1.50
C4_04990C_A	10.39	8.58	7.83	3.19	3.51	3.49	3.45E-12	-1.50
C1_04990C_A	19.93	16.34	17.19	7.30	6.88	6.16	9.08E-15	-1.50

C2_07140W_A	56.40	101.84	99.49	39.04	28.63	31.59	1.60E-05	-1.50
C1_12210W_A	12.38	10.95	13.78	4.26	4.47	5.40	8.15E-13	-1.50
CR_00830W_A	8.04	10.16	9.43	4.76	3.28	2.46	4.65E-05	-1.50
CR_05430W_A	0.96	0.68	1.33	0.31	0.21	0.61	0.039434548	-1.50
C2_06700W_A	4.35	4.18	4.02	2.15	0.65	1.99	0.002559635	-1.50
C1_10690W_A	6.50	9.47	10.59	3.70	2.96	3.50	8.17E-06	-1.50
C5_03470C_A	13.60	8.28	33.21	6.93	6.51	7.59	0.007334791	-1.50
C3_00710W_A	22.29	23.15	20.78	7.87	6.93	10.53	3.77E-12	-1.50
C4_02030W_A	2.96	2.41	2.45	1.08	0.95	0.92	1.23E-10	-1.51
C5_02860C_A	92.87	112.80	129.93	46.46	35.89	46.07	4.52E-10	-1.51
C7_00490C_A	11.16	6.00	18.07	2.40	5.80	5.10	0.008894336	-1.51
C4_01850C_A	4.57	3.60	2.39	0.78	1.24	1.95	0.001403265	-1.51
C6_01950C_A	16.76	22.01	28.77	8.93	8.08	8.77	3.07E-07	-1.51
C3_03800W_A	139.77	193.92	176.53	67.76	69.10	57.03	9.17E-10	-1.51
C5_05320C_A	335.91	352.33	298.08	117.80	131.33	123.68	2.07E-20	-1.52
C3_07210W_A	3.55	2.07	1.88	1.13	1.58	0.00	0.273386764	-1.52
C3_07440W_A	3289.17	4763.90	3858.08	1576.40	1507.79	1435.60	2.01E-10	-1.52
C7_03650W_A	17.45	19.00	18.15	7.90	6.59	6.10	1.01E-11	-1.52
CR_00890C_A	4.50	8.76	10.22	4.71	2.35	1.73	0.026139638	-1.52
C4_00990W_A	10.85	17.65	7.97	5.23	4.17	4.42	0.000106168	-1.52
C4_04410C_A	80.73	59.92	75.17	27.37	27.72	25.77	3.39E-18	-1.52
C4_02770C_A	3.90	4.91	3.65	1.86	1.65	1.15	6.52E-05	-1.52
CR_01020C_A	4.29	3.15	1.98	1.50	0.02	2.04	0.21311848	-1.52
CR_07710W_A	185.68	206.87	137.55	82.47	85.14	29.65	0.001627266	-1.52
C2_05910W_A	18.29	11.72	13.48	4.94	5.73	5.50	2.03E-11	-1.53
CR_04510W_A	2521.81	2676.47	2646.34	1048.67	971.54	918.30	6.26E-22	-1.53
C4_01730C_A	10.44	7.54	8.90	2.63	4.23	3.13	6.80E-08	-1.53
C6_01480W_A	5.60	2.76	3.56	1.68	1.32	1.35	0.001643249	-1.53
C4_06340W_A	10.26	3.30	10.69	3.22	1.61	4.18	0.007786671	-1.53
C3_02900W_A	14.44	12.94	11.85	5.00	6.04	3.52	4.32E-07	-1.53
C3_02870C_A	2.47	2.04	2.83	1.02	0.92	0.76	1.63E-05	-1.54
C1_09300C_A	6.38	9.17	6.64	2.60	2.97	2.68	2.02E-06	-1.54
C7_03830C_A	21.22	19.81	8.25	4.17	5.81	8.18	0.002021581	-1.55
C1_10300W_A	7.59	4.49	1.53	1.86	1.09	2.03	0.013763863	-1.55
C1_03750W_A	132.37	100.25	135.30	45.73	40.32	49.65	1.32E-15	-1.55
CR_09220C_A	1.70	2.22	0.23	0.37	0.24	0.88	0.129736287	-1.55
C1_11370C_A	3.41	4.99	4.22	1.33	1.44	1.88	8.19E-07	-1.56
CR_02850C_A	20.20	12.09	14.82	4.79	6.13	6.20	2.20E-09	-1.56
C1_02970W_A	25.70	198.53	155.80	62.01	30.11	50.43	0.040493	-1.56
C3_02180C_A	12.33	18.37	21.03	8.42	4.52	6.10	4.29E-05	-1.56
C7_04170W_A	5.42	3.73	3.85	0.87	1.69	2.12	0.000468831	-1.57
C1_00800C_A	3.02	3.02	4.47	2.35	0.68	0.82	0.019157997	-1.57
C1_13070C_A	6.06	5.02	6.02	2.81	1.53	1.88	2.78E-06	-1.57
C4_04620C_A	13.42	31.77	38.43	16.05	8.61	6.10	0.006808435	-1.57
C1_01950C_A	20.89	24.09	30.08	8.17	9.77	9.38	1.09E-08	-1.57
C3_00430W_A	3.81	3.07	3.27	1.47	0.38	1.80	0.011685829	-1.57
C6_01870C_A	9.14	11.89	12.16	4.94	3.03	4.18	2.20E-07	-1.57
CR_04770C_A	14.13	7.22	6.98	3.35	3.89	2.91	0.000201122	-1.57
C4_02110W_A	78.12	68.41	61.40	25.04	24.92	25.47	2.67E-21	-1.57
C5_03410C_A	2.11	2.80	3.60	1.21	0.38	1.39	0.017059426	-1.58
CR_10480W_A	56.02	55.14	109.40	24.59	26.38	29.14	2.13E-05	-1.58
C1_13150W_A	1.91	2.08	2.07	0.95	0.56	0.60	0.023022465	-1.58
C4_04590W_A	245.68	184.61	280.88	145.19	1.02	112.58	0.394112495	-1.58
C4_03530W_A	1.25	1.66	2.29	0.50	0.66	0.70	0.000348819	-1.59
C1_13970C_A	21.12	15.22	14.25	5.21	6.44	6.44	1.43E-11	-1.59
C1_08310W_A	54.48	51.65	42.93	15.25	19.12	19.06	4.75E-16	-1.59
C5_04830W_A	27.40	20.69	22.40	8.37	10.10	6.62	6.17E-10	-1.59
C1_14190C_A	52.77	53.16	80.31	7.35	29.61	29.96	0.006217338	-1.59
C5_05450C_A	129.25	150.84	150.69	48.08	56.84	49.57	1.72E-15	-1.59
C2_04340C_A	15.64	10.97	8.82	3.70	4.55	4.22	1.47E-06	-1.60
C4_02070W_A	4.84	6.05	6.92	1.21	2.59	2.54	0.000108532	-1.60
C7_01570C_A	1.48	1.18	1.48	0.45	0.49	0.48	0.000534065	-1.60
C4_01550C_A	31.07	33.46	35.54	15.37	9.72	10.53	1.84E-09	-1.60

CR_03890W_A	14.26	11.97	8.32	3.78	5.38	2.99	9.81E-06	-1.60
C7_01050W_A	1.53	1.24	1.13	0.49	0.68	0.17	0.021312724	-1.61
C7_03600W_A	11.79	8.97	9.39	4.53	3.44	2.66	4.10E-08	-1.61
CR_06770C_A	23.19	40.48	39.87	15.65	9.11	12.27	2.47E-05	-1.61
C2_06800C_A	2.66	3.16	4.33	1.85	0.56	1.17	0.005673588	-1.61
C4_06110C_A	90.12	128.00	99.93	43.10	37.72	32.17	1.23E-09	-1.61
C3_03660W_A	23.49	24.64	18.00	7.80	8.15	7.35	9.69E-14	-1.61
C2_06190W_A	116.51	117.63	115.03	44.84	40.41	37.57	3.34E-23	-1.62
C1_11520C_A	141.76	117.14	144.70	45.72	53.04	42.69	2.41E-16	-1.62
C3_04640W_A	12.31	8.10	6.92	2.43	3.54	3.56	1.86E-07	-1.62
C5_03320C_A	1.05	0.91	1.04	0.08	0.43	0.54	0.032911775	-1.62
C1_05950C_A	13.87	10.85	9.75	3.94	2.94	5.18	4.14E-08	-1.62
C1_05020C_A	11.68	10.26	11.55	4.13	3.29	4.31	7.90E-17	-1.62
CR_09930W_A	26.54	24.77	23.51	8.53	8.65	8.96	2.03E-27	-1.63
C4_04840C_A	12.32	10.93	12.23	3.69	5.15	3.52	2.63E-10	-1.63
C1_11470C_A	5.48	6.45	6.49	1.55	0.66	4.17	0.017098774	-1.65
C3_03640W_A	14.10	13.45	15.12	4.80	4.17	5.69	1.18E-16	-1.65
C4_06890W_A	22.05	28.07	30.52	9.75	9.47	8.55	2.20E-11	-1.65
CR_04680C_A	10.57	7.74	10.85	3.29	2.86	3.69	8.35E-06	-1.66
CR_02440W_A	14.20	11.93	13.17	4.97	4.12	4.22	1.50E-08	-1.66
C2_06020W_A	20.31	13.30	28.28	6.28	7.62	7.12	9.85E-07	-1.67
C1_08060W_A	365.50	398.90	426.43	190.99	142.29	69.57	8.15E-05	-1.67
C6_01090C_A	2.76	2.15	0.98	0.57	0.53	0.88	0.007938842	-1.67
C7_00380W_A	22.59	12.92	35.15	7.27	8.06	8.50	6.99E-05	-1.67
C2_03270W_A	6856.90	8379.59	7680.38	2598.90	2544.50	2621.64	1.73E-21	-1.68
C2_03020C_A	42.10	37.78	37.61	13.27	10.40	15.98	2.26E-16	-1.68
C4_01240C_A	22.17	27.48	27.49	7.11	7.80	11.08	2.04E-09	-1.68
C7_04250W_A	6.88	2.33	2.37	1.46	0.95	1.40	0.002360428	-1.69
C3_01950C_A	55.28	50.28	61.26	16.55	15.88	23.59	3.72E-13	-1.69
C2_04830W_A	4.74	3.92	2.93	1.07	1.33	1.43	6.01E-07	-1.69
C1_01100W_A	14.93	9.19	14.42	4.07	3.99	4.81	4.43E-12	-1.69
C5_01800C_A	21.76	21.38	27.73	8.69	7.67	7.37	1.59E-14	-1.69
C4_06820C_A	59.80	40.40	35.12	14.90	15.81	14.03	3.71E-11	-1.70
C2_01510C_A	2.35	3.05	3.86	0.63	0.91	1.46	0.000974214	-1.70
CR_10100C_A	288.07	325.00	435.90	167.11	89.34	93.61	2.73E-06	-1.70
C3_06280W_A	96.56	66.83	113.18	27.86	33.51	30.14	9.63E-11	-1.70
C1_07030C_A	16.73	17.34	16.88	4.65	5.67	6.48	1.20E-15	-1.71
C1_07470C_A	3.03	2.64	0.94	0.69	0.91	0.54	0.003450417	-1.71
C2_10580W_A	39.80	51.23	56.68	20.12	14.12	14.44	4.72E-09	-1.72
C2_03170W_A	8.70	5.73	8.22	1.33	2.11	3.97	0.000429997	-1.72
C1_00830W_A	15.98	22.56	16.31	6.68	4.90	6.56	4.29E-10	-1.72
C1_03870C_A	1.62	1.50	1.36	0.42	0.51	0.50	4.30E-06	-1.72
CR_03790C_A	4.66	7.42	5.04	1.13	1.51	2.91	0.002180407	-1.72
C6_04410C_A	17.95	28.46	26.93	8.28	7.75	8.12	2.98E-09	-1.72
C5_01380W_A	0.69	1.34	1.59	0.43	0.36	0.38	0.001389523	-1.72
C7_02610C_A	87.67	109.40	127.15	38.35	33.01	35.11	4.54E-13	-1.72
C5_03930C_A	3.42	2.86	1.35	0.55	0.63	1.29	0.002125733	-1.73
CR_00910W_A	1830.13	1638.43	1945.86	213.10	296.39	1269.84	0.010183055	-1.74
C3_05790C_A	15.39	9.80	8.26	2.94	1.80	5.89	0.001833163	-1.75
C2_09730C_A	26.83	27.54	24.56	10.41	6.79	8.09	2.00E-13	-1.75
C5_02460C_A	22.24	34.18	29.82	8.73	8.27	10.78	2.99E-10	-1.76
C1_00840C_A	2.42	2.09	2.46	0.61	0.64	0.95	1.71E-07	-1.76
C3_01180C_A	21.08	14.69	12.80	4.95	5.45	4.98	4.54E-13	-1.76
C2_02860W_A	110.48	129.08	147.98	44.93	36.00	43.33	1.04E-15	-1.76
C6_01060C_A	5.10	3.18	5.54	1.37	1.49	1.51	4.27E-08	-1.76
CR_09240C_A	58.32	58.16	54.05	18.65	7.90	28.15	0.000152095	-1.76
C1_00520W_A	34.37	46.04	42.95	12.65	11.98	14.75	1.68E-13	-1.77
C1_08780W_A	45.42	64.98	56.34	16.91	18.91	17.30	2.29E-13	-1.77
C1_14480W_A	14.71	56.28	57.39	33.66	1.76	5.85	0.109944927	-1.77
C5_00650C_A	64.06	48.15	54.73	13.73	18.09	20.84	5.18E-15	-1.77
CR_10070C_A	113.68	54.67	53.56	27.67	19.79	21.80	8.53E-07	-1.77
C3_07200C_A	8.22	5.18	4.21	2.76	2.49	0.15	0.064030907	-1.78
C3_04480C_A	4.86	4.67	3.66	1.41	1.28	1.45	1.74E-08	-1.78

C1_07230W_A	206.59	204.44	252.96	39.83	97.66	70.98	3.16E-06	-1.78
C2_05100C_A	3.11	11.15	13.40	2.89	2.52	3.40	0.003257397	-1.78
C4_01750C_A	5875.51	7726.26	6882.30	2399.64	2121.74	1927.22	2.52E-15	-1.78
C1_08190C_A	12.25	14.47	12.57	3.93	3.36	5.06	1.47E-14	-1.79
C3_06170C_A	12.29	7.19	11.21	5.79	2.43	1.24	0.004911135	-1.79
C7_00630C_A	1.48	1.32	1.17	0.09	0.53	0.59	0.009913129	-1.79
C4_07090C_A	89.65	82.54	69.58	31.41	26.74	16.34	2.16E-08	-1.80
C7_04060W_A	1.33	1.38	0.96	0.14	0.49	0.50	0.002756847	-1.80
C5_02710W_A	75.38	80.83	121.22	27.62	28.75	29.70	2.21E-11	-1.80
CR_00570W_A	10.04	10.10	6.54	1.57	3.07	3.61	7.43E-06	-1.81
C7_04160W_A	9.03	12.81	7.48	1.96	3.80	3.29	3.74E-06	-1.81
C4_01760W_A	116.19	103.14	91.11	26.69	32.91	35.86	3.55E-20	-1.81
C5_00710W_A	1.21	2.34	1.73	0.51	0.44	0.65	6.15E-05	-1.83
C3_01780C_A	30.42	21.15	31.52	4.94	11.73	8.25	1.42E-05	-1.83
C1_14470W_A	41.57	101.94	101.06	58.48	3.80	12.88	0.171194524	-1.83
C3_04340W_A	107.51	117.09	115.97	36.29	33.76	32.87	4.55E-30	-1.84
C5_03750W_A	3.95	3.99	2.04	0.92	1.08	0.95	0.001730629	-1.84
C1_11080W_A	138.86	117.28	116.14	39.01	34.41	38.40	1.91E-39	-1.84
C2_08380C_A	13.44	9.09	11.36	4.34	2.72	3.07	6.61E-11	-1.85
C6_04190C_A	5.25	3.66	1.61	1.31	0.71	1.11	0.001258654	-1.85
C1_04760C_A	5.56	4.12	9.80	2.33	1.21	2.30	5.23E-05	-1.85
C1_14570C_A	11.29	18.95	21.05	11.40	1.80	2.19	0.02137102	-1.85
C3_01280W_A	55.51	57.46	61.04	15.88	16.86	18.78	1.27E-27	-1.87
C2_03010C_A	172.34	347.96	207.98	80.05	21.26	117.56	0.003090013	-1.88
C2_07420W_A	159.89	190.93	161.56	63.68	54.23	31.87	2.50E-08	-1.88
C1_09540W_A	10.06	6.97	7.28	2.12	2.81	2.13	9.32E-12	-1.88
C2_06940C_A	28.94	28.37	26.96	7.44	4.34	13.00	3.96E-06	-1.89
C3_02060W_A	9.19	14.67	11.72	3.44	2.75	4.07	2.40E-09	-1.91
C5_03970W_A	1.10	0.50	0.54	0.19	0.21	0.19	0.005161545	-1.91
C3_03470W_A	2.38	3.23	2.25	0.78	0.55	0.89	9.57E-07	-1.91
C1_11730W_A	31.92	24.33	23.87	7.25	8.34	7.11	4.38E-23	-1.92
C3_01940C_A	114.29	159.25	152.82	47.04	36.23	38.41	5.30E-14	-1.93
C5_03510C_A	79.60	79.53	73.68	19.89	21.10	24.16	3.16E-39	-1.95
C2_03120W_A	43.53	45.57	47.68	12.57	12.56	13.06	2.54E-39	-1.95
C4_01250W_A	27.49	23.60	24.15	6.24	6.23	8.37	4.98E-20	-1.96
C5_01010W_A	7.44	9.12	9.37	4.06	2.76	0.25	0.024889426	-1.97
C5_01090C_A	20.81	20.75	22.06	6.37	5.41	5.74	9.14E-30	-1.97
C6_01510W_A	6.26	9.11	8.75	2.87	1.83	1.96	1.66E-08	-1.97
CR_01400W_A	813.30	554.28	762.41	189.45	116.79	282.80	3.45E-08	-1.97
CR_09360W_A	12.02	10.68	12.31	3.34	3.39	2.87	3.98E-22	-1.97
C2_09420W_A	13.19	11.73	14.04	1.37	3.62	5.74	9.66E-05	-1.98
C6_01420C_A	20.49	34.36	39.13	10.53	5.80	9.72	4.00E-07	-1.98
CR_02370W_A	8.38	9.41	13.46	3.02	3.14	2.37	8.10E-09	-1.98
CR_02280W_A	28.73	28.13	26.71	7.93	8.20	6.67	3.79E-23	-1.98
C6_04380W_A	56.93	47.12	53.25	14.64	15.13	13.02	1.55E-34	-1.98
C3_04190W_A	14.58	12.77	11.19	3.81	2.50	4.10	3.57E-08	-1.98
CR_09680C_A	3.16	1.89	3.30	1.18	0.49	0.56	8.19E-05	-2.00
C6_04400W_A	6.19	16.69	12.39	3.59	3.46	2.35	0.000425623	-2.00
C1_09250W_A	85.78	103.53	95.25	27.16	24.51	25.39	9.22E-29	-2.00
C2_05860C_A	1.99	2.14	1.52	0.34	0.41	0.72	5.53E-06	-2.01
C3_05610W_A	0.35	1.46	0.97	0.21	0.25	0.27	0.006257329	-2.01
C1_04770C_A	2.29	1.44	25.09	3.81	0.72	3.32	0.093069598	-2.01
C2_04230W_A	227.06	313.66	280.26	74.42	78.18	67.73	2.67E-18	-2.01
C6_03700W_A	10.44	5.04	8.61	2.29	1.83	2.27	2.01E-10	-2.02
C2_02640C_A	103.79	56.25	57.57	15.85	3.65	39.01	0.008085431	-2.02
C1_11720W_A	23.01	28.45	26.96	5.53	7.03	7.91	2.29E-13	-2.04
CR_09010C_A	44.18	66.02	64.48	8.41	16.55	21.24	5.24E-07	-2.05
C6_03320W_A	191.58	227.39	220.17	48.90	58.28	60.23	2.64E-26	-2.05
C3_07590W_A	48.14	88.59	89.93	20.03	19.29	20.17	3.40E-09	-2.05
C3_03860W_A	14.17	22.89	22.57	4.99	5.44	5.06	1.11E-09	-2.06
C1_04900W_A	28.55	27.62	23.76	7.45	8.87	4.20	2.92E-09	-2.06
C6_00960W_A	19.54	19.26	30.48	6.60	5.04	6.22	1.08E-12	-2.07
C6_00440C_A	123.77	180.62	197.05	50.06	45.43	33.83	2.57E-11	-2.07

C2_06460W_A	7.37	7.91	14.77	1.69	2.66	3.36	2.57E-06	-2.07
C2_05460W_A	4306.89	5090.31	4489.96	1274.39	1205.76	1089.63	1.73E-28	-2.07
C3_05990C_A	8.95	7.12	7.06	1.86	1.93	2.08	8.76E-27	-2.08
C1_14580C_A	15.32	18.85	25.17	10.36	2.09	2.67	0.002460774	-2.08
C2_03370W_A	3.38	6.18	7.75	1.65	1.96	0.78	0.000100074	-2.09
CR_00080W_A	40.42	57.20	36.20	9.98	19.47	4.24	0.000336399	-2.09
C3_07360W_A	40.78	50.29	51.13	13.09	12.02	10.69	3.56E-21	-2.10
C7_02830C_A	7.44	10.04	7.30	3.54	1.55	1.12	4.40E-05	-2.11
C2_09400C_A	14.72	17.01	19.10	1.74	4.73	6.19	9.45E-06	-2.12
C3_05310W_A	24.95	24.03	11.56	1.78	8.19	4.89	0.0004142	-2.13
C2_02850W_A	0.93	1.23	1.31	0.19	0.55	0.09	0.006529031	-2.13
CR_02020C_A	27.70	21.89	22.77	5.15	5.84	6.73	2.54E-32	-2.14
C5_00660C_A	42.12	33.84	36.72	9.14	8.00	10.42	2.68E-36	-2.14
C4_01740W_A	11.77	7.10	7.16	2.87	1.13	2.23	1.95E-05	-2.15
CR_06110C_A	4.95	3.72	3.35	0.83	0.88	1.16	1.02E-14	-2.15
C3_03630W_A	2.78	3.69	4.08	1.25	0.90	0.38	3.80E-05	-2.16
CR_09170C_A	14.90	22.70	18.65	5.79	4.10	3.71	2.01E-11	-2.16
C1_09580C_A	3.68	2.58	2.38	0.52	0.45	1.06	1.88E-06	-2.17
C1_00680W_A	4.57	9.47	6.85	1.83	1.52	1.69	1.06E-08	-2.17
C4_06980W_A	1.36	1.07	1.84	0.28	0.36	0.33	0.000305791	-2.18
C1_10610W_A	15.04	11.68	8.88	2.00	3.14	3.28	4.48E-12	-2.18
C7_03720C_A	38.91	34.26	35.93	10.58	10.05	5.09	2.63E-10	-2.18
C6_03360C_A	11.71	3.41	9.19	1.96	2.27	1.38	0.000147184	-2.19
C6_00330C_A	168.19	163.03	201.68	40.81	47.52	37.83	4.33E-26	-2.19
C2_00100C_A	356.39	327.95	351.64	82.00	81.78	81.43	1.37E-76	-2.19
CR_10200W_A	2.05	2.67	3.39	0.52	0.88	0.43	1.98E-05	-2.21
C7_00220W_A	2.97	3.07	4.02	0.50	0.83	0.92	7.29E-08	-2.24
C2_06340W_A	0.42	0.53	0.88	0.18	0.09	0.13	0.000974679	-2.24
C6_03250W_A	26.66	15.78	14.49	4.92	3.51	4.40	1.09E-13	-2.25
C6_02210W_A	14.21	14.88	5.93	2.44	3.46	1.85	3.17E-05	-2.25
CR_04060C_A	252.13	201.72	225.49	49.22	51.19	52.40	3.69E-57	-2.26
C6_02010C_A	226.93	160.23	285.28	55.91	45.04	50.39	4.50E-17	-2.26
C5_02440C_A	7.30	13.82	13.80	1.91	2.74	3.21	6.15E-08	-2.27
C1_03000W_A	3.36	2.94	3.73	0.80	0.73	0.70	1.15E-14	-2.27
C5_04340W_A	11.18	12.30	25.90	4.44	3.29	3.17	3.23E-07	-2.29
CR_01440C_A	537.44	916.12	943.65	210.90	138.37	175.55	4.48E-12	-2.32
CR_02490W_A	19.47	13.08	23.96	3.51	5.05	3.45	8.99E-12	-2.33
C6_00940C_A	1.27	1.21	1.09	0.07	0.38	0.26	0.000911886	-2.34
C1_04660W_A	12.49	8.75	14.38	3.65	1.16	2.78	2.06E-07	-2.34
C3_05290C_A	3.72	5.18	2.75	1.34	0.64	0.48	4.36E-06	-2.35
C4_04230W_A	6.76	1.93	5.52	1.00	1.09	0.85	9.43E-06	-2.36
CR_06270W_A	18.56	16.38	23.00	3.46	4.04	4.42	2.99E-17	-2.38
C1_05620C_A	1.83	2.11	1.39	0.35	0.50	0.23	9.74E-06	-2.38
C5_03530C_A	2.31	2.70	3.18	0.37	0.38	0.87	7.44E-05	-2.39
C3_03360W_A	23.11	22.33	30.61	5.23	5.52	4.80	2.02E-24	-2.40
C1_14630C_A	4.35	4.30	2.36	1.28	0.23	0.65	0.000354737	-2.40
C6_03390W_A	12.16	13.08	14.95	2.95	2.15	3.11	1.09E-19	-2.41
C6_03030W_A	0.83	1.60	0.62	0.19	0.30	0.12	0.00178493	-2.42
C7_00270W_A	46.07	48.90	64.25	6.98	22.02	2.68	0.000870705	-2.42
C2_04450W_A	3.45	5.08	3.74	1.20	0.22	1.03	0.000121572	-2.42
C1_09740C_A	8.26	8.56	8.24	4.53	0.42	0.00	0.076859928	-2.43
C4_04320W_A	105.75	140.23	138.77	33.10	16.93	27.38	2.02E-14	-2.44
C1_14520W_A	87.12	72.12	84.90	36.33	2.38	8.05	0.003604023	-2.49
C6_01110W_A	45.11	36.42	47.35	7.75	6.35	10.42	9.02E-25	-2.51
C1_05000W_A	31.13	25.54	24.54	5.06	4.83	5.38	1.27E-53	-2.52
C4_02100C_A	17.02	10.98	8.62	1.28	2.73	2.70	1.18E-09	-2.54
C1_02630C_A	4.96	6.17	5.57	0.94	1.05	1.03	4.57E-19	-2.56
CR_04220C_A	16.00	12.65	14.93	2.53	2.97	2.43	2.01E-33	-2.56
C4_00860C_A	175.26	330.72	199.58	47.90	38.86	43.18	8.68E-15	-2.57
C5_03310C_A	1.72	1.87	1.55	0.33	0.42	0.15	4.07E-06	-2.57
C1_06870C_A	68.18	69.20	57.70	13.74	8.57	12.75	5.82E-22	-2.58
C4_00440C_A	17.93	24.22	26.36	3.75	5.09	3.41	8.75E-18	-2.60
C3_05080W_A	2.03	2.09	1.54	0.26	0.37	0.33	1.98E-08	-2.63

CR_02570C_A	98.45	94.81	118.80	17.38	18.42	18.18	6.36E-49	-2.64
CR_06550C_A	104.52	93.09	79.18	14.84	17.73	15.02	1.18E-35	-2.65
CR_08510W_A	30.64	18.12	24.48	4.06	3.97	4.40	1.67E-27	-2.66
C1_09720W_A	336.83	399.20	392.12	147.06	29.05	15.33	0.000675371	-2.66
C1_08790W_A	89.02	132.95	114.00	15.78	23.31	18.18	1.05E-20	-2.67
CR_07740W_A	26.61	43.49	30.98	7.20	4.18	4.93	1.43E-11	-2.73
C2_10180W_A	6.86	6.69	5.03	0.84	0.85	1.28	8.94E-17	-2.73
C5_01100C_A	37.61	39.70	44.74	7.58	5.30	6.95	1.90E-35	-2.74
C3_04270C_A	32.54	58.84	57.60	8.15	7.90	8.08	3.07E-17	-2.75
C3_04660C_A	6.89	6.25	6.56	1.25	1.07	0.77	2.99E-18	-2.77
C3_03130C_A	1.47	0.85	1.72	0.02	0.35	0.24	0.00052643	-2.77
C4_02990C_A	33.73	72.65	88.63	13.98	6.83	10.25	3.69E-09	-2.78
C6_03860C_A	2.81	1.56	3.08	0.28	0.23	0.52	1.01E-05	-2.86
C4_03380C_A	22.28	24.92	28.76	4.47	3.50	3.28	8.14E-30	-2.86
C7_00240W_A	41.98	49.97	52.61	9.69	10.37	0.97	0.000150788	-2.87
C4_00430W_A	71.43	101.82	110.65	12.27	15.70	14.02	3.96E-26	-2.88
C2_06470W_A	37.15	48.04	56.62	7.68	5.35	7.94	2.88E-24	-2.88
C1_05710C_A	2.43	2.72	4.26	0.26	0.42	0.66	1.87E-09	-2.89
C1_11530C_A	7.04	6.43	6.41	1.07	1.00	0.75	9.28E-24	-2.92
C3_05170W_A	15.69	19.72	17.89	2.80	2.27	2.40	2.23E-34	-2.95
C3_04350C_A	4.41	6.50	4.28	1.09	0.67	0.35	2.79E-08	-2.95
C4_05540W_A	63.06	61.84	72.88	10.34	9.27	8.03	4.07E-48	-2.95
CR_00740C_A	0.75	22.62	19.54	1.49	2.11	2.44	0.00251985	-2.97
C3_05440C_A	1.11	1.80	1.24	0.06	0.00	0.50	0.013168696	-2.98
C4_05780C_A	3.96	3.95	3.83	0.55	0.57	0.45	5.67E-28	-3.00
C7_01170C_A	23.59	45.21	34.98	11.77	1.63	0.16	0.063947472	-3.04
C2_10240W_A	32.96	23.98	35.48	3.76	4.02	4.02	5.94E-41	-3.07
C6_02480W_A	2.76	4.37	5.30	0.50	0.33	0.70	3.50E-10	-3.09
C3_00600W_A	3.79	4.50	3.54	0.51	0.49	0.46	2.21E-20	-3.11
C5_03480C_A	72.47	69.71	154.46	14.43	11.10	10.21	1.37E-15	-3.17
C6_01990W_A	7.95	14.86	8.44	1.09	1.09	1.50	2.64E-17	-3.20
C5_03440W_A	19.33	13.24	18.32	1.47	2.20	1.93	3.69E-14	-3.21
C7_01560C_A	3.36	2.93	4.73	0.28	0.33	0.58	1.08E-12	-3.24
C3_02390W_A	30.32	20.46	12.42	1.82	2.36	2.68	8.50E-19	-3.30
C3_05600W_A	2.78	9.11	6.28	0.37	0.83	0.68	1.03E-07	-3.33
C3_05840W_A	1.91	1.13	2.60	0.26	0.18	0.13	1.12E-08	-3.33
C6_01180C_A	7.72	7.08	8.55	0.68	0.43	1.02	2.10E-23	-3.52
C7_03300C_A	37.61	29.72	33.57	3.27	2.45	3.56	1.45E-65	-3.55
C1_05960W_A	35.02	34.02	37.46	3.60	3.71	2.48	5.58E-48	-3.55
C2_08050C_A	5.73	5.69	6.60	0.52	0.50	0.60	2.93E-37	-3.56
C4_02080W_A	1.96	3.00	2.04	0.11	0.22	0.24	2.46E-09	-3.61
C4_00450C_A	51.79	48.88	59.50	4.05	5.63	3.91	4.66E-49	-3.66
C2_07280W_A	3.46	2.98	1.74	0.26	0.24	0.15	4.41E-14	-3.70
C4_01800W_A	19.71	33.93	27.94	2.41	3.33	0.97	1.18E-13	-3.70
C1_08410C_A	108.98	116.99	149.11	13.58	8.46	9.17	2.25E-39	-3.70
C7_01380W_A	9.90	3.78	3.30	0.60	0.43	0.30	2.75E-10	-3.72
C2_07570W_A	819.47	798.25	770.17	53.27	69.91	61.53	4.03E-104	-3.80
C3_01540W_A	38.10	50.53	75.40	4.53	3.65	4.48	1.29E-27	-3.81
C3_02070C_A	7.89	2.24	7.35	0.09	0.65	0.50	8.85E-08	-3.85
C3_00320W_A	801.05	638.15	897.76	44.13	49.13	69.82	1.06E-58	-3.96
C1_04650W_A	3.99	5.08	6.16	0.41	0.17	0.42	1.74E-22	-4.02
C7_01390W_A	48.13	22.49	14.04	1.03	0.20	3.98	1.30E-06	-4.08
C5_02790C_A	4.24	4.57	4.83	0.10	0.26	0.28	9.14E-30	-4.43
C1_12910W_A	33.42	16.68	9.47	0.00	1.51	0.65	7.34E-08	-4.71
C2_10160W_A	7.90	6.02	6.43	0.11	0.27	0.22	1.65E-34	-5.10
C2_06970W_A	2.00	2.69	2.48	0.13	0.00	0.00	1.53E-09	-5.75
C6_02100W_A	13.92	9.39	12.92	0.45	0.10	0.00	8.44E-19	-6.02
C5_03500W_A	53.65	60.98	64.13	1.34	0.84	0.70	4.21E-90	-6.07
C3_03570C_A	13.56	1.10	5.80	0.07	0.06	0.02	1.93E-11	-6.91
C4_01340W_A	3.49	3.39	3.54	0.00	0.00	0.00	9.36E-08	-7.53
CR_04210C_A	35.44	48.79	56.61	0.16	0.12	0.12	1.04E-76	-8.46
