# Supplementary material for: The prebiotic inulin affects virulence factor expression in Candida albicans
Source: mBio. 2026 May 14;17(6):e03851-25. doi: 10.1128/mbio.03851-25 (PMC13251390; doi:10.1128/mbio.03851-25)
Supplement: Table S2 — Candida strains. [file mbio.03851-25-s0005.pdf]

Supplementary Table S2. *C. albicans* strains used in this study.

A. Clinical isolates

Isolate Number	Cluster	Infection	Source
SC5314	1	invasive	1
IHEM16614	2	oropharynx	2
J990102	3	vagina	2
AM2005/0377	4	oral cavity	2
CEC711	4	superficial	3
CEC3636	1	commensal	3
CEC3638	3	commensal	3
CEC3543	4	superficial	3
CEC3544	1	commensal	3
CEC3547	11	commensal	3
CEC3596	3	invasive	3
CEC3610	4	commensal	3
CEC3637	3	superficial	3
CEC3662	1	invasive	3
CEC3669	2	superficial	3
CEC3675	4	invasive	3
CEC3690	2	superficial	3
CEC3691	2	commensal	3
CEC4022	2	unknown	3
CEC4103	13	superficial	3
CEC4492	11	environment	3
CEC4510	11	superficial	3
CEC4512	1	superficial	3
CEC4943	13	superficial	3
CEC5028	13	superficial	3
CEC5020	13	superficial	3
CEC5120	11	unknown	3
CEC5255	3	commensal	3

Sources:

1. Gillum AM *et al.* (1984) Isolation of the *Candida albicans* gene for orotidine-5'-phosphate decarboxylase by complementation of *S. cerevisiae ura3* and *E. coli pyrF* mutations. *Molec. Gen. Genet*. 198, 179-182.
2. MacCallum DM, Castillo L, Nather K, Munro CA, Brown AJP, Gow NAR, et al. Property Differences among the Four Major Candida albicans Strain Clades. Eukaryot Cell. 2009 Mar;8(3):373–87.
3. Christophe d'Enfert, Institut Pasteur.

B. Laboratory strains

Strain	Name	Parent	Genotype	Reference
SC5314	SC5314	-	blood isolate	1
CAI4	CAI4	SC5314	*ura3Δ::imm434/Δura3Δ::imm434*	2
SN152	SN152	SC5314	*arg4Δ/arg4Δ, leu2Δ/leu2Δ, his1Δ/his1Δ, URA3/ura3Δ::imm434, IRO1/iro1Δ::imm434*	3
Ca2475	*hcm1*	SC5314	*hcm1Δ::SAT1/hcm1Δ::SAT1*	4
sak1	*sak1*	SC5314	*sak1Δ::FRT/sak1Δ::FRT*	5
NM23	*gpr1 gpa2*	CAI4	CAI4, *gpa2Δ::hisG/gpaΔΔ::hisG, gpr1Δ::hisG/gpr1Δ::hisG-URA3-hisG*	6
CR323	*cyr1* (*cdc35*)	CAI4	CAI4, *cdc35Δ::hisG/cdc35Δ::hisG, pVEC-URA3*	7
CKY157	*czf1*	CAI4	CAI4, *czf1Δ::hisG/czf1Δ::hisG*	8
HLC52	*efg1*	CAI4	CAI4, *efg1Δ::hisG/efg1Δ::hisG-URA3-hisG*	9
GOA31	*goa1*	SN152	SN152, *goa1Δ::URA3/goa1Δ::ARG4*	10
sef1	*sef1*	SN152	SN152, *sef1Δ::CdHIS1/sef1Δ::CmLEU2*	11
tye7	*tye7*	SN152	SN152, *tye71Δ::CdHIS1/tye7Δ::CmLEU2*	11
tpk1 tpk2	*tpk1 tpk2*	SN152	SN152, *tpk1Δ::LEU2/ tpk1Δ::FRT, tpk2Δ::HIS1/ tpk2Δ::ARG4*	12

References:

1. Gillum AM *et al.* (1984) Isolation of the *Candida albicans* gene for orotidine-5'-phosphate decarboxylase by complementation of *S. cerevisiae ura3* and *E. coli pyrF* mutations. *Molec. Gen. Genet*. 198, 179-182.
2. Fonzi WA & Irwin MY (1993) Isogenic strain construction and gene mapping in Candida albicans. *Genetics* 134, 717-728.
3. Noble SM & Johnson AD (2005) Strains and strategies for large-scale gene deletion studies of the diploid human fungal pathogen *Candida albicans*. *Eukaryot Cell*. 4, 298-309.
4. Avelar GM *et al.* (2024) A CO_2 sensing module modulates β-1,3-glucan exposure in *Candida albicans*. *mBio* 15, e0189823.
5. Ramirez-Zavala *et al.* (2017) The Snf1-activating kinase Sak1 is a key regulator of metabolic adaptation and in vivo fitness of *Candida albicans*. *Molec Microbiol* 104, 989-1007.
6. Maidan MM *et al*. (2005) The G protein-coupled receptor Gpr1 and the Galpha protein Gpa2 act through the cAMP-protein kinase A pathway to induce morphogenesis in *Candida albicans*. *Molec Biol Cell*. 16, 971-1986.
7. Rocha CRC *et al*. (2001) Signaling through adenylyl cyclase is essential for hyphal growth and virulence in the pathogenic fungus *Candida albicans*. *Molec Biol Cell* 12, 3631-3643.
8. Brown DH Jr *et al.* (1999) Filamentous growth of *Candida albicans* in response to physical environmental cues and its regulation by the unique *CZF1* gene. *Molec Microbiol*. 34: 651-662.
9. Lo HJ *et al.* (1997) Nonfilamentous *C. albicans* mutants are avirulent. *Cell* 90, 939-949.
10. Bambach *et al.* (2009) Goa1p of *Candida albicans* localizes to the mitochondria during stress and is required for mitochondrial function and virulence. *Eukaryotic Cell* 8, 1706–1720.
11. Noble *et al.* (2010) Systematic screens of a *Candida albicans* homozygous deletion library decouple morphogenetic switching and pathogenicity. *Nature Genetics* 42, 590–598.
12. Cao C *et al.* (2017) Global regulatory roles of the cAMP/PKA pathway revealed by phenotypic, transcriptomic and phosphoproteomic analyses in a null mutant of the PKA catalytic subunit in *Candida albicans*. *Molec Microbiol* 105, 46-64.
